# A socio-ecological framework examination of drivers of blood pressure control among patients with comorbidities and on treatment in two Nairobi slums; a qualitative study

**DOI:** 10.1371/journal.pgph.0001625

**Published:** 2023-03-10

**Authors:** Shukri F. Mohamed, Teresia Macharia, Gershim Asiki, Paramjit Gill

**Affiliations:** 1 Division of Health Sciences and the NIHR Global Health Research Unit on Improving Health in Slums, Warwick Medical School, University of Warwick, Coventry, United Kingdom; 2 Chronic Disease Management Unit, African Population and Health Research Center (APHRC), Nairobi, Kenya; 3 Department of Global Health and Population, Lown Scholars Program, Harvard T.H. Chan School of Public Health, Boston, Massachusetts, United States of America; 4 RTI International, Nairobi, Kenya; 5 Academic Unit of Primary Care (AUPC) and the NIHR Global Health Research Unit on Improving Health in Slums, University of Warwick, Coventry, United Kingdom; ESIC Medical College & PGIMSR, INDIA

## Abstract

Despite the known and effective treatments to control blood pressure, there is limited information on why there are high uncontrolled hypertension rates in urban slum settings. The aim of this paper is to explore the views of treated people with uncontrolled hypertension and other key stakeholders on the facilitators and barriers to blood pressure control among people with comorbid conditions in two Nairobi slums. The study was conducted in two Nairobi slums namely, Korogocho and Viwandani. This study used a qualitative methodology using interviews and focus group discussions. Barriers and facilitators to blood pressure control were explored using the Social Ecological Model (SEM) framework. A total of 57 participants were interviewed for this study. There were 31 in-depth interviews and two focus group discussions among participants with uncontrolled hypertension and with comorbidities. Additionally, 16 key informant interviews were conducted with healthcare providers and decision/policymakers. All interviews were audio-recorded, transcribed verbatim and analysed thematically. This study identified barriers and facilitators to blood pressure control among patients with uncontrolled hypertension at the patient/individual level, family and community level, health system level and at the policy level. High cost of hypertension medicines, the constant unavailability of medicines at the health facilities, unsupportive family and environment, poor medicines supply chain management, availability and use of guidelines were among the barriers reported. The results show that uncontrolled hypertension is a major public health issue in slums of Nairobi and they highlight barriers to blood pressure control at different levels of the socio-ecological model. These findings can be used to design holistic interventions to improve blood pressure control by addressing factors operating at multiple levels of the socio-ecological framework.

## Introduction

Uncontrolled hypertension (UHTN) is an important risk factor for cardiovascular diseases (CVDs) and a leading contributor to death [1,2]. Globally, an estimated 1.28 billion people had hypertension in 2019 and the global age-standardised prevalence of hypertension was 32% in women and 34% in men among adults aged 30–79 years [3]. The highest proportion (1.04 billion—75%) of people with hypertension were from low-and-middle-income-countries (LMICs) while 25% (349 million) were from high-income-countries (HICs) [4]. The highest prevalence (30%) of hypertension is in the African region compared to 18% in the Americas region among those aged 18 years and over [5]. Urbanization is thought to be a key driver for the rise in hypertension in SSA [6] and with urbanization, more and more people are moving to cities and living in slums or slum like conditions with limited health-services available. Currently more than half (55%) of the global population live in urban areas. The UN predicts that this proportion will rise to 68% by 2050 [7]. In 2017, a Lancet article reported intense urban growth over the last 50 years with more than half of city populations living in slums [8].

The increasing trend and high burden of hypertension in LMICs is worrisome particularly because most of the limited resources for healthcare spending is allocated to managing infectious disease burden in these countries. The burden of hypertension among slum populations specifically is high and rising [9]. Barriers to blood pressure control exist at various levels. The Social Ecological Model (SEM) provides different levels to assess barriers in blood pressure control.

Previous literature has shown that economic constraints have been cited as a major barrier to blood pressure control at the individual level while having knowledge and understanding of ones’ own condition are thought to be an important facilitator at this level [10]. Literature supports the importance of family and community in hypertension management. For instance, a study conducted by Flynn and colleagues [11] reported that family members of individuals with hypertension usually helped them with meal preparations, taking their medications and attending appointments. Similarly, a study conducted in Eritrea reported that patients with hypertension highly valued the support they received from their families and community in hypertension care [10]. Health systems in SSA are already overburdened with communicable diseases. Gaps in capacity for implementation of essential non-communicable disease (NCD) intervention have been identified in low resource settings [12]. A study conducted in the slums of Nairobi also found major gaps in staffing, equipment and drugs for handling chronic diseases [13]. Despite the known and effective treatments to control high blood pressure, there is a dearth of information on the drivers of large uncontrolled hypertension rates in urban slum settings [14–17]. The aim of this study was to enrich our understanding on the facilitators and barriers to blood pressure control among people with comorbidities that exist at different levels of the socio-ecological model in two Nairobi slums. The study also explored why treated patients with hypertension still have uncontrolled blood pressure.

## Methods

### Conceptual framework

**Fig 1:**
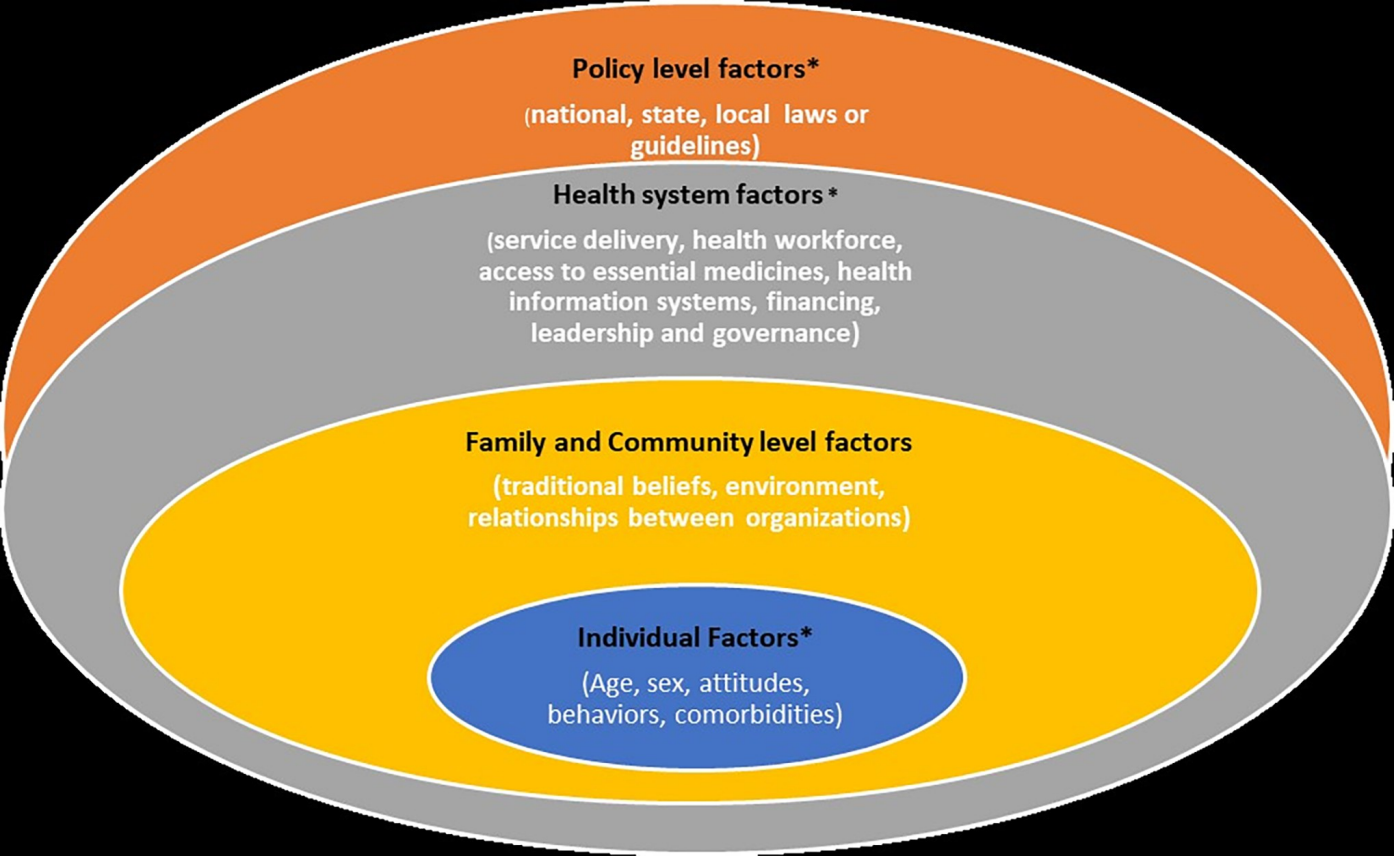
Factors affecting uncontrolled hypertension at the different levels.

This study used the Social Ecological Model (SEM) framework adapted from the Centers for Disease Control and Prevention [18] to understand the multiple levels of factors associated with uncontrolled hypertension and the interactions between the different levels within this system. There are four levels in this adapted SEM: Individual; family and community; health system, and policy/enabling environment (Fig 1). This model takes into account the complex interplay between the different levels and allows for the understanding of the range of factors that put people at risk for uncontrolled hypertension or protects them from having uncontrolled blood pressure. The overlapping levels in the model show how factors at the different levels influence each other. The solutions and gaps in hypertension care can be investigated by assessing these factors at these levels. Uncontrolled hypertension (UHTN) in this study is defined as systolic blood pressure of ≥140 mmHg and/or diastolic blood pressure of ≥90 mmHg in a patient taking anti-hypertensive medication.

### Study site and participants

**Fig 2:**
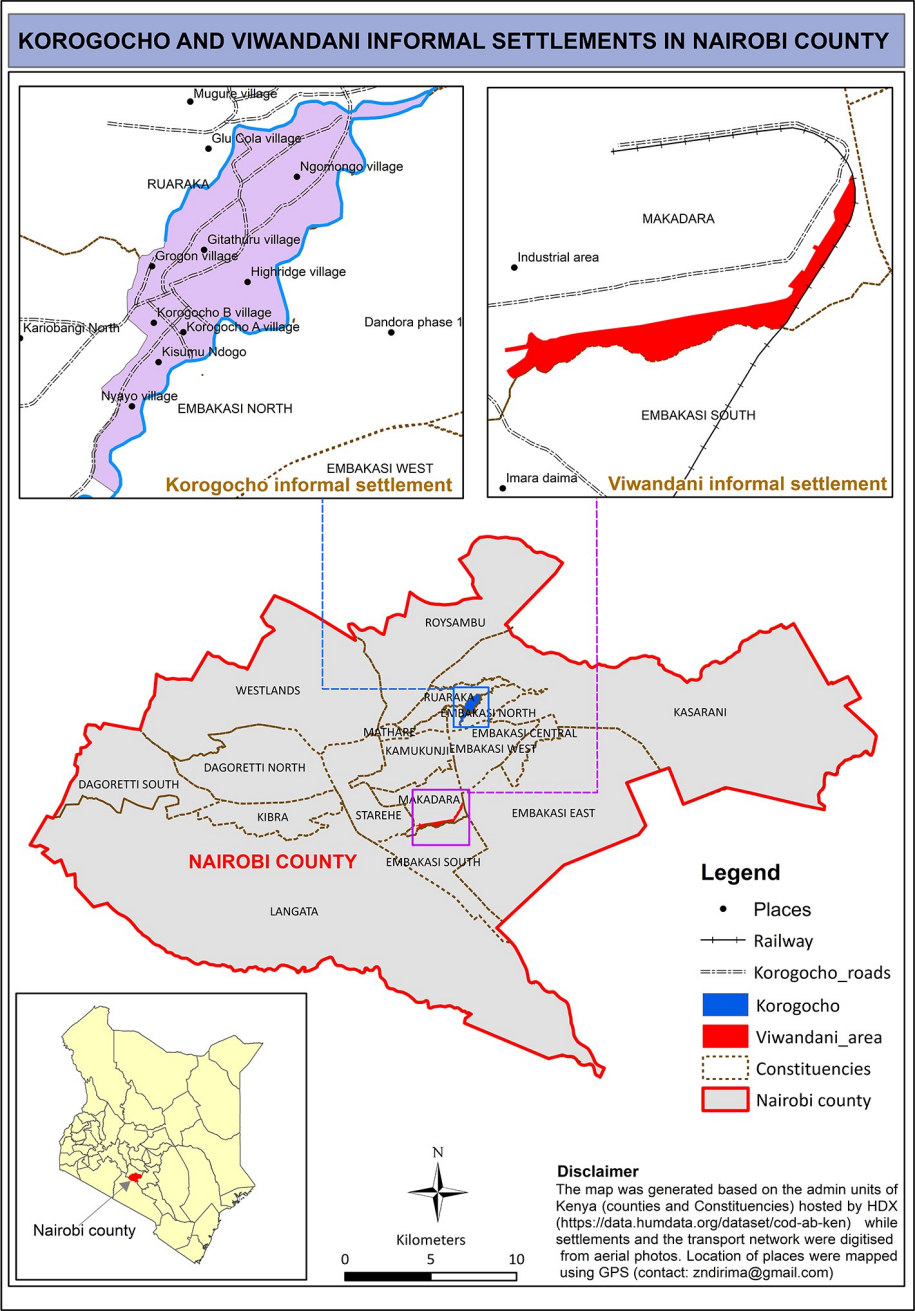
Map with the location of the Nairobi Urban Health and Demographic Surveillance System (NUHDSS) sites (Korogocho and Viwandani).

The study was conducted in two informal settlements or slums in Nairobi (Kenya) namely, Korogocho and Viwandani (Fig 2). The two slums are located on the outskirts of Nairobi City about 10 km from the city center. Viwandani slum is located near the city’s industrial area and it is home to many low income young people working in the industries who are predominantly male. Due to the industrial nature of work that favors employment for men only, a high proportion of married men in Viwandani do not live with their spouses who have been left behind in their rural origins to either farm and or take care of the children. The Korogocho site by contrast is a more established slum settlement with a high proportion of married men living with their spouses and children [19]. The study sites were chosen as the African Population and Health Research Center (APHRC) has been running the Nairobi Urban Health and Demographic Surveillance System (NUHDSS) in these two slums since 2003. The NUHDSS captures routine information on births, deaths and migration from households three times a year. In 2018, the NUHDSS covered 88,798 individuals in 33,462 households (APHRC 2018). The NUHDSS [20] provides a sampling frame for many nested studies including AWI-Gen study [21] from which this current study drew its study participants. The AWI-Gen study collected data on sociodemographic, anthropometric, biomedical and genetic information from 2003 study participants between the ages of 40 and 60 years in the NUHDSS using a cross-sectional survey.

The AWI-Gen study sample was used to purposively sample residents of the two slums with uncontrolled hypertension and comorbid conditions using the participants’ most recent blood pressure measurements (collected in 2018). All participants from the community were adults aged 45 years and older, previously diagnosed with hypertension, and had at least one of the following comorbidities; diabetes, dyslipidemia and overweight/obesity in the 2014–2015 AWI-Gen survey, and were receiving care for uncontrolled hypertension. Healthcare providers in the community and relevant decision/policymakers at county and national level were also approached.

### Study design

This study used a phenomenology approach to gain an in-depth understanding of the facilitators and barriers in controlling blood pressure among patients who are on treatment for hypertension and have a comorbid condition. A phenomenological approach is a form of qualitative enquiry that emphasizes lived experiences of individuals by exploring the meaning of a phenomenon while gaining a deeper understanding of the phenomenon [22]. The main goal of this approach is to identify a phenomena by how it is perceived by those with the lived experiences [23]. The consolidated criteria for reporting qualitative research (COREQ) was adopted in this study.

Data were collected via focus group discussions (FGDs) and in-depth interviews (IDIs). In-depth interviews were used because they provide rich participant views and allow for issues to be explored in more depth [24,25]. FGDs were used to supplement the interviews because they give participants an opportunity to reflect on other participants’ views while building on their views and they give a good understanding about participants’ views on the topic of interest [24,26,27]. IDIs and FGDs were conducted among people with uncontrolled hypertension and comorbidities while on hypertension treatment. Key informant interviews (KIIs) were conducted among key actors shaping hypertension care (healthcare providers, and decision/policy makers).

### Characteristics of study participants

A total of 57 people participated in the study. Thirty-one IDIs (15 in Korogocho and 16 in Viwandani) were conducted. Two FGDs were also conducted; one in each of the two slums among participants with uncontrolled hypertension and comorbidities. Eleven KIIs were conducted among healthcare providers in service provision for hypertension in the two study communities. In addition, five key stakeholder interviews were conducted with representation from the ministry of health; two from the national level and three others were from the sub-county health levels (Table 1). The sample size was determined by theoretical saturation [28].

**Table 1:** Sample characteristics.

Interview type	Participants	Number
**Site 1: Korogocho**		
In-depth interviews (IDIs)	Males	6
	Females	9
KIIs–Healthcare providers	Males	4
	Females	1
Focus group discussions (FGDs)	Males	3
	Females	2
**Site 2: Viwandani**		
In-depth interviews (IDIs)	Males	5
	Females	10
KIIs–Healthcare providers	Males	3
	Females	3
Focus group discussions (FGDs)	Males	3
	Females	3
**Site 3: National/county level**		
KIIs–Decision and policy makers	Males	3
	Females	2
Total		57

#### In-depth interviews and focus group discussions

IDIs and FGDs were conducted among participants with uncontrolled hypertension and comorbidities to understand their experiences and views about their hypertension care. They were also asked about facilitators and barriers to blood pressure control and solutions to the barriers mentioned at each of the SEM levels. Once no new information was emerging from the interviews, data collection was stopped.

#### Key informant interviews with key stakeholders

For the key stakeholders’ interviews (policy/decision-makers and healthcare providers), an initial list of purposively selected study participants was generated with varying representation in sectors. Their selection was based on their role in hypertension care provision in the community or their ability to influence policy and decision making for hypertension care. Snowballing [29] was also used to identify additional key informants during interviews with the initial key informants selected. Key informant interviews were conducted with decision/policymakers to get their views on the challenges in the access and uptake of hypertension care in the study community and what can be done to improve access and uptake of hypertension care while interviews with healthcare providers sought to ascertain the healthcare providers’ prescription practices, conformity and knowledge of national guidelines, and how they treat patients with comorbidities. Both categories of the key stakeholders further provided their views on factors associated with uncontrolled hypertension in the community using the different levels in the SEM framework as a guide.

### Guide development, training of interviewers and pilot study

The initial topic guides were developed from the literature review informed by the conceptual framework. All study participants were asked to describe facilitators, barriers and solutions to blood pressure control at different levels of the adapted SEM (patient, family or community, health system and policy level). The guides were further revised following the pilot study.

Two research assistants collected the data for this study. Both had previous experience working in the community and had prior experience conducting qualitative interviews. They were conversant with the local language (Swahili) and the cultural nuances in the community. The team were trained on the study rationale, objectives, study approach, data collection procedures, note-taking and phone based interviews. They were further trained on research ethics and the study’s informed consent process. The team also reviewed the guides to understand the purpose of each question and the objective it answered.

A virtual pilot study was undertaken outside the study areas to test all the study guides. A debriefing session was held following the pilot exercise to discuss questions that were unclear, had wrong instructions, questions respondents struggled with or questions that were difficult for the participant to understand in all the developed guides. The guides were then revised accordingly before the actual the data collection.

### Data collection

Due to the COVID-19 pandemic, most of the interviews were conducted by phone. The FGDs were conducted in a face-to-face set up that adhered to COVID-19 control measures. The 2018 blood pressure recordings were used for selection into the study. Before the interviews begun, all study participants were asked to complete a brief questionnaire to provide their demographic information. To enhance data quality, the research assistants submitted their summary notes daily and several debriefing sessions were held with them to assess the quality of data that were collected. Data collection took place from June to August 2020 and data collection was stopped when theoretical saturation was reached—no new information was being generated from the interviews [28].

### Analysis

All interviews were recorded. Audio-recordings were first transcribed verbatim by a professional then translated into English by an independent translator. Transcripts were reviewed daily by the research team in order to get a sense of theoretical saturation. All the transcripts were imported into NVivo software (version 12, QSR International) for coding and further analysis.

The analysis was guided by Braun and Clarke’s six steps to conduct thematic analysis [30]. Coding and identification of quotes to go with each theme was done by two independent researchers. All the data were analysed and integrated together in the presentation of the themes. Using the SEM framework, data were examined and four major themes were deductively developed; these were facilitators and barriers to blood pressure control at the 1) patient/individual level, 2) family and community level, 3) health system level and 4) policy level. The solutions to the above barriers are also presented under each theme (Table 2).

**Table 2:** Key themes and subthemes; facilitators, barriers and identified solutions.

Factors	Facilitators	Barriers	Solutions
Individual Level Factors	○ Good understanding of blood pressure management ○ Adherence to medical and lifestyle advise ○ Blood pressure monitoring ○ Stress management ○ Health insurance	○ Poverty, low socio-economic status ○ Inability to adhere to diet ○ Patients comorbidities & age ○ Patient’s perception of physical activity ○ Limited knowledge on blood pressure ○ Unfavourable medication side effects & pill burden ○ Behavioural factors: smoking & alcohol use ○ Denial of hypertension diagnosis ○ Language barrier	○ Resources: income generating activities ○ Free medicine ○ Hypertension knowledge
Family and community level factors	○ Social /family support ○ Supportive social environment ○ Easy access to facilities	○ Stigma and unsupportive family environment ○ No support to older people ○ Social, physical and stressful home environment ○ Limited hypertension knowledge	○ Use of community health workers ○ Attending clinic with care providers
Health System factors	○ Technical capacity of healthcare providers ○ Relationships with care providers ○ Quality of care ○ Facility hours of operation ○ Availability of medication ○ Free medication	○ Inadequate follow ups & lack of appointments ○ Providers heavy workload and bad attitude ○ Low quality of care ○ Limited provider training opportunities ○ Poor supply management system ○ High cost of medication & unavailability of medication (stock outs) ○ Facilities not accepting patient’s insurance ○ Expired medication ○ Facility hours of operation ○ Shortage of healthcare providers & long wait times ○ Limited and worn-out equipment and tests & delays in equipment maintenance and replacement	○ Imparting hypertension knowledge to patients ○ Training & increasing healthcare providers ○ Setting up special hypertension clinics & follow up mechanisms for patients ○ Availing medication, tests and equipment including free medication ○ Removal of taxes on hypertension medicines & medication subsidies ○ Reducing patient wait times
Policy level factors	○ Availability of guidelines	○ Lack of guidelines in some facilities ○ Policies prohibiting hypertension medicines in lower-level facilities ○ No specific budget allocation dedicated for hypertension care ○ Limited evidence to support policy development	○ Research and interventions to inform policies ○ Policy that would allow free medication including policy removing taxation on hypertension medication ○ Availability of care guidelines in each facility

### Ethics

This research study was approved by AMREF (P773/2020) and University of Warwick ethical review boards (BSREC 54/19-20). Informed consent was collected from all participants in accordance with approved ethical procedures and guidelines. For the phone interviews, verbal consent was sought and it was audio-recorded. For the face-to-face interviews, written consent was obtained and this was documented.

## Results

### Organisation of the results

The results from this research are organized to match the various levels of the study’s conceptual framework.

### Perception and experiences of patient level facilitators and barriers

Knowledge, behaviour, practices, and healthcare experiences of residents who had uncontrolled hypertension living in the selected study sites were explored. The findings showed that a patient’s blood pressure control was facilitated by having a fair understanding of what they needed to do. They mentioned adherence to medication, frequent monitoring of BP, salt reduction, physical activity, diet control, weight control and lowering of alcohol consumption as key facilitating components of hypertension management. Monitoring of blood pressure regularly was mentioned and the monitoring ranged from daily, every three days to every three months. A good number of respondents recorded their readings for future reference. Respondents also noted that they knew what their target blood pressure was supposed to be.

*“I was told that I am not supposed to take a lot of salt and again I used to love meat so much but nowadays I eat vegetables a lot but just a little meat because meat is good but not too much of it*. *I used to take a lot of salt and I think that’s what was affecting me so much” [IDI (Viwandani)*, *UHTN participant*, *200715_005*, *female 56]*.

Respondents mentioned managing stress as a facilitator in controlling their blood pressure. They felt it improved their emotional and physical health, which ultimately lowered their high blood pressure. Stress management techniques mentioned included exercising, listening to music, focusing on something calm or peaceful and talking to their friends.

*R1*: *“My experience is staying away from many issues that can make me get angry or make me think a lot*… . *You also need to commit yourself or avoid them because this condition [HTN] gets worse when you engage yourself in many thoughts*. *It becomes worse when you come across something that annoys you” [FGD (Korogocho)*, *UHTN participant (R1)*, *Male 61]*.

Despite the patients’ experiences of how to manage their blood pressure, study participants frequently reported poverty as a significant barrier to blood pressure control in their communities because it restricted their access to medication. Majority of the participants repeatedly reported not getting medicine at the health facilities they visited and that unaffordability of hypertension medications affected their adherence to blood pressure medications. The situation was worsened by the COVID-19 pandemic with many people losing their jobs. Given the reality of low financial resources in the study area where sources of income were limited and wealth status was low, high costs of the medicine were felt to constrain the success of medication adherence. Respondents noted that they would only buy the medications that they could afford and stay without medications on the days they could not afford. Concerns about the cost of hypertension care went beyond the cost of medication to include other associated costs including consultation, testing and transport.

*“The problem that I face is like sometimes I don’t have money to buy drugs and when I go to the hospital I am told that the drugs are not available and the doctor directs you to go buy the drugs at the chemist yet you don’t have money*. *You will just have to stay with your high blood pressure condition because you don’t have money*. *That’s one of the challenges that we face” [FGD (Korogocho)*, *UHTN participant (R1)*, *Male 61*]

Having some sort of health insurance coverage was mentioned as providing access to healthcare. Patients reported getting treatment in some health facilities was facilitated by having health insurance.

*There are some areas where my insurance card helps me and other places I use cash so if I go there then I will not be assisted the way I am supposed to be assisted [IDI*, *(Korogocho)*, *UHTN participant*, *200712_2239*, *Male 58]*.

The few individuals that mentioned having health insurance, mentioned having the National Health Insurance Fund (NHIF). This was reported as the government-initiated health insurance that is mainly accepted at government (public) facilities as well as a few other private health facilities in the community. Even though the public facilities accepted the NHIF, study participants reported that they hardly had medicines in stock. Others also mentioned that the facilities did not accept their insurance because the government was not reimbursing the health facilities for the services they provided. Healthcare providers noted that not having health insurance was a barrier to blood pressure control because the alternative would be to buy the medication though many patients in the community were not able to afford this.

*“*… . *currently as we talk*, *patients are really running away from public facilities because they say even if you have NHIF and not a cash patient you are told to buy medicine even pain medicine*. *So at times you find that you are told to buy*, *you don’t have that money*, *yet your NHIF deduction has been made” [KII (Viwandani)*, *Health care provider*, *200701–0035*, *0426*, *0425]*.

Respondents noted that they were conflicted on whether to spend the little money they had on food or medicine to control their blood pressure. There was a perception among the patients that they had to prioritise eating for survival over buying their medications.

*“I went there and I was told to go buy [medicine] but sometimes I find that I don’t have money to buy drugs*, *I just buy a little flour so that my kids can eat*. *It is hard to choose whether to buy drugs or food for my kids*. *That’s the challenge that I face [IDI (Korogocho)*, *UHTN participant*, *200713_0720*, *female 55]*.

Participants also reported that their low economic status inhibited their ability to follow the recommended diet stating that the diet recommendations did not consider their financial status since they could only afford one meal a day. In addition, there was a challenge when only one individual in the family had hypertension or another comorbidity, thus requiring that there are two budgets to cater for food in the family, which was considered unfeasible.

*“you go to a seminar where you are taught on what you should eat and the food that you are advised to eat are expensive compared to the food that is prepared for the other people at home and you can’t buy your food and let others go without*. *You are forced to eat what is available because that’s what can be used by the majority at home*. *This endangers you to an extent that it becomes hard to control your blood pressure because you are told to eat traditional vegetables*, *but you end up taking what is available*. *[FGD (Korogocho)*, *UHTN participant (R4)*, *Female 61]*.

There were misconceptions and difficulties in implementing physical activity in this community. The most common exercises mentioned were house chores and walking. Some respondents stated that they were too old for exercise and they seemed to perceive that exercise was only vigorous activity such as running.

*I have not considered doing physical exercise unless I decide to walk for a short distance because at this age I can’t engage myself in running*. *[UHTN participant (R4)*, *Female 61]*.

*“Am very old*, *I cannot do exercise*, *I cannot do many things*. *I cannot even run” [IDI (Korogocho)*, *UHTN participant*, *200712_0542*, *Male 62]*.

Apart from discouraging physical activity, age was also mentioned as a barrier as it led to forgetfulness that affected the patients’ care in terms of medication adherence and keeping appointments for follow up. Health care providers also noted that it was not easy to care for old patients especially those who lived alone. They mentioned that the older patients were stubborn and hardly followed instructions. In addition, older patients were described as vulnerable because they relied on other people for their care including food preparation and healthcare

*“*…*the other one is the old age*. *Explaining to that old woman that she is required to take medicine daily because her pressure is high*, *there are those who will not even remember their return date when they are booked*. *So that is hectic and when you ask her to come with a guardian or a treatment buddy she will tell you that they are working and they don’t have time to come” [KII (Viwandani)*, *Health Care Provider*, *200701_0043]*.

The stresses of daily life and responsibility of taking care of the family were also reported as barriers in blood pressure control. Participants stated that hypertensive patients were prone to stress especially due to their low economic status that hindered their capability to buy medication and provide for their family.

*“So in our community maybe hypertension is at high because people from this community are struggling to make a living that leading to stress so most suffer from the same high blood pressure because of being unable to manage stress” [KII (Korogocho)*, *Health Provider*, *200531_1126272]*.

Knowledge on the causes and management of hypertension was limited among the majority of the respondents and this resulted in medication non-adherence. In addition, some of the interviewees were unaware of the asymptomatic nature of hypertension and the rationale for its lifelong treatment. The idea of having to take drugs continuously was also thought to be a burden and some respondents reported that they only took drugs when they felt unwell or when they experienced hypertension side effects. Medication side effects were a significant barrier to blood pressure control in the community. Some respondents reported having to stop taking their medications due to unfavourable side effects.

*“when young people have high blood pressure*, *they become depressed and ask if they will be taking the medication throughout their lifetime*. *They fear taking the medicine for a long period” [KII (Viwandani)*, *Health Care Provider*, *200703_0034]*.

*“The first thing is that my body is weak because there are some tasks that I cannot do*, *I can’t do any heavy task or if I do then I’ll be taking rest every now and then and the other thing is like my manhood is not active*…*not following instruction and not taking medicine though some medicines have side effects*, *side effects make some people to stop taking medicine like for example the one that I mentioned about my manhood*. *One decided to stop taking certain types of drugs when he sees such” IDI (Korogocho)*, *UHTN Participant*, *200712_2239*, *Male 58]*.

While patients possessed some general knowledge of their condition and hypertension, the level of knowledge was limited. Only a few patients were able to recall what their optimal BP was or could identify their target BP as informed by the health care provider and not all could remember their most recent blood pressure measurements.

Behavioural factors such as smoking and alcohol consumption were noted by health care providers to be very prevalent in the study community and this was a significant barrier to blood pressure control.

*“There are some patients especially men who have already been diagnosed and when they come here*, *maybe it is their first time they come here*, *some of them don’t know if they are hypertensive you see when you are taking history or maybe when they come*, *they have already taken alcohol*. *You know you can immediately know someone who is drunk and smelling alcohol*. *Then when you try to take history you find that he takes alcohol and maybe cigarettes and every time he comes to attend clinic you see that he is drunk*. *So you see someone is taking drugs when he is taking alcohol and those other cigarettes*, *it’s very hard to control blood pressure” [KII (Korogocho)*, *Health Care Provider*, *200709_0207]*.

Most respondents reported that they also had other comorbidities complicating their management. Comorbidities were mentioned as a barrier to practicing lifestyle changes to control hypertension especially diet modifications. In addition, the comorbidities meant that the number of drugs increased which was a burden to the patient. The most common comorbidity mentioned by respondents were diabetes and dyslipidemia.

*“For me am both diabetic and hypertensive so I think I have two challenges*, *one comes from the way am supposed to manage diabetes and the way am supposed to take antihypertensive [medications] to control my blood pressure but the biggest challenge that I have is to control diabetes that is causing high blood pressure” [IDI (Korogocho)*, *UHTN participant*, *200712_2239*, *Male 58]*.

In addition, denial and use of herbs was mentioned as a barrier by both healthcare providers and participants with uncontrolled hypertension.

*“I can say it is the denial because some of them they don’t accept at all that they have hypertension and also the use of herbal products because some believe in myths that if you take ABCD you should*, *for example if you take lemons or those ABCD they can heal your condition” [KII—Health care provider*, *200630–0306]*.

*Respondent*: *they decide to just either use herbal medicine or just staying with mediction and just hope that the blood pressure will lower on its self*. *[KII (Korogocho)*, *Health care provider*, *200627_0158]*.

### Proposed patient-level solutions

Most of the solutions recommended at this level were around securing resources to enable the patients to access hypertension care. Patients recommended income generating activities to enable them buy medications and cater for their other expenses. Patients also reported that if they could get free medication, then that would reduce their stress and in turn their blood pressure would be controlled. Others thought a change in their environment would help control their blood pressures. Some knew they needed to take their medications in order to control their blood pressure and so they felt it was important to work hard in order to pay for their medications.

*“for me I would really be happy if I can get someone to finance me to get those drugs and food*, *change my current way of life*, *the place where am staying so that I can stay at a place with some fresh air and clean environment”–[IDI (Korogocho)*, *UHTN participant*, *200711_001*, *Female 58]*.

Policymakers felt it was important for patients to get more information about their condition and how they can manage themselves well. Patients with hypertension felt that getting advice from the health care providers was important for their care. Health care providers suggested using community health volunteers (CHVs) with similar characteristics as the patients (e.g., an elderly CHV to pass the message to the patients in a language they understood).

*“Most important for me is education*. *If we have education for the clients to understand what they are going through and how it can be managed*. *We need to empower them with information*. *You know if we demystify the disease at their level*, *teach them how to control so that they take care of their generations”*. *[KII policy/decision maker*, *200629_2302]*.

### Perception and experiences of family and community-level facilitators and barriers

The availability of CHVs who work in the community was seen as a facilitator for blood pressure control in the community. The CHVs were credited for supporting the elderly in taking medication, giving hypertension information to the community members and referring patients to the hospitals for further care. In addition, the CHVs supported the health providers in reaching those who did not come to the blood pressure clinics

*“Sometimes I am helped by those people who bring medical services here [CHVs]*. *At this moment there is one called [name] who comes to help me…we are advised on how we can live with this condition*, *how we can be eating or drinking” [IDI (Korogocho)*, *UHTN participant*, *200710_002*, *Female 56]*.

*“As for now there are many programs that are addressing hypertension in the community*… *We’ve had some CHVs who give health talks to the community*… *Who are referring patients to us from the community*. *They were given some blood pressure machines to be going around taking the blood pressures then they refer those that have high blood pressure to the facility”*. *[KII—Health care provider*, *200627–0158*]

In addition, having social and physical support from family members to help the elderly clients was mentioned as an important facilitator. These family members were reported to provide health care assistance including accompanying the elderly to the health facilities for their regular appointments, supporting them in adhering to medication use as advised, and reminding them of the appointment dates.

*“*… .… .… ., *If at all an old lady comes and you ask her to bring along the her guardian and [the guardian/care provider] agrees to bring them and they agree that they stay together and she will be giving her the medicine as you have instructed*, *she will be attending her clinics*, *if they do the investigations like the ones that are supposed to be done after every six months then it becomes nice but if the patient comes alone and she is old*, *then there is nothing that you can do”*. *[KII—Health Provider*, *200701_0043]*.

Most participants reported having easy access to clinics where they went for screening and medication. They reported these clinics to be near their homes and therefore did not require a lot of resources to get to them. The community was reported to have both public and private health facilities. Some of the community members noted that they went outside their community for care when they needed specialised care and when referred.

Some patients reported their neighbourhoods to be supportive environments for blood pressure control. They noted getting support from neighbours with regards to food and money to buy medicines when they could not afford to buy for themselves. The community environment was also described by some respondents as an avenue to de-stress. Respondents noted that they would assist their friends and neighbours when they felt stressed for social support. The friends and community members also tried not to stress the patients.

*“There are women who assist me when I don’t have food to eat*. *They bring me food or even bananas and they tell me to cook because my son is not capable*. *He was stopped from working after cases of COVID-19 were identified in Kenya*.*” [IDI (Korogocho)*, *UHTN participant*, *200710_002*, *Female 56]*.

Despite the aforementioned facilitators, many barriers were mentioned at this level. Even though many patients reported accessing care in the community, other patients had to go far from their homes when they needed specialised care, which was a barrier as they needed to have transport. In addition, patients who wanted to avoid the stigma associated with having hypertension opted to seek care far from their homes.

*“Transport to come to the facility*. *Maybe [it] is their due date [appointment] to come to the facility*, *someone doesn’t have means to get to the facility”*. *[KII—Health Provider*, *200531–1126272]*.

An unsupportive family environment was also noted as a barrier in the community. Participants with hypertension reported not being able to eat different foods as required for their condition from those consumed in the house. Some respondents also noted that there was no one to accompany the older patients for their appointments and language was sometimes an issue. Others mentioned the noisy community environment also meant that it was not conducive for blood pressure control. Since family members are involved in influencing the lifestyle of the patients, including the food they eat and the money and time given to seeking health care and adherence to medication, lack of information among them led to lack of support. Patients also reported a stressful home environment as a barrier. Uncooperative spouses, alcoholic and drug abusing children were among the stresses mentioned.

*“Yeah*, *the other thing is lack of support*. *Some families don’t support their loved ones because you can see an elderly person who has kids but she can’t be brought for medication*. *From the community we can say lack of information is the same as ignorance” [KII—Health care provider*, *200701_0043]*.

Participants also reported stigma that they experienced as patients with hypertension. This led to the patients having to travel far to seek medical attention just to conceal their HIV status. Another barrier identified at the family/community level was the lack of knowledge within the community which has led to myths and misconceptions in the community about hypertension. For instance, some people in the community believed that being diagnosed with hypertension was a death sentence hence there was no need to seek medical care while others thought that once one has hypertension, it is not treatable or manageable. Myths and misconceptions in the community were also reported as barriers.

*“[at the] community and family level*, *you will understand that stigma is one of them…many [have] stigma and they are in denial…when one is in denial and [have] stigma*, *you will find out that the stigma from the community will cause it…Or some*, *some due to those underlying factors that we talked off*, *they have to travel very far so that the community does not understand them very well…for instance there is one that is HIV positive and also hypertensive…they won’t come to the facility around due to stigma”*. *[KII—Healthcare provider*, *200626–001]*.

Participants noted an increase in number of traditional healers in the community and it was thought that they may be affecting the patients with hypertension adversely.

*“I think traditional doctors are increasing in number and they don’t know what they are doing or they are doing some things that might affect other people*. *There are those people who go to seek care from those people and I think it is not good*. *The government should create awareness and awareness should be created on hypertensive and diabetic patient so that they can know where they can go to seek care*. *Some are told that if they eat somethings they will help them in managing their blood pressure and they end up losing their money” [IDI (Korogocho)*, *UHTN participant*, *200712_2239*, *Male 58]*.

Finally, the environment where the study was carried out was described as a barrier for a number of reasons. The limited space in the urban informal settlements discouraged exercise and walking due to congestion. In addition, the environment impacted on the foods the patients consumed.

*“In terms of physical and structural we also realize that there is no adequate space for physical activities*…*and where there’s space there is the issue of security…especially in the informal settlements…because you expect them maybe to do a morning jog which might not be very feasible due to safety reasons in those areas…and also the way the settlements are*, *there are spaces where people can do physical activities*. *[KII policy makers*, *200701–0035*, *0426*, *0425*]

### Proposed family and community level solutions to the barriers

Several solutions were suggested at the family and community level. The most mentioned solution was making use of the community health strategy to support continuous monitoring and screening as well adherence to medication and clinic appointments.

*Maybe we can use CHVs*. *When you get a client*, *first when you diagnose a patient with hypertension*, *you connect them to a CHV who will be calling them to know where they stay even locations and even if it’s possible they pick up their medicine for them which they are taking and to be reminding them of their clinics*. *For those who can’t make it to the clinic*, *they can take the medicine to where they can access them” [KII health provider*, *200531_1126272*]

Another solution was the creation of general awareness in the community about their condition. This involved where patients needed to seek treatment.

*The government should create awareness [in the community] and…*. *hypertensive and diabetic patients [should be educated] so that they can know where they can go to seek care*.*” [IDI (Korogocho)*, *UHTN participant*, *200712_2239*, *Male*, *58]*.

The health care providers who mentioned language barrier as an issue suggested that the patients could come with their care providers for appointments. Health care providers felt that this would help during the clinic appointment and it was hoped that the care provider would support the patient with adherence to the medication due to being present when the information is given to the patients

*“One is that they come*, *for those that have language barrier should come with their care providers so that we are able to relay the information through their care providers” [KII health care provider*, *200627_0158]*.

### Perception and experiences of health system level facilitators and barriers

Health professionals were mentioned to be the key source of information for hypertensive patients. Patients reported having good communication with their doctors and the service providers were generally described to be cooperative. The capacity of the health providers was also reported as a facilitator in that the healthcare providers were able to treat patients who had comorbidities and were able to change the prescription when needed. Some of the facilitators mentioned at the provider level included provider training received and the providers’ ability to follow the guidelines for hypertension care in the facility.

According to the study participants, their doctors changed their medication according to their blood pressure measurements. Most patients with uncontrolled hypertension mentioned that their medications had changed over time. Some reported taking higher doses while others noted that the number of drugs for hypertension also increased.

*Moderator*: *Have you been taking the same number for the last one year*?

*Respondent*: *It depends with the measurement*. *They change drugs if the blood pressure is very high–[IDI (Korogocho)*, *UHTN participant*, *200711_001*, *Female 58]*.

Health care providers offering continuous care and following up with their clients was also noted to be a facilitator. Some health care providers were also working with CHVs to support in follow-up of patients

*“By use of CHVs*, *we give them cards and then TCAs [(to come again (date)/next appointment date)] and then we do follow up by calling them to remind them that they are supposed to come to the clinic as soon as their medicine are done” [KII Health Care Provider*, *200531_1126272]*.

The patients reported that they were happy with the care they were receiving and there seemed to be good rapport between the patients and the health care providers. Some even got financial support from the health care providers who were also described as being friendly. One respondent noted that their blood pressure was stabilized when it was really high while others mentioned that they were also advised on how to manage their blood pressure.

*“I told you that they check my blood pressure measurements and if they find that my blood pressure is very high then they ask me to sit down somewhere then they break a tablet and give me to swallow*, *then I my pressure is measured again after some time then they talk to me on how am supposed to eat to manage my blood pressure*, *then they give me water”*. *[IDI (Korogocho*, *UHTN participant*, *200713_0305*, *Female 62]*.

*Also that you are served better and the doctors at [facility] advise you well on how your blood pressure should be*. *[IDI (Viwandani)*, *UHTN participant*, *200710_0535*, *Male 57]*.

Further, some respondents described that the wait times were appropriate and they were able to return to their daily activities after their appointments.

*Yeah*, *they serve us well*. *Hypertensive patients are treated fast when we go there*. *[IDI (Korogocho)*, *UHTN participant*, *200710–001*, *Female 44]*.

Respondents further noted many facilitators in regards to health systems. Healthcare providers noted that there were systems in place for following up patients particularly those whose blood pressure have not been controlled. Other respondents also mentioned that systems were in place in their facilities to quantify their needs for medicines for hypertension care even though the shipment for medicines mostly never arrived on time due to various reasons.

*“We are able to quantify the needs; we are able to order on time but we are not able to receive supplies on time”*. *[KII–Policy/decision maker*, *200629_2302]*.

Some of the respondents noted they were able to find medications in the facilities they visited and that the medications were offered at no cost. They also mentioned they rarely found stock outs of the medications they needed and in the instances that the facility they used was out of medication, they were given a date to come back for their medication.

*“At [facility] drugs are available and if they are no drugs they just tell you to come on a certain day and you will find them*. *They don’t tell you to go and buy drugs like for my condition*.*”*. *[IDI (Korogocho*, *UHTN participant*, *200711_004*, *Male 56]*.

*“It’s hard; maybe you can miss drugs once [at the health facility] but not many times”*. *[IDI (Korogocho)*, *UHTN participant*, *200711_001*, *Female 58]*.

Very few participants indicated that the operating hours of the facilities they visit for hypertension care was fine. A few of the respondents mentioned that the facilities they visited provided 24 hour services while others mentioned specific hours in which hypertensive patients were seen.

Despite the provider-level facilitators reported, many barriers were reported about the healthcare providers in the community. Inability to regularly follow-up and closely monitor patients was described as a significant barrier. Healthcare providers in the community felt their message would have more impact if they were able to follow-up with their patients more frequently. The main barrier to regular follow-up and monitoring was lack of appointments in the facilities they worked in. Other barriers mentioned about the care the providers gave were lack of time due to the providers’ heavy workload, lack of training and knowledge or expertise to treat hypertensive patients that had comorbidities.

*“Because of the long queues*, *you can wait for long and even decide not to attend the next clinic” [IDI (Korogocho)*, *UHTN participant*, *200710–0606*, *Female 62]*.

The high workload of doctors was noted as a challenge as there was no time for a detailed discussion between the patient and the service providers. From the interviews, it was clear that the respondents were desperate for information on hypertension as they were not getting answers from their health care providers.

*“I asked but these doctors from public hospitals don’t answer some questions*. *The just take their pens to write when you try to ask the questions*. *They just tell us to continue taking drugs and if you tell them that you are tired of taking drugs then they add you other drugs” [FGD (Korogocho)*, *UHTN participant (R3)*, *Male 61]*.

The expertise of the healthcare providers was also mentioned as a barrier. Some patients did not trust the care they received so they either stopped seeking care and only went to buy medication from chemists or changed hospitals.

*“[It] reached a time that I lost trust with the doctors that were treating me at [name of hospital] because they were just checking my blood pressure and gave me prescriptions in case they found that it was high but at [national referral hospital] I would say they are more active*, *they check what could be wrong with the patient*, *my liver was checked at [name of referral hospital] but at [name of community hospital] I was just going to collect drugs and go back home” [IDI (Viwandani)*, *UHTN participant*, *200722_0058*, *Male 52]*.

Another barrier mentioned by patients with hypertension was that providers were noted to offer the patients only what was in stock, which was particularly challenging for those patients on multi-drug regimens. Health facilities were also described to have a poor supply management system as medication stock outs were mentioned repeatedly as a major barrier to blood pressure control. Patients and health care providers alike mentioned this to be a problem. Patients reported that even if the medications were free in public facilities, these facilities often lacked the medications and patients had to purchase the medication elsewhere. This was particularly challenging for those patients who were on multi-drug regimens for their hypertension treatment. In addition, due to the inefficient supply system, it was noted that when some medicines arrived, some were expired and patients were using them before they realized they were expired.

*Respondent*: *“I can say that they get good care but the only problem that they have is drugs*. *When we go to the hospital*, *like for me I only get one type of drug and I don’t get the other one so there is that problem of getting drugs…We have stayed for long without getting drugs*. *We have been buying all the drugs from last year August*. *We had a one problem*, *this drug called…6*:*26–6*:*30(Not clear) came in late and they came when they were already expired*. *We only used them for one month and realized that they were expired we were not getting any drugs at that time*. *[IDI (Viwandani)*, *UHTN participant*, *200621_002*, *Female 63]*.

A surprising finding in regards to out of stock medicines at the public facilities is that patients reported that they were always asked to purchase the medications from elsewhere and that the medications were always available in private pharmacies yet they were unavailable at the public facilities in which they sought care where they expected to receive the medications for free. It also emerged that facilities purposely did not stock all types of medications for hypertension—they only stocked the cheaper medications.

*R1*: *They should also consider the drugs because if you go to the hospital and you are told that there are no drugs and so you will be given one type and asked to go and buy the other one*. *That one also contributes*. *They should make sure that hypertensive and diabetic drugs are available at the hospital*. *It really surprises me that you don’t get some drugs at the hospital but when you go to the chemist you find them there yet a chemist is privately owned*. *Who is more powerful between the government and the private chemist owners*? *The government should be having everything and personally I think there is a challenge there–[FGD (Korogocho)*, *UHTN participant (R1)*, *Male 61]*.

*“Currently what I can say about that our facility does not stock all the hypertensive drugs*. *They only stock the ones that are cheaper to the patients which sometimes is not effective to the patient”—[KII—Health care provider*, *200627_0158]*.

Medication stock out was a common feature in most public facilities regardless of where the facility was located. In addition to not stocking medications, a near absence of public facilities was mentioned in the community. In each of the two study communities, respondents mentioned that there was only one public level facility in each of the study sites and that they almost always didn’t not have medications. Healthcare providers at the public facilities also advised the patients to buy medications that were out of stock in other private facilities.

*Respondent*: *In Korogocho it’s like we don’t have a public facility*, *its only one and it’s like it is never stocked with drugs*. *It’s at the chief’s camp but the problem is that there are no drugs there and when you go there you are told to go buy—[IDI (Korogocho)*, *UHTN participant*, *200710–0606*, *Female 62]*.

*Respondent*: *I told you that I go to a hospital managed by the city council called [public facility name]*. *This is a public dispensary and I am not charged when I go there but we are asked to buy if there other drugs that are not available*. *[IDI (Viwandani)*, *UHTN participant*, *200710_0535*, *Male 57]*.

Facility hours of operation was cited by many as a barrier to blood pressure control. The government owned facilities did not operate beyond the normal working hours (8am to 5pm) or on the weekend thus limiting care to those who have to work and need care outside those working hours. The clinic days for hypertension care was also reported to be once a week and the hours to be seen were also short.

*“So this complications may come at night when access is limited*. *So access on time can only be from 8–5 and Monday to Friday*, *outside those hours you may not get access to care”—[KII–Policy/decision maker*, *200701_2332]*.

Another major barrier mentioned was that the facilities were short staffed; this shortage of staff was the norm across all the facilities thus leading to high workload to the current staff and this also led to long wait times for patients to be seen. Healthcare providers reported that if they were not available on the clinic day, then the patients would have to come back on another day and so the patients would have to do without their medications if they were completely out. Patients with hypertension also noted that they had to wait months to be seen. Others reported that sometimes they would spend their whole day to be seen, while some patients mentioned not booking other activities on the same day as their clinic days. They also noted that they had to arrive very early if they were to be seen early. Patients noted that they were attended to faster in private facilities. Policymakers for the area also noted that there was a shortage of staff in the public community facilities thus the staff are overwhelmed.

*“Because of the long queues*, *you can wait for long periods and even decide not to attend the next clinic”—[IDI (Korogocho)*, *UHTN participant*, *200710–0606*, *Female*, *62]*.

*For me I think staffing [is a barrier]*, *because if I am not around then they [patients] will not go home with drugs*, *the patient will have to leave their other duties to come here again and they will get tired*.*”—[KII-health care provider*, *200701_0043]*.

The patients also mentioned the attitude of the healthcare providers as a barrier. Some patients felt that they couldn’t consult the health providers and some mentioned that the healthcare providers shouted at them.

*“They are supposed to follow up and guide us on how we are supposed to live but we cannot be the ones telling the doctor what to do because he will think that we are commanding him and that’s why we just keep quiet and change the facility*… *I would like to tell you to talk to those people [healthcare providers] if at all you work with them because hypertensive patients become worse when they are shouted at*.*” [IDI (Viwandani)*, *UHTN participant*, *200715_006*, *Female 52]*.

Quality of care at the facilities was reported to be compromised because of the high workload thus the doctors were not able to give proper attention to patient care.

*“In our experience*, *the feedback we get from counties is that there is generally a critical health workers shortage*. *And that is in their report*, *but from our own observation we have found out that in the health facilities that provide hypertension care the clinics tend to be very understaffed*. *And the [facility] tends to lean towards overwhelming the staff that is there*. *…*. *Like there is a clinic once in every two weeks so the provider gets a mass of patients to come in on a certain day*. *And that impacts on the quality of care provided and also impacts on the perception that there is health worker shortage”—[KII–policy/decision maker*, *200703_0220]*.

Lack of equipment, reagents for tests and lab tests in general were cited as barriers to hypertension care in the community because it meant patients needed to go and get the tests in places that required them to pay for the tests. Study participants also reported that sometimes the equipment broke down or they needed maintenance. Delays in replacing and repairing equipment were also cited.

*“We don’t have the capacity and the ability to do routine care and follow up investigations like kidney tests and the patient is not able to afford’’*.*—[KII–health care provider*, *200627_2209]*.

*“*..*some of the monitoring equipment sometimes they break down and may need some maintenance and things”—[KII–policy/decision maker*, *200701_2332]*.

### Proposed solutions at the health-system level

Various solutions at the health providers’ level were proposed by the patients, the health care providers themselves as well as the policy/decision makers. The respondents noted that it was important to ensure the patients and the community in general be knowledgeable on hypertension. However, the health providers noted that the heavy workload was a hindrance to achieving this. A healthcare provider mentioned increasing the number of healthcare providers’ as a solution to the heavy workload and increasing providers’ capacity through trainings and seminars.

*“those that provide care should be advising us on what to do*. *Advice is good” [IDI (Korogocho)*, *UHTN participant*, *200712_0542*, *Male 62]*.

*“There should be more training for the health care workers on the current guidelines and management of hypertension and diabetes*. *Now if the management can add another health care worker who is equipped with knowledge to handle hypertensive clients then this I think will be good” [KII health care provider*, *200627_0158]*.

To counter the heavy workload, setting up special clinics for hypertensive patients was proposed as a possible solution. In addition, the need to set up a follow-up mechanism for patients was proposed. The option to provide continuous support was also mentioned as a possible solution.

*“So for workload I think we schedule for their special clinic*. *Like we just schedule like 3 days of the week where a client knows*, *even come one knows that from this hour to this hour we are attending to hypertensive clinic*…*maybe that could help” [KII health care provider*, *200531_1126272]*.

The main solutions mentioned at the health system mainly revolved around availability of medications, tests and equipment for hypertension. Solutions particularly for the public facilities were to ensure that they have continuous medications in stock. A consistent supply of medications for hypertension was suggested by patients and healthcare providers who acknowledged the medication stock out was a major challenge affecting blood pressure control. They also suggested that people with hypertension be treated that same way people with HIV are treated because people with HIV are never without medication.

*“*..*that there is availability of drugs*, *make investigations available and affordable and if possible make them free”*. *[KII healthcare provider*, *200627_2209]*.

*“I said that it would be better if they could help us get drugs easily*. *They should treat us the same way they treat those patients with other diseases like HIV because it is very hard for HIV people to miss drugs”*. *[IDI (Korogocho)*, *UHTN participant*, *200720_001*, *Male 60]*.

Majority of the respondents felt that medications for hypertension should be provided for free. Almost all respondents felt the cost of hypertension medications was prohibitive and the government needed to intervene and provide these medications for free especially to the elderly.

*“Maybe if the government can make sure that the elderly get those drugs or if they can be sent even some little money for medication”*. *[KII healthcare provider*, *200703_0034]*.

Participants also suggested that taxes needed to be removed from hypertensive medications to make the medications cheaper for patients to buy. They also suggested that a donor can be approached to help with medications while another suggestion was to engage a non-governmental organization to help with the provision of subsidized medications for hypertension.

*“The government should know what to do like maybe not taxing such drugs so that the prices can be cheaper”*. *[IDI (Korogocho)*, *UHTN participant*, *200712_2239*, *Male 58]*.

*“I think on our management part*, *maybe to liaise with some NGO who can be supporting the drugs for the patients…*.*” [KII healthcare provider*, *200627_0158]*.

Since healthcare provider staffing was a major barrier, most of the respondents suggested that the number of doctors attending to patients with hypertension needed to be increased so that the long wait times are reduced for patients.

*“They need to add the number of doctors and by doing that then it will take a shorter period and we will get time for us to hustle for ourselves*.*” [IDI (Korogocho)*, *UHTN participant*, *200721_003*, *Male 50]*.

*“Number one*, *increase the number of staff*, *number two*, *maybe access to facility and infrastructure*.*”—[KII—policy/decision maker*, *200701_2332]*.

Some respondents felt that providers needed to provide hypertension care counselling on issues relating to what they could do to improve their blood pressure control and information on what would be the ideal nutrition for them.

Participants noted the need for better linkage between facilities to improve referrals. They also recommended special clinics in the community to reduce referrals to outside facilities which some patients find difficult to access. Strengthening linkages between the community and the facility was also suggested as was the integration of programs to avoid missing other conditions that the patients have that need attention.

### Policy-level facilitators and barriers

Few facilitators at the policy level were mentioned. Healthcare providers mentioned that policies/guidelines were available for the management of hypertensive patients.

*“We have policies on how to manage hypertension”*. *[KII—Healthcare provider*, *200531_1126272]*.

*“The guidelines are there*. *Like when do we diagnose hypertension*, *when do we start medication and how do we scale up or scale down*. *So the guidelines especially the clinical ones are in place”*. *[KII—Policy/decision maker*, *200702_0216]*.

Barriers mentioned at the policy level included the lack of guidelines, not having up to date guidelines on hypertension, guidelines not being cascaded to lower level facilities and not having a budget line or specific allocation for hypertension care within the healthcare budget.

*“Not really*, *mostly we have the MOHs like in the bigger hospitals where we can consult*, *we consult from them but we don’t have national guidelines that we are using currently*.*” [KII—Healthcare provider*, *200701–0035]*.

*“eehhh*,*[guidelines] not the latest version*. *It is an old one*. *A very old one”*. *[KII—healthcare provider*, *200625_002]*.

It further emerged that to have access to certain hypertension medications and products, health facilities needed to be designated at a higher level and this may have contributed to the medication stock outs experienced in the lower level facilities.

*“So there is…Eeehh I’ll call it unapparent lack of harmony in the policies of the ministry*. *And the general programming within the ministry has a detrimental effect on care for hypertension*. *Where you find that up until late last year*, *the access to health products for hypertension was limited to certain levels of health facilities—basically from level four and above*. *Secondary care and above*. *Where at the same time the guidelines for care for hypertension were promoted or cascading to the lowest levels like level 2 and 3*. *So there is discordance between what the guidelines say and what these facilities can access in regards to products to provide for hypertension care”*. *[KII policy/decision maker*, *200703_0220]*.

Allocation of resources was cited also as a barrier to blood pressure management. It was noted that there is no specific allocation or budget line for hypertension care, which falls under non-communicable diseases. It was further noted that much of the emphasis in regards to resource allocation was more geared towards communicable diseases yet there was a growing burden of non-communicable diseases.

*“So you find that at the national level there are no dedicated budgets for programming for hypertension despite being condition that has high prevalence in the country”*, *[KII policy/decision maker*, *200703_0220]*.

*“We don’t have a specific allocation for example for NCDs that is non-communicable diseases of which hypertension falls under*. *I think we all*, *how do I put it*. *All health care budgets are combined*. *Again you see we are battling a lot of communicable diseases*. *So the non-communicable ones are eeehh*. *We don’t give them the emphasis that they deserve because we are overwhelmed already*. *Our emphasis is more on communicable ones like you have seen of late*, *the non-communicable diseases like hypertension and diabetes are going up”*. *[KII policy/decision maker*, *200702_0216]*.

Healthcare providers felt there was an absence of healthcare providers in the policymaking arena. They felt that health care representatives needed be present in decision making.

*“The only thing I can say about that is that when they are making their decisions*, *most of us are usually not involved*, *not like we need to be involved but come of our leaders like let’s say the clinic officers council*, *the nurses council they need to be involved so that the information reaches us if there are changes in the management”*. *[KII healthcare provider*, *200627_0158]*.

It was suggested that data on hypertension needed to be strengthened. More data was needed to know the number of people dying from hypertension as it is known for Malaria. Data was mentioned to be important because it informs policies needed. Increased research on hypertension was suggested to inform interventions and policies.

*“And then also provide data like a database*. *I think also our database is not very good…*..*It is easy for me to say there is this number of people dying from malaria each year but it’s not easy for me to to get the number of people dying from hypertension for example every year”*. *[KII policy/decision maker*, *200702_0216]*.

### Proposed policy-level solutions

At the policy level, several strategies were suggested by the study participants that would help with blood pressure control. Respondents emphasized repeatedly that the government needed to provide hypertension medications and tests at no cost. Respondents felt that patients with hypertension needed to be accorded the same benefits as patients with HIV and TB and be given medications and testing at no cost.

*“It would be better if the government ordered that high blood pressure medication be given for free but the government is not thinking about us*. *They are not giving us drugs for free*. *The government should think about cancer patients*, *those that are hypertensive and also the Diabetic ones”*. *[IDI (Korogocho)*, *UHTN participant*, *200713_0305*, *Female 62]*.

*“The medicines for TB are free*, *the medicines for HIV are free*, *and why don’t they [government] make the medicine for hypertension free as well*?*” [KII healthcare provider*, *200626_001]*.

Study respondents suggested that medications for hypertension should not be taxed in order to make them more affordable for patients, leading to better adherence. Additionally strategies suggested that donors who can support the provision of hypertension drugs should be approached. Some hypertensive patients felt they needed monetary support from the government so that they could buy their medications and foods in line with hypertension care. Another strategy suggested to help with reducing the medication burden was the implementation of universal health coverage.

*“The government should know what to do like maybe not taxing such drugs so that the prices can be cheaper”*. *[IDI (Korogocho)*, *UHTN participant*, *200712_2239*, *Male 58]*.

*“If the government would implement universal health whereby people who don’t have money are catered for”*. *[KII—Healthcare provider*, *200625_002]*.

In regards to guidelines, there were suggestions that it should be available at all facilities and the guidelines needed to be the most updated versions. It was further suggested that there should be policies for non-communicable diseases as they are for communicable diseases.

*“They should make sure that every facility has a guideline”*. *[KII—Healthcare provider*, *200701_0043]*.

*“They should come up with policies that don’t just focus on communicable diseases but also non-communicable diseases*.*” [KII—policy/decision maker*, *200702_0216]*.

Healthcare providers suggested that there be more awareness and advocacy activities around NCDs in general hopefully to garner the same attention that HIV has. Policy and decision makers noted more effort needs to be put on non-communicable diseases as has been put on communicable diseases.

*“To create more awareness*, *give health education on management of care of patients with NCDs…Updates yeah*. *Especially from my experience the education on NCDs is never rampant like the HIV*, *you know HIV everybody knows about it but when it comes to NCDs it seems that nobody cares as in it’s not highlighted as much*. *(In Swahili) There’s not as much education on it like those other things that are highlighted …now that corona has come it is being highlighted*, *or malaria but these ones for NCD there is no one who is concerned with them”*. *[KII—healthcare provider*, *200703_0034]*.

## Discussion

This study used the SEM framework [31] to assess the facilitators, barriers and solutions to blood pressure control among participants with uncontrolled hypertension and comorbidities in two Nairobi slums. This study provides key insights collated from patients’, healthcare providers’ and policy/decision makers’ perspective on the facilitators and barriers encountered by people with uncontrolled hypertension in the slums and solutions to identified barriers. The application of the SEM framework to analyse the data collected demonstrates that there is a need to intervene at multiple levels of the SEM framework.

Our findings reveal that access to medication is a major barrier to blood pressure control among patients with uncontrolled hypertension and comorbidities in Korogocho and Viwandani. High prices and the poor socio-economic capabilities of the urban slum residents in this study have limited access to treatment thus affecting compliance to hypertension medications. High cost of medicines has been confirmed in slums across Africa and other low-and-middle income countries [32,33]. While access to medicines is a barrier to blood pressure control in some low-middle-income countries, it seems other countries have started embracing universal health coverage thus providing healthcare and medications for free. For instance a study conducted in Eritrea looking at barriers and facilitators of hypertension management reported that patients appreciated their governments support in providing free medication for hypertension thus improving adherence to medication [10]. A study in Malaysia also found that patients with hypertension had no problem with accessing medications because they were provided free of charge at public facilities [34].

At the individual level, adherence to medication regimens by patients is also affected by the regular unavailability of drugs in the facilities that are expected to provide them for free. Adherence was further worsened by the cost of the medicines thus patients were buying inadequate doses and missing or skipping doses due to inability to buy the medicines. This was evident in a previous study conducted among low income earners in five regions in Kenya [35]. The study found that 38% of households forwent healthcare needs due to lack of money and at times bought less than their required treatment regimen. While compliance to medications is important in hypertension management, in this study compliance is affected due to inaccessibility of medications in the community. Unavailability of medication and the cost of medications were barriers mentioned by the majority of study participants and this adversely affected blood pressure control and adherence to treatment. One of the sustainable development goals’ (SDG) target, “*access to quality essential health-care services and access to safe*, *effective*, *quality and affordable essential medicines and vaccines for all*” [36] is not being met in these two communities.

Other barriers at the patient level were poverty, lack of formal employment, lack of information and knowledge, misconceptions, not having medical cover, being older in age and depending on others for hypertension care. These barriers are similar to those reported in similar settings across sub-Saharan Africa [10,37]. Knowledge about hypertension plays an important role in blood pressure control. In the current study patients’ knowledge was limited and the study participants with uncontrolled hypertension did not seem to understand the rationale for the lifelong treatment of hypertension. Previous research has shown lack of knowledge coupled with misperceptions about the disease can affect adherence to treatment [38,39]. A study by Meinema et al. [39] conducted among African Surinamese and Ghanaians with uncontrolled hypertension in the Netherlands showed that using a culturally adapted hypertension education program gave the patients a better understanding of hypertension and improved their understanding about the chronic nature of hypertension thus improving medication adherence.

In the current study, all the patients were older adults with other comorbidities in addition to having uncontrolled hypertension. Comorbidities can have an effect on blood pressure control, and this may explain the inadequacy of blood pressure control in this population. Studies in high income countries have shown that people with comorbidities have higher risks for uncontrolled hypertension [40,41]. A review of the literature estimated that more than 50% of the older adults have multimorbidity and the prevalence of multimorbidity increases with age [42]. A recent study conducted in the current patient population also found that close to a third (28.7%) of the study participants had multimorbidity (defined as two or more chronic conditions) and the commonest identified chronic conditions were hypertension and obesity in this population [43]. There is also literature from similar settings showing that hypertension co-exists with other comorbidities [16,44–46]. Excess weight is a known risk factor for high blood pressure. An earlier study also in the same study population suggested lifestyle changes in this community has led to a rise in overweight and or obesity [47]. The prevalence of overweight/obesity coupled with hypertension may explain the inadequacy of the blood pressure control. A recent study in China has found a positive association between BMI and blood pressure [48]. Therefore, in addition to clinical management of patient comorbidities, this population will require lifestyle changes to address modifiable risk factors to enable them to get their blood pressures under control.

Staffing for healthcare providers was noted to be low in this study. Previous research has shown that increased health care personnel staffing has a positive effect on health outcomes [49,50]. However, most low- and middle-income countries including Kenya lack the needed number of healthcare personnel to provide essential services. Likely reasons for this shortfall include migration of health personnel in search of greener pastures and the reduced capacity of countries or institutional bodies graduating people with the needed qualification for healthcare. It has been estimated that countries with fewer than 23 physicians, nurses and midwives per 10,000 population generally fail to achieve adequate coverage rates for selected primary health-care interventions [51]. The pervasive lack of skilled care is likely the reason for reduced communication among physicians with patients as has been reported in the current study. Good communication between patients and their physicians has many benefits. Benefits include improved compliance to prescribed treatments [52]. A recent article by Zulman and colleagues [53] looking at practices to foster patient physician connection in clinical encounters pointed out how impersonal patient and physician encounters have become.

A health service provision assessment conducted in the above two slums about a decade ago revealed that the majority of public health facilities did not have the required staff, equipment, drugs or the mandate to handle chronic diseases [13]. These barriers continue to persist in 2020 and as a result, the majority of healthcare visits continue to occur in private facilities which are also typical in the slum areas. Drug and equipment stock out in public facilities force the poor urban populations to visit private pharmacies for care thus increasing their out of pocket expenditure [35]. A study conducted in a rural part of Western Kenya also found that lack of drugs at the facilities patients visited was among the health system barriers to blood pressure control [37]. An earlier study by Buigut et al [54] in the study area showed that seeking care in a public health facility was associated with increased odds of experiencing catastrophic health expenditure and for this reason many informal slum residents would forgo health service utilization. A more recent collaborative study looking to improve slum health in the study area also found that many households in the study area spent a significant proportion of their money on healthcare [55].

Out of pocket payments for health services and lack of health insurance coverage was identified in this study as a barrier to accessing health care coverage. A study conducted in the above two slums in 2012 revealed nearly 90% of the slum residents did not have access to any type of health insurance [56]. A more recent study in 2018 in the Viwandani slum revealed only 43% of the sampled population had health insurance [57]. While this is an improvement from the study conducted in 2012 by Kimani and colleagues, the coverage is still very low. A study by The Improving Health in Slums Collaborative [58] examining inequalities of healthcare need, access, use and expenditure within slums including the current study areas revealed that there is a very high degree of inequality of household budgets in slums and that this translates into inequities in the access to and use of healthcare services in slums.

Decision making requires reliable information. Hypertension and NCDs in general have not received the same type of support in funding as communicable diseases as evidenced by global initiatives such as the Global Fund to Fight AIDS, Tuberculosis and Malaria (GFATM) and the United States President’s Emergency Plan for AIDS Relief (PEPFAR) which have significant resources and good reporting systems. In this study, participants reported lack of and limited mortality data on hypertension. This type of information is readily available for other conditions such as Malaria, Tuberculosis and HIV due to the funding attached to these programs. Funding of research and good information systems are therefore important in hypertension care in order to provide early warning, basis for planning, analysis of health data among other functions [36].

Hypertension has been identified to be a major contributor [59] to the observed rising deaths due to NCDs in Kenya. These deaths have risen from 35% to 45% in a span of seven years (2003 to 2010) [60]. Results from the current study showed that there were no clear guidelines for hypertension care in some of the facilities which is likely to contribute to some of the above deaths. It also emerged that even though the guidelines are disseminated to all levels of the health facilities, access to medication is limited to facilities in the higher levels thus also contributing to the gaps in access in medication observed in the two slum communities.

This study showed that majority of patients lacked health insurance and this has been previously described in the 2013 Kenya budget and utilisation survey. The survey reported that approximately 83% of the Kenyan population lack financial protection from health care costs and about 1.5 million Kenyans are pushed into poverty each year as a result of paying for health care [61]. The 2015/16 Kenya Integrated Household Budget Survey revealed 19% of the Kenyan population had some form of health insurance which is a slight improvement from the 2013 estimate [62]. Even though the provision of public health care in Kenya is subsidized, it is inadequate due to the dense population in urban areas. Furthermore, the public health care system suffers from inadequate infrastructure and workforce, long queues, and shortage of drugs [63]. In 2018, the Government of Kenya committed to achieving Universal Health Coverage (UHC) by the year 2022. This is a bold initiative and a major step in the right direction for many Kenyans who lack financial protection. The National Hospital Insurance Fund (NHIF) is the main insurance scheme in Kenya and it is expected to improve the provision of healthcare services in Kenya. The scheme covers both the formal and the informal sector. Coverage is high in the formal sector due to the mandatory nature of contributions from employers while the coverage from the informal sector is very low due to the voluntary nature of contributions. The majority of the study respondents in this study have strongly articulated the need to have free healthcare and the full implementation of the UHC in Kenya can make this a reality. Otherwise, the provision of care will be inequitable and more biased towards those who can afford the premium contributions.

### Strengths and limitations

To our knowledge, this is one of the first studies to ask healthcare providers, policymakers and patients with uncontrolled hypertension and comorbidities in an urban slum setting to explore their experiences and views about facilitators, barriers and solutions to hypertension management at the different levels of the SEM framework. This study captured an integrated and diverse range of perspectives on facilitators and barriers to blood pressure control in slum communities by purposively engaging with patients with uncontrolled hypertension, health care providers and policy/decision makers. Examining uncontrolled hypertension through the socio-ecological model has increased our understanding of how to tackle blood pressure control while highlighting potential strategies at the different levels of the SEM. However, some limitations should be noted. The results from this study are from two Nairobi slum communities and even though it may be applicable to other similar slum settings; it may not be generalizable beyond slum communities. While a strength of this study is that views from different study participants were sought for each of the SEM levels including the families/community members who did not participate in the study, it is thus possible that this particular groups’ perspectives may not have been accurately captured by the current study respondents. Another limitation to consider is the timing of data collection which corresponded with the current pandemic spread of SARS-CoV-2 which posed disruptions in the delivery of health care services. Thus it may be that some of the patients’ challenges discussed could have been a result of the measures put in place to curb further spread of the virus. Similarly, the switch to phone interviews for most of the interviews rather than the traditional face-to-face interviews due to the pandemic may have reduced or limited the level of detail needed to capture the non-verbal cues that are important in guiding further discussions. Nonetheless, findings from this study can help inform efforts to develop multi-level interventions to improve hypertension control among similar urban slum residents.

### Recommendations

At the patient level, barriers affecting patients’ access to hypertension medication need to be removed and or alleviated through the provision of free medications or subsidized medicines. Also, more frequent educational sessions should be conducted with patients so that they are well informed about their conditions and what they need to do to control their blood pressure. At the community level, hypertension care awareness is critical in ensuring a good understanding among the community and family members on hypertension care. Approaches at this level should also consider more involvement of community health workers/volunteers. At the health system level, approaches should focus on improvements at various levels within the health system structures such as; human resources, health management, health systems and governance. Lastly, at the policy level there is need for policies and directives that ensure equitable care is received by all including those in the slum communities or those seeking care at lower level health facilities. To address this, policy makers could consider expanding the healthcare mandate at lower level facilities by extending treatment for hypertension care in lower level facilities.

## Conclusion

This study presents the findings from a qualitative study of multiple levels of factors associated with uncontrolled hypertension. The findings demonstrate that uncontrolled hypertension is a major public health issue in slums of Nairobi and it is associated with barriers at different levels of the socio-ecological framework. The findings from the present study can be used to design interventions to address the interplay of factors operating at multiple levels of the SEM, from the patient level all the way to the policy level. Importantly there is a need for policies that facilitate equitable care in slums through increased access to subsidized or free medication.

## Supporting information

S1 File Community transcripts. (ZIP)

S2 File Health care provider transcripts. (ZIP)

S3 File Policy makers transcripts. (ZIP)
