# Supplementary material for: A socio-ecological framework examination of drivers of blood pressure control among patients with comorbidities and on treatment in two Nairobi slums; a qualitative study
Source: PLOS Glob Public Health. 2023 Mar 10;3(3):e0001625. doi: 10.1371/journal.pgph.0001625 (PMC10021823; doi:10.1371/journal.pgph.0001625)
Supplement: S1 File — (ZIP) [file pgph.0001625.s001.zip › Community/200621_002 (Pilot).docx]

**Moderator: {Name}**

**Code: 200621_002 (Pilot)**

**Moderator:** In this area there is a high burden of uncontrolled high blood pressure. We would like to understand how this community perceives current hypertension care services. What are your views about the care provided for high blood pressure in this community?

**Respondent: Pardon please**

**Moderator:** What are your views about the care provided for high blood pressure in this community?

**Respondent: I can say that they get good care but the only problem that they have is drugs. When we go to the hospital, like for me I only get one type of drug and I don’t get the other one so there is that problem of getting drugs. The other problem that we have is that we lack a big hospital so when your blood pressure is very high then you have to go for a checkup in another facility like Mbagathi or Mama Lucy and sometimes transport is a problem and we suffer when we are in a bad condition. The other problem is that most people don’t know if they have that condition and so you can just see a person suffering yet he doesn’t know and for those who know that they are sick they just ignore taking drugs. That the problem that we have in this community**

**Moderator:** Is there anything else that you can add?

**Respondent: In addition I can say the poverty situation is also contributing to this problem because maybe someone is stressed because of how life is at this time when there is this pandemic and getting money is hard. Like now the government is giving out food but you have never received any. I think that is also contributing a lot**

**Moderator:** Is there any god thing that you can add?

**Respondent: What I can add is that I think that most of the people who are suffering due to this condition are these young mothers who are pregnant**

**Moderator:** You had mentioned that you receive good care, can you elaborate?

**Respondent: If in can elaborate?**

**Moderator:** Yes

**Respondent: About the pregnant mothers or about ours**

**Moderator:** Yours that you had said before that they attend to you very well. What did you mean?

**Respondent: Attending to us well like for example in our clinic at the health Centre we are over 400 patients and we only go for clinic on Thursday and we only have one doctor attending to us. I was there in May and I was booked again for clinic in August. There is a problem with doctors and that’s why we have to wait for many months and we would love to be attending clinic at least every month**

**Moderator:** How can we solve the drugs problem?

**Respondent: We have stayed for long without getting drugs. We have been buying all the drugs from last year August. We had a one problem, this drug called…6:26-6:30(Not clear) came in late and they came when they were already expired. We only used them for one month and realized that they were expired we were not getting any drugs at that time and those that were available did not have demand especial the drug that I am using that is called …7:05… (Not clear). It has really been long since I found it at the hospital. I can say that I have not been able to get those drugs at the hospital for the last one year and so I just have to buy**

**Moderator:** What of this problem that you mentioned about having a big hospital. What would be the solution to that?

**Respondent: I think patients need to be checked every time especially those that if they have been taking antihypertensive or diabetes drugs for long. They need cardiac checkup or even head checkups coz sometimes you can be feeling a headache. For me I was doing well with this National Hospital Insurance but for now I can pay because I am pressed. It becomes a challenge when I am needed to do such a test because I will be referred to a hospital like Kenyatta. I was referred to Kenyatta the other day because of a problem that I had. It really costs a lot. I would propose free tests to patients who have had this condition for long because it’s good to have frequent checkups because sometimes the eyes are disturbing, you take long without doing a kidney test. It cost above 2000 shillings to do a kidney test at Kenyatta. The big problem that we have mostly is head ache. I was almost hit by a stroke due to high blood pressure. I went round in Nairobi and the place that I got treatment was at Thika level 5 hospital. That’s where I went and I was served so well. I had to lie to that doctor that I live in Thika yet I was still living in Viwandani. I told him that I know that the services are offered for free at Mbagathi at Kenyatta but I couldn’t go there because I was in a bad condition. The doctor treated me and I got healed. It would be better if we found these services easily**

**Moderator:** You told me that you go to the health centre for your clinic

**Respondent: Yes**

**Moderator:** Is this health centre located within the community that you stay in?

**Respondent: It is located in Viwandani and it’s the only Health Center in my community**

**Moderator:** What services do you get when you go for clinic?

**Respondent: We get services like blood pressure checkup, they check the weight and for me I like being checked on my sugar level but its two months since I did the last checkup coz sometimes the required gadgets are not available at the hospital so the doctor just does an outward test and if he doesn’t do that he checks your blood pressure and tells you that it is ok but since you know your body condition then you just go to another facility and pay 100 bob for you to be checked better**

**Moderator:** Do you get advice when you go for clinic?

**Respondent: Maybe if we go one by one because we only have one doctor serving many patients and it’s the same doctor who checks both the hypertensive patients and the other patients. The doctor is always overwhelmed coz if it is like on Thursday, she has to attend to the other patients, the diabetic patients, the ones that are hypertensive and still the one that attends to emergency cases. We have to report at the facility early coz by doing that the doctor can extend and serve us till late in the evening. Maybe you wanted to know your diabetes status and so you left the house in the morning without taking breakfast so you have to stay there hungry**

**Moderator:** Do you pay for the tablets that you get there?

**Respondent: We don’t pay for drugs when we get them at the hospital but most of the time we only get one type like last time I got only one type and it was in May and my next clinic should be in August. I can’t see my doctor before then**

**Moderator:** Do you know the names of the drugs that you are taking?

**Respondent: They are called…13:14-13:18… (Not clear)**

**Moderator:** How do you take them?

**Respondent: I take 13:28… (Not clear) in the morning at around 8:00 and 9:00 and in the evening I take…13:37… (Not clear) as I go to sleep**

**Moderator:** You told me that you were booked for august clinic when you went for your last clinic in May. Do you attend your clinics on a monthly or weekly basis?

**Respondent: We used to go there on a monthly basis but it was changed because of this pandemic. We were told that we are not supposed to mingle with other people because of our condition unless one is sick. We are given drugs that can last for a longer period so that we can take them as we access ourselves from home. I am just at home now**

**Moderator:** On to the other question

**Respondent: Yes**

**Moderator:** What do you think are the reasons why your blood pressure is not controlled?

**Respondent: From my side I can say that I had clotting problem in the year 2009 that led to swelling of my legs and instead of the doctor checking if it was due to blood pressure problem, he said that it was a clot. When I was being treated of the clot at Kenyatta that’s when they knew that I had blood pressure condition though I also came to know by myself that the other condition might have been caused by blood pressure so I had blood pressure before but it had not been detected**

**Moderator:** Do you have any other conditions apart from that?

**Respondent: I came to know at around March that my sugar levels were very low. It had reached 3 and I had a headache problem that I had to go to Mbagathi but they couldn’t tell the problem but through APHRC, there is one person who came to my house and checked me and she realized that my sugar levels were low. I was so happy coz she told me what to do and according to the way I had been advised before that I should avoid sugary foods, I just knew that that could have been the reason and so I decided to be using a little sugar when I feel like my body needs sugar**

**Moderator:** How is your normal day?

**Respondent: The way I work?**

**Moderator:** Do you leave the house or you just stay indoors?

**Respondent: like for now I am not working and my husband is not working too, we are just doing some casual jobs. I just do my normal house chores like the woman in the house and when I am done I take my shawl and a mask and I just walk within the village**

**Moderator:** How is the drugs procedure?

**Respondent: I take my drugs in the morning and in the evening on a daily basis**

**Moderator:** What are the barriers to seeking hypertension care services in this community?

**Respondent: I can’t understand the barriers because am the only one who has this condition in my family though I was told that my elder brother also has this condition**

**Moderator:** How long do you take to be served when you go to the health centre for your clinic?

**Respondent: We are supposed to be there by 8:00am and sometimes we go there as early as 7:00 am because of the queues. For now it’s worse because sometimes we have to stand because of social distancing especially when we find people sitting on the bench. The hospital is small and maybe many people came early. The doctor wants us to be there very early so that she can finish with us before she starts attending to the other patients**

**Moderator:** As an individual, what else can make you not to go and get quality care?

**Respondent: There is nothing that can make not to go for treatment just that I didn’t take this condition seriously like I do now but now I know how I am supposed to stay with my condition because of the problems that I have had. I like going for checkup so that I can know my condition**

**Moderator:** How is the quality of care in the facility that you go to?

**Respondent: I can say it is ok based to my ability but the problem is that the drugs are not available always but it is getting better because the doctor that we have attends to us very well. We had a doctor who was attending to us well but the one that we have currently attends to us well and she listens to us one by one**

**Moderator:** Ok. What do you think about policies or what the government is doing to that hinders hypertension care in your community?

**Respondent: What I can tell the government is that they just make hypertension and diabetes treatment free because it becomes a challenge if you have blood pressure and you have kidney problems. I told you that I almost got a stroke? It was a big problem coz we used a lot of money on that and the drugs were so expensive and sometimes I couldn’t get the drugs that I was supposed to use. I used to go all the way to Thika to seek treatment and getting drugs was a problem**

**Moderator:** You told me that there drugs that are available and some are not available. What would be the solution to that?

**Respondent: There is a time I asked the doctor to know what is it that she had not done because the drugs are available like for now the drugs that are available don’t have demand like the drugs used by pregnant mothers who are hypertensive but those that have a lot of demand like …23:40… (Not clear) have not been available the whole of this year**

**Moderator:** We are about to finish

**Respondent: Yes**

**Moderator:** How has the current COVID 19 situation affected how you get hypertension care on this community?

**Respondent: It has affected me because I used to see my doctor monthly but nowadays I see my doctor after 3 months. That’s one of the problems and the other problem is that I worry much about my condition because I cannot do my business like I used to do because I cannot go to places that have many people. I am always worried because of my condition. I think it will be worse for me if I got that condition coz I don’t have the same ability to fight as those people who don’t have other diseases**

**Moderator:** Is there anything else that you would like to add?

**Respondent: The other thing that I can add is that we have a lot of stress during this period because we could access treatment easily but now we cannot go out of Nairobi even you can’t get the service that you want is not available here. When we talk with other patients at the health centre, most of them say that they like visiting a doctor based at {Place} but they can’t go to {Place} now**

**Moderator:** Ok, is there any other thing that you would want us to talk about in regards to hypertension?

**Respondent: What I would like to say is that a machine should be brought at the health centre because we use medicine for like the first year, second year and you find others that have been attending clinic for 10 years. So we need a machine so that we can be doing kidney tests easily or we get connected to another facility that is not expensive. We can get a facility that we can be able to pay for cardiac checkups, head checkups. The other thing is …27:02-27:04… (Not clear). It is very important. We can have that machine and have a doctor to teach us on how we can go about it because some of the patients are old and others are young because I do see young people with diabetes so that they can be told how they can live**

**Moderator:** Are you ok up to there? Could you be having any other question that you would like to ask me?

**Respondent: There is this thing that always keeps on disturbing me. I can just be sitting alone then all of a sudden I just feel like my heart start beating faster for some time or I feel like my body is frozen and when that happens I normally go to see a doctor to check my blood pressure condition because I don’t have a gadget that can help me do the test on myself. I have to pay 50 bob for a test and you find that I don’t even something to eat because of this current situation that we don’t know when it will come to an end. Let me tell you of what happened, there is something that I did, I don’t know what I was thinking of. I took the drug that I normally take in the morning and in the evening I went at the place that I keep my drugs and took the drug that normally take in the morning and so I used it twice in a day and now I asked myself if I will take the drug in the morning now that I had taken it at night. What came to mind was that if I had my own machine then I would check my blood pressure in the morning for me to know if I will use that drug at that time or not but now that I didn’t have it, I just woke up in the morning and when it reached 10:00am I felt like I couldn’t manage and so I took the drug again but I was so worried.**

**Moderator:** Ok

**Respondent: I would really be happy if I had a machine because I would be able to monitor myself**

**Moderator:** Ok. Thank you so much for the information that you have given me and I believe that it will help us in our research. Thank you so much for your time

**Respondent: Thank you**

**Moderator:** Good day. Bye

**…END…**
