# Supplementary material for: A socio-ecological framework examination of drivers of blood pressure control among patients with comorbidities and on treatment in two Nairobi slums; a qualitative study
Source: PLOS Glob Public Health. 2023 Mar 10;3(3):e0001625. doi: 10.1371/journal.pgph.0001625 (PMC10021823; doi:10.1371/journal.pgph.0001625)
Supplement: S1 File — (ZIP) [file pgph.0001625.s001.zip › Community/KOCH_IDI_UHTNC_200710_002.docx]

**Moderator: {Name}**

**Code: KOCH_IDI_UHTNC_200710_002 (**same interview as KOCH-IDI-UHTN-200710-001)

**Moderator:** You confirm that I have read to you and you have understood the information that I have read to you. You have been given an opportunity to consider the information, ask questions and tour questions have been answered satisfactorily

**Respondent: Ok**

**Moderator:** You understand that you participation is voluntary and you are free to withdraw at any time without giving any reasons without any of your legal rights being affected

**Respondent: Yeah**

**Moderator:** You understand that the data collected during this study maybe looked at by individuals where it is relevant to your taking part in this study. You give permission for these individuals to access your data

**Respondent: Yes**

**Moderator:** You confirm consenting to be audio recorded and you also consent to the use on anonymized verbatim quotations

**Respondent: Yes**

**Moderator:** You are happy for your data to be used in future research?

**Respondent: Yes**

**Moderator:** You agree to take part in this study?

**Respondent: Yes**

**Moderator:** This community has been identified to have a high burden of uncontrolled hypertension which is a leading factor to premature deaths and disability. I am trying to gather information about hypertension care in your community. To avoid hypertension related complications, it is recommended that people with high blood pressure can change their lifestyles in regards to diet, physical activities, smoking, alcohol consumption and using blood pressure medication**.** So tell me about your experience with having high blood pressure**.** Tell me about your experience with having high blood pressure

**Respondent: I started feeling headache at the back of my head and on the sides at the ear and when the pain goes I used to feel like blood is coming out and I could also feel dizzy**

**Moderator:** For how long have you been having high blood pressure?

**Respondent: For six years**

**Moderator:** How often do you check your blood pressure?

**Respondent: I test like twice of thrice a week**

**Moderator:** Do you record your pressure?

**Respondent: Yeah, at the hospital**

**Moderator:** Can you remember your last blood pressure readings

**Respondent: Yesterday’s reading was 182/89**

**Moderator:** Do you have any other condition apart from high blood pressure?

**Respondent: No**

**Moderator:** Ok.What have you been told is your target blood pressure by your health provider?

**Respondent:** 100 or 90

**Moderator:** What do you mean when you say 100 or 90? Elaborate please

**Respondent: The best measurement should be at least 100 on the higher side or 90 on the lower side**

**Moderator:** Do you know the drugs that you are using?

**Respondent: Yes**

**Moderator:** What’s the name?

**Respondent: One is called nefidepine and the second one is called enzoprine and the third one is …4:26… (Not clear)**

**Moderator:** How do you take them?

**Respondent: I take 4 in the morning and 4 in the evening**

**Moderator:** How many drugs were you taking when you were diagnosed with high blood pressure?

**Respondent: I started with a half, then a full one the later on two then I started taking four in the morning**

**Moderator:** Have you been using the three types of drugs from before?

**Respondent: Yeah, since then**

**Moderator:** How has having high blood pressure affected you?

**Respondent: My blood pressure goes down for maybe 2 hours after I take the drugs and at some point later my pressure blows high that I start sweating**

**Moderator:** You have had this condition for some time?

**Respondent: Yeah, 6 years**

**Moderator:** How has it affected your life for these years?

**Respondent: It has not affected my life but maybe when I go for an injection that’s when I feel good. I can go for a whole month even two. I go for that when the tablet is not working**

**Moderator:** Apart from using drugs, how else do you manage your blood pressure?

**Respondent: You mean by drinks or by food?**

**Moderator:** Apart from using drugs, how else do you manage your blood pressure?

**Respondent: I go meet my fellow women where we share ideas to avoid thinking so much or I can to a noisy place like where I was then and try to think of good things**

**Moderator:** How is your diet?

**Respondent: For my diet, when I get food, like for vegetables, I normally take a lot of greens with a small piece of ugali and I use very little salt. I also east fruits**

**Moderator:** What about exercise do you do exercise?

**Respondent: Yeah, I walk from here to number ten on foot**

**Moderator:** How is your normal day?

**Respondent: It’s always good**

**Moderator:** I mean, do you normally leave your house or you just stay inside

**Respondent: I just stay in the house**

**Moderator:** What else do you do to control your blood pressure?

**Respondent: Sometimes I try to be busy doing some work at home then after that I relax a little then try to get something to drink then I relax**

**Moderator:** Who do you see when you go to the hospital for your hypertension clinics?

**Respondent: I normally go to see a doctor at the health centre**

**Moderator:** Do you normally see a doctor or a nurse?

**Respondent: I always see a doctor**

**Moderator:** What can you say in regards to the way your health care provider is managing your high blood pressure

**Respondent: I can say that he manages me and if my pressure rises higher he gives me money and writes a letter to refer me to a bigger hospital where I get admitted**

**Moderator:** What are your views concerning your health care provider?

**Respondent: He is always here but he went home and got locked there**

**Moderator:** Have you ever soughted treatment elsewhere?

**Respondent: No**

**Moderator:** Where can you get antihypertensive services in your community?

**Respondent: Sometimes am helped by those people who bring medical services here. At this moment there is one called {Name} who comes to help me**

**Moderator:** Which clinic do you attend?

**Respondent: It’s called {Place} clinic**

**Moderator:** Is it public or private?

**Respondent: It’s public**

**Moderator:** What kind of services do you receive when you go there?

**Respondent: We are advised on how we can live with this condition, how we can be eating or drinking like using just a little sugar or even taking tea without sugar**

**Moderator:** Have you ever gone to seek medication outside Korogocho?

**Respondent: Yeah, in Kiambu**

**Moderator:** Why?

**Respondent: Because of high blood pressure**

**Moderator:** Why were you not able to get that service at Korogocho?

**Respondent: Because they don’t have the injections**

**Moderator:** How were you served in Kiambu?

**Respondent: I was given drugs and the doctor was so close to me, talking to me. The doctor couldn’t leave my side because the pressure was so high and he thought that at any time I can die. The doctors always sit there. They normally exchange shifts**

**Moderator:** Do you receive drugs for free or you pay from your pocket when you go to the clinic?

**Respondent: We are given for free but sometimes when the drugs are not there we are told to go buy and when we don’t have money we get a Good Samaritan who offers to buy for us because they know that this condition is dangerous**

**Moderator:** How do you attend your clinics?

**Respondent: I go once in a month then during the other days I go thrice a week just to check my blood pressure**

**Moderator:** Do you have any difficulties in managing your blood pressure?

**Respondent: I don’t experience**

**Moderator:** You don’t have any difficulties in managing?

**Respondent: No I don’t experience any difficulty**

**Moderator:** You had talked about financial challenges before, was the bill cleared by insurance or you paid cash?

**Respondent: My friends helped me pay**

**Moderator:** What are your views on age in regards to pressure, could that be a challenge?

**Respondent: My age?**

**Moderator:** Yes, your age

**Respondent: I would say that it’s a challenge because I don’t have anything. It’s really a big challenge to get food, paying rent is a challenge. When I think of that my pressure becomes worse and when I go to measure my blood pressure I find it very high**

**Moderator:** Did you say you don’t have other conditions apart from high blood pressure?

**Respondent: Yes**

**Moderator:** You are using three types of drugs?

**Respondent: Yes**

**Moderator:** Do you use these drugs as prescribed?

**Respondent: yeah, very well. I do take them at 7 if they are supposed to be taken by 7. In the morning and in the evening**

**Moderator:** Are you using alcohol or cigarettes?

**Respondent: No, I am saved, I don’t take those things**

**Moderator:** You told me that you were admitted at Kiambu and your family helped you to raise money that was used to settle the bill. Are there any challenges from your family that make it difficult for you to maintain blood pressure control?

**Respondent: No**

**Moderator:** What of the place where you live?

**Respondent: There are mothers who assist me when I don’t have food to eat. They bring me food or even bananas and they tell me to cook because my son is not capable. He was stopped from working after cases of COVID 19 were identified in Kenya.**

**Moderator:** Looking at your care providers, how well is the treatment they are offering you?

**Respondent: It’s ok**

**Moderator:** And where you attend your clinic, looking at the way they treat you, would you say that they normally do a good job to everybody

**Respondent: Yes, it is ok**

**Moderator:** How are the working hours for the clinic that you attend?

**Respondent:** It is good. We normally report at 8, given drugs and then advised on how we can stay

**Moderator:** You said that you are given advises on how to stay, are the advices ok? Do they really satisfy you?

**Respondent: Yeah, it’s ok**

**Moderator:** When we look at the health systems, do you get drugs?

**Respondent:** Sometimes we get them but there other times when I go for 3 drugs I find that they only have one but the doctor helps me on how to get the other ones

**Moderator:** How does the doctor assist you?

**Respondent: He gives me money from his pocket to help me go get the drugs**

**Moderator:** How is the foundation of the facility?

**Respondent: It is ok**

**Moderator:** You said that you are o with their working hours?

**Respondent: Working hours are very ok.**

**Moderator:** Is the space at the clinic enough for all of you?

**Respondent: Yes**

**Moderator:** Have you ever been told about guidelines for people with high blood pressure?

**Respondent: We have not yet been told bet we were told that we will be taught on that when we attend next week clinic**

**Moderator:** What do you think could be the possible solutions to the challenges that you have told me. You mentioned about finances when you talked of the day that you were admitted and your friends had to contribute to pay for your bill. What do you think could the solution to that financial challenge?

**Respondent: Themselves they don’t have work; they just contribute to help me get money**

**Moderator:** You also mentioned that your son is not working due to this COVID19, what you think would be the solution to that

**Respondent: It can be that I am in big problem**

**Moderator:** I mean a possible solution

**Respondent: It becomes tough for me to pay rent**

**Moderator:** How has the current COVID 19 situation affected how you get hypertension care in your community?

**Respondent: I normally go to the health centre**

**Moderator:** I am asking how the current COVID 19 situation has affected how you get hypertension care in your community

**Respondent: It becomes a burden**

**Moderator:** Can you elaborate

**Respondent: It becomes tough because we can’t get food, money to pay rent, when you go to the hospital you find that instead of being given the three drugs you are only given one because they don’t have the other 2. It becomes a challenge because even the drugs don’t reach at the hospital**

**Moderator:** You talked about money, what would be the possible solution to this

**Respondent: The best solution was for me to benefit by getting treatment and living in a way that will help me fight pressure**

**Moderator:** What can the health providers do differently?

**Respondent: The only different thing they can do is advise me to go to the hospital when my blood pressure is high, they can also help me get transport like I said earlier so that I go get treatment from a bigger facility**

**Moderator:** What can be done differently at the facility that you normally attend?

**Respondent: The different thing could be when they don’t have the injections; I am forced to call my friends to contribute money to buy that drug for me to be injected**

**Moderator:** Is there anything else that you would want us to talk about in regards to hypertension?

**Respondent: Maybe you help me on the other side**

**Moderator:** You need to be helped in regards to pressure?

**Respondent: Yes, even on how I can pay my house rent and even on how I can get food so that I can be able to take medicine**

**Moderator:** Am happy for the time that you have given me and I see that this will help us as we continue doing our research. Thank you so much

**Respondent: Amen**

**Moderator:** Have a good day

**Respondent: Thank you**

**…END…**
