# Supplementary material for: A socio-ecological framework examination of drivers of blood pressure control among patients with comorbidities and on treatment in two Nairobi slums; a qualitative study
Source: PLOS Glob Public Health. 2023 Mar 10;3(3):e0001625. doi: 10.1371/journal.pgph.0001625 (PMC10021823; doi:10.1371/journal.pgph.0001625)
Supplement: S1 File — (ZIP) [file pgph.0001625.s001.zip › Community/KOCH-FGD-200807_1015_01.docx]

**Moderator: {Name}**

**Code:** **KOCH-FGD-200807_1015_01**

**Moderator:** This community has been identified to have a high burden of uncontrolled hypertension which is a leading factor to premature deaths and disability. I am trying to gather information about hypertension care in your community. To avoid hypertension related complications, it is recommended that people with high blood pressure can change their lifestyles in regards to diet, physical activities, smoking, alcohol consumption and using blood pressure medication**.** So tell me about your experience with having high blood pressure**.** Tell me about your experience with having high blood pressure

**R1: My experience is staying away from many issues that can make get angry or make me think a lot. Like for example now there are no jobs and there is no money and so it is your responsibility to put your mind straight so that you wouldn’t be affected with this condition. You also need to commit yourself or avoid them because this condition gets worse when you engage yourself in many thoughts. It became worse when you come across something that annoys you, when you realize that you don’t have money so you just have to avoid them**

**Mod:** Let us hear from respondent number 2. Tell us about your experience with having high blood pressure

**R2: Just as R1 said, my heart always beats that I even feel weak when I quarrel with my kids. When such happens, I normally leave my house to go somewhere I can relax a bit then I come back later. That’s according to me**

**Mod:** R3 tell us about your experience with having high blood pressure

**R3: When you have this condition you will just know when your blood pressure is high because that moment you will be feeling weak and you always feel like you don’t like anything. Whenever you see people talking you just think that they are talking about you when you go to the house you don’t en see that the utensils are dirty, you just sleep. Problems are there**

**Mod:** R4?

**R4: Hypertension is a bad condition because blood pressure can rise at any time and it doesn’t matter if you are at home, on the road or anywhere else. It is a condition that puts you in a situation that you can die at any time. My experience is that I do ride a bike when it is high. There many issues that leads to this and can be found at home, places of work and life challenges especially with us who are in this age of bringing up kids. There is a time I was thinking that those people that are done with bringing up kids are much better compared to the others because every now and then you will hear this and that from the kids and their mother yet all those people that you stay wit don’t have high blood pressure you go to a seminar where you are taught on what you should eat and the food that you are advised to eat are expensive compared to the food that is prepared for the other people at home and you can’t buy your food and let others go without. You are forced to eat what is available because that’s what can be used by the majority at home. This endangers you to an extend that it becomes hard to control your blood pressure because you are told to eat traditional vegetables but you end up taking what is available. Secondly those people that you eat with them are the same people who always make noise. There need to be understandings with either your wife or husband and when it comes to that point when someone comes with noise like you know how ladies are. When you calm down that’s when they go high. I think that the government should come up with civic education to inform people how they should stay with patients who have this condition at the same time you find that you are somehow alone and you end up forgetting taking medicine because blood pressure involves forgetfulness. There is no one to remind you and the others don’t care because they have not been taught or told to be reminding me as a patient or ask me if I have taken drugs. You end up forgetting to take your drugs today, tomorrow you forget and this makes the blood pressure to rise and you find yourself taken to the hospital again. With my experience I have realized that blood pressure is a condition that is unpredictable. You can’t know about the next minute**

**Mod:** R5, kindly tell us about your experience with having high blood pressure

**R5: I can say that this blood pressure condition is not a good thing. You just find yourself with it without knowing where it came from but the issues that you keep in your brain like for example quarrelling, many thought, such things make you develop that condition unknowingly. You just feel like you have a headache and when you go to test then you find that your blood pressure is high but when you buy medicine from a chemist and take them then you feel like your body start cooling down so you just need to avoid those things and if you do that your blood pressure will be normal**

**Mod:** Starting from R1, tell me for how long have you been having high blood pressure?

**R1: it’s really been long**

**Mod:** Like how many years?

**R1: From 2005 up to date**

**Mod:** R2, for how long have you been having high blood pressure?

**R2: I knew that I was having this condition when I was delivering my 2^nd^ child in the year 1992**

**Mod: From 1992 to date?**

**R2: Yes, up to date**

**Mod:** R3, for how long have you been having high blood pressure?

**R3: The year 2001 is when knew that I have it**

**Mod:** 2001upto now?

**R3: Yes**

**Mod:** R4?

**R4: I knew about it in 2014**

**Mod:** What about you?

**R5: I knew that I have it on the day that I was checked and I can’t remember the date**

**Mod:** Which year was that?

**R5: Let me check here**

**Mod:** Is it on the same day that we checked you?

**R5: Yes. That’s when I knew**

**Mod:** Which year was that?

**R5: It was in 2018**

**Mod:** So we can see that everybody has been having this condition for a long period

**All in unison:** **Yes**

**Mod:** Starting again from R1, how often do you check your blood pressure?

**R1: You mean checking blood pressure measurements?**

**Mod:** Yes

**R1: I normally don’t take long. Maybe after like 3 months. I always go to check so that I can know how I am doing**

**Mod:** Where do you go for checkup?

**R1: I used to go to {Name of the facility} but the facility was closed and I changed and now I go to the facility located at the {Name} place**

**Mod:** Do you note down your blood pressure measurements after you have been checked?

**R1: That is always done by the person who does the check up**

**Mod:** What were the measurements the last time you checked?

**R1: I think it was 218**

**Mod:** 218

**R1: Yes**

**Mod:** Over what?

**R1: I cannot remember that**

**Mod:** Is it noted down?

**R1: Yes**

**Mod:** Do you go with the records or you leave them there?

**R1: I go with a copy and leave another copy at the hospital**

**Mod:** Did you carry your records?

**R1: I didn’t carry any because I was not told to carry anything**

**Mod:** Ok. R2, tell us how often do you check your blood pressure?

**R2: I go for checkup after one month**

Mod: **One month?**

**R2: Yes**

**Mod:** Ok. And where do you go for your blood pressure checkup?

**R2: At the {Name} place**

**Mod:** Do you record your blood pressure measurements somewhere?

**R2: Yes, in this book**

**Mod:** Kindly check for me the last blood pressure measurement readings

**R1: 158/71**

**Mod:** R3 tell me how often do you check your blood pressure?

**R3: We were told to always back for checkup after one month but after with the current issues, we just go when we feel like because there are no appointments now. We just go for checkup and collection of drugs whenever we feel unwell**

**Mod:** Where do you check your blood pressure?

**R3: At the {Name} place**

**Mod:** Do they record down?

**R3: Yes, we are a group of diabetic and hypertensive patients. The doctor always writes down after measuring us. I have a book in the house**

**Mod:** Can you remember your last reading?

**R3: Yesterday it was at around 140 something there over 80**

**Mod:** R4, how often do you check your blood pressure?

**R4: I used to go to {Name of the facility}in 2014 but I stopped going when I felt that it had cooled down. I stayed for long until when I was found out that I had H pylori. I realized that blood pressure came back when I was treating H pylori. I was not diabetic in 2014 so but I realized I am diabetic when I was checked again on blood pressure. I was given drugs that I am still using as we monitor it. I have not gone to any hospital yet. I just pay and check at a chemist that has that gadget as I continue with life. I checked on Monday and I was supposed to go back again on the other Monday. When I checked on Monday it as 145/79**

**Mod:** Ok. R5, tell me how often do you check your blood pressure?

**R5: I have never checked again from the day you checked me that day. I can’t lie**

**Mod:** You have never checked?

**R5: Yes, I just went to the chemist where I was checked and given pain killer drug because of my head that was aching**

**Mod:** You had a headache that time

**R5: Yes and nowadays I just go but drugs at the chemist whenever I feel like am having a headache**

**Mod:** Have you been using any antihypertensive from the day you were diagnosed with high blood pressure in 2018?

**R5: I just buy drugs whenever I feel like my head is aching**

**Mod:** Ok. How do you get those drugs?

**R5: I get them at the chemist**

**Mod:** Do you go for any hypertensive clinic?

**R5: No**

**Mod:** Ok. R1, tell me if you have any other condition apart from high blood pressure

**R1: Sometimes I do have problems with my back**

**Mod:** R2?

**R2: I don’t know if this drugs that am taking made me to have allergy because sometimes I cough to an extend that I even don’t sleep at night and I also have pneumonia**

**Mod:** R3. Do you have any other condition apart from high blood pressure?

**R3: Arthritis**

**Mod:** R4?

**R4: Just as I told you, I am diabetic and I also have ulcers**

**Mod:** Ulcers?

**R4: Yes**

**Mod:** R5, do you have any other condition apart from high blood pressure?

**R5: No I don’t have**

**Mod:** There isn’t?

**R5: No**

**Mod:** Let us go back to R1. Has your doctor ever told you what your target normal blood pressure should be?

**R1: No**

**Mod:** Ok. R2. Has the doctor ever told you what your target normal blood pressure should be?

**R2: There is a time I went to deliver at {Name of a facility} where I was told that it was high but I don’t remember what I was told it should be**

**Mod:** But you were told

**R2: Yes, I was told**

**Mod:** R3?

**R3: We just read that from people who tell us that it is ok if it is over 80. The number above doesn’t matter a lot**

**Mod:** Who told you that?

**R3: My fellow patients**

**Mod:** Other patients at the clinic?

**R3: Yes, the doctor has never told me**

**Mod:** Your doctor has never told you?

**R3: Yes. He just checks us and leaves us there. You are the one to ask how it is and if it is bad he tell you and asks if you are taking drugs**

**Mod:** R4?

**R4: I have never been told**

**Mod:** You have never been told?

**R4: No. when I went there I was told not to stop taking drugs because to him it was like I am using witchcraft. That’s what the doctor told me but he never mentioned how it should be**

**Mod:** Ok. R5, have you ever been told you what your target normal blood pressure should be?

**R5:** **I don’t know the maximum pressure**

**Mod:** Back to R1. Tell me about the antihypertensive drugs that you are taking. How many tablets are you taking?

**R1: I always divide my tablet into two. I take one half in the morning and another one in the evening. So I take 1 tablet a day**

**Mod:** R2. Tell me about the antihypertensive drugs that you are taking. How many tablets are you taking?

**R2: I take 1 tablet and there is also another one also that I cut into two and in the evening I take 1**

**Mod:** So you take 2 per day?

**R2: Yes**

**Mod:** R3. How many tablets do you take?

**R3: I take 1 full tablet and there is another one that I cut into two**

**Mod:** You mean 1 and a half?

**R3: Yes. I take one full tablet and cut the other one. There is a full one and a half one**

**Mod:** R4, how many tablets do you take?

**R4: …21:19... (Not clear) I take 1and the other one that they say that they take 1 I also take 1 it is called enarapline. For blood pressure I do take a full tablet instead of half**

**Mod:** So you always take 2 tablets per day

**R4: Yes**

**Mod:** R5, how many tablets do you take

**R5: I said that I only take drugs when I feel like am having a headache and I only take 2 tablets**

**Mod:** You take 2 tablets

**R5: Yes**

**Mod:** On the day when you feel like having a head ache?

**R5: Yes**

**Mod:** Ok. Back to R1, have they been adding you more tablets or have they been reducing them from the day that you were told that you are hypertensive?

**R1: They have never been added**

**Mod:** How have they been?

**R1: They have been the same**

**Mod:** R2. Have they been adding you more tablets or have they been reducing them from the day that you were told that you are hypertensive?

**R2:** **There is a time when I used to take one but nowadays I take 2**

**Mod:** So they were added?

**R2: Yes**

**Mod:** R3, have your tablets been added or reduced from the time that you were told that you are hypertensive?

**R2: I used to take one then I was told to take a half but at the moment I am taking one and a half. The number of drugs is increasing instead of reducing**

**Mod:** R4?

**R4: I started with a half a tablet and now I am taking full**

**Mod:** So you have added

**R4: Yes**

**Mod:** R5. You told me that you have always been taking two tablets. Have you been adding or reducing from the time you started?

**R5: I just take 2 tablets when I feel like my head is aching**

Mod: You take only 2

**R5: Yes, just two**

**Mod:** R1, tell me how high blood pressure has affected your life. You had told me many things before when I asked you about your experience with having high blood pressure but can you tell me on how blood pressure has affected your life

**R1: I can say that it has affected my life because I normally feel my heart beating faster when I am asleep. I always feel like there is a drum in my chest to an extend that I sometimes ask myself at that moment if I am dying. That’s how it affects my life and again it becomes a problem when I see anything that shocks me**

**Mod:** R2, you also told me about your experience earlier, tell me what you think that you didn’t mention in regards to the way blood pressure is affecting your life

**R2: It affects me in that I always feel like beating that boy that was here whenever my blood pressure is high. He doesn’t take in instruction and maybe he doesn’t understand because he is a kid**

**Mod:** R3, how has blood pressure affected your life?

**R3: I can say that blood pressure makes one scared. You can even be scared off by a small child. There are some works that you cannot do because you are hypertensive. Your body can’t allow you even if you decide to do some kind of jobs. You just do what you can do to live and again you can’t see far**

**Mod:** R4. You had also told me about your experience, is there anything that you can add in regards to the way blood pressure is affecting your life

**R4: It has affected me directly by removing me from my work because I cannot ride a bike because I should avoid being scared of other vehicles on the road. Secondly I do forget a lot. I wouldn’t have come here if you didn’t remind me coz I had already forgotten after we talked yesterday. I can put something somewhere and forget where I kept it. I have to find someone to help me look for it again. Like for now I don’t even know where my ID is. It has affected me in life and in my thoughts. Coming to the society, there are people that I can’t stay close to them. I have to avoid and reduce the number of people that I interact with.**

**Mod:** R5, How has blood pressure affected your life?

**R5: My heart beats hard and faster and I also do feel headache when I am shocked by anything or when am told of any bad news**

**Mod:** Back to R1, Apart from using antihypertensive, what else do you do to ensure that your blood pressure becomes normal?

**R1: I avoid places that are noisy. I prefer sitting with other older people where we can just talk about life, job and what we can do to better or lives but I don’t like engaging myself in quarrelling or wrangles because my blood pressure rises when I go to such places. That’s why I avoid such places**

**Mod:** Tell me about the foods that you take or body exercises

**R1: With body exercise, personally I don’t like boarding matatus or bodabodas, I prefer walking. I can walk from here to Dandora phase 5 and back on foot but food is a challenge just as my friend had said before. Based on what you always say, a person with diabetes or hypertension is supposed to eat certain types of food but in my community you can’t choose what to eat because first you don’t have money to buy what you are supposed to eat, you just eat what has been prepared by your wife and kids because you don’t have money. You can’t say that you can’t eat certain foods yet you don’t have money to buy what you want to eat. You have to eat that ugali with sukuma or any other thing that is available. You just eat for you to live but not as you would want to eat**

**Mod:** R2, Apart from using drugs, what else do you do to keep your blood pressure under control?

**R2: I try walking on foot. I also do avoid places that are noisy and quarrelling**

Mod: What else have you not mentioned?

**R2: Like what?**

**Mod:** Like exercise, food

**R2: With food it is hard because sometimes you don’t eat at lunch time and what you can get is ugali and cabbage. You just have to eat that food because of problem and the income that is very low. We only manage one meal coz sometimes you can’t manage to get breakfast so you just eat what is available. It is only God who helps us**

**Mod:** R3, Apart from using antihypertensive, what else do you do to ensure that your blood pressure becomes normal?

**R3: You cannot direct blood pressure on what to eat when you have it. It is not me to decide on what to eat but my condition will direct me and if you fail to do that then what you will feel later will make you feel like you rather die. When you are told to stop taking salt please do that even if it means adding water in you food, sometimes I add water in my tea because of sugar. When you visit your neighbors or your friends and find that they have already cooked, some might even say that you are boasting coz sometimes you have to reduce the food that they serve you or even sometimes you even feel like adding some water to reduce the taste of salt and at the same time you have to control the condition using drugs. For exercise, my work involves walking; I can go to the market and buy some vegetables and walk around hawking in {a place} and {a place}. That’s the exercise that I do. I don’t sleep because if I say that I want to sleep with this blood pressure condition then it will be worse.**

**Mod:** Ok. R4, tell me what you do to control your blood pressure apart from taking drugs

**R4: There are some ingredients that I mix when I have money. I have at some point mixed garlic, turmeric and ginger and I put them in my food. This makes my food taste like it is hot and you find that the same food has been prepared without cooking oil. When I take that food I normally feel that my blood pressure is ok but the problem is that you need money for transport to the market and to buy those all those things for you to use them and when they are finished then you have to wait till when you get money again for you to go to the market again. I have been using that and I feel like it is very good but for now I am not using it.**

**Mod:** Ok

**R4: Secondly, I like listening to music and music calms my mind but you find that the music that I like listening to is not the same music that others would like to listen to. There is music that brings down blood pressure and there is music that makes blood pressure to rise yet those that are in the house prefer listening to the music that makes your blood pressure to rise. They don’t want to listen to your type of music**

**R3: We even switch off the radio**

**Mod:** You switch off the radio?

**R3: You just watch the TV and switch of the radio because others are really nagging and yet you don’t even know them**

**Mod:** What about physical exercise?

**R4: I have not considered doing physical exercise unless I decide to walk for a short distance because at this age I can’t engage myself in running. There is this person that was diagnosed with blood pressure and he thought that he could control it by running. He went for a running exercise at {a place} but I don’t know what happened, the guy just fell by the roadside and died. It was said that he died of corona yet he had gone for exercise**

**Mod:** Ok. Who informed you about this concoction that you told me about?

**R4: I was told by somebody and I think I also read it in the internet**

**Mod: You read it in the internet**

**R4: Yes, there is even another one that I read but I can’t manage to get it because I don’t have enough money**

**Mod:** R5, tell me apart from using drugs, what else you do to control your blood pressure

**R5: Sometimes I walk a lot especially when I have to go to places that are not very far. Do you know that there are other places that you can’t go on foot? Those are the places that I use a vehicle to reach but I prefer walking other places that are near and I feel like I can reach on foot**

**Mod:** What about food?

**R5: with food I can eat but for now I don’t have enough money and so I have to use what the money that I have can buy even if it is Sukuma, cabbage or any other type of vegetable with ugali**

**Mod:** Ok. R1, who do you see when you go to measure your blood pressure at the {Name] chief’s place?

**R1: I don’t know their names but I remember the one who used to attend to us there was called {Name}**

Mod: **{Name}**

**R1: Yes, but I don’t know the names of the other ones**

**Mod:** R2, you also told me that you also go to the {Name} place. Do you normally see the same person or who do you see when you go there

**R2: I always see a doctor called {Name}**

**Mod:** R3, who do you see?

**R3: I also see {Name}**

**Mod: {Name}**

**R3: Yes**

**Mod:** R4, you told me that you don’t go for clinics but you go to the chemist

**R4: I don’t know their names**

**Mod:** Who do you see in the chemists that you go to?

**R4: I always see a madam called {Name} at the chemist that I go to. She is the one who checks my blood pressure measurements**

**Mod:** Ok, R5, you told me that you have not been checked again

**R5: Yes**

**Mod:** Do they check your blood pressure when you go to buy drugs?

**R5: I just buy drugs**

Mod: You have never gone for clinic?

**R5: No**

**Mod:** Ok. Back to R1, What can you say about your health care provider at the {Name} place in regards to the way he is managing your blood pressure condition?

**R1: I can’t say that he attends to me badly because he normally asks me how I am doing and I answer him according to the way that I am feeling so I can say that he is bad. He is ok.**

**Mod:** Have you ever sought treatment elsewhere apart from the {Name} place?

**R1: No**

**Mod:** R2, What can you say about your health care provider at the {Name} place in regards to the way he is managing your blood pressure condition?

R2: He is not bad

Mod: What can you say about him?

**R2: He is good because he always checks me and if informs me if my blood pressure is high. He also tell me to stop using salt, he also tell me what do like doing exercise and keeping away from many thoughts**

**Mod:** Have you ever sought treatment elsewhere apart from the {Name} place?

**R2: Kenyatta, Kiambu, Mbagathi, at the sisters’ place and Tumaini**

**Mod:** What did they tell you that you think is ok?

**R2: They say that my blood pressure is very high. I stopped going to the sisters’ hospital because of financial issues. I even have their card with me**

**Mod:** Ok

**R2: I also have {Name} facility card**

**Mod:** So you have all the cards?

**R2: Yes**

Mod: Where will you go when you get money?

**R2: I usually go to the sisters’ hospital when the public hospital is not working**

**Mod:** Ok. R3

**R3: I can say that that facility is not bad because the always give us drugs when they are available and they always inform us when they don’t have drugs and another thing is that you can’t miss out on HTCZ but they tell us to go and buy the other drugs. They really try because that is more important**

**Mod:** Because you get one and buy the other one

**R3: Yes, you get one and buy the other one**

Mod: R4, You told me that always go to the chemist. What can you tell me in regards to the way he is managing your blood pressure condition?

**R4: Not only those that I meet at the chemist but even those that I met when I went to the health facility are not bad because I even got drugs there. They told me that the6y are monitoring to know how the doctor will follow up on me because I have never used diabetic drugs from the time I was diagnosed with diabetes because it has never been severe so am following up because we don’t have any history of diabetes at my place. Maybe it’s just something that triggered the sugar to make it rise. He explained to me very well because sometimes I can start using something that I will never stop**

**Mod:** R5, You told me that you don’t go for clinic and you also don’t go anywhere for checkup. What can you say about the person that you always meet when you go to buy your drugs?

**R5: I just buy drugs from a shop because I already know what is disturbing me and I always feel good when I take drugs so there is nobody that attends to me. For a shop keeper he just gives me drugs when I give him money**

**Mod:** So he has never told you anything

**R5: He can’t tell me anything.**

**Mod:** Ok. We will go to the fourth question. We still have like 2 more questions. So R1, where can you get hypertension care within our community?

**R1: You can get that if you go to the {Name} place, {Name} clinic but for now I don’t know if {Name of a facility} are still operating but there are several places. You can also be attended to if you go to the sisters’ place, At {a place} there is also a city council hospital where you can be attended to. There are many facilities.**

**Mod:** You told me that you always go to the{Name} place, what kind of services do you get when you go there?

**R1: The service that is get is hypertension care only, I don’t have another problem**

**Mod:** Is the hospital located within your community?

**R1: Yes**

**Mod:** I wanted you to tell me about services like treatment, blood pressure measurement and if those health care providers do give you advice.

**R1: Let me say that a person only speaks about the services that they get. I have never received those other services and so I can’t say if those services are or if they are not**

**Mod:** R2, you told me that you also go to the {Name} place. Tell me about the services that you get when you go there

**R2: I go there and they attend to me and give me drugs if they are available and if they don’t have they always tell me to go buy like for now I am coughing, they checked if I have TB and they found out that I don’t have it and they advised me to continue taking my drugs or I go buy. I can say that they really do help me**

**Mod:** What about pressure

**R2: They check my blood pressure and I always tell them if I have any other condition**

**Mod:** R3, tell me the types of services that you get

**R3: I can say that I get the services because even if I go there with another problem like there is a time when my legs were paining and I was referred to Kiambu where they did an X-ray and realized that I have arthritis. They attend to blood pressure and also other conditions and if he is not able to help you he always refers you to another place and he tell you that he cannot attend to you without the X ray images. That’s the good thing about them. You will have to go to where you have been referred to, come with the images so that he can attend to you but the problem is that they don’t have drugs because you will have to buy after he tell you that they don’t have the drugs but if the drugs are available they just gives us like I said before**

**Mod:** R4, tell me the type of services that you always get at the place that you visit?

**R4: I went to the hospital like a normal patient and I was told that I am hypertensive and they started treating me. Just like that**

**Mod:** What about the drugs?

**R4: I get drugs there enaraprene and**

**Mod:** So you get others for free and the others you buy?

**R4: Yes**

**Mod:** R5, tell me about the services that you get where you go

**R5: I don’t get any service because I have never gone anywhere apart from when I was checked and I was given this form**

**Mod:** Back to R1. Tell me the challenges you face in seeking hypertension care. You had told me that getting drugs is a problem and sometimes you also don’t get to eat the foods that you are supposed to be eating. Tell me any other challenge that you face in seeking hypertension care

**R1: The problems that I face is like sometimes I don’t have money to buy drugs and when I go to the hospital I am told that the drugs are not available and the doctor directs you to go buy the drugs at the chemist yet you don’t have money. You will just have to stay with your high blood pressure condition because you don’t have money. That’s one of the challenges that we face**

**Mod:** We will go in this order; we will talk about your individual factors that you think might be the reason as to why your blood pressure is not normal. You told me that sometimes you buy drugs when you find that you have one type of drug and you don’t have the other one, you also told me that sometime you find yourself getting angry. What do you think are the individual factors that make your blood pressure not controlled?

**R1: Reasons as to why my blood pressure is high?**

**Mod:** Yes

**R1: There are many factors, let’s say for example like for us men when you don’t have a job and you don’t have money, that could be a reason that might lead to high blood pressure coz you start thinking of what to tell your wife when you go back home. Children are looking up to you yet you don’t have money. There are many reasons that can lead to rise in blood pressure yet it is not your wish but it’s because of how life is. Us men go through a lot of issues like not having a job and not having money that lead to rise in our blood pressure**

**Mod:** What about you taking your drugs on time?

**R1: I take my drugs on time; I have never missed taking my drugs**

**Mod:** Tell me about the family or community factors that might hinder you from managing your blood pressure

**R1: On family, my friend said that us who are more matured or we have finished bringing up or taking care of our kids have no problems with our children and I want to say that there are problems for us who have big kids. At that time a boy feels like he is a grown up man and a girl feels like her breasts have grown enough. They can’t take in what you tell them. They always say that their dad is noisy. The boy will tell you to stay away from his life and by doing this they make your blood pressure to rise. Big children are also a problem and it becomes worse because they don’t take in what you tell them. This makes you feel like your kids think that you are not the one that sired them, they think that I am not their father and you start making noise or even arguing with yourself. You think of leaving and going away from them and find a place like in Maasai land in Kajiado because they are stressing you. There are things that you cannot avoid. This child comes home drunk yet you yourself you have never taken alcohol, the other one comes home beaten while you yourself you have never been beaten from your childhood. Some issues contribute to our blood pressure condition**

**Mod:** What are the health care provider factors that hinder you from managing your blood pressure?

**R1: I don’t have a problem with my healthcare provider and I have never had any thoughts caused by him**

**Mod:** What about the time that you take at the facility as you wait to be treated?

**R1: I have no problem with time because they always attend to us and give us drugs when we go there then we come back to our normal engagements**

**Mod:** What about trainings?

**R1: Trainings are ok because he always tells us not to use a lot of salt. Such types of trainings**

**Mod:** Tell me about the health system factors or what you think that the government should do to manage your blood pressure

**R1: I can say that I heard that there is this money that the government gives to the old people, youth, and development projects but they don’t think about us who have this blood pressure and diabetic conditions. I think that they should also consider us and know that we are going through hard time coz even we are not eating the way we are supposed to be eating. The government should consider us because this pandemic is stressing us**

**Mod:** And what about the drugs?

**R1: They should also consider the drugs because if you go to the hospital and you are told that there are no drugs and so you will be given one type and asked to go and buy the other one. That one also contributes. They should make sure that hypertensive and diabetic drugs are available at the hospital. It really surprises me that you don’t get some drugs at the hospital but when you go to the chemist you find them there yet a chemist is privately owned. Who is more powerful between the government and the private chemist owners? The government should be having everything and personally I think there is a challenge there**

**Mod:** R2, we will just go in the same order. Tell me the challenges that you face in managing your blood pressure condition starting with your individual challenges

**R2: Personally sometimes I don’t even get sleep because of this blood pressure condition. At night you don’t get to sleep even for a minute. You always hear when the rats are running around yet the drugs are not available and there is no money. You feel like the heart is beating, you don’t have money, you did not take drugs and that contributes to the problem and you feel like drums are beating in your chest so you start asking yourself when the morning will come so that I can see the light again. You wake up immediately when the cock crows yet you don’t have anything to do. You start going around your house washing or just even doing something for you to get tired so that you can fall asleep for a few minutes. That is the problem that I face**

**Mod:** What about you taking your blood pressure medicine as directed?

**R2: Sometimes you don’t take them as directed. There is a time that I went to the chief’s place and I was asked why I didn’t buy drugs that I was supposed to buy. I told them the truth and they told me that I don’t take care of myself. I told them that there is nothing that I can do without money, I just ask God to help me. The doctor told me that my condition is serious but for me there is nothing that I can do and so I just decide to continue that way. When I have money I do go to our place on the days that they are attending to HIV patients coz at least with 50 bob I can buy some drugs there.**

**Mod:** What are the family or community factors that might hinder you from managing your blood pressure?

**R2: My kids, like my first born takes a lot alcohol. There is a time I went to our area chief to ask him if he can help me on that because his alcohol intake is really bad. Sometimes I pick him from the trenches and that causes my blood pressure to rise because of such thoughts. I went to see the area chief yesterday because I wanted him to help us now that our kids are taking alcohol with this Corona but he was not available. You don’t get food and the kids are making you to think yet you are sick. We are really in a bad state just like my brother mentioned. If that is the eldest son so he is the only one that I can depend on yet he is the one sleeping in trenches coz of alcohol**

**Mod:** What of your health care provider’s factors? You told me that you go for clinics at the chief’s place

**R2: Yes**

**Mod:** What can you tell me about your health care provider located at the {Name} place in regards to the way he is managing your blood pressure

**R2: My health care provider tries his best. He attends to me well, I don’t have any problem with him coz he does what he can. There is nothing he can do when drugs are not available**

**Mod:** What about the trainings that you receive there?

**R2: He trains us well. He tells us not to take food that have a lot of oil and he also tells us to use just a little salt. There is a time when I was told that I should completely avoid salt because my blood pressure was very high**

**Mod:** What about the time that you take at the facility when you go for clinic?

**R2: He serves us well**

**Mod:** Time

**R2: I don’t have problem with time**

**Mod:** What are the government factors that can hinder you from managing your blood pressure condition?

**R2: I would request the government to consider us the way they do to people with AIDs. There is a time that I went to the village elder and asked him to write my name because he was writing people’s names. I requested him to write my name because I also have a problem with drugs and I take drugs like the other patients. He told me that he is writing 4000 names but for people living with AIDs. I told them to give me a slot because the drugs are the same but he told me that he can’t write names of patients who are hypertensive and I told him that it is ok.**

**Mod:** R3, we will go in the same order. Tell me about the individual factors that you think might be hindering you from managing your blood pressure

**R3: I have been trying to take my drugs from the day that I was diagnosed with blood pressure though it is hard to control just as this lady said. Our kids don’t take in instructions and my husband I can’t mention because he also used to take alcohol and I don’t know if he is the reason to why I have this condition or if it is my kids. There is a time that one of my kids was sick, this guy knows him. There is a time he came and they almost fought with him but it is because of his condition. I don’t know if that’s where it came from or it came from elsewhere. Problems are many in this community. We just hold on to that and when we go to clinic we are told that the government doesn’t consider hypertension to be a disease. They say that it is a lifestyle condition and we should avoid stress yet our homes are full of stress. You lest stress in the house as you go to seek treatment yet they tell you to avoid stress. Stress contributes a lot**

**Mod:** What about you taking your drugs on time as directed by the doctor?

**R3: I take drugs on time because these drugs should be taken at the exact time that you are told to take because they don’t work if you don’t take them on time. There is a time when HTCZ was not available at the hospital but people complained and it was brought. The government doesn’t consider this as a disease though they know how it can be treated but the problem is that there is no money**

**Mod:** On community and family factors you mentioned that your kids do disturb you at home

**R3: Yes**

**Mod: You always quarrel with them. Tell me about other community factors that you didn’t mention and you think that they hinder you from managing your blood pressure**

**R3: Pardon**

**Mod: You said that kids at home have their issues, they take alcohol**

**R3: Yes, they take Miraa**

**Mod:** What can you add to that?

**R3: I was told that blood pressure comes from the family but I don’t know if that is true but I know that my mother died at 81 years of blood pressure but I don’t know if it is because I was born of a woman who had this condition. Some say that it is hereditary coz if it is controlling them I don’t use a lot of salt, I don’t use sugar, I don’t use oil. I am really trying all means but my blood pressure is still not controlled. I thought that I will be healed but it is not getting getter**

**Mod:** Ask your health provider that question the next time you go for your clinic

**R3: I asked but these doctors from public hospitals don’t answer some questions. The just take their pens to write when you try to ask the questions. They just tell us to continue taking drugs and if you tell them that you are tired of taking drugs then they add you other drugs**

**Mod:** Tell me about your health care providers’ factors

**R3: This condition is not getting better; he can even see that because he cannot there is no change from the time he checked last month and the current readings. Tell me the problem please because even if it is chest problems then there are places that people go to be treated. Why it that this condition is never treated? That is the question that I always ask myself. Can you use medicine for 5 years on a condition that is not getting treated?**

**Mod:** You want to tell me that your health care providers don’t give you adequate information?

**R3: They only tell us not to use a lot of salt, to take drugs on time. That’s the story. If you think of stopping using drugs when the condition is controlled the blood pressure becomes worse again after 3 days. You will find it reading at 190/100. Those drugs are just pain killers, they don’t treat anything**

**Mod:** What can we do about the government?

**R3: The government said the other day that hypertension patients should take care of themselves because stress is the reason behind this condition. They always tell us to avoid stress. They only consider diabetic patients but for us they just tell us to avoid stress and we should stop eating without doing exercise. That’s the answer. We are really trying to do that**

**Mod:** R4, we are still following the same order. What do you think that you are not doing as an individual that is hindering you from managing your blood pressure?

**R3: As an individual I am supposed to do exercise but I don’t do because of congestion. There is no space to do exercise at the place where I stay especially with this corona and even if it is going to the gym them you have to pay so I don’t do exercise. Secondly I don’t eat way I am supposed to eat at home. I have both big and small kids in high school and in primary school. They are big kids so when we talk sometimes I have to be calm so that we can relate well. Avoiding thinking becomes hard because as we are here, I still have to provide food today. I am thinking of what to do after I leave this place yet we are told to avoid thinking**

**Mod:** You have mentioned the community, family and individual factors, tell me about your health care providers factors

**R3: He said that we are still monitoring and so I leave it at that but I realized that when I went there on Monday and my blood pressure was high, I was given drugs but my head, chest and back were aching. I went home and waited as my head was still aching and I was feeling dizzy. I just told myself that I had malaria and so I went to the chemist and bought HCN and after using that antimalarial doze then the pain elapsed. These people need to be trained to know that these hypertensive patients don’t have only hypertension alone. I could have died of malaria as they w were treating blood pressure**

**Mod:** What about government factors? Where is it weakening?

**R4: It is weak because nowadays blood pressure condition is not only affecting those that are above 60 years. Looking at OVC, people that are more than 60 years do get money, there is an uwezo fund loan for the youths but those of us who are between 35 years to 40 have been left yet we are the ones who have a heavy burden. We don’t have access to any loans or supports. The government has left us yet we are the ones that have the burden and it is helping those that don’t have any burden but we are not complaining because god is helping us. Third point is that the government is taking care of those patients that have HIV. There was a time that I was affected by HIV because one person in my family died of HIV but luckily it was a discordant couple. The processes that I went through with the kid that I was left with though he died in the year 2010. If there was a mechanism laid for high blood pressure patients same as the one that has been laid for HIV patients then it would be better because any one that has blood pressure is someone who is productive because he thinks a lot and he is creative. You can’t just sleep and get this condition. These people who have high blood pressure must be engaging their mind a lot and if they are boosted a little then they can do better**

**Mod:** R5, in the same order like the others. Tell me what you are not doing as an individual that hinders you from managing your blood pressure

**R5: Avoiding thinking**

**Mod:** What else are you not doing as an individual?

**R5: I don’t get you**

**Mod:** What is it that you can do to manage your blood pressure?

**R5: I said that I should avoid thinking a lot**

**Mod:** Is there any other thing?

**R5: No**

**Mod:** What are the family factors that hinder you from managing your blood pressure?

**R5: For the family, you should always agree on what to do in case you stay with another person. You shouldn’t be disagreeing. If we agreed to do something and you change and do it the other way then that will bring up issues and that can lead to a rise in blood pressure**

**Mod:** Ok. What about your health care provider? What is it that they are not doing and you feel that your blood pressure would be normal if they did?

**R5: It’s about the drugs. When you go to the hospital and you don’t find drugs then they tell you to get the drugs from the chemist where it is vet expensive and you can’t afford them so you just come back home with your condition and buy the drugs that you can afford at a shop because you don’t have enough money**

**Mod:** What about the government? What do you think that they can do?

**R5: It would be better if they supplied the drugs so that we can get them at the hospital when they are prescribed instead of us going to buy them outside**

**Mod:** Back to R1. What do you think we can do to solve this? What can you do to solve your individual problems that you had mentioned?

**R1: Personally I think that for us to manage this blood pressure condition, then you as partners from the NGO should see what you can do just like the other person said. You can source some money for us to do business because while doing business then you will not have to think a lot on what to eat or what to do with your kids for example we have parents who have kids at school and these kids need school fees that he can afford because he does not have a good job. As an organization you can look at that situation and see how hypertensive patients can be assisted to starts small businesses to occupy their mind so that they are not idle to think what they can do or what their kids can eat. When you have a business you will always be thinking of going to the market to buy things that you will sell. Thinking will be reduced and this will help us manage the blood pressure**

**Mod:** What about your health care provider, what can he do differently?

**R1: There is nothing that he can do differently because he is just like me. He also complains just like me when I take my complains to him so there is no way he can help me but if there is something that you can do to help us with our condition then please do so that we can also feel like the other people**

**Mod: What about the government? Everyone has told me about their problems. You told me about how they are supposed to stock drugs and you also said that you are ok with the time that you take at the hospital as you wait to be treated**

**R1: Yes**

**Mod:** What do you think that they can do differently for your blood pressure to be normal?

**R1: I leave the government out of this because we were writing names of people who were to get food from the government but they have never gotten anything from the time Corona was mentioned in the country. We were told to write their names so that they can be given food and 1000 shillings on every week but they have never gotten anything. I was told that people from the other villages have gotten the money but not all of them, only 100 people out of 300. I prefer leaving the government out of this so that we can deal with you as an NGO because you can listen to us and you can attend to our problems as first as possible.**

**Mod:** Ok. R2. What do you think that you can do differently as an individual?

**R2: I can say just like my brother said. Just remember us because you have eyes that can see ahead of us. Just feel mercy and help us so that we can feel like the other patients. It would be better because we are not physically challenged. We can be happy if we can have our small businesses so that we can get money that will enable us buy drugs, food and help our kids or even pay rent. We would really be happy if you did that.**

**Mod:** What do you think that your health care provider could do?

**R2: I don’t see anything wrong with my health care provider because he is just employed and he helps where he can**

**Mod:** Government?

**R1: They should have mercy and remember us BP patients just like they remember the other patients. They should know that we are also patients**

**Mod:** R3, tell me what you can do differently as an individual to manage your blood pressure

**R3: Most blood pressure patients don’t think that blood pressure hurts. The problem comes when he gets angry and thinks a lot on what to eat in the future or how will they eat, when i think of my children who are not working and they are disturbing me, I don’t have a place to do business and I don’t have capital, I just have to hawk around. Just like that lady said, having a place where you can be selling your things will help you reduce your thinking because thinking contributes a lot. When I am busy I don’t even think that I am having blood pressure because I’ll be happy**

**Mod:** What about your health care provider? You told me that they don’t give you appropriate information. What can they do differently?

**R3: I remember of one health care provider who used to take alcohol. Such a person can understand your problems when you tell him. There those care providers that take alcohol and I know of one who used to come to work while drunk. You go to him with your blood pressure condition and maybe for him he is using alcohol to control his blood pressure then you meet**

**R4: You can’t help each other**

**Mod:** Which facility was this?

**R3: It’s just here in Nairobi. Did not go to the facility that he referred me to go because I knew I will be told to pay yet I didn’t have money. Most of them are not scared of anything because they are not supervised and they are used to treating people that way and they come across many cases. They just ask you about your condition and advice you to continue taking drugs. You can’t believe in them so you just go home and pray to your God but if you had your business then you will choose to go to private hospital and not go to public facilities because you will be treated in the right manner. Those people that have money don’t have blood pressure problems coz they are treated well but for us it’s a problem because we don’t have money. It is not easy to treat a condition that affects the heart. We can be happy if we could be having something to depend on but moving here and there is a problem**

**Mod: What about the government?**

**R3: I said that reaching the government is a problem because it is the same government that is telling people to avoid thinking a lot and take a well-balanced diet. I remember that there was an old lady who had blood pressure condition and she decided not to go to a public hospital and she started going to the chemist like this person. Am sure you also saw that they are not helping you. Is not that she likes going to the chemist. If she is told that people are getting treated then she will go back to the public hospital. When you have money you go to private hospital because you will get drugs and they will do the required checkup on you to know the problem**

**Mod:** R4, what would you differently as an individual to manage your blood pressure

**R4: If I was able then I would love to do a job that I will be able to do while am relaxed and not running up and down on the road then this will really help me. If I get a place where I will be busy them my blood pressure will go down secondly there are those things that I am trying to do to myself like taking water. I have already taken 4 glasses of water as we are sitting here and that really helps me a lot. I can do that as an individual**

**Mod:** What about your health care provider?

**R4: My health care provider has a problem because with his level of education, job and stress, just like my friend said, he has his own problems like me and if I look at him it’s like he also needs help**

**Mod:** What about the government?

**R4: The government is the main reason to why everybody is hungry like for example when COVID 19 came. I f I had over 50k in my account then my blood pressure would be normal. With 50,000 in your account you are secured. They started writing names when COVID came and by the way we have not written our names. There are some things that make the blood pressure to be high but its only God who helps us. You go to the road and you hear a person saying that he has received 7000 shillings, he has received 3000 shillings, I received 2000 and again I received 1000. This is the same person and you stay in the same village but for you have never received anything. Which criteria do they use? They said that NGO can be used but even NGOs have problems with getting in the slum. There is a problem because those that write names use people that area in the slums and those people that are in the slum know the people that they write. I wouldn’t want to go dipper into that but we do suffer alone because the NGO use people who write the same people or other people from Dandora, mile saba or anywhwere else then people in Korogocho remain in the same state. I don’t know how we can involve the government**

**Mod: R5, tell me what you can do differently as an individual**

**R5: If you are busy hen you will not have a lot of things in mind and by that blood pressure will not disturb you**

**Mod: What about your health care provider?**

R5: What do I tell my health care provider?

**Mod:** Let us leave that, what about the government? I know you can’t talk about the health care provider because you don’t attend clinic

**R5: Yes, I have never gone elsewhere**

**Mod:** What can the government do differently?

**R5: The government should take care of people. Just the other day I saw people writing names in the slum and the people who gave their names were later on given rice but I was not around. I tried following up on that but I did not get anything. Got angry and decided to leave that**

**Mod:** You got angry and decided to leave that?

**R5: Now if they tell you that they will bring to you and you follow up and you don’t get it, I was told to go and wait in the house. If my name was written down then where did my package go? I just decided to leave that**

**Mod:** Back to R, we just a have one question then we finish. How has this COVID situation affected how you get hypertension care in this community?

**R1: this COVID situation has affected us so much because most of the work is not being done. Sometimes when you go to the hospital you find it is like they are not working because they don’t want people to crowd there so you just have to commit yourself as an individual to know how you will get drugs. It has affected us in many ways because the hospitals don’t attend to people the way they are supposed to be attended to. It has really affected us as community members**

**Mod:** R2. How has this COVID situation affected how you get hypertension care in this community?

R2: It has affected me because even this masks that we are using to cover our mouths are a problem. It really hard for me to breath when I am wearing them and other times there is a lot of heat, you are breathing faster and d you are going for clinic. When you reach there you are told not to move closer to the person attending to you. You just put on your mask but there is a very big problem. That’s one and the other problem is income. If you had someone who as helping you then the ways are blocked and you can’t get money from where you used to get. That is a problem and it becomes a big burden

**Mod:** R3. How has this COVID situation affected how you get hypertension care in this community?

**R3: I can say that Corona has caused us problems like for us we used to hawk vegetables to people’s houses but you can go there now because they have closed their houses. I remember there was one who told me to leave and she will call me when Corona is gone. I used to supply her with vegetables. He was peeping at the gate and I could only see the mask. If you decide to go to your rural home you will be surprise that even your friends will not get close to you because they think that you came with Corona from Nairobi and they should keep distance. It has affected friendship. People don’t relate the way they used to relate before. You can’t go to places where people are crowded and you cannot visit people. You just move to get vegetable and flour for you to cook and sleep. You cany just walk around**

**Mod:** R4. How has this COVID situation affected how you get hypertension care in this community?

**R4: It has really affected a lot because if you go to the hospitals you will find that the new arrangements are consuming a lot of time because of the spacing and again there time for people to be taught on what they should do and what they shouldn’t do secondly COVID led to closure of schools and so everybody is at home to disturb you so COVID had affected almost all areas. I don’t know if there is any sector that has not been affected. You go to the hospital then someone comes and tells you to stand up because they are fumigating. You get annoyed and leave the facility because you know that you will not receive treatment on that day. COVID has really caused a lot of effects**

**Mod:** R5. How has this COVID situation affected how you get hypertension care in this community?

**R5: Corona has really created division among people. You find you close friend who you can’t leave behind and you are forced to. It again led to loss of jobs. All the offices are closed and if you go to any office you are told to wait till that time when corona is completely gone. We can do many things but because of corona then it becomes hard**

**Mod:** R1. The last question, what do you think that you did not mention about blood pressure and you feel like you would want to talk about?

**R1: I would like to request for training on where this condition came from and what causes blood pressure so that we can be able to train our kids at home and tell them that these are the factors that lead to high blood pressure and it is good if they avoid this for them not to be hypertensive. I was asking myself what leads to high blood pressure. Is it coming from bathing or taking dirty foods or water? You can’t tell because every time you go to the hospital you are told that your blood pressure is high and you don’t know what to do. I would say that it will be good if we get researches that can get the real cause of this problem so that we can understand because we don’t understand. If you ask us what is hypertension we will just tell you that it is when your blood pressure is high and that’s all. We don’t understand what it is. That’s all I can say. We just need to know what causes blood pressure**

**Mod:** R2, what do you think that you did not mention about blood pressure and you feel like you would want to talk about?

**R2: I would like you to help me if you can, like for me my mother had blood pressure and later on she developed diabetes then stroke before she died. My father also died the other day. He had blood pressure then he developed diabetes then he died of cancer. Will I also go through the same or how will it be for my case**

**Mod:** People have different bodies

**R2: I fail to understand because it has killed two people**

**Mod:** People have different bodies and we cannot say that it will be that way

**R2: I really don’t understand**

**Mod:** We just pray because everybody has a different body, so I can tell you this then it turns out differently so it would be better if you pray for yourself and if you have other questions that you feel that I have not answered then it will be better if you ask your care provider because he is the one that has been following you from the start, he knows when you started taking drugs and he has your measurements from such a date to such a date. If I tell you and we just met today and I will get to know your today’s blood pressure so you will not be able to tell me how it was from the start. You understand me?

**R2: Yes**

**Mod:** So if you go to that person who has been following up on you and tell him that you have such a question and it is about this or that. It will be easy for him to tell you because he has been following up on you because he knows your blood pressure and he will advise you on what to do. It would be better if you asked him

**R2: Ok**

**Mod:** R3**.** What do you think that you did not mention about blood pressure and you feel like you would want to talk about?

**R3: I can say that pressure has killed many of people. They just fall when walking on the road. You might have left your house going for a journey then you just fall on your way. When you fall down you are either taken to the hospital or you just die there. There are only two options. It has really killed many even in houses. There was one who was taking a bath the other day and he just fell down in the bathroom ad died because of blood pressure. From the time I was diagnosed in 2001, I only knew because I had another condition and when I saw that the doctor had prescribed HTCZ for me and when I knew more about pressure is when I realized that I was given that drug because I had blood pressure**

**Mod:** So you were not told when you were given drugs for the first time

**R4: Yes. I was not told then I came to realize that I have been having this condition for a long period. There are many people who have it but they don’t know but for us that have pressure and God is still protecting us, we need to be very thankful. You can die of any disease but this one kills people very fast but for us God has always protected and what I can add is that I ask God to continue blessing us so that these drugs that we are taking can heal us. That’s all I can add in regards to blood pressure**

**Mod:** R4. What do you think that you did not mention about blood pressure and you feel like you would want to talk about?

**R4: Pressure is a monster and as I said earlier, pressure is worse than AIDs. With AIDs you can stay for long if you are taught about it and on how to use your drugs. With pressure you can just leave your house just like this lady said and when it gets high you just fall down. It is bad but if there is an environment in which it can be contained that one is able to manage himself well in the morning then one can live for long but when you have stress or other issues on the road like those people who are hit by vehicles on the road, those that cause accidents on the road do all those because of pressure. Pressure is a condition that needs attention more than the one given to AIDs secondly like that lady who said that her dad had blood pressure and diabetes and her mother also had stroke, such things can happen just like I told you that I started using antihypertensive the other day and I have never used diabetes drugs. I use a lot of water because I fear those drugs because drugs have effects and they can either cause cancer. They just give people drugs but they don’t teach them the effects of the drugs that they are taking and how one can avoid the effects that come with the drugs that they are using. The doctor just tells you to used rugs but how do you dilute them? How do you neutralize those chemicals that you are taking? There are no such teachings to blood pressure patients and that is another monster. You can be treating blood pressure then you get cancer or stroke. I said at first that those caregivers need extra training. Ass APHRC I normally tell those that come to my house I always tell them how the slum is because I know that you do take that report somewhere just as I read here and it can be used elsewhere so I can say the health providers need extra training. He shouldn’t just work but create time to advance and know the effects of drugs because the other day I was given enarapryl and if I look at the drugs and I look at the person giving me I can agree that I am supposed to use it because if you are using a drug that may lead to kidney failure yet you have blood pressure. That makes the problem worse. If there is what can be done to control kidney failure then there is need to study a lot.There is a question that I would want you to ask because for me I don’t know. She said that they have a group, what’s the purpose of the group are they taught or is it a support group?**

**R3: That group was meant to help people plan on how they can be buying drugs. Doctors were part of the group for them to be buying for us drugs. We gave out money like 200 bob each. There was a group that was at {Name of a facility} and it really helped us because they used to give us drugs and everybody was giving 100 bob. We were not missing drugs and we were paying 100 bob only**

**Mod:** So you use to come up as a group at provide, give 100 bob then you get the drugs

**R3: Yes for every condition be it diabetes or what**

**Mod:** So you were not missing drugs then

**R3: Yes**

**Mod:** Are you answered?

**R4: Wait**

**R3: if it was possible that the patients came together at this public hospital, just that it is not easy to bring people together because there those that refused but they would have formed a good Chama**

**R4: did that group help you? Let’s start from there because that when I realized that I have blood pressure**

**R3: Yes, it helped us**

**R4: The problem that was there was that they looked for people because personally my blood pressure was checked at my door and when I was diagnosed positive that’s when I was referred to Provide. There other who didn’t know that they had blood pressure but they were told to go there. I didn’t opt to take drugs though there is a lady that asked me and I told her that I will try something else because I used to ride a bicycle at that time. They introduced people to drugs because they could get them for free the they started telling people that we will stop giving you drugs because you are paying the money and it became pressure on top of pressure because they are supposed to pay and they don’t have money. Many people died**

**R3: It’s true**

**R4: There was an NGO that was supporting that and when it collapsed then many people died because they were now supposed to pay for them to get drugs. I was trying to say that one should not start using drugs then again stop because that is your life. I know of many people that I found there and they are not alive. Some were not very old and personally I did not start taking drugs then. That is my own research. I just researched on myself and I found them being told that they will not be getting drugs because you did not pay last month and the other month. You understand?**

**Mod:** Ok. R5. What do you think that you did not mention about blood pressure and you feel like you would want to talk about?

**R5: I can say that blood pressure is a bad condition and it’s only the doctor who can help me by telling me the cause of that condition. You go for a test then you are told that you have hypertension and what I know with hypertension is just rise in blood pressure. You don’t know what’s next after knowing that you have high blood pressure. You can be given drugs and there are others who don’t even use those drugs because they are scared. You force them to take the drugs while at the hospital and you direct them on how to use them at home but when they reach home they don’t use them and so there is no day that their blood pressure will be normal,. It will always be rising because they don’t use drugs and when they are asked they say that they took all the drugs and they finished them. I would like the doctors to tell us where this blood pressure condition comes from, what are the causes and by that we will be able to know if we do this then we will get high blood pressure condition. Taking a lot of drugs become toxic in the body which might even cause other diseases and these diseases causes other things**

**Mod:** We are done, thank you for your time and I think that all that you have told me have been captured in our recorder and I have understood. I will note down all that you have told me and the information is confidential and we will ensure that this information reaches those that are supposed to know this so that they can change what should be changed and better the ones that are not ok. Thank you for your time and I wish you well as you go home

**…END…**
