# Supplementary material for: A socio-ecological framework examination of drivers of blood pressure control among patients with comorbidities and on treatment in two Nairobi slums; a qualitative study
Source: PLOS Glob Public Health. 2023 Mar 10;3(3):e0001625. doi: 10.1371/journal.pgph.0001625 (PMC10021823; doi:10.1371/journal.pgph.0001625)
Supplement: S1 File — (ZIP) [file pgph.0001625.s001.zip › Community/KOCH-IDI-UHTN-200710-0606.docx]

**Moderator**

**Code: KOCH-IDI-UHTN-200710-0606**

**Moderator:** You confirm that I have read to you and you have understood the information that I have read to you. You have been given an opportunity to consider the information, ask questions and tour questions have been answered satisfactorily

**Respondent: Ok**

**Moderator:** You understand that you participation is voluntary and you are free to withdraw at any time without giving any reasons without any of your legal rights being affected

**Respondent: If I leave where?**

**Moderator:** You understand that the data collected during this study maybe looked at by individuals where it is relevant to your taking part in this study. You give permission for these individuals to access your data

**Respondent: Yes**

**Moderator:** You confirm consenting to be audio recorded and you also consent to the use on anonymized verbatim quotations

**Respondent: Yes**

**Moderator:** You are happy for your data to be used in future research?

**Respondent: Yes**

**Moderator:** This community has been identified to have a high burden of uncontrolled hypertension which is a leading factor to premature deaths and disability. I am trying to gather information about hypertension care in your community. To avoid hypertension related complications, it is recommended that people with high blood pressure can change their lifestyles in regards to diet, physical activities, smoking, alcohol consumption and using blood pressure medication**.** So tell me about your experience with having high blood pressure

**Respondent: I knew after my mother fell sick and died. That’s when I became sick because I had used a lot of money to treat her. I started having many thoughts and I think that’s what led to that. So my question is, how can you help me?**

**Moderator:** Let us continue answering questions for now then we will know where the problem is

**Respondent: ok**

**Moderator:** How long have you had this high blood pressure?

**Respondent: For like 4 or 5 years**

**Moderator:** 4 years?

**Respondent: Yes 4**

**Moderator:** How often do you check your blood pressure?

**Respondent: I checked after 2 years after my mother’s death. That’s when I found that I had pressure**

**Moderator:** For now, how long do you take for you to check your blood pressure?

**Respondent: I just take some time then I go to taste and when I go there I am always told that the pressure is still high and when I take drugs the pressure comes back to normal. I don’t follow up on my blood pressure frequently**

**Moderator:** Where do you go to check your blood pressure?

**Respondent: At the hospital**

**Moderator:** Do you record your blood pressure measurements?

**Respondent: No, I don’t record**

**Moderator:** And how do you know if your blood pressure is high or low if at all there is no place that you record your measurements?

**Respondent:** I go for checkup when I just feel like my body is not normal

**Moderator:** Do you have any other condition apart from high blood pressure?

**Respondent: No, I don’t have another condition**

**Moderator:** What have you been told is your target blood pressure by your health provider?

**Respondent: I didn’t ask, I was just told that it is high and that’s all I know and I just take medicine and when I feel that it is ok then I stop**

**Moderator:** Tell me about the drugs that you are taking

**Respondent: I just call them a brown one and the white one**

**Moderator:** Its ok, it’s not a must you mention the names. I just want to know if the drugs that you take

**Respondent: maybe I check and ready or somebody reads the name for me because I don’t know how to read**

**Moderator:** Have you been added other tablets since you started taking medication like you said four years ago? Has the doctor been adding you the number of drugs or he has been reducing

**Respondent: I only go to the hospital when my blood pressure is high**

**Moderator:** Where do you go?

**Respondent: {Name of the facility}**

**Moderator:** Have you been added more tablets or have they been reduced in number from when you were diagnosed with high blood pressure?

**Respondent: When I take drugs my pressure comes back to normal**

**Moderator:** How many tablets were you given at first?

**Respondent: I didn’t as but I was told that the pressure is high**

**Moderator:** How has high blood pressure affected your life?

**Respondent: sometimes when its high I can’t walk for long, I can’t do some other things that’s when I go take drugs for it to come down at least**

**Moderator:** Apart from taking drugs, what else do you do to control your blood pressure?

**Respondent: I sew sacks at {Name of a place} and I always think a lot when I don’t have money and my blood pressure becomes high when I think a lot. Sometimes our sales of sacks go down and I have to think of what the kids will eat or any other work that I can do**

**Moderator:** What of dieting and doing exercise?

**Respondent: you know when you don’t sell and you don’t have what to eat then you have to think a lot**

**Moderator:** Who do you see when you go to the facility? Who attends to you?

**Respondent: I don’t know their names. I just go to the hospital**

**Moderator:** What did the last person that attend to you tell you?

**Respondent: I don’t know their names but I normally see women and some men there**

**Moderator:** What can you tell me about your health care provider on how he is managing your blood pressure?

**Respondent: He does it well, he normally ask us questions then he tell us to take medicine**

**Moderator:** Have you gone to any other facility to seek health care apart from this facility?

**Respondent: I went to {Name of the facility}**

**Moderator:** What did they tell you about your blood pressure?

**Respondent: I was told that my blood pressure was high and they advised me to continue taking drugs and I continue attending clinic**

**Moderator:** So you told me that sometimes you go to {this facility} and other times you go to {this facility}?

**Respondent: Yes**

**Moderator:** What made you start going to {Name of the facility} yet you were going to {this facility} initially?

**Respondent: I didn’t have money to go to {Name of the facility} and I didn’t know what to do**

**Moderator:** So where do you go nowadays

**Respondent: I have not gone anywhere. The ones that am using I bought from a shop**

**Moderator:** When did you taste last?

**Respondent: It’s been a while since i took my last measurement**

**Moderator:** Which service did you get the last time you went to the hospital?

**Respondent: I went to a shop; I didn’t go to the hospital**

**Moderator:** How did they attend to you at the shop that you went to? What were you told?

**Respondent: I was told that my pressure was high and I should continue taking my medicine frequently. I used to take drugs and sometimes I don’t. I was told not to stop but continue taking drugs**

**Moderator:** You had told me that you used to pay at the facility

**Respondent: No, at {name of the faculty} is where we used to pay. At {Name of the facility} we don’t pay**

**Moderator:** So you don’t pay at {Name of the facility} but at {Name of the facility} you pay

**Respondent: Yes, when I used to go there we were not paying**

**Moderator:** Ok, so how were you getting drugs and health care service at {Name of the facility}?

**Respondent: They used to take our blood pressure measurements then they direct us where we were supposed take medicine and we were told when we were supposed to come for the next clinic. I stopped attending clinic so that I could monitor how am fairing on and that is when I started feeling pain in my legs and decided to go back but I have not gone to the hospital. I decided to buy drugs from a chemist shop**

**Moderator:** Do you have any difficulties in managing our blood pressure? You had told me that sometimes you lack the money required to buy these drugs

**Respondent: Yeah, sometimes I don’t have money when our sales are down. Sometime we even go to bed without a meal and you can’t find anything to do**

**Moderator:** What are your own individual factors that make you not be able to control your blood pressure?

**Respondent: You know I usually feel some pain when I take those drugs and that why I decide to stop for sometimes. I normally feel some stomach pains and you need to eat well when taking those drugs and sometimes I don’t eat well and that why I stop**

**Moderator:** What of the costs, do you use any insurance

**Respondent: No, I don’t have**

**Moderator:** What are the community or family factors that can make you not to be able to control your blood pressure?

**Respondent: It’s just lack of money. When you have money that when you can control because you can buy all that you are required of and when you lack money that when you think and the pressure goes up**

**Moderator:** What are the doctors at the health facility doing that makes you unable to control your blood pressure?

**Respondent: Because of the long queues, you can wait for long and even decide not to attend the next clinic**

**Moderator:** What can you say about the way they treat you or their health care services?

**Respondent: They are ok but sometimes there are no drugs and we are told to go buy**

**Moderator:** Do they give you any information concerning blood pressure?

**Respondent: No they don’t**

**Moderator:** And do you miss drugs and find long queues in the facility that you attend? Tell me about the challenges you face at the hospital

**Respondent: Sometimes we miss drugs or we find long queues that make me decide not to go to the hospital again**

**Moderator:** What is the government doing that makes you not able to control your blood pressure?

**Respondent: In Korogocho it’s like we don’t have a public facility, its only one and its like it is never stocked with drugs. It’s at the chief’s camp but the problem is that there are no drugs there and when you go there you are told to go buy**

**Moderator:** How can we solve the problems you mentioned? Like how do we solve these individual problems like you mentioned that sometimes you don’t have money or you have not eaten well and so you cannot take medicine and you also said that sometimes there are no drugs in the hospital? So what do you think can be done to stop this problem?

**Respondent: I don’t know what I can do, if its business that I will do or what. For my business maybe I change my business but I don’t have money to change. If I had money I would change my business and start another one so that I can at least get what to eat**

**Moderator:** You said that sometime at the hospital you miss drugs, what can they do for you to get better services?

**Respondent: I don’t know if the problem is the large number of people at the hospital or what could be the problem**

**Moderator:** What do you think can be done to solve the large numbers issue?

**Respondent: I don’t know why there are large numbers there coz everybody has his or her own needs. The government can be checking what’s happening at the facilities. They might be selling the drugs or something of that sort**

**Moderator:** You have shared a lot and we are about to finish. How has CORONA affected hypertension service delivery in the community?

**Respondent: For these CORONA, I am told that there is food that is donated to the community but we don’t get. There are many services that come but we don’t get. Like me I have never received anything and I don’t know where all this is done**

**Moderator:** On to the last question, is there anything else that you fell we were supposed to talk about hypertension and we have not mentioned?

**Respondent: I am asking to know how you can help us based on the questions that you are asking us**

**Moderator:** I can’t get you clearly, kindly repeat

**Respondent: How will you follow up to assist us?**

**Moderator:** Based on your blood pressure problem, we called you because we got your number in our list that we took sometimes back and we did research where we took blood samples and did everything. I was given your number from that list and I called you to inform you about this research that we are doing and we wanted to know why your blood pressure has not been controlled yet you are on drugs though sometimes you say that you are not on drugs. We wanted to know why your blood pressure is not controlled from the last time that you were tested and why it is not ok

**Respondent:** Sometimes the blood pressure goes down. When i get food or drugs well then the pressure goes back to normal but when I think a lot that when the pressure goes up again

**Moderator: Ok**

**Respondent: Sometimes its high, sometimes it’s low so you can’t tell the exact problem**

**Moderator:** That’s why I asked those questions for us to know where the problem is and how the problems can be sorted. So that we can know if the problem is the drugs or if the problem lies on the patients or even the government and see how we can help you

**Respondent: So the problem is food, money,**

**Moderator:** So that’s what we wanted. After collecting all this information from you then we will know how to solve.

**Respondent: Ok, for us we are just in Korogocho**

**Moderator:** Ok, thank you so much for the information that you have given me and the time that you have taken to share this information concerning your blood pressure and your way of life and I think from the information you have given me we can get some other information that can be combined to know where exactly the problem is

**Respondent: Ok**

**Moderator:** Thank you

**…END…**
