# Supplementary material for: A socio-ecological framework examination of drivers of blood pressure control among patients with comorbidities and on treatment in two Nairobi slums; a qualitative study
Source: PLOS Glob Public Health. 2023 Mar 10;3(3):e0001625. doi: 10.1371/journal.pgph.0001625 (PMC10021823; doi:10.1371/journal.pgph.0001625)
Supplement: S1 File — (ZIP) [file pgph.0001625.s001.zip › Community/KOCH-IDI-UHTN-200711_001.docx]

**Moderator:{Name}**

**Code: KOCH-IDI-UHTN-200711_001**

**Moderator:** This community has been identified to have a high burden of uncontrolled hypertension which is a leading factor to premature deaths and disability. I am trying to gather information about hypertension care in your community. To avoid hypertension related complications, it is recommended that people with high blood pressure can change their lifestyles in regards to diet, physical activities, smoking, alcohol consumption and using blood pressure medication**.** So tell me about your experience with having high blood pressure

**Respondent: It causes headache or maybe if you walk for long you feel like you have a hot chest and sometimes it causes some pain in the legs making them to swell. Just some pain in the organs and the back**

**Moderator:** For how long have you been having high blood pressure?

**Respondent: I have stayed with it for like 7 years there**

**Moderator:** How often do you check your blood pressure?

**Respondent: I go for checkup after every month**

**Moderator:** Where do you check your pressure?

**Respondent: At the hospital**

**Moderator:** What’s the name of the hospital?

**Respondent: I can go to either {Name of the facility} or the Health Center**

**Moderator:** Do you record your blood pressure measurements?

**Respondent: Yeah, they record**

**Moderator:** Have you ever been told your target blood pressure by your health provider?

**Respondent: Can I remember what they record? I cannot remember the way they record**

**Moderator:** your health provider can tell you that this level of pressure is ok, has he ever told you?

**Respondent: I was told sometimes back but I forgot**

**Moderator: Ok, do you have any other conditions apart from pressure?**

**Respondent: I only have high blood pressure, I am not diabetic**

**Moderator:** Are you using drugs

**Respondent: Yes, I use**

**Moderator:** Which type of drugs are you using?

**Respondent: It’s called element and the other one is caked nephidepene**

**Moderator:** Did you start using these drugs when you were diagnosed with pressure or?

**Respondent: There was a certain project sometimes back that was giving the drugs for free but it reached a time when we started paying**

**Moderator:** You don’t have insurance like NHIF

**Respondent: No I don’t**

**Moderator: You said that you are using the two types of drugs?**

**Respondent: Yes**

**Moderator:** Have you been using the same drugs for the past seven years when you were diagnosed?

**Respondent: Yeah**

**Moderator:** Do you understand me?

**Respondent: Yeah, it’s past like 7 years**

**Moderator:** My question is, you are using the two types of drugs. Did you start taking these drugs that time when you were diagnosed or you started later?

**Respondent: I started later**

**Moderator:** Can you elaborate, later what time exactly?

**Respondent: It was after like one year**

**Moderator:** Do we say that you have used these drugs for one year?

**Respondent: Yes**

**Moderator:** Have you been taking the same number for the last one year?

**Respondent: It depends with the measurement. They change drugs if the blood pressure is very high**

**Moderator:** How has high blood pressure affected you?

**Respondent: Affecting me life wise or?**

**Moderator:** Yes, how has it affected you personally life wise?

**Respondent: It affects my body to an extend that I don’t have strength sometimes, sometimes I get sick. The bad thing with pressure is that when you see or hear something that is shocking, you your blood pressure goes up.** **For me my experience is that I diarrhea immediately I hear any shocking news even if I wasn’t sick. That’s the challenge that I go**

**through. This condition doesn’t want you to be mad, it doesn’t want any shocking news. The body changes immediately. You can’t even understand what the problem is**

**Moderator:** Apart from medication, how else do you manage your high blood pressure?

**Respondent: I follow on how am advised in diet, I use a little salt, I don’t add salt in food, I take a lot of vegetables and fruits**

**Moderator:** Do you normally stay in the house or you do leave the house?

**Respondent: When we go for clinics we are told to be walking for long on foot and not just sit in the house, we are also advised not to be motorbikes every now and then. We are told to do exercise by walking because walking for long distance helps in lowering the blood pressure**

**Moderator:** Are you using any traditional medicine

**Respondent: No, I don’t use traditional medicine**

**Moderator:** What else do you do to control you blood pressure?

**Respondent: Avoiding stress, you just control your thinking even when you hear of anything**

**Moderator:** You told me that you normally go to the Health Center or {Name of the facility}. Who do you see when you go there? Do you see a doctor or a nurse?

**Respondent: A doctor**

**Moderator:** In your views, how is the health care provider managing your blood pressure

**Respondent: He must take measurements fast**

**Moderator:** How is he managing you?

**Respondent: Well. Even his advises are good. He advises us on how we can stay**

**Moderator:** Have you sought care elsewhere apart from the Health center and {name of the facility}?

**Respondent: No I have not. You knob with financial issues one can even lack the money required for him to go to the hospital and when you don’t have the money you just stay and God watches over you**

**Moderator:** You said you are advised and measured your blood pressure when you go there, is there any other thing that you can add on to that?

**Respondent: Just advice on how you can sustain yourself and how you can use medicine and being encouraged to walk for long**

**Moderator:** Are you given drugs for free when you go there?

**Respondent: No**

**Moderator:** At {Name of the facility}?

**Respondent: That program ended**

**Moderator:** What about the Health Centre?

**Respondent: You pay**

**Moderator:** After how long do you go for your clinic?

**Respondent: Once in a month but when drugs are available we are given drugs that can take us for a week then you go back of after 10 days**

**Moderator:** Do you have any difficulty in managing your blood pressure?

**Respondent: No**

**Moderator:** Looking at you as an individual, you told me that you normally buy your drugs

**Respondent: Yeah, but now the pressure is lack of money. You know you can’t go on the booked date if you don’t have money. You must have money for you to go to the hospital**

**Moderator:** Looking at your age, does it make it difficult for you to manage your blood pressure?

**Respondent: Age is a problem. You know when you get old that when diseases become many in your body not like when one is young. All sort of disease come when one is old, that when today you feel this way tomorrow you feel the other way**

**Moderator:** Do you take your drugs as prescribed by the doctor?

**Respondent: Yeah, I do take them**

**Moderator:** Do you use alcohol or smoke cigarette?

**Respondent: I have never used either alcohol or cigarettes even in my young age**

**Moderator:** Looking at the family side, do they have any problem in managing your blood pressure?

**Respondent: Not much**

**Moderator:** What of where you stay? The environment?

**Respondent: The environment is not very good. Where we stay in Korogocho the environment is not very good**

**Moderator:** What about food, is it available?

**Respondent: You struggle and get just a little, sometimes you miss, just like that**

**Moderator:** Does your health care provider contribute to managing of your blood pressure?

**Respondent: No, he doesn’t contribute**

**Moderator:** What’s your view on the way you are treated when you go to the health facility that you go for clinic?

**Respondent: Their work is not bad**

**Moderator:** What of the time that you go?

**Respondent: I normally go during morning hours**

**Moderator:** Does that contribute anything?

**Respondent: That one doesn’t contribute to any problem**

**Moderator:** Looking at the advice that you are given, is it ok or it contributes to any problem?

**Respondent: The advice is ok**

**Moderator:** You have told me that you normally take drugs there, are there times that you miss drugs?

**Respondent: It’s hard; maybe you can miss drugs once but not many times**

**Moderator:** What of the time that you are attended by the nurses, is it ok?

**Respondent: They start as from 9**

**Moderator: Looking at the space in the facility, is it enough?**

**Respondent: The space is enough**

**Moderator:** Have you ever been told about the guidelines that are supposed to be used on people with blood pressure?

**Respondent: They gave us**

**Moderator:** Can you elaborate a further?

**Respondent: Guidelines explains to us. You can find someone who doesn’t know how to take care of him or her or even others who doesn’t know the type of foods that they are supposed to use, like others who are eating foods that have a lot of oil. Others eat chips and we are not supposed to eat chips so the guidelines explains to him/ her on how he should stay**

**Moderator:** You just told me some barriers to controlling your blood pressure like you mentioned lack of money, what can be the solution to that?

**Respondent: It is always a challenge if you don’t get someone to help you sustain yourself either by getting money, drugs, food because you are not working, you are just sitting at home and you have other needs then you become stressed**

**Moderator:** What can be our solution to age, I know age contributes. What can we do to our age?

**Respondent: If it is possible, those people that are old can be helped life wise, When someone is assisted, he will be able to get medicine, food then their pressure will not be high because stress contribute a lot to high blood pressure. We think a lot on where to get money, what we will eat. Such things contribute**

**Moderator:** You also mentioned the condition of the place that you stay. What would be the solution?

**Respondent: It’s so hard to solve that coz if you don’t have money to enable you move to a better place with a clean environment then you will just have to stay there. Maybe if one gets someone to support him move from that place and help him or her better his or her life**

**Moderator:** What can you do to manage your blood pressure?

**Respondent: It can be managed when you have some work that you are doing because from that one will be able to get some money and with money the blood pressure will not go high because you can be able to get fruits, eat a lot of vegetables, you will be able to get what you are supposed to use**

**Moderator:** What else can your health provider do?

**Respondent: He works according to how he was trained based on drugs issues, the way he communicates**

**Moderator:** What else can be done at the place where you go for clinic?

**Respondent: Maybe the get donors to donate hypertensive and diabetes drugs for them to given help people of low social economic status**

**Moderator:** How has COVID 19 affected your hypertension health service delivery in your community?

**Respondent: It has affected a lot because we can’t get money to go to the hospital, sometimes you lack money to buy food**

**Moderator:** What else can you add in regards to high blood pressure?

**Respondent: for me I would really be happy if I can get someone to finance me get those drugs and food, change the my current way of life, the place where am staying so that I can stay at a place with some fresh air and clean environment**

**Moderator: I am happy with the discussions that we have had, I am also happy with the time that we have spent and I believe that what we have discussed will help us in our research. Thank you**

**Respondent: Remind me your name**

**…END…**
