# Supplementary material for: A socio-ecological framework examination of drivers of blood pressure control among patients with comorbidities and on treatment in two Nairobi slums; a qualitative study
Source: PLOS Glob Public Health. 2023 Mar 10;3(3):e0001625. doi: 10.1371/journal.pgph.0001625 (PMC10021823; doi:10.1371/journal.pgph.0001625)
Supplement: S1 File — (ZIP) [file pgph.0001625.s001.zip › Community/KOCH-IDI-UHTN-200711_004.docx]

**Moderator: {Name}**

**Code: KOCH-IDI-UHTN-200711_004**

**Moderator:** This community has been identified to have a high burden of uncontrolled hypertension which is a leading factor to premature deaths and disability. I am trying to gather information about hypertension care in your community. To avoid hypertension related complications, it is recommended that people with high blood pressure can change their lifestyles in regards to diet, physical activities, smoking, alcohol consumption and using blood pressure medication**.** We well start our questions, Tell me about your experience with having high blood pressure

**Respondent: This thing started in 1989, if I work very hard I feel it, when I walk I feel it and now it reached a time when it was deteriorating so I was taken to {Name of the facility} hospital and after I was taken there I was diagnosed of heart disease. This is why I was under a regulation of going to the hospital every month for some other contact. I have been going up to 1994 then I left. They gave me some medicine that I am continuing to use up to this moment. When I feel bad I just go to the chemist and buy these medicines. These medicines are of high amount and this is why I could not finish my secondary because of the work that I had from my bosses. My heart was beating very harshly and I could not even do something. At the time of studying I could feel it beating very rapidly and this is why I remained just there is school. I could not complete**

**Moderator:** Ok, you have told me that you have been having high blood pressure since 1989 and that’s around 31 years ago and right now you have a heart disease and right now you are on treatment

**Respondent: Am on treatment but am not going there, I am just using the medicine that they gave me because going there is very expensive so I was diagnosed by the doctor and told me to buy the medicine at the time my heart is deteriorating of beating, I just go to the chemist and buy the medicine when I have money and if I don’t have money sometimes I cry so much that a neighbor gets money to go buy me the drugs**

**Moderator:** Which hospital did you go to?

**Respondent: I was taken to {Name of the facility} national hospital**

**Moderator:** Do you now the treatment that you are on?

**Respondent: The treatment is just to use medicine at the time of deteriorating of heart beating**

**Moderator:** At the moment, which medicine are you taking?

**Respondent: At the moment the medicine that a I take they are two types of medicine one is called Enderol and the other one is very difficult to pronounce. These enderol tablets I cut them into two. Do half in the morning and a half at night**

**Moderator:** Do you remember the treatment that you were on when you started having hypertension condition?

**Respondent: at first when I was going to the hospital, doctors were giving me Valium, it was a yellow tablet**

**Moderator:** When did you start using the ones that you mentioned?

**Respondent: These ones even yesterday at night when you called me I am using it even now because at this time of coldness, I normally do with them**

**Moderator:** You had talked about endroll and the other one. For how long have you been using them so far?

**Respondent: Since 1989**

**Moderator:** All of them?

**Respondent: Yes, since 1989, one was this ampretinile that I mentioned I started using it in 1998 and the one that I was using before was Enderol and imipramine**

**Moderator:** Has there been change in the quantity of drugs that you were using then and now?

**Respondent: they work together because they were the ones who changed for me the medicine of today**

**Moderator:** I am asking if there is a change in the amount of drugs that you are using. Was it added or you started taking one or a half. Is it still the same since you started taking the drugs?

**Respondent: They are not the same because they are different in action because another one makes me to sleep and another one that is called enderol is used to cure the disease of the heart**

**Moderator:** Ok, how often do you go for blood pressure measurement?

**Respondent: When I go there they must see my pressure coz always it is very high**

**Moderator:** How often do you go for clinics?

**Respondent: Every month I go. It’s just nearby. I go to the dispensary here. I have a book**

**Moderator:** What was your blood pressure reading when you pressure was measured last?

**Respondent: The last time it was very dangerous. At the time I went there, I was given another medicine**

**Moderator:** Could you recall the medicine?

**Respondent: That’s a medicine that I don’t know its name and I went to the chemist to buy it, at the chemist I was given it and after taking it in the night I was about to be taken to the hospital because I was about to die. The medicine was working more rapidly. It had more power than me. I could not use it. At this moment am still having them. I just keep then. I just took them once and I have never used them again**

**Moderator:** Do you remember the value of pressure they did?

**Respondent: I cannot remember, honestly, I am out of the house at the moment, I could had read in the book that they wrote**

**Moderator:** Apart from pressure, do you have any other condition?

**Respondent: Sometimes I don’t know whether this kind of disease of heart beating is making me sometimes my left side… I can’t walk properly sometimes. Sometimes my left side is somehow, I just feel something injuring it**

**Moderator:** Ok, normally when you go to your health provider, has he told you the normal target of blood pressure?

**Respondent: The last time I went to the dispensary , I was measured and the doctor who diagnosed me, I was doubtful of him because the way he was asking me where I live, normally where do you go, which work do you do? Your place of sleeping, how many meters is it from here to your place. I asked myself, does this doctor think that am going to die soon or what**

**Moderator:** Did the doctor tell you what your normal reading should be?

**Respondent: I saw it like this doctor was surprised with me the time I got there, I have never gone there again since then because that day I was about to die. My heart was thoroughly that I could not even walk. I was looking like a mad person during that time. I swallowed eggs, I did what, everything I took and then I had the feeling stopped immediately**

**Moderator:** You told me that you go to {Name of the facility} for clinics

**Respondent: Yes, {Name of the facility} I left in 1994**

**Moderator:** I {Name of the facility} have they told you about normal level of blood pressure. Like how it should be?

**Respondent: That is why when I went there they gave me another medicine that I have just told you**

**Moderator:** When you go there, how are they? How do they treat you?

**Respondent: At {Name of the facility}?**

**Moderator:** Yes

**Respondent: My disease was surprising people there because at the time I went there they called very many students from the University of Nairobi about 20 of them learning from me. The places I was taken to if it is now I can use one million shillings but that time it was done for free**

**Moderator:** What are they doing now that they were not doing then?

**Respondent: Those doctors?**

**Moderator:** Yes

**Respondent: I cannot tell coz I have not gone there now**

**Moderator:** When was the last time you went for clinic?

**Respondent: The last time was last year when I went to clinic at Korogocho**

**Moderator:** So since then you have not gone for any clinic?

**Respondent: The reason why I don’t go is because I just feel alright when I use medicine so I said no need of going but these medicine can help me. I did wrong to go to the dispensary because I was given a wrong medicine that wanted to kill me**

**Moderator:** And when you go to {Name of the facility}, how do you think they are managing your blood pressure?

**Respondent: When I go there they look at me very properly like am their child**

**Moderator:** Apart from {Name of the facility} and Korogocho, is there any other place you have gone to seek care on hypertension?

**Respondent: When I was at {Name of the facility}, I was looked at by a doctor called Prof. Name. Have you ever heard of that name? He is the one that diagnosed me then left me to Dr. Name. I had very big people there looking at me**

**Moderator:** How has high blood pressure affected you?

**Respondent: The time when it wants to come, I just feel my heart beating in a very dirty way. It beats like this tibi tibi tibi like that and then it starts**

**Moderator:** Apart from medication, how else do you manage your blood pressure?

**Respondent: They told me not to take a lot of salt, not to take a lot of sugar, I don’t take too much of oil, I was told many things that I do not do with now**

**Moderator:** Could you let me know? Tell me I want to hear about them

**Respondent: About?**

**Moderator:** What else do you do apart from medication? You have talked about your diet. What else?

**Respondent: Some times when I feel it, you know this thing comes differently. Sometimes I just feel my body is warm and I just go to the house and take water and handkerchief. I do with that until it comes back again**

**Moderator:** What else can you use apart from diet to make your blood pressure go back to normal?

**Respondent: Just resting enough. You just rest then later on you will feel like you are faring on well but when it does that then I am forced to take medicine which is cut into two halves. I was told that when I cut, it begins work immediately and that’s why when it comes I do take this medicine called enderol and I cut it into two before I swallow it**

**Moderator:** How is your normal day?

**Respondent: when I leave my house, I just do small businesses in Korogocho, that’s my work**

**Moderator:** Do you do exercise?

**Respondent: I can’t do exercise because of that. Exercising can lead to my death. That’s why I don’t do exercise**

**Moderator:** Have you ever used traditional medicine?

**Respondent: When this thing started in 1989, that’s when my mother and my dad brought a traditional healer who extracted some blood from me and the blood was put in a basin…18:14… (Not clear). I think that made me feel better**

**Moderator:** Are you using any traditional medicine now?

**Respondent: No I don’t use any traditional medicine now but sometimes I only use traditional medicine but for malaria. I was told a lot concerning this blood pressure. The disease comes with a lot of things. They can cause chest pains, stomachache… this disease has a lot of things because when the blood fails to go around the body and I feel very sick coz the blood goes in a way where it’s not needed to and when it goes in a normal way that’s when I feel in my body that I am in a normal way**

**Moderator:** Where would you access hypertension care services in your community? You had talked about the health center. What services were you offered when you went there

**Respondent: Do you know why people like going to hospitals that charge? When you go there you just look like an ordinary person. They just look at you when you tell them anything. You can be attended to very well at the hospitals that charge but not these ones that offer free services. The last time I went here I thought that they saw like I cannot live on this earth. Just don’t know what they thought about me**

**Moderator:** Now that you said that you used to go to {Name of the facility}, were you going to {Name of the facility} because of what happened to you at the health Centre or?

**Respondent: The types of the disease that I had when I was told to go to {Name of the facility}. You know I sell small small items here, someone saw me touching my chest and asked me what was wrong and I told him that my heart was beating very hard. He told me that he will come the following day in the morning and take me to Dr. Name. That’s when we started going with him there, I was taken there by Dr. Name cousin and that’s why they really treated me well**

**Moderator:** Did you pay for drugs at the health Centre from your pocket or you paid using an insurance card

**Respondent: I was told to go buy drugs from a chemist. They just gave me prescription on a paper**

**Moderator:** You said that you don’t like their instructions, what about the instructions that you were given at {Name of the facility}

**Respondent: At {Name of the facility} is always better but I don’t go there coz going there is very expensive. That’s why we go here and there**

**Moderator:** So have you attended clinic these year or not

**Respondent: This year I have not**

**Moderator:** But your pressure is measured?

**Respondent: When I was asked to go to {Name of the facility}I was very happy because I was handled correctly and again I was told to go to another provide clinic I can’t tell where it is. They do measure even at [Name of the facility] in Korogocho. We are measured there to see our grades**

**Moderator:** How often do you go for your clinic at {Name of the facility} after you have been given an appointment?

**Respondent: I told you that I stopped going to {Name of the facility} in 1994 but when I used to go there I was going there after evert 3 months then it changed to after two months then after one month but I stopped going because I was getting better**

**Moderator:** Do you have any difficulties or barriers in managing your blood pressure?

**Respondent: I just feel myself better when am in Nairobi. I have many years and I have to go home but I ask myself if I go home, will I manage coz this thing can kill me at any time. This heart disease is a very bad disease and I just think that when I go home and I don’t get money and am there, how I will help myself and am ready to go now. I am 66 years now and I don’t know what I will do when I go to get these medicines because it’s very difficult to get them at home there**

**Moderator:** What about your family, do you think they find it difficult in helping you manage blood pressure

**Respondent: The problems are many, sometimes you may think of doing something and when the pressure rises than you just have to leave it. I am a servant in my church and when I think of visiting my church members but I cannot because when I come I just sit down and rest**

**Moderator:** What of your living environment and diet, are they creating any hindrances in managing your blood pressure?

**Respondent: I think that when am in a mad house, a house that is not built by blocks, when I sleep inside it I just feel fresh but that one that is built by blocks and has got no good ventilation then I cannot live in that house. A house should be open. I can stay in such a house in the night or day time.**

**Moderator:** Do you think that your health providers have any difficulty in managing your blood pressure?

**Respondent: I can say that when I used to go to {Name of the facility} that’s when I saw so many people who were very much eager to help me. {Name of the facility} is a very good place for providing care to people**

**Moderator:** What of the health system? As you go to {Name of the facility}, looking at the infrastructure there at the hospital, does it have any barriers in managing you blood pressure?

**Respondent: What do you mean when you talk about barriers?**

**Moderator:** You can talk about drugs, do you find them there?

**Respondent: At {Name of the facility} drugs are available and if there are no drugs they just tell you to come on a certain day and you will find them. They don’t tell you to go buy drugs like for my condition, I used to go to cardiac clinic where we were told not to think that we will just be treated for free. They used to write their charges and take them. I don’t know who used to pay for this but we were treated like grownups whenever we went there. They used to talk to us very well that we left there smiling and not thinking about our condition. These were people who were well trained on that matter**

**Moderator:** From the policy level, do you see like there is any problem in managing your blood pressure?

**Respondent: Where?**

**Moderator**: Government, do you see any hindrance from the government in managing your blood pressure?

**Respondent: They can provide care if they know. When I went to the hospital at Korogocho that is when they were supposed to take a step but they lied to me that there is a vehicle that comes and they will call me when it come but since I went there I have never been called. I just remember the drug that they gave me that almost killed me then I decided not to go there again**

**Moderator:** Have you ever been told about hypertension guidelines?

**Respondent: They have not told me anything about that, I was told just what I have told you like what I can use, how I can be, you know this condition does not one any quarrel or make noise to you anytime. Those around you should just laugh with you and treat you like a kid for you to be an important person but if you find that your kids are making noise and you also have a noisy wife, sometimes if they ask for money to buy food then that’s when you start thinking then the disease starts**

**Moderator: What do you think would be a solution to the barriers that you mentioned? You told me that you would want to go home but because of lack of money**

**Respondent: I told you that I want to go because with my age I just don’t want to continue staying in Nairobi but if I go there, how will I be getting money to buy these drugs? These medicines are the ones making me earn respect now**

**Moderator: What would be the solution to that?**

**Respondent: If at all I can get any person who can give me advice that can help me then I can thank him or her because I wanted to go but I cannot go because I use… you know this matters about heart disease that is pressure, it comes a times when you just feel very sick only to realize that your heart was not beating properly, that’s why you feel that but when you are just given that medicine and swallow then within 5 minutes you start feeling well and this is why I just think by myself that when I go home, what shall I do, am I going to stay there for only one week then I go? I am really worried about my going home but it will force me to go because it is time, time has reached for me to go**

**Moderator:** Ok, you had talked about medicine in {Name of the facility}; sometimes you get them sometimes you are told to go back another day. What do you think can be a solution for that?

**Respondent: If am at home and am told to come collect medicine here?** **I can be very proud of that because getting money for coming and going I think I can get because my life is very much important to my family**

**Moderator:** Do you have any other thing to add there?

**Respondent: No I would like also to thank you because I never knew that you could ring me yesterday but I always think that… I don’t think there are other people who can look at my life. Yesterday I was very proud of you and I thank you very much for talking to me on this kind of the disease that is affecting me all the time and I would like you as my child to give anything that can make me stay longer than this and God will help you and I will thank you very much. That’s the only thing I can say**

**Moderator:** Ok, I have only 2 questions to finish. At this time we have COVID19, how has it affected you and the hypertension care that you are receiving in your community?

**Respondent: Community how they have helped me?**

**Moderator:** Am saying that we have COVID 19 at this time, how has it affected how you get hypertension care in this community

**Respondent: Very good question and I thank you for that. In this community where I am right now, very many people are coming with papers to write names of people and at long last we don’t see anything. My name has been recorded around 8 times here in Korogocho and I have got nothing and if you listen very well, you will hear that this thing have owners yet we are struggling. We have gotten nothing here. I have kids here that I have educated to form four level but one day when this sweeping job that was brought to us by the president here in Korogocho came, the elders in Korogocho gave these chances to their people and their kids. People complained that other youths were removed from that work and they were told that they will return back to work on 15^th^ but my kids can’t get the chance. I don’t know who are being given these positions. I have kids who can help me but there is no job. That’s the problem in Korogocho**

**Moderator:** How has COVID19 affected how you get hypertension care?

**Respondent: They have not yet come here but we really seek to know our status coz we can be sitting here yet we have the disease but we don’t know. We have not seen those people here, we just hear that people have been tested, certain number of people have been found positive but in Korogocho we have never seen anything**

**Moderator:** Ok, is there anything else you would want to talk about hypertension

**Respondent: There is nothing I can say because I have told you everything but correct me if there is anything that I have told you wrong but I just keep quite because now am keeping time waiting for you coz sometime I can get something from you**

**Moderator: Thank you so much for your time and we know that whatever we have talked about will help us our research that we are doing, Thank you for your time**

**Respondent: If we can go beyond that then I think I will be very happy because that thing can help me up to my last time**

**Moderator: Thank you so much**

**…END…**
