# Supplementary material for: A socio-ecological framework examination of drivers of blood pressure control among patients with comorbidities and on treatment in two Nairobi slums; a qualitative study
Source: PLOS Glob Public Health. 2023 Mar 10;3(3):e0001625. doi: 10.1371/journal.pgph.0001625 (PMC10021823; doi:10.1371/journal.pgph.0001625)
Supplement: S1 File — (ZIP) [file pgph.0001625.s001.zip › Community/KOCH-IDI-UHTN-200712-001.docx]

**Moderator: Name**

**Code:** **KOCH-IDI-UHTN-200712-001**

**Moderator:** This community has been identified to have a high burden of uncontrolled hypertension which is a leading factor to premature deaths and disability. I am trying to gather information about hypertension care in your community. To avoid hypertension related complications, it is recommended that people with high blood pressure can change their lifestyles in regards to diet, physical activities, smoking, alcohol consumption and using blood pressure medication**.** So tell me about your experience with having high blood pressure

**Respondent: High blood pressure entails the use of thing that are supposed to be used like fruits, medicine and one to follow what the doctor advices him or her to do…Hallo**

**Moderator:** I can hear you

**Respondent: First is to follow the doctor’s instruction, take a well-balanced diet so that you can have enough blood and water in the body for you to be like the others or for you to manage your blood pressure**

**Moderator:** For how long have you been having high blood pressure?

**Respondent: This is the third year but we are taught by the doctors that we are supposed to use greens in plenty, fruits and take medicine and by that we will just be like the other people and we are just ok**

**Moderator:** How often do you go for blood pressure checkup?

**Respondent: As for now the gadgets are many, you can take the measurement from home, you can decide to go to the doctor at the health center and they will measure you. For now there is no fear, you can go as many time as you want**

**Moderator:** Let’s talk of you as an individual, where do you go to check your blood pressure and how often do you go?

**Respondent: I normally go to the health center and I do the check up after every 3 months**

**Moderator:** Do you have a place where you record your measurements?

**Respondent: No I have not recorded**

**Moderator:** Can you remember your last measurements?

**Respondent: for now I can’t remember because am far from home. I could have checked in on a paper**

**Moderator:** Do you have any other condition apart from pressure?

**Respondent: No, I just get sick of Malaria then I get well**

**Moderator:** Has your doctor told you what your target blood pressure should be?

**Respondent: Blood pressure should be, you know there is weight. You are not supposed to go beyond 100. You are supposed to be around 85 and 80 and bellow. For me I was more than one hundred and but it went down when I started to use fruits and vegetables**

**Moderator:** Which types of medicines are you using now? Can you tell me?

**Respondent: The medicine that I use is the small yellow tablet**

**Moderator:** Do you know the name?

**Respondent: I don’t understand the name but I know the tablet. Even when I go to the hospital they normally read and they know**

**Moderator:** You told me that you have had pressure for the last 3 years. Did you start using this tablet when you were diagnosed with high blood pressure?

**Respondent: I started going to the facility after I was diagnosed with hypertension**

**Moderator:** How do you take this medicine?

**Respondent: I take one tablet per day**

**Moderator:** You have been taking medicine that way since the time that you were diagnosed with pressure?

**Respondent: Yeah, it’s only one type and it’s yellow in color. You can’t use two before eating something**

**Moderator:** How has high blood pressure affected you?

**Respondent: High blood pressure requires you to have some things that you are supposed to use. You must have vegetables and fruits but sometimes you can miss fruits and vegetables and at that moment there is nothing you can do**

**Moderator:** Apart from using drugs, how else do you manage your blood pressure? You had told me that you eat vegetables, fruits in plenty and drugs, what else do you use?

**Respondent: Even water, banana, well balanced diet, pumpkin and many other things but the problem is in how to get them**

**Moderator:** What else apart from a well-balanced diet do you use to manage your blood pressure?

**Respondent: You have to go for tests**

**Moderator:** Have you ever used traditional medicine?

**Respondent: I used traditional medicine way back then and when I noticed I decided to stop using them**

**Moderator:** How is your normal day?

**Respondent: At the moment I don’t see anything**

**Moderator:** Do you leave your house or you normally stay indoors?

**Respondent: I do leave the house sometimes to go look for a job. Sometimes I find job sometimes I don’t. I do casual jobs like washing clothes but now there is a problem with this Corona. No one wants anyone to go do the washing for them**

**Moderator:** What else do you do to manage your blood pressure?

**Respondent: It’s only what I have shared. I normally do all that that I have told you. If its medicine I do take, if it’s a well-balanced diet I do eat when I find food and if it is going for checkup I do go for tests. I go for tests when I feel that my body is not ok and I go back to see the doctor in case I am asked to do so**

**Moderator:** You told me that you always go to the health center. Who do you see when you go there? Do you see a doctor or a nurse?

**Respondent: Only doctors can do that work, there is no one who can do that if he or she is a doctor**

**Moderator:** I am seeking to know if it’s a doctor or a nurse

**Respondent: Doctor**

**Moderator:** Ok, when you meet this doctor, what can you say in regards to the way your health care provider manages you?

**Respondent: I just tell the doctor what I feel when I meet him and he checks me to see how I am and after that he takes his responsibility of attending to me coz he can’t just leave me like that. If there are no medicines he directs me to go buy the drugs coz sometimes there are no drugs coz sometimes there are no drugs at the facility. Sometimes you can go and be told that there are no drugs so you have to plan yourself**

**Moderator:** What’s your view concerning the way your doctor manages you?

**Respondent: My view is that my doctor is very important because as you live in this world you have to go to see a doctor because you can’t know your condition by yourself**

**Moderator:** I was seeking your views on how your doctor attends to you. How is he doing this?

**Respondent: He attends to me and he directs me on what I am supposed to do, the way I can live, He tell me all that because he cannot just give you medicine and not direct you on what you are supposed to do**

**Moderator:** Have you ever gone elsewhere aside from the health care to check your blood pressure?

**Respondent: The other place is the chemist because when I miss drugs at the health center then I just go to the chemist. You can only go to the chemist when you have money but if you don’t have money then it becomes hard**

**Moderator:** At the health center do you pay for your drugs or they are for free?

**Respondent: We were told to buy and they were giving us the prescription but we didn’t have money. Sometime we are told to give 200 and something yet we didn’t have the money and so we decided to be taking the prescription to the chemist where you can get drugs that can take you for two or one day instead of just sitting there without taking drugs**

**Moderator:** What kind of services do you get when you go for to seek health care at Korogocho?

**Respondent: Just the normal service like hoe you are supposed to live, how you are supposed to take medicine and how you can get means on how to help yourself because you can’t just sit without doing as instructed by the doctor**

**Moderator:** Do they measure your pressure?

**Respondent:** Yeah

**Moderator:** Ok, do you have any challenges with managing your blood pressure?

**Respondent: I don’t have problem**

**Moderator:** You had told me before that sometimes you don’t get drugs to an extend that you are forced to go buy at the chemist

**Respondent: Like for now there are no drugs, I went to this facility in Kariobangi but they also didn’t have drugs. They just do the measuring and leave you. I decided that I can’t leave my body suffer when I have my own money like 20 shillings and if I buy two tablets they can help me for 2 days. One drug goes at 10 shillings**

**Moderator**: Looking at your age, do you think it’s a hindrance in managing your blood pressure?

**Respondent: No**

**Moderator:** Does the way you take drugs hinder you from managing your blood pressure?

**Respondent: No, I have no problem with that. I told you the problem would have been taking drugs without eating because the drug has strength**

**Moderator:** Ok. Do you use alcohol or cigarettes?

**Respondent: No I don’t use alcohol**

**Moderator**: Looking at your family, are they hindering you from managing your blood pressure?

**Respondent: At the family is very had, there are those who have blood pressure and there are those who don’t have**

**Moderator:** What of the diet? The way you cook your food with your family, does it pose any challenge in managing your blood pressure

**Respondent: No**

**Moderator:** What of your health care providers, for example at the health center where you go, does the doctor have any problem in managing your blood pressure?

**Respondent: No they don’t have any problem**

**Moderator:** What of the environment at the hospital?

**Respondent: The environment at the hospital is not bad because am a CHV. We have roster where I go twice in a week to clean the hospital**

**Moderator:** What of the quality of treatment?

**Respondent: I said there are times when there ae enough drugs and there are times when there are no drugs, when drugs are there I normally see them being given out to manage patients well**

**Moderator:** What of the time that you go for treatment, does it pose any challenge in managing your blood pressure?

**Respondent: We go there in the morning hours and when the doctor comes he makes his own plan. He measures us and we go home. Initially they were taking our measurements then we take tea with two mandazis then we go home after we have been measured**

**Moderator:** Is the space at the hospital enough

**Respondent: For now it is enough**

**Moderator:** When we look at the policies, have you ever been told about the guidelines?

**Respondent: No**

**Moderator:** What’s your view about the government? Do they have a challenge in managing blood pressure?

**Respondent: Yeah, they can’t miss. You know there is nothing that is 100% perfect. There must be a problem**

**Moderator:** What would be the solution to the hindrances that you have mentioned? You had told me about money where you said that there are times when you get money to buy drugs for only one day first. What would be the solution to that?

**Respondent: Drugs should be provided in plenty so that a person like me doesn’t suffer because getting that 10 shillings to buy medicine is not easy, sometimes I have to go borrow**

**Moderator:** You also mentioned that sometime there are no drugs in the hospital. What would be the solution to that?

**Respondent:** The government should bring medicine

**Moderator:** How has COVID 19 affected how you get hypertension care service in your community?

**Respondent: At the moment people are being measured but now it’s hard, people have to get what to eat and drink. This COVID19 has blocked everything. There is no way one can get money. We can’t even get the casual jobs the world is now tough**

**Moderator:** How has it been for you to receive hypertension care service in this COVID era?

**Respondent: For now I don’t even see, for now it is hard**

**Moderator:** Have you been attending clinic during this time?

**Respondent: Nowadays you can go and find that there is no one at the facility even the doctor himself**

**Moderator:** Is there anything else that you would like to mention about high blood pressure that you feel that we have not talked about?

**Respondent: Just help people because at this moment people are should get drugs and well balanced diet**

**Moderator:** Ok, thank you for your time, this will really help us in our research, I really appreciate for your time

**Respondent: Thank you too**

**Moderator:** Good day

**…END…**
