# Supplementary material for: A socio-ecological framework examination of drivers of blood pressure control among patients with comorbidities and on treatment in two Nairobi slums; a qualitative study
Source: PLOS Glob Public Health. 2023 Mar 10;3(3):e0001625. doi: 10.1371/journal.pgph.0001625 (PMC10021823; doi:10.1371/journal.pgph.0001625)
Supplement: S1 File — (ZIP) [file pgph.0001625.s001.zip › Community/KOCH-IDI-UHTN-200721_003.docx]

**Moderator: Name**

**Code: KOCH-IDI-UHTN-200721_003**

**Moderator:** This community has been identified to have a high burden of uncontrolled hypertension which is a leading factor to premature deaths and disability. I am trying to gather information about hypertension care in your community. To avoid hypertension related complications, it is recommended that people with high blood pressure can change their lifestyles in regards to diet, physical activities, smoking, alcohol consumption and using blood pressure medication. So tell me about your experience with having high blood pressure. Tell me about your experience with having high blood pressure

**Respondent: The problems that I have been having?**

**Moderator:** Just talk in general about how it has been with blood pressure

**Respondent: I have been having headache problems, it makes me weak and it also increases my heart beat**

**Moderator:** For how long have you been having high blood pressure?

**Respondent: Its 15 years since I k new that I have high blood pressure**

**Moderator:** Where do you check your blood pressure readings?

**Respondent: I used to check at provide but nowadays I go to any hospital when I feel unwell**

**Moderator:** How often do you check your blood pressure?

**Respondent: I can’t tell how often coz I used to go to provide for clinic monthly but from the when they closed I have just been checking anywhere at any time when I don’t feel well**

**Moderator:** Do you record your blood pressure measurements?

**Respondent: No**

**Moderator:** Can you remember your blood pressure readings for the last checkup you had?

**Respondent: I think it was 130 something, 135. I can’t remember**

**Moderator:** Have you been told what your normal target blood pressure reading should be?

**Respondent: No, he has not told me**

**Moderator:** Do you have any other condition apart from high blood pressure?

**Respondent: No, I don’t have another condition**

**Moderator:** Tell me about the drugs that you are using

**Respondent: For blood pressure**

**Moderator:** Yes

**Respondent: I use some yellow and sometimes I do white tablets**

**Moderator:** How do you take them?

**Respondent: When it is…4:05 (Not clear) two per day then after …4:07… (Not clear) I take one per day**

**Moderator:** How do you take the yellow tablet?

**Respondent: I used to take 2 yellow tablets per day. Sometime I am given yellow tablets other times I am given a white tablet when I go to the hospital**

**Moderator:** Which one are you using now? Both yellow and white or one?

**Respondent: It depends with the facility that I will go to or maybe a chemist**

**Moderator:** For now how many are you taking?

**Respondent: The last time I was given some pink drugs in a packet**

**Moderator:** How do you take them?

**Respondent: 1 per day**

**Moderator:** Have you been using these tablets from the time that you were diagnosed with blood pressure?

**Respondent: Not regularly. When there was a clinic at {Name of the facility} that’s when I used to take them every time and when the facility closed then I started using one when my blood pressure is high and I could stop using when my blood pressure was normal**

**Moderator:** For how long have you been using the ones that you are using now?

**Respondent: I used antihypertensive 2 weeks ago**

**Moderator:** Why are you not using now

**Respondent: You know for now I have buy and I am not ok financially**

**Moderator:** How has high blood pressure affected you?

**Respondent: I experience increase in heartbeat, my body becomes weak and my head really aches**

**Moderator:** Apart from using drugs, how else do you manage your blood pressure?

**Respondent: I was told to avoid thinking too much and incase I am annoyed then I just move from where I am annoyed and find some other place**

**Moderator:** What about your diet?

**Respondent: It’s just normal according to how someone is eats. I eat whatever I come across**

**Moderator:** What about exercise? Do you do exercise?

**Respondent: I don’t do light job, my work is hard and it don’t involve sitting**

**Moderator:** Who were you seeing when you used to go to provide?

**Respondent: There are doctors who used to come once in a week**

**Moderator:** What can you say in regards to the way your doctor has been attending to you?

**Respondent: He attended to me. Actually he is the one who told me that I am hypertensive and he advised me on what to do to manage it**

**Moderator:** Have you ever sought care elsewhere apart from provide?

**Respondent: I have gone to other facilities in cases that I have felt unwell. I have just been going to any facility that is close to me**

**Moderator:** What have you been told at the other facilities in regards to blood pressure?

**Respondent: They just give you drugs and tell you to come back when you finish drugs**

**Moderator:** What services do you receive when you go to the hospital?

**Respondent: They just check my blood pressure measurements and after doing that they give me drugs**

**Moderator:** Do you buy your drugs or you are given for free

**Respondent: I do buy**

**Moderator:** Looking at you as an individual, do you have any problem with managing your blood pressure?

**Respondent: The problem is how to get drugs**

**Moderator:** Do you have insurance?

**Respondent: Yes, I have insurance but for now it’s not active. It has not been active for more than a year now**

**Moderator:** Looking at your age, is it a hindrance in managing your blood pressure?

**Respondent: Age?**

**Moderator:** Yes

**Respondent: How, like my current age?**

**Moderator:** Yes

**Respondent: I was born in 1970. I think my age could be a problem. Another cause is the normal problems at home**

**Moderator:** What about the drugs. You told me that you took your drugs two weeks ago. Is that a problem in managing your blood pressure?

**Respondent: I don’t have a problem. I usually get back to normal when I finish my drugs. There is a doctor at the hospital who told me that I can stop taking medicine if I can control my blood pressure. He is still the one who told me that I just excuse myself if someone offends me and by doing that my blood pressure will not rise and he also told me to avoid cigarettes and alcohol**

**Moderator:** Looking at you family and the community, are they a hindrance in managing your blood pressure?

**Respondent: Yes, I have a daughter who has been disturbing me and sometimes she makes my blood pressure to rise that’s why I told you that family is the main cause**

**Moderator:** Looking at where you stay, there are these foods that are not good, do you use them?

**Respondent: What kinds of foods are you referring to?**

**Moderator:** Like chips, nyama choma

**Respondent: I used to take them way before but nowadays there is no money**

**Moderator:** Looking at your health care provider, is he causing any hindrance in managing your blood pressure?

**Respondent: My health care provider?**

**Moderator:** Yes, you had told me that there is a time that you used to go to {Name of the facility} and other times you used to go to the chemist. Was there any problem with your health care provider at {Name of the facility}?

**Respondent: No, there was no problem**

**Moderator:** How was the treatment that you were receiving there?

**Respondent: It was ok and the health care provider was friendly**

**Moderator:** What about the information and the health talks that you used to get?

**Respondent: He used to give me talks and he could give me advice**

**Moderator:** How were the clinic hours?

**Respondent: We used to go for clinics in the morning though there were many people and again it used to depend with the time when you reach at the facility because the doctor used to come at around 8:30-9:00 there. It was up to us to reach early because if one came late then he or she will find other people there and he or she will have to wait for sometime**

**Moderator:** Looking at the drugs, were you able to get them there?

**Respondent: Yes, I used to get drugs there. I was not buying**

**Moderator:** They used to give you?

**Respondent: Yes, we were not buying and that’s why we could always have drugs to take when provide was still operating and when we have to buy and we don’t have an income and the family on the other side is looking at us then we will just buy drugs when we need them**

**Moderator:** Looking at the government policies, is there any problem?

**Respondent: Government?**

**Moderator: Yes**

**Respondent: I don’t see anything that the government has helped us with because this doctor is not even under government. I don’t see anything that the government has helped me with in regards to blood pressure**

**Moderator:** What can be the solutions to the challenges that you mentioned? I will mention the ones that you have told me so that we can see how we can solve them. You told me about finances because sometimes you buy your drugs and you have not been taking drugs for two weeks now. What would be the solution to that?

**Respondent: The solution to that would be the government to provide us with drugs so that we can get drugs when we visit any hospital. They charge 50 shillings to check blood pressure and when you don’t have the 50 shillings then you will have to suffer or maybe you just go to the chemist and ask for antihypertensive. The attendant will just give you drugs and maybe pressure is not the problem or maybe you are using three different types of drugs. When you tell the attendant that you want white tablets and she tells you that she has yellow ones and you are in pain then you will just have to take the yellow one maybe it has other side effects on your body. I would request the we be given these drugs for free, leave alone me, maybe for a case of an old woman or an old man or even a kid who can’t raise 50 shillings, for me maybe I can raise 50 shillings but there is this other person who cannot raise 50 shillings. This 50 shillings is for blood pressure checkup, you need to buy drugs and again you have to pay for you to see a doctor. This condition can be controlled if we found means on how someone can get drugs and tests anytime anywhere**

**Moderator:** Looking at your age, is there a solution?

**Respondent: Age?** **Like solution to what?**

**Moderator:** We have talked about hindrances and we said that age is a factor and so we are looking at the solutions. What do you think would be the solution to age?

**Respondent: Is age a factor on pressure. You mean a solution to age?**

**Moderator:** We were talking about hindrances before and you said that your age is also contributing so we are trying to look at the solution to that

**Respondent: I can’t answer that because the problem cannot just leave at once and if at all there was a way which a problem can just leave then I don’t see how this sickness would be disturbing me but solving a problem is not easy on my side**

**Moderator:** Looking at your family side, you told me that your daughter is always disturbing you. What would be the solution to that?

**Respondent: I have tried my level best as a parent. First she dropped out of school, I tried talking to her, I took her to a college and she dropped out again, she gave birth and her son is there who is also disturbing. I don’t know what to do and so I leave it to God**

**Moderator:** We also talked about time. You said that you could take long before being treated at provide. What could be the solution to the time problem?

**Respondent: Like for me a casual worker, I have to wake up early to hustle. They need to add the number of doctors and by doing that then it will take a shorter period and we will get time for us to hustle for our selves**

**Moderator:** Looking at the government, what could be the solution?

**Respondent: I have told you on how the government can help us**

**Moderator:** Ok. How has COVID 19 affected the way you get hypertension care service in your community?

**Respondent: It has caused so many problems and when problems increase the blood pressure becomes a challenge because you have to find how you can eat yet there are no jobs and the family is also demanding. With all this then you must think and we were told that blood pressure rises because of thinking so much but again this condition has affected the whole world and we cannot blame anyone. We cannot blame nature**

**Moderator:** How has this affected the way you get health care service?

**Respondent: It is the reason as to why it has taken me long without taking drugs because there is no income and at the hospital you can only be served to the level that your many can pay. You will not be able to get enough drugs and that’s why I said that there wouldn’t be a problem if we could get these drugs for free. We would be getting the required dose to control the condition**

**Moderator:** Is there any other thing that you would want us to talk about I relation to high blood pressure?

**Respondent: I can’t think of anything else apart from that**

**Moderator:** Ok, thank you for your time and this information that you have shared will help us in our research. Thank you so much
