# Supplementary material for: A socio-ecological framework examination of drivers of blood pressure control among patients with comorbidities and on treatment in two Nairobi slums; a qualitative study
Source: PLOS Glob Public Health. 2023 Mar 10;3(3):e0001625. doi: 10.1371/journal.pgph.0001625 (PMC10021823; doi:10.1371/journal.pgph.0001625)
Supplement: S1 File — (ZIP) [file pgph.0001625.s001.zip › Community/KOCH-IDI-UHTNC-200710_0306.docx]

**Moderator: {Nmae}**

**Code:** **KOCH-IDI-UHTNC-200710_0306**

**Moderator:** This community has been identified to have a high burden of uncontrolled hypertension which is a leading factor to premature deaths and disability. I am trying to gather information about hypertension care in your community. To avoid hypertension related complications, it is recommended that people with high blood pressure can change their lifestyles in regards to diet, physical activities, smoking, alcohol consumption and using blood pressure medication**.** So tell me about your experience with having high blood pressure

**Respondent: I have both high blood pressure and diabetes**

**Moderator:** So you have both high blood pressure and diabetes?

**Respondent: Yeah, and sometimes body aches and my leg also pains so much**

**Moderator:** For how long have you been having high blood pressure?

**Respondent: For many years, almost 10 years**

**Moderator:** For 10 years

**Respondent: Yes**

**Moderator:** How often do you check your blood pressure?

**Respondent: You mean taking measurements?**

**Moderator:** Yes

**Respondent: I always take measurements when I go to the hospital**

**Moderator:** Do you have a place where you record your pressure?

**Respondent: I don’t have measuring gadget, I only take measurements when I go to see my doctor**

**Moderator:** Do they record your measurements somewhere?

**Respondent: Yeah, they record. I have a place where they record. I go to the sisters hospital**

**Moderator:** When were you measured last?

**Respondent: One day I was at my rural home. You know the measuring at my rural home is not the same as the measuring done here**

**Moderator:** How was your pressure when you were measured last

**Respondent: I can’t remember the date**

**Moderator:** You said that you have high blood pressure and diabetes and your body also aches?

**Respondent: Yes, my leg also pains**

**Moderator:** Has the doctor ever told you your target blood pressure?

**Respondent: Sometimes when I go there when my pressure is high it reads 90, sometimes 120, sometimes 80, just like that**

**Moderator:** Ok, now tell me about your high blood pressure drugs

**Respondent: How do I tell you about drugs?**

**Moderator:** Tell me about the drugs that you are using to manage your pressure and how many types of tablets are you using

**Respondent: I can’t know the names because I only take the drugs once am given. I don’t read the names**

**Moderator:** How many drugs do you take?

**Respondent: I told you that I don’t know how to read. I just swallow the drugs**

**Moderator:** How many tablets do you swallow?

**Respondent: They give me a white tablet, a long one, a red one and a yellow one**

**Moderator:** Ok, you said that you’ve been having this condition for the last 10 years, have they been adding you more drugs are they have been reducing?

**Respondent: What?**

**Moderator:** Tablets

**Respondent: I just told you that I only receive the drugs but I don’t know the names**

**Moderator:** I was asking to know, from when you were diagnosed with high blood pressure ten years ago, how many tablets were you taking at first?

**Respondent: I don’t know. At first it was 130 and I was given many tablets. I didn’t count to know the exact number of tablets because I was given the tablets to help myself**

**Moderator:** For now, have they added or reduced your tablets

**Respondent: Normally when I go to the hospital I see a doctor who prescribes drugs then I go to the pharmacy who ask me if I want drugs that will last for one week or two for him to know what to give you because it depends with the amount of money that you have**

**Moderator:** Ok

**Respondent: If you tell him that you want drugs that can last you for two weeks then that’s what he writes for you. When I go there after 2 weeks they prescribe drug worth 1500 sometimes 2000 or sometimes 1200. That’s how it goes**

**Moderator:** Ok. How has this high blood pressure condition affected you?

**Respondent: Sometimes I feel headaches or sometimes if feel that my temperatures are high. Just like that**

**Moderator:** Apart from taking drugs, is there anything else you do to control your blood pressure?

**Respondent: No there is nothing else apart from drugs**

**Moderator:** What of food?

**Respondent: I was not asked to avoid taking any food, the doctor told me not to use a lot of sugar but with food I eat every types of food. There is no food that I don’t take**

**Moderator:** What of doing exercise?

**Respondent: I can’t manage that because am an old mother**

**Moderator:** Apart from taking drugs, is there anything else you do to control your blood pressure?

**Respondent: I don’t take anything else**

**Moderator:** Ok, who do you see when you go to the hospital coz of your blood pressure?

**Respondent: I see the doctor because I go on the day that I am booked. I can’t just go there on any day. I go there on the booked date because I know that there will be a doctor who comes from another facility and deals with this kind of diseases**

**Moderator:** How is this doctor managing your blood pressure?

**Respondent: He is a good doctor coz when I go there he measures my hand then he directs me to go extract blood for it to be tested too then from there I go to a doctor who prescribes drugs that I am supposed to be given**

**Moderator:** Where do you go for your clinics?

**Respondent:** **I go to the sisters’ hospital when I am in Nairobi**

**Moderator:** Which hospital?

**Respondent: {Name of the facility}**

**Moderator:** Ok, have you gone somewhere else apart from where you mentioned?

**Respondent: When I am at my rural home I go to another one**

**Moderator:** So, where do you stay?

**Respondent: I stay at {Name of a place}**

**Moderator:** {Name of a place}?

**Respondent: Yes, when you are in {Name of a place}, the village that is near the market**

**Moderator:** Where can you get hypertension services in the community that you live in?

**Respondent: You mean drugs?**

**Moderator:** Where can you get hypertension test services in the community that you live in

**Respondent: Maybe I go to the a facility in {Name of a place} or at {Name of the facility} because the {Name of the facility} is closed**

**Moderator:** What services do you get at the {Name f the Facility}?

**Respondent: When I go for clinic?**

**Moderator:** Yes

**Respondent: I don’t go on Monday; I go there on the other day**

**Moderator:** Tell me about the services you get at the sisters’ place. How are the services there?

**Respondent: I told you that I only go for drugs. They take my measurements then they give me drugs**

**Moderator:** I wanted to know the services that you get there like for example drugs, blood pressure measurements, if they do teach you so that I can know how it is there

**Respondent: Just the drugs because when I go there that’s what I am given. I used to go to a different hospital before where they did tests but they couldn’t tell my problem but when I went there they diagnosed my condition and I liked going there since they diagnosed me I believed that they will give me drugs. I just feel that I am getting better when I go to that place. I don’t have money for the other hospital**

**Moderator:** Do you have any problem in managing your blood pressure condition?

**Respondent: I have a problem with my leg and sometimes I feel that my blood pressure is high and I am also diabetic**

**Moderator:** What makes it difficult for you to control your blood pressure?

**Respondent: What do I tell you? We should just avoid thinking so much because this pressure rises when one gets any problem. To control pressure one should avoid thinking too much. When you engage your brain so much thinking of what to do or what to eat then the pressure will rise**

**Moderator:** Tell me the individual factors that make you not be able to control your blood pressure. You told me that you have problem with medicine and you also mentioned that the that you miss drugs in the hospital that you go for your clinics, you also said that your age is also a factor and you also added that sometimes you take drugs and other times you don’t because you don’t have money

**Respondent: And also sometimes I think of what to eat. When one gets money he or she must set aside money for drugs and food because you can’t take drugs and miss food**

**Moderator:** What are the family or community factors that make you unable to control your blood pressure?

**Respondent: nowadays if you have kids they can help you but first they must sort their problems in their homes first. The economy is tough, others would want to help you but they have their own kids, others don’t have money and you can’t beat them. You just distribute the little they give you to find what you will eat.**

**Moderator:** You said that you attend your clinic at the sisters’ place to get high blood pressure care services. What challenges do they have that make you unable to control your blood pressure?

**Respondent: I told you that it rises because of thinking too much**

**Moderator:** Do you experience any problem at the {Name of the facility}?

**Respondent: When the doctor gives you drugs then the decision becomes yours. If you don’t take the drugs then that’s your own problem**

**Moderator:** Do you receive any teachings there?

**Respondent: Where?**

**Moderator:** At the {Name of the facility}, do they give you advice?

**Respondent: The doctor tell you on how you can take drugs, there is no any other advice**

**Moderator:** Does it take you long to be served at the {the facility} are you are served faster?

**Respondent: You just go home once they are done with you**

**Moderator:** You said sometimes you miss drugs when you go to the hospital but do you take long or a short time at the hospital?

**Respondent: We just make ques in case we find that there are many people to be served. That’s how it is at the hospital. You can’t be served before those that you found there are served**

**Moderator:** Are there enough measuring gadgets at the {Name of the facility}?

**Respondent: Yeah, they have everything, I always see them measuring people**

**Moderator:** From what you have told me, what do you think can be done better to help you manage your blood pressure? What else can we do to solve the problems that you have mentioned?

**Respondent: You mean what else can we do?**

**Moderator:** You told me that you have a problem with the prices; you said that sometimes you lack drugs and you also mentioned problems that come from the family, you also told me that you think a lot and you also said that you take long for you to be served at the hospital so what can we do to solve these problems?

**Respondent: What else do I tell you and I have already told you that we have problems with finances and when there is a problem is you to know how you can help us. You are the one to know because I have told you all the problems. Just find a way to help me with my problems**

**Moderator:** What can you do differently as a high blood pressure victim to manage your blood pressure? What can you do that you think you are not doing

**Respondent: People should not think a lot on how to get drugs and food. I don’t have pressure problems when I have all those because at that moment I am full, I can take medicine, and my brain is relaxed, I can rest a little during the day or wash my clothes. I always feel good when I sleep knowing that I don’t have any problem when I wake up. You also know that we use our brains when we think and your pressure rises when you engage your brain too much**

**Moderator:** What can the doctor that you see at the facility do differently to manage your blood pressure?

**Respondent: I told you that I go there when I have money**

**Moderator:** Yeah, you told me that you go to the {Name of the facility} where you are given medicine but your pressure is still not ok

**Respondent: Their medicine is not bad; it depends with what you have. I can’t say that the doctor is bad because I know the door that I use when I go there and when I go there I just see the doctor who measures my blood pressure before treating me. He sends me to be extracted blood after I have been measured and I take my blood back to the doctor who measured my pressure who prescribes the drugs then I go to the pharmacy to collect my drugs**

**Moderator:** Ok, what about the hospital. What can you tell me about the hospital? What can they do differently to manage your blood pressure?

**Respondent: That Swahili is tough I don’t understand**

**Moderator:** You said that you go to the {Name of the facility} for clinic

**Respondent: I don’t have a problem with the doctor; he is not the one that makes my pressure to rise. He is just a doctor. The doctor doesn’t not cause any problem in regards to my pressure**

**Moderator:** What of the hospital itself? How is the hospital?

**Respondent: I go to the {Name} hospital where currently they have clinics for….26:12- 26:15… (Not clear)**

**Moderator:** How has COVID 19 affected the way you get blood pressure services?

**Respondent: What’s up with Corona?**

**Moderator:** I mean how Corona has affected the way you get your hypertension care services at the hospital

**Respondent: I normally go for measurements at the hospital**

**Moderator:** You mean that you have not had any problems with your clinic attendance?

**Respondent: No**

**Moderator: Is there anything else that you would like to talk about this pressure and you feel that we have not talked about**

**Respondent: No, there is nothing else**

**Moderator: Ok, thank you, I’ll call you later**

**Respondent: Ok**

**…END…**
