# Supplementary material for: A socio-ecological framework examination of drivers of blood pressure control among patients with comorbidities and on treatment in two Nairobi slums; a qualitative study
Source: PLOS Glob Public Health. 2023 Mar 10;3(3):e0001625. doi: 10.1371/journal.pgph.0001625 (PMC10021823; doi:10.1371/journal.pgph.0001625)
Supplement: S1 File — (ZIP) [file pgph.0001625.s001.zip › Community/KOCH-IDI-UHTNC-200710-001.docx]

**Moderator: Name**

**Code:** **KOCH-IDI-UHTNC-200710-001**

**Moderator:** This community has been identified to have a high burden of uncontrolled hypertension which is a leading factor to premature deaths and disability. I am trying to gather information about hypertension care in your community. To avoid hypertension related complications, it is recommended that people with high blood pressure can change their lifestyles in regards to diet, physical activities, smoking, alcohol consumption and using blood pressure medication**.** So tell me about your experience with having high blood pressure

**Respondent: Mostly my legs pain, I feel a lot of heat and headache that forces me to go out at night just to feel the wind blow. Sometimes feels heat and other times cold**

**Moderator:** For how long have you been having high blood pressure?

**Respondent: Three years**

**Moderator:** Do you go to clinic or where do you normally go for tests?

**Respondent: I go for clinic at {Name of the facility}**

**Moderator:** Were you told your measurements the last time your pressure was measured?

**Respondent: The doctor just told me that my blood pressure was high and he took me to another room where I stayed for almost an hour then he referred me to{Name} Hospital for them to check …3:19… (Not clear) then I went but I was not treated there because I didn’t have the money that they were asking for and I went back and informed my doctor and then he gave me some two little tablets that were red and white in color**

**Moderator:** Do you know those drugs? Can you tell me the names?

**Respondent: I can’t read my doctors handwriting but I know the tablets. There was one that was white. I used to break the tablets into two at the beginning but nowadays I take a full tablet**

**Moderator:** When was it changed?

**Respondent: You mean the tablet**

**Moderator:** Yeah, when did you start swallowing a full tablet?

**Respondent: I just told you that I have been having this condition for the last 3 years and at the first the doctor used to tell me that I should break the tablet into two and swallow on half at night but when the condition worsened that’s when he told me to take two tablets. There this one that is small and red in color and a white one. I swallow the two tablets**

**Moderator:** Have you been told your target blood pressure when you go for measurement?

**Respondent: The doctor just tell me that my pressure is not ok and I am supposed to avoid thinking but I tell him that I cannot avoid thinking because I have many things that need me and that’s why I have to think. Sometimes he tells me to relax because my blood pressure might cause me problems. So sometimes I relax but when I face any problem my blood pressure shoots again**

**Moderator:** Were you told your readings the last time you were measured?

**Respondent: If you want to know that then I will have to go to the doctor day you will call me so that he can read that book because I also have a file opened. I just go with my book but they keep the records in my file**

**Moderator:** How often do you take your blood pressure measurements?

**Respondent: I do measure monthly**

**Moderator:** Do you have other conditions apart from high blood pressure?

**Respondent: My doctor told me that I am diabetic and my legs are swollen. The doctor asked me why my legs were swollen and I told him that they swell coz sometimes when I wash clothes and water spills on them**

**Moderator:** How has this condition of high blood pressure affected you?

**Respondent: Do you know that I bend while walking? I can’t walk for a long distance and I used to walk straight but nowadays I have to bend on one side**

**Moderator:** Apart from using medication, how else do you manage your blood pressure?

**Respondent: My doctor told me to use a lot traditional vegetables and to avoid using a lot of oil, that’s what he told me**

**Moderator:** What else?

**Respondent: Also fruits**

**Moderator:** What about exercise?

**Respondent: He also told me to be walking when I wake up in the morning for my legs stop bending the way they are**

**Moderator:** Do you use traditional medicine?

**Respondent: No I have never used any traditional medicine, I fear them. There is a time way back when I used traditional medicine while still menstruating and it made me bleed a lot and I have never used it again**

**Moderator:** Was the traditional medicine meant for high blood pressure?

**Respondent: No**

**Moderator:** What was it for?

**Respondent: It is not now, it is like 5 or six years ago**

**Moderator:** What else do you do to manage your blood pressure?

**Respondent: I was told to do exercise and where we stay you just have to do something; you are not supposed to just sit because you are sick. One has to do some house chores for you to feel that your body is ok**

**Moderator:** How has hypertension affected you?

**Respondent: it has affected me a lot, I just told you that I walk bending on one side. I cannot walk the way I used to walk before, I bent on one side and I cannot do casual jobs like I used to do, I am just in the house**

**Moderator:** Do you have anything else to add?

**Respondent: No**

**Moderator:** Apart from using medication, how else do you manage your blood pressure? You mentioned diet, exercise and use of drugs

**Respondent: That’s all**

**Moderator:** Do you see a doctor or a nurse?

**Respondent: I see a doctor, a big doctor at the facility. I hear people call him a Clinic Officer**

**Moderator:** What can you say about the way your health provider is managing your blood pressure?

**Respondent: He knows my condition to an extend that he attend to me first whenever he sees me in a queue. He is a very good doctor; he never wants to see me in a queue.**

**Moderator:** Ok, have you ever sought treatment elsewhere?

**Respondent: Only at {Name of the facility} where I was told to go but you know one has to pay there and getting money is a problem more so with this Corona. There is no money**

**Moderator:** Where can you get hypertension care service in this community? You had told me about {Name of the facility}, is there any other place?

**Respondent: I was told about Name of the facility and Name of the hospital or in Kiambu county**

**Moderator:** Is there any other place within Korogocho village?

**Respondent: No, we just go to {Name of the facility}**

**Moderator:** Tell me the normal procedure when you go there and the services that you get there, how do they start?

**Respondent: when I go there I am asked to sit on a seat then they bring the blood pressure measuring gadget to measure my pressure and after that they measure my weight and height then I go to see the doctor**

**Moderator:** Do they give you medicine when you go for the clinics?

**Respondent: Drugs have not been there during this Corona time so when we go to the hospital the doctor tells us to buy if we can and if you can’t buy then we have to wait until when the drugs will come**

**Moderator:** So you mean that you don’t pay there?

**Respondent: We are not charged but the doctor tells us to buy when the drugs are not there**

**Moderator:** How often do you normally go for clinic?

**Respondent: Once in every month**

**Moderator:** Did you say that you were referred to {Name of the facility}the last time you attended clinic?

**Respondent: Yeah**

**Moderator:** So you have not gone elsewhere since that time

**Respondent: No I have not gone elsewhere**

**Moderator:** Do you have any problem with managing your blood pressure?

**Respondent: I just told you that my legs are swollen**

**Moderator:** You said that you were asked to pay money at {Name of the facility}

**Respondent: When you enter {Name of the facility} you have to be given a card that you must pay for and after paying for the card and before you see a doctor you are asked to do a blood test and other tests that you have to pay for yet you don’t have money**

**Moderator:** Do you pay from the pocket or you use an insurance card?

**Respondent: I don’t have an insurance card**

**Moderator:** Do you think that your age is a challenge?

**Respondent: My age is not a problem coz am not that old, my body is just weak because of this condition**

**Moderator:** You told me that you are also diabetic, when were you diagnosed of diabetes

**Respondent: I have been having blood pressure for 3 years and I have been diabetic for one year.** **I was told not to take sugar; I normally take tea and porridge without sugar**

**Moderator:** Do you take your drugs as prescribed by the doctor?

**Respondent: Yeah, I take medicine as prescribed**

**Moderator:** You told me that you used to do some casual jobs but nowadays you don’t go because your legs are swollen. What else do you do to help yourself at home?

**Respondent: For now I don’t do anything but sometimes food is distributed at our houses by people from Miss Koch organization. They usually bring us something to eat when they visit us**

**Moderator:** Are you using either alcohol or cigarettes?

**Respondent: No, I have never**

**Moderator:** Does your family have any problem in managing your blood pressure?

**Respondent: There is one lady who used to work in town who used to help me but nowadays she doesn’t help me coz she is no longer working**

**Moderator:** Is your diet a challenge in managing your blood pressure?

**Respondent: Feeding is a problem because traditional vegetables are expensive and sometimes we have to eat kales when we can’t get traditional vegetables and again we only eat one meal a day**

**Moderator:** Looking at your health care provider, does he contribute to your challenge of managing your blood pressure?

**Respondent: No, he is just ok**

**Moderator:** What of the quality of health care that you receive, how is it?

**Respondent: Their health care is ok**

**Moderator:** Looking at the way they do their work, is the number enough to serve you well?

**Respondent: Yeah, they serve us well. Hypertensive patients are treated first when we go there**

**Moderator:** What of clinic hours?

**Respondent: We are told to go early**

**Moderator:** Are the hours affecting you in any way?

**Respondent: No, I normally go there at 9 and I always get treated**

**Moderator:** How does the information that you receive from your doctor help you?

**Respondent: Its helping me coz I am feeling better than I was feeling before**

**Moderator:** Looking at drugs, you said that the drugs are not available coz of Corona. How was it before?

**Respondent: It was ok. We were not missing drugs. We used to get drugs on the day that we were booked for clinic**

**Moderator:** And when you go for clinic, do you find the space ok? The space at the hospital

**Respondent: It’s ok**

**Moderator:** Have you ever been told about the guidelines that are supposed to be followed at the hospital as they attend to you?

**Respondent: For me I can say that they attend to me well**

**Moderator: What can be the solution to the hindrances that you have mentioned? You had told me about issues of money**

**Respondent:** What I pray to God is that I get money. Last time you really helped me when you sent me 500 shillings because my landlord was about to chase me out of the house and I gave him that money

**Moderator:** You also talked about lack of drugs, how can we solve that?

**Respondent: We can just go to the chemist and get drugs worth the money that we have.at the clinic we are given drugs that can take us for one month and when there are bot there then we are forced to buy drugs that can take us for a day or two or three**

**Moderator:** You also talked of that lady that used to help you and at the moment she is not able to help you, how can we help you on that?

**Respondent: For now she is just there**

**Moderator:** How has COVID 19 affected how you receive your hypertension care services?

**Respondent: It has affected us coz sometimes you don’t get medicine when you go for clinic. It would be better if we could get drugs**

**Moderator:** What else?

**Respondent: Feeding is a problem, paying rent is a problem. Those are the things that contribute and again if you have kids then you have to think and when you think that’s when the pressure rises**

**Moderator:** Is there anything else that you want us to talk about in regards to high blood pressure condition?

**Respondent: Yeah, tell me if you know**

**Moderator:** I would like to hear from you if at all, there is anything else that you would want us to talk about

**Respondent: No, there isn’t**

**Moderator: Ok, thanks so much for the time that you have given us and I hope that it will help us in our research**

**Respondent: Ok**

**Moderator: Thank you very much**

**…End…**
