# Supplementary material for: A socio-ecological framework examination of drivers of blood pressure control among patients with comorbidities and on treatment in two Nairobi slums; a qualitative study
Source: PLOS Glob Public Health. 2023 Mar 10;3(3):e0001625. doi: 10.1371/journal.pgph.0001625 (PMC10021823; doi:10.1371/journal.pgph.0001625)
Supplement: S1 File — (ZIP) [file pgph.0001625.s001.zip › Community/KOCH-IDI-UHTNC-200712_0542.docx]

**Moderator: {Name}**

**Code: KOCH-IDI-UHTNC-200712_0542**

**Moderator:** This community has been identified to have a high burden of uncontrolled hypertension which is a leading factor to premature deaths and disability. I am trying to gather information about hypertension care in your community. To avoid hypertension related complications, it is recommended that people with high blood pressure can change their lifestyles in regard to diet, physical activities, smoking, alcohol consumption and using blood pressure medication**.** So, tell me about your experience with having high blood pressure**.** Tell me about your experience with having high blood pressure

**Respondent: Sometimes I walk of foot, I eating foods that were are told to eat, and I don’t like problems. The most important thing is walking on foot and eating foods that I am told to eat. For now I don’t see anything else**

**Moderator:** For how long have you been having this high blood pressure condition?

**Respondent: For a while**

**Moderator:** Like for how long

**Respondent: From 2015**

**Moderator:** How often do you check your blood pressure?

**Respondent: I have had it for long please**

**Moderator:** You told me that you have been having high blood pressure from 2015

**Respondent: It is even longer than that**

**Moderator:** So how long do you take for you to check your blood pressure?

**Respondent: I go for check up on every Tuesday, once a week**

**Moderator:** Once s week?

**Respondent: Yeah, and we pay**

**Moderator:** Where do you go to check your blood pressure?

**Respondent: I normally go to {Name of the facility}**

**Moderator:** What’s the name of the place?

**Respondent: I think it is called {Name} church**

**Moderator:** Do you record somewhere your blood pressure measurements

**Respondent: He gives me a receipts and a small paper after paper that I use to make payment**

**Moderator:** What was the reading the last time you were measured?

**Respondent: the last reading was 118/80**

**Moderator:** Do you have any other condition apart from high blood pressure?

**Respondent: Yeah, I have diabetes**

**Moderator:** Has you doctor ever told you what your target pressure readings should be?

**Respondent: He told me that my pressure is not going down, it is always high**

**Moderator:** Has he ever told you your target level?

**Respondent: He told me that it’s supposed to be 149**

**Moderator:** Over what?

**Respondent:** I have forgotten

**Moderator: Ok**

**Respondent: I think it’s over 60 or 70**

**Moderator:** Over 70?

**Respondent: I have forgotten**

**Moderator:** Tell me about the antihypertensive medicines that you are using

**Respondent: I use …5:48… (Not clear) and a small white tablet**

**Moderator:** Ok

**Respondent: I use 3**

**Moderator:** You are using three types of medicine?

**Respondent: Yes**

**Moderator:** Have your drugs been added or have they reduced from the day you started using antihypertensive?

**Respondent: The drugs have been added, there is no drug that has been reduced, and they are very many. It’s even is a problem buying the drugs**

**Moderator:** Have you ever asked you doctor why he has been increasing your drugs?

**Respondent: The doctor tells us that it’s a regulation by the government. There is nothing we can do**

**Moderator:** Have you ever asked the doctor the reason as to why he was increasing your medicine?

**Respondent: I have asked him severally but he says that that is according to government regulations. That’s what he tell me**

**Moderator:** Has the doctor ever told you that the strength of your medicine has increased since you started taking them

**Respondent: They changed long time ago**

**Moderator:** How has hypertension affected your life?

**Respondent: It has caused many problems. Sometimes I find it hard to walk; sometimes I feel that my body is heavy. It’s just like that**

**Moderator:** Apart from using medicine, how else do you manage your blood pressure?

**Respondent: I just take medicine as I have been instructed. There is no any other way that I can use to manage my pressure**

**Moderator:** What about dieting and doing exercise?

**Respondent: Am very old, I cannot do exercise, I cannot do many things. l cannot even run**

**Moderator:** What of food?

**Respondent: Food is ok because I eat what I have been cooked for**

**Moderator:** What else do you do to ensure that your pressure is ok?

**Respondent: I cannot see anything else**

**Moderator:** Apart from taking drugs, what else do you do?

**Respondent: Just the work that I do**

**Moderator:** What do you do?

**Respondent: I do my own work**

**Moderator:** Who do you see when you go to {Name of the facility}?

**Respondent: I see a doctor who takes my blood pressure measurements and he tells me that my pressure is not good. There is a time when he added me water. I have some little problems. Did I tell you that my legs are swelling?**

**Moderator:** Yes, you told me**,** what can you say about the way your doctor is managing your blood pressure?

**Respondent: There is a problem. There are doctors who don’t know. Blood pressure matters are very tough because you can meet a doctor who doesn’t know, it’s like he is being taught about the condition. You know people are different**

**Moderator:** What’s your view about the doctor that attends to you at {Name of the facility} in Kariobangi?

**Respondent: I could have changed if I had somewhere else I could go. These drugs are very expensive so you just have to persevere as you wait for your God**

**Moderator:** Have you ever sought treatment elsewhere apart from the {Name of the hospital?

**Respondent: There is a time I went to a hospital in Kiambu but I don’t think that the treatment was ok. I was served very fast then they left me**

**Moderator:** Was it a hospital or a clinic

**Respondent: It was a hospital**

**Moderator:** You mean {Name of the hospital}?

**Respondent: Yeah, I was admitted there**

**Moderator:** Which year was this?

**Respondent: It was in March**

**Moderator:** March this year

**Respondent: No February this year. I was admitted there**

**Moderator:** Sorry. Where do you stay currently?

**Respondent: I stay in {Name of a place}**

**Moderator:** Ok, so the hospital that is closer to you is {Name of the facility}?

**Respondent: Yeah if you know {Name of a place} then you can meet me at the church**

**Moderator:** What services do you receive at the {Name of the facility}?

**Respondent: They measure my pressure and then prescribe my medication then I pay for the medicine and go home**

**Moderator:** Tell me about the drugs that they give you and the advice that they share with you

**Respondent: You know that’s a hospital. When i go there they take the blood pressure measurements, he tell me to use antihypertensive then I go home**

**Moderator:** You said that you go for clinic on every Tuesday of every week?

**Respondent: Yes, every Tuesday**

**Moderator:** Ok, do you have any problem in managing your blood pressure?

**Respondent: There are many problems**

**Moderator:** Tell me please those problems

**Respondent: Sometimes I don’t have money and that make me not to go for clinic, there are other times when I am given medicine and I finish them before my clinic date and there is nothing I can do. I just wait for my next clinic on Tuesday the other week**

**Moderator:** You mean that you are given medicine every time you go for clinic on Tuesday?

**Respondent: Yes, am given on every Tuesday**

**Moderator:** How long can the drugs that they gave you this week on Tuesday last?

**Respondent: They gave me…14:35- 14:36… (Not clear)**

**Moderator:** How long does the drugs last

**Respondent: Just one week and if I get money I can go back**

**Moderator:** Ok. You were telling me the problems that you have. I remember that you said sometimes you lack money to buy drugs

**Respondent: Yes, I lack money**

**Moderator:** You told me that you are not young

**Respondent: Yes, am not young, am very old**

**Moderator:** Tell me the individual factors that are making it hard to control your blood pressure

**Respondent: You may think of doing something but you are not able to do. You have to think about it**

**Moderator:** What about you taking drugs on time?

**Respondent: Prescriptions are always the same because I am supposed to take drugs in the morning at 9am and at 9:00pm night**

**Moderator:** So you take your drugs twice a day

**Respondent: Yes. I take** **twice a day**

**Moderator:** What of communal and family factors that hinder you from managing your blood pressure?

**Respondent: Maters of the house are very heavy. There are sometimes when you get angry when you see that your business is not doing well and also these COVID19. There are many things mummy**

**Moderator:** Tell me about where you live if there are any challenges on getting food, getting a space for walking. Just tell me about the place that you stay

**Respondent: Getting food is hard. You may want to eat this type of food, but you don’t have, you may want to do something but you can’t do it. There are many problems**

**Moderator:** Tell me about the quality of treatment that you get at the sisters’ place

**Respondent: We go there on Tuesday. I’ll go there if I’ll be having money to be attended to and purchase medicine**

**Moderator:** Tell me about the hours that you take to be served at the clinic. Does it take long or short time for you to be served?

**Respondent: It does not take long. I normally take like 20 minutes**

**Moderator:** Are you given any advice when you go for clinic at {Name of the facility}?

**Respondent: No**

**Moderator:** Does the doctor teach you about blood pressure? What does he do?

**Respondent: He measures blood pressure, he prescribes medicine then he tell you to go take the medicine and tells us to come back on Tuesday and when you go back the doctor checks your measurements again. We don’t get any other instructions**

**Moderator:** Tell me about the government, what they are doing that or what are they doing to help you manage your blood pressure

**Respondent: The government is not doing enough because we have many hypertensive patients. They can decide to give us medicine but they are not. We are forced to look for money to go buy drugs and if you don’t have the money then that’s your problem. That’s where the problem is**

**Moderator:** You have mentioned many challenges caused by you as an individual, health care providers and the government. What do you think could be the possible solution to the problems that you mentioned? Starting with you, what do you think you can do differently to manage your blood pressure?

**Respondent: Pressure needs exercise and many other things. If we get a volunteer of somebody to be paid by the government to help us does exercise then it would be better because people are really pressed. You are sick and you don’t have money to go to the hospital. There are many problems**

**Moderator:** You also told me that there are times that you go to the hospital but you miss drugs

**Respondent: Yeah, we do miss**

**Moderator:** Tell me what we can tell the government to do so that we can be helped

**Respondent: Tell the government to help us get medicine if they can and those that provide care should be advising us on what to do. Advice is good**

**Moderator:** What can you do differently as a hypertensive patient to manage your blood pressure?

**Respondent: For the pressure to rise or go down?**

**Moderator:** To be at the normal required target

**Respondent: You will only be told your progress after your pressure has been measured because pressure is not visible. Sometimes you wake up feeling heavy**

**Moderator:** How has COVID19 affected service delivery for hypertensive patients?

**Respondent: If we can be served then it will be better**

**Moderator:** Am asking how COVID19 has affected service delivery for hypertensive patients at the hospital?

**Respondent: That’s a big hindrance**

**Moderator:** Tell me how

**Respondent: Hello can you hear me?**

**Moderator:** That place is noisy, I can’t hear you. Kindly move away from those kids. We are about to finish

**Respondent: Ok**

**Moderator:** Tell me if there is anything that we have not talked about pressure and you would want us to talk about

**Respondent: We have talked about everything. You have told me about that high blood pressure and I don’t see any hindrance**

**Moderator:** Thanks for the time that you have set aside for us to have this conversation

**Respondent: Ok**

**Moderator: And I hope that the challenges that you have given me will make us able to see where the problem is**

**Respondent: Ok**

**…END…**
