# Supplementary material for: A socio-ecological framework examination of drivers of blood pressure control among patients with comorbidities and on treatment in two Nairobi slums; a qualitative study
Source: PLOS Glob Public Health. 2023 Mar 10;3(3):e0001625. doi: 10.1371/journal.pgph.0001625 (PMC10021823; doi:10.1371/journal.pgph.0001625)
Supplement: S1 File — (ZIP) [file pgph.0001625.s001.zip › Community/KOCH-IDI-UHTNC-200712_2239.docx]

**Moderator: {Nmae}**

**Code: KOCH-IDI-UHTNC-200712_2239**

**Moderator:** This community has been identified to have a high burden of uncontrolled hypertension which is a leading factor to premature deaths and disability. I am trying to gather information about hypertension care in your community. To avoid hypertension related complications, it is recommended that people with high blood pressure can change their lifestyles in regards to diet, physical activities, smoking, alcohol consumption and using blood pressure medication**.** So tell me about your experience with having high blood pressure**.** Tell me about your experience with having high blood pressure

**Respondent: For me am both diabetic and hypertensive so I think I have two challenges, one comes from the way am supposed to manage diabetes and the way am supposed to take antihypertensive to control my blood pressure but the biggest challenge that I have is to control diabetes that is causing high blood pressure**

**Moderator:** For how long have you been hypertensive?

**Respondent: Since 2004**

**Moderator:** How often do you check your blood pressure and where do you go to check?

**Respondent: I just go to check to a clinic when I feel that my blood pressure is high**

**Moderator:** Do you note down your measurements after checking at the clinic?

**Respondent: Yeah, I do record and go where I normally go for treatment in case I find that the blood pressure is high**

**Moderator:** What was the measurement the last time you checked?

**Respondent: The last one?**

**Moderator:** Yeah, the last one

**Respondent: I can’t remember the last one but it was not bad according to the one who measured me. I was told that its high but it was not very bad**

**Moderator:** You said that there is a place that you go when your blood pressure is high, what’s the name of the place that you normally go to

**Respondent: There is a nearby clinic located in Korogocho**

**Moderator:** You said that there is a place that you go when you find that your pressure is high

**Respondent: There is a place that I go for treatment**

**Moderator** Where?

**Respondent: {Name of the facility}**

**Moderator:** Have you ever been told by your doctor about your target pressure level?

**Respondent: He normally give me and he explains to me how am supposed to take care of myself**

**Moderator:** What did he tell you that you measurement should be?

**Respondent: I don’t remember well, I just depend on what the doctor tells me when I go for checkup and he tell me that my blood pressure is ok**

**Moderator:** Tell me about your medication on high blood pressure. How many drugs were you taking at first?

**Respondent: I started with two types**

**Moderator:** As per now you are still taking two?

**Moderator: For now am taking only one**

**Respondent:** Have you increased the types of the drugs that you were taking when you were first diagnosed or have they reduced in number?

**Respondent: They were added**

**Moderator:** Did the doctor tell you the reason as to why they increased in number?

**Respondent: I started with two but at the moment am taking four**

**Moderator:** Did the doctor tell you the reason as to why they increased in number?

**Respondent: The drugs are increasing coz maybe I can’t manage or because of diabetes**

**Moderator:** Has the strength of the drugs that you are taking increased or how has it been?

**Respondent: It’s increasing because when he finds that my pressure is very high, the he tell me to take two**

**Moderator:** Tell me how blood pressure has affected your life

**Respondent: The first thing is that my body is weak because there are some tasks that I cannot do, I can’t do any heavy task or if I do then I’ll be taking rest every now and then and the other thing is like my manhood is not active**

**Moderator:** Apart from taking drugs, what else do you do to manage your blood pressure?

**Respondent: Mostly I try doing exercise; I walk instead of taking motorbikes or vehicles and trying to look at my feeding because I am diabetic**

**Moderator:** You said that you attend your clinics at {Name of the facility}, who do you see when you go there?

**Respondent: There are doctors who deal with diabetes and pressure**

**Moderator:** Are they there specifically to deal with diabetes and high blood pressure?

**Respondent: Yeah, there is a doctor who deals with that kind of people**

**Moderator:** What can you say in regards to the way these doctors are managing your pressure?

**Respondent: I meet around three different doctors but so far I can say that they are good**

**Moderator:** Have you ever sought hypertensive treatment elsewhere apart from where you have been going?

**Respondent: I started at {Name of the facility} and there the treatment was standard, everything was ok, but I had to change because they don’t accept use if insurance card. I changed and went to {Name of the facility} but I realized that they didn’t have any information about diabetes. I am on insulin so they were telling me to go there for injection but I couldn’t managed to go there in the morning and evening yet I can inject myself. That when I realized that they are not informed on diabetes. I changed and went to S.O.S in {Name of a place} but I found out that they don’t have someone who is qualified to do that that’s when I went to {Name of the facility. At {Name of the facility} I can say that I have been assisted by the people that are there**

**Moderator:** What kind of services do you get when you go to {Name of the facility}?

**Respondent: There are many types of test at {Name of the facility}; I do all the tests that are requires on diabetes and when my pressure is high. They just send me to a lab where I take all the tests**

**Moderator:** How far is {Name of the facility} from where you stay? Is it far or just near?

**Moderator: It’s far and I must use a matatu. I stay at {Name of a place}and you can see that Korogocho is far from {Name of the facility}**

**Moderator:** Why can’t you get this hypertensive care service at a place closer to where you stay?

**Respondent: Like where?**

**Moderator:** You said that you stay in {Name of a place} but you go to {Name of the facility}, why can’t you get these services where you stay or closer to where you stay

**Respondent: I think they don’t have a lab; they will have to refer you outside when they need lab services and again I use an insurance card and if I am treated then then I will have to pay cash. There are some areas where my insurance card helps me and other places I use cash so if I go there then I will not be assisted the way I am supposed to be assisted**

**Moderator:** For of these services that you get using your insurance card, where do drugs fall?

**Respondent: Sometimes I don’t get all the drugs, sometimes am forced to go to the chemist and they are very expensive**

**Moderator:** Tell me about the advice that you are given there and also tell me how often you go for clinic

**Respondent: I attend my clinic monthly and they monitor how the situation is and the give advice on what one is supposed to do**

**Moderator:** You said that it is difficult to manage your pressure because you are diabetic and you also said that sometimes you lack drugs

**Respondent: Yeah, sometimes drugs are not there and we are forced to go but at the chemist**

**Moderator:** And you also told me that you can’t get services in the clinic closer to you because you would like to use an insurance card and they don’t allow that there. Is there any other problem that you can add to that?

**Respondent: Apart from that I would say that their laboratories are not standard because there some test that should be done like the heart beat among others but they dint have that. Their laboratories are of low standard**

**Moderator:** What are the individual of personal factors that make you not to be able to control your blood pressure?

**Respondent: I think to control my blood pressure I just need to manage diabetes, take medicine ,do exercise and eat what I am supposed to eat and that the challenge because it is only one person in the family so sometimes having two budgets for one family becomes a challenge especially at this moment**

**Moderator:** What are the communal or family factors that can make it a challenge for you to control your pressure?

**Respondent: I think its misunderstanding; maybe your family doesn’t know your condition because this condition doesn’t want one to be angered, when you are angered then the blood pressure rises, the other issue is food, maybe they have prepared a different type of food when you are supposed to take a different type so you don’t understand each other.**

**Moderator:** What do you think that your health provider is not doing to help you manage your blood pressure?

**Respondent: Time is the big issue because the doctor might want to see maybe 10 patients but the time doesn’t allow him. He will end up not having enough time with each person and if one has questions then the doctor might not answer as he is supposed to because he has to consider time and the patients that he is supposed to attend to**

**Moderator:** How about the number of health care providers that are available during clinic days, are they many or?

**Respondent: Patients or**

**Moderator:** I mean the care givers

**Respondent: There is only one doctor and the others are nurses that only take pressure measurements then they leave the rest for the doctor**

**Moderator:** What of health system factors, what do you think makes it hard for hypertensive patients to control their blood pressure

**Respondent: Not following instruction and not taking medicine though some medicines have side effects, side effects make some people to stop taking medicine like for example the one that I mentioned about my manhood. One decided to stop taking certain types of drugs when he sees such**

**Moderator:** What are government factors that can make one unable to control blood pressure?

**Respondent: I think the drugs are very expensive and sometimes you go to the hospital but you don’t find drugs and when you go to the chemist you find that the drug that can last you at least for one month goes at 5000 shillings and maybe you need two types. Someone is forced to by a quarter dose instead of buying the whole dose. The government should know what to do like maybe not taxing such drugs so that the prices can be cheaper**

**Moderator:** What can you do as an individual to manage your blood pressure?

**Respondent: I think what I can do is just be strict and follow the instructions, take drugs as instructed and do exercise**

**Moderator:** What can your health care provider do differently?

**Respondent: They need to do research or improve in their studies because like diabetes and high blood pressure but especially diabetes is a disease that keeps on changing and not only diabetes, diseases keep on changing and different drugs are invented. They should go for refresher courses. If they go there then they will be able to help us**

**Moderator:** What of the government, what can they do differently?

**Respondent: I think that we don’t have people who are specialized in the government when it comes to diabetes and pressure. We don’t have specialized doctors to deal with such conditions. Doctors are there but they are there but they are very few so I think they should train many doctors on hypertension and pressure**

**Moderator:** Ok, we are about to finish, How has COVID 19 affected the way you receive hypertension care at the hospital?

**Respondent: What happened is that when I went there I was told to take drugs that can last me for three months because we were told to stay indoors and not to interact with people by going to the hospital every month. That’s why I was given drugs that could last me for three months while am indoors**

**Moderator:** What else has been affected by COVID 19 in terms of you receiving hypertension care?

**Respondent: The challenge in services is like missing drugs and there is no money. That’s the challenge that we have. People are financially affected yet some of us are on special diet. Getting that becomes a challenge**

**Moderator:** On to the last question, is there anything else that you feel that we have not talked about and we need to talk about in regards to high blood pressure?

**Respondent: I think traditional doctors are increasing in number and they don’t know what they are doing or they are doing somethings that might affect other people. There are those people who go to seek care from those people and I think it is not good. The government should create awareness and awareness should be created on hypertensive and diabetic patient so that they can know where they can go to seek care. Some are told that if they eat somethings they will help them in managing their blood pressure and they end up losing their money**

**Moderator:** Ok, we are done and I appreciate you for the information that you have given me and I think the challenges and everything that you have told me will reach the ones that are supposed to hear and it will help us as the community and hypertensive patients who don’t have this information. Thank you for your time and the information that you have given me.

**Respondent: Welcome**

**…END…**
