# Supplementary material for: A socio-ecological framework examination of drivers of blood pressure control among patients with comorbidities and on treatment in two Nairobi slums; a qualitative study
Source: PLOS Glob Public Health. 2023 Mar 10;3(3):e0001625. doi: 10.1371/journal.pgph.0001625 (PMC10021823; doi:10.1371/journal.pgph.0001625)
Supplement: S1 File — (ZIP) [file pgph.0001625.s001.zip › Community/KOCH-IDI-UHTNC-200713_0021.docx]

**Moderator: {Name}**

**Code:** **KOCH-IDI-UHTNC-200713_0021**

**Moderator:** This community has been identified to have a high burden of uncontrolled hypertension which is a leading factor to premature deaths and disability. I am trying to gather information about hypertension care in your community. To avoid hypertension related complications, it is recommended that people with high blood pressure can change their lifestyles in regards to diet, physical activities, smoking, alcohol consumption and using blood pressure medication**.** So tell me about your experience with having high blood pressure**.** Tell me about your experience with having high blood pressure

**Respondent: My husband died a death that I didn’t expect and this really shocked. He just…2:50… (Not clear) in the morning and the next minute I heard that he was no more. That’s when this condition started. From then my blood pressure has been rising whenever I hear about anything that shocks me for example this COVID situation. I did a diabetes test that turned out negative so the only condition that I have is high blood pressure**

**Moderator:** For how long have you been having this high blood pressure condition?

**Respondent: Since 2011. He died in June the year 2011**

**Moderator:** How often do you check your blood pressure?

**Respondent: I go to a clinic located close to my house after every two or three weeks**

**Moderator:** Where do you go to check your blood pressure measurements?

**Respondent: At the health Center**

**Moderator:** Do you record your measurements after the test are done?

**Respondent: Yeah, like the last measurements I was told that my blood pressure was 178 and I was told that it was high**

**Moderator:** It was 178 over what?

**Respondent: it was 178 and last time it was 153 so I don’t know if that is ok. How is it supposed to be?**

**Moderator:** It would be better if you always asked your doctor the normal blood pressure target

**Respondent: I will ask because the last measurement was 178**

**Moderator:** What if the other reading? It was 179 over what?

**Respondent: 110**

**Moderator:** 178/110?

**Respondent: Yes**

**Moderator:** What did the doctor tell you on that day?

**Respondent: He told me that it was very high that I try and reduce thinking and I try finish taking drugs coz sometimes I do forget. He also told me that I am supposed to be attending clinic**

**Moderator:** Has your doctor ever told you your target blood pressure?

**Respondent: He told me that it’s supposed to be around 150/90**

**Moderator:** Tell me about your high blood pressure medicines. How many types of drugs have you been taking?

**Respondent: I can’t pronounce the names but I do take a white and a yellow tablet but the names are hard to pronounce**

**Moderator:** How many tablets do you take?

**Respondent: I take two**

**Moderator:** You said that you have been having this condition since 2011, have they added you more drugs or they have been reduced in number?

**Respondent: My blood pressure goes gown when I take my drugs as directed but I was told that it’s not a must that I take the drugs continuously from date 1^st^ to date 30^th^ because most of the time they burn me coz I also have ulcers. I was told that I can also use thorn melon but I don’t miss taking drugs**

**Moderator:** Has the number of your drugs reduced or increased from the time you started taking medication

**Respondent: My pressure reduces when I stop thinking. I can’t say that the drugs are not good. They really do help me**

**Moderator:** Has the doctor increased or reduced the number of your drugs?

**Respondent: He has never reduced; He has never changed my drugs**

**Moderator:** How has blood pressure affected your life?

**Respondent: It has really affected because I find it had to get up when my blood pressure is high yet am the only one to provide coz I don’t have a husband. I am really affected coz I have to look after my kids; I live in a rental house so I just have to get up even when my pressure is high. There is a day I fell down at Eastleigh**

**Moderator:** Sorry

**Respondent: I had gone to look for some casual job then I fell down but I was helped**

**Moderator:** What do you do to manage your blood pressure apart from taking drugs?

**Respondent: My doctor told me to try reading a book or a bible to avoid thinking and try to ignore most of the things that you can’t manage now that you can’t totally avoid thinking because you have a family. He also told me that I should avoid being alone most of the time and try finding company**

**Moderator:** What about food and exercising?

**Respondent: He told me about that and he gave me a rope to skip. I do skip rope and I also run in the field because the doctor told me that my weight is also contributing to my blood pressure condition**

**Moderator:** You told me that you go to the Health enter for your clinics, who do you see when you go there?

**Respondent: I see Dr. Name**

**Moderator:** What can you tell me I regards to the way Dr. Name manages your blood pressure

**Respondent: He is very ok coz my blood pressure goes down even before treatment because of the way he counsels me. Personally I can say that he is ok and other patients say that he is good**

**Moderator:** Have you ever gone elsewhere apart from the health center to seek hypertension care?

**Respondent: Yeah, I went to {Name of the facility} and my blood pressure was measured**

**Moderator:** What did the doctor tell you there?

**Respondent: At {Name of the facility} I was told that my blood pressure was not that bad but I was advised to do exercise**

**Moderator:** When do you go to the health center?

**Respondent: I go there on Fridays and Mondays mostly**

**Moderator:** So you go there twice a week?

**Respondent: No, if I go for clinic this week on Monday, then I go on Friday in the following week for drugs but they just do the blood pressure measurement in case I have not finished my drugs yet. We were told that we can just check anytime and not necessarily wait until when the blood pressure is high. Sometimes I find that my blood pressure is high, sometimes I find it low but it’s all because of stress**

**Moderator:** How is it when you attend your clinics Monday or Friday?

**Respondent: You mean the way we are served?**

**Moderator:** Yes

**Respondent: I can’t say that it is bad. They attend to us very well especially us, people with high blood pressure**

**Moderator: How do you get your drugs?**

**Respondent: I have never been told that there are no drugs; I always find them when I go there. I have never been told to go and buy drugs**

**Moderator:** Do you have any problem with managing your blood pressure?

**Respondent: On my side?**

**Moderator:** Yes

**Respondent: Problems are always there but for somethings you just put God to in front**

**Moderator:** What of communal or family problems that might hinder you from managing your blood pressure?

**Respondent: You will always experience such things once someone close to you dies because people will say many things, maybe your child thinks that you are refusing to do what he or she wants to be done. You just need to ignore that**

**Moderator:** What of the place that you go for clinic, what are they doing that hinders you from managing your blood pressure?

**Respondent: I cannot see anything that is wrong there just that the pressure gets worse later like one week after you leave the clinic because of the issues that we find in the community. The doctor always tells me to try and manage my blood pressure coz he cannot find a reason as to why it rises yet it was ok the other day. I can say it is because of what I am going through and not the doctor**

**Moderator:** What about the government, what are they doing that hinders you from managing your blood pressure?

**Respondent: I don’t know what to say about that my sister because things are tough**

**Moderator:** Ok, you mentioned some challenges like you forgetting to take drugs and you also told me that you think a lot, what can you do differently to manage your blood pressure?

**Respondent: I told you that its only one hand that is providing**

**Moderator:** Yes

**Respondent: So sometimes I can go out and fail to get any casual job then you come back with lots of stress that you end up forgetting but if God can open a door that I will be waking up going somewhere for work then I will be very grateful**

**Moderator: We are about to finish**

**Respondent: Ok**

**Moderator:** How has COVID19 affected the way you get your hypertension care service in your community?

**Respondent: It has contributed a lot because sometime we could go and find that the doctor is not there and sometimes like two to three weeks ago, there is a time that we went but found out that there were no drugs though I had drugs at that**

**Moderator:** Ok, the last question now. Is there anything else that you feel we have not discussed about high blood pressure and you feel that we should discuss?

**Respondent: No, I think you have asked me everything but there is this question that I would like to ask you.**

**Moderator:** Ok

**Respondent: Can high blood pressure be transmitted from the family?**

**Moderator:** It would be better for you to confirm from the doctor who has been attending to you from 2011 or the one that is attending to you at health center to tell you and advice you what you can do to control your blood pressure and if you are not satisfied with what the doctor tells you, you can ask him again so that you can understand better. It will be hard for me because I have not been following up on you so I can’t know how the situation is with your family.

**Respondent: Ok. Thank you very much and may God bless you for attending to me**

**Moderator:** Thank you and have a good day

**Respondent: Thank you**

**…End…**
