# Supplementary material for: A socio-ecological framework examination of drivers of blood pressure control among patients with comorbidities and on treatment in two Nairobi slums; a qualitative study
Source: PLOS Glob Public Health. 2023 Mar 10;3(3):e0001625. doi: 10.1371/journal.pgph.0001625 (PMC10021823; doi:10.1371/journal.pgph.0001625)
Supplement: S1 File — (ZIP) [file pgph.0001625.s001.zip › Community/KOCH-IDI-UHTNC-200713_0305.docx]

**Moderator: {Name}**

**Code:** **KOCH-IDI-UHTNC-200713_0305**

**Moderator:** This community has been identified to have a high burden of uncontrolled hypertension which is a leading factor to premature deaths and disability. I am trying to gather information about hypertension care in your community. To avoid hypertension related complications, it is recommended that people with high blood pressure can change their lifestyles in regards to diet, physical activities, smoking, alcohol consumption and using blood pressure medication**.** So tell me about your experience with having high blood pressure**.** Tell me about your experience about your high blood pressure condition

**Respondent: For me, high blood pressure brings me down totally most of the time. It hurts my head that I cannot even do anything for myself that I fail to understand if there is any other thing apart from high blood pressure. It really pains me like for now I feel like my head is aching**

**Moderator:** For how long have you been having this high blood pressure condition?

**Respondent: For almost 16 years**

**Moderator:** How often do you check your blood pressure measurements?

**Respondent: I go for a clinic at {Name of the facility} where they check me; I also go to a city council hospital for checkup after every three days because my blood pressure condition rises high**

**Moderator:** So you told me that you go to {Name of the facility}and sometimes you go to a clinic located closer to where you stay

**Respondent: Yes. At {Name of a place}**

**Moderator:** Do you record you blood pressure measurement after you have been measured?

**Respondent: I don’t know how to read**

**Moderator:** Is there a person who writes for you?

**Respondent: They right for me and sometimes when it’s high I am told to drink water or sometimes am told to go to the hospital**

**Moderator:** Do you have any other condition apart from high blood pressure?

**Respondent: Yeah**

**Moderator:** What’s the other condition?

**Respondent: Cancer**

**Moderator:** Cancer?

**Respondent: Yes**

**Moderator:** Which type of cancer?

**Respondent: Stomach cancer**

**Moderator:** Have you ever been told your blood pressure targets when you go for clinics at { Name of a place} or {Name of a facility}?

**Respondent: They always tell me, it is me who don’t understand coz they do write for me on a paper but I don’t know how to read**

**Moderator:** Tell me about your drugs, how many tablets do you take?

**Respondent: I am using three types of tablets**

**Moderator:** Ok

**Respondent: Like for now I have a white, red and another one that is heart shaped**

**Moderator:** From the time that you were diagnosed, would you say that your tablets have been increasing or they have been reducing in number?

**Respondent: For those years that I have told you, there is a time when I used to take two tablets but I was later on added another tablet that I am taking together with my hypertension drugs**

**Moderator:** So you are now taking three

**Respondent: Yeah so I don’t know if my pressure is going higher**

**Moderator:** What did your doctor tell you when he added you that drug?

**Respondent: He told me that my blood pressure is not going down and when it goes down I am told that I should be admitted because it is very low**

**Moderator:** Are you telling me that there are times when your blood pressure is very high and there are other times that it is very low to an extend that you get admitted?

**Respondent: Yes**

**Moderator:** Ok, how has high blood pressure affected your life?

**Respondent: The situation is bad my daughter to be sincere. With this condition there are time when I cannot get money to buy drugs and my head aches when I don’t take drugs, there are other issues that it can cause you. This condition is really making things worse for me. One can die just because of thinking about a small issue**

**Moderator:** What else do you do to manage your blood pressure apart from taking drugs?

**Respondent: I take water**

**Moderator:** What about food?

**Respondent: You know the state of life in this community, we just eat what we get when you get**

**Moderator:** What about doing exercise?

**Respondent: I am very old**

**Moderator:** Ok **.**Who do you see when you go for your clinics at {Name of a place} or {Name of a facility}?

**Respondent: Doctor**

**Moderator:** Can you tell me how your doctor is managing your high blood pressure condition?

**Respondent: He puts something on my hand then there is another thing that he presses like that.**

**Moderator:** How is he managing your high blood pressure condition?

**Respondent: He attends to me because he tells me if my blood pressure is high or low after he checks me. That doctor does a very good job because he checks me, he advices me, he asks to know of what is disturbing me**

**Moderator:** Ok, sometimes you go to {Name of the facility}, sometimes you go to {Name of a place}. What made you stop going to {Name of a place} and started going to {Name of the facility}?

**Respondent: I started going to {Name of the facility} because I was diagnosed with cancer last year but one. It is like three years since I was diagnosed with cancer and so I go there on every Monday and they must take my measurement whenever I go there. The do all the tests even diabetes tests but its long since I was tested of diabetes**

**Moderator:** Are you diabetic?

**Respondent: I don’t know if I am diabetic**

**Moderator:** Ok, what were you told when you were tested for diabetes?

**Respondent: I was told that my sugar levels were a little bit high but I don’t know if I am diabetic because I don’t take diabetes drugs**

**Moderator:** What kind of services do you get when you go for hypertension clinic? You told me that they take your blood pressure measurements, what else they do?

**Respondent: I told you that they check my blood pressure measurements and if they find that my blood pressure is very high then they ask me to sit down somewhere then they break a tablet and give me to swallow, then I my pressure is measured again after some time then they talk to me on how am supposed to eat to manage my blood pressure, then they give me water**

**Moderator:** You said that you go to {Name of the facility} on every Monday

**Respondent: Yes**

**Moderator:** Is this clinic for cancer or high blood pressure?

**Respondent: It is for cancer**

**Moderator:** Ok

**Respondent: But they must check my blood pressure when I go for cancer clinic**

**Moderator:** Ok

**Respondent: On Mondays I go for cancer clinic and they must check my pressure when I go for cancer clinic**

**Moderator:** What are the individual factors that make you unable to manage your blood pressure?

**Respondent: Financial problems, I told you that sometimes there is no money my daughter**

**Moderator:** Financial problems?

**Respondent: Yes**

**Moderator:** What about buying drugs?

**Respondent: We buy our drugs; there is no to who gives us drugs for free**

**Moderator:** Ok

**Respondent: They just write for me on a paper for me to buy. Why don’t you just give us sometimes for free?**

**Moderator:** Ok. And you also said that you are not young?

**Respondent: I have more than 60 years**

**Moderator:** Ok, what about family problems that make you unable to control your blood pressure?

**Respondent: I have a daughter the one that talked to you**

**Moderator:** Yes

**Respondent: She is also not healthy, she has her conditions but she is the one taking care of me. That’s a challenge**

**Moderator:** What about your health care providers’ factors, what do you think that he is not doing to help you manage your blood pressure??

**Respondent: My doctor is a good one, he talks to me and advices me to stop thinking so much and he tell me on how I can manage my pressure even at {Name of a place}, there is no doctor that is bad. They all tell me all that.**

**Moderator: what of the facility clinic hours? Tell me about that**

**Respondent: They should adjust on their time; we really wait for long before we are served**

**Moderator:** What about the space for hypertension patients at the clinic?

**Respondent: What do you mean? I don’t know what you are talking about**

**Moderator:** I mean the space at the clinic, how is it?

**Respondent: The space is not bad, I don’t see if there is a problem with space**

**Moderator:** What about drugs? You said that sometimes the drugs are there sometimes they are not there. Kindly tell me more about medicine

**Respondent: I told you that there is no any single day that I went to the health Centre and I was given medicine**

**Moderator:** So you always buy all the drugs?

**Respondent: I buy them my daughter**

**Moderator:** I now understand

**Respondent: Ok**

**Moderator:** Now tell me about the government, what is it that you think they are doing that makes it hard for you to manage your blood pressure?

**Respondent: It would be better if the government ordered that high blood pressure medication be given for free but the government is not thinking about us. They are not giving us drugs for free. The government should think about cancer patients, those that are hypertensive and also the Diabetic ones**

**Moderator:** Ok

**Respondent: If they are giving HIV drugs for free then they should also give us drugs for free**

**Moderator: Ok, you have told me many things. What can you as an individual who is hypertensive do to manage your blood pressure?**

**Respondent: I do this; I go out and walk around. That’s what I do**

**Moderator:** Ok

**Respondent: Then I do some little chores just to keep me away from thinking. That what I do to see if my blood pressure can go down**

**Moderator:** You said that the doctors do tell you about everything and you don’t have any problem with them

**Respondent: I don’t have any problem with the doctors. There is no any doctor who has offended me in any way**

**Moderator:** And at the hospital you said that they take long to serve you, you also said that there are times when you are many patients at the facility and you also mentioned that there are no drugs at the facility, the doctor writes for you the drugs for you to go buy

**Respondent: I always buy**

**Moderator:** You also told me that you want the government to give you drugs for free

**Respondent:** Yes

**Moderator: And also to help cancer patients and those that are diabetic**

**Respondent:** Yes

**Moderator:** Ok, and for this Corona problem, how has it affected your hypertension care service delivery in your community?

**Respondent: People like us who are 60 years are being told to stay indoors, I don’t go for clinics, last month I was supposed to go to {Name of the facility} but I didn’t go am just here even today on Monday I have not gone, my daughter told me to go but I refused**

**Moderator:** So today you didn’t go

**Respondent: I refused to go because I was told to stay indoors coz of Corona and I also have the other condition, I’ll die faster and I am still in pain**

**Moderator:** Ok. Now the last question, what do you think that we have not mentioned about Corona and you would want us to talk about?

**Respondent: There is nothing else my daughter; it is like you have told me everything**

**Moderator: Ok, thank you for your time and I know that this information will help us and other people including you so that we can be able to formulate these rules that can help you**

**Respondent: Yes**

**Moderator: Thank you so much**

**Respondent: Welcome**

**…END…**
