# Supplementary material for: A socio-ecological framework examination of drivers of blood pressure control among patients with comorbidities and on treatment in two Nairobi slums; a qualitative study
Source: PLOS Glob Public Health. 2023 Mar 10;3(3):e0001625. doi: 10.1371/journal.pgph.0001625 (PMC10021823; doi:10.1371/journal.pgph.0001625)
Supplement: S1 File — (ZIP) [file pgph.0001625.s001.zip › Community/KOCH-IDI-UHTNC-200713_0720.docx]

**Moderator: {Name}**

**Code:** **KOCH-IDI-UHTNC-200713_0720**

**Moderator:** This community has been identified to have a high burden of uncontrolled hypertension which is a leading factor to premature deaths and disability. I am trying to gather information about hypertension care in your community. To avoid hypertension related complications, it is recommended that people with high blood pressure can change their lifestyles in regards to diet, physical activities, smoking, alcohol consumption and using blood pressure medication**.**So tell me about your experience with having high blood pressure**.**Tell me about your experience with having high blood pressure

**Respondent: One can be shocked and also feel very bad if somebody tells you some things. It affects one more if he or she does not take medicine**

**Moderator:** For how long have you been having high blood pressure?

**Respondent: From 2017**

**Moderator:** How often do you go to check your blood pressure?

**Respondent: I go once in every month**

**Moderator:** Where do you go to check your blood pressure?

**Respondent: At the health center**

**Moderator:** Do you record your blood pressure readings after you have been checked?

**Respondent: Yeah, I have a book that I record in**

**Moderator:** What was your last blood pressure measurement?

**Respondent: The last reading was 230/94**

**Moderator:** Over what?

**Respondent: Let me check my record shortly**

**Moderator:** As she brings the book, I remember that you told me that you are also diabetic

**Respondent: Yes**

**Moderator:** When did you become diabetic?

**Respondent: It was the same time during the 2007 post-election violence that shocked me and after the violence I went to the hospital where I was told that I am both hypertensive and diabetic**

**Moderator:** Both conditions at the same time?

**Respondent Yes, it was at the same time:**

**Moderator:** You told me that you attend your clinic at the Health center

**Respondent: Yes**

**Moderator:** And they check your pressure there

**Respondent: Yes**

**Moderator:** Has your doctor ever told you what your target blood pressure should be?

**Respondent: He told me that it is supposed to be 120/80 for pressure and for diabetes it is supposed to be 5 but shouldn’t go beyond 10**

**Moderator:** Tell me about your hypertensive drugs, how many tablets have you been taking?

**Respondent: I take a tablet called nepheprine, Enaraprine and sometimes I get Euriprine (6:16-6:18). I take those ones**

**Moderator:** So you take three tablets

**Respondent: Yes**

**Moderator:** You said that you have been taking antihypertensive from when you were diagnosed in 2017 up to now, have your drugs been increasing in number or they have been reducing in number?

**Respondent: They increase in number**

**Moderator**: What reason did the doctor give you that made him add the number of your drugs?

**Respondent: He adds after measuring my blood pressure and finds that it is high**

**Moderator:** Has your doctor ever told you that he is increasing the strength of the drugs as the adds the number of your drugs?

**Respondent: Yeah, mostly for diabetes because I inject myself so he adds me the number of drugs that am supposed to inject myself**

**Moderator:** Ok. And how has high blood pressure affected your life?

**Respondent: It is worse when blood pressure is high like my last reading that was 205/ 110. I was given medicine and told to wait for one hour then I go back for my pressure to be measured again**

**Moderator:** Ok

**Respondent: When I went back they found that it was 148/84**

**Moderator:** Ok

**Respondent: The doctor just prescribed drugs that I came home with**

**Moderator:** Apart from taking antihypertensive drugs, what else do you do to manage your blood pressure?

**Respondent: I just do exercise like walking fast for a long distance and when I do that my heart beats louder. Just exercise**

**Moderator:** What about food?

**Respondent: I take vegetables and fruits. I was told to take traditional vegetables, a lot of water and fruits. I was also told not to take a lot of salt and sugar, I should eat brown ugali, I am not supposed to eat meat that has a lot of fat and I also don’t eat the skin of a chicken coz the doctor said that it had a lot of fat, soda. Things like that**

**Moderator:** Ok. Have you been doing as instructed?

**Respondent: Yes, I try so hard**

**Moderator:** Who do you see when you go to the health center for you clinics?

**Respondent: Doctors are there that do blood pressure measurement and diabetes tests**

**Moderator:** What can you say about those doctors in regards to the way they are managing your blood pressure

**Respondent**: **They manage my pressure very well but the problem is my blood pressure that akways goes high and most of the times they cannot tell the reason as to why my blood pressure goes high**

**Moderator:** Ooohh

**Respondent: They always ask me what the problem is, if I take foods that I am not supposed to or if there is anything that I don’t do but for me I always do what they instruct me to do**

**Moderator:** Have you sought care elsewhere apart from the Health center

**Respondent: I go to {Name of the facility} for my major clinic after like 3 years. I went there last year and I was told to go back in 2022. My major clinic is at {Name of the facility} but I go to the health Centre for my normal clinics**

**Moderator:** What do they tell you when you go to {Name of the facility}?

**Respondent: They also fail to understand why my blood pressure is always high. They always tell me that its either I take foods that am not supposed to take or maybe I have so many thoughts or I don’t take drugs as am supposed to**

**Moderator:** Where do you stay currently?

**Respondent: I stay in {Name of a place}**

**Moderator:** So you get all your hypertension care services at the health center

**Respondent: Yes, I go there**

**Moderator:** Tell me about the services that you get there

**Respondent: The services are ok the bad thing is that sometimes we don’t get drugs when we go there. I didn’t get drugs the last time I went there and I was told to go buy but sometimes i find that i don’t have money to buy drugs, I just buy a little flour so that my kids can eat ugali. It is hard to choose whether to buy drugs or food for my kids. That’s the challenge that I face**

**Moderator:** You told me about the lack of money and sometimes you are told to buy drugs.

**Respondent: Yes**

**Moderator:** Is there any other problem that might hinder you from managing your blood pressure?

**Respondent: Just lack of drugs**

**Moderator:** What are the individual factors that might hinder you from managing your blood pressure?

**Respondent: I think the problem is the life style. The kind of life that we are living whereby we have to think of what our kids will eat and how the kids will live. Just thoughts**

**Moderator:** What about you taking drugs at the time that you are supposed to take them

**Respondent: With drugs I really try but the problem comes when you don’t have the drugs. I can miss drugs today and take them tomorrow, that challenge is also there**

**Moderator:** What about family or communal factors? You told me that sometimes you are stressed about what kids will eat,

**Respondent: Yes**

**Moderator:** What are other communal factors that might hinder you from managing your blood pressure?

**Respondent: With this current life style you can’t get enough but ewe buy drugs with the little we get. The current lifestyle with kids**

**Moderator:** Ok. Looking at your doctor, what do you think that he is not doing that is hindering you from managing your blood pressure?

**Respondent: He is trying but the problem comes when there are no drugs. In that case the doctor just writes for you the drugs that you are supposed to take and when you go for the drugs you are told that the drugs are not available so we are forced to go look for money to buy drugs but sometimes we don’t have the money**

**Moderator:** What about the time that you take at the facility for you to be treated? How lng do you take?

**Respondent: At the hospital?**

**Moderator:** Yes

**Respondent: We take like 4 hours. We go there in the morning but at this time they don’t want a big number of people due to Corona and if you don’t go early then you will not get drugs so we are forced to go there as early as 7. They serve a small number of people; by to they are done with treating**

**Moderator:** What about the government, what do you think that they are not doing? You told me that there are times when you don’t get drugs

**Respondent: Yes**

**Moderator:** What else do you think they are doing that hinders you from managing your blood pressure?

**Respondent: Just drugs and thinking a lot sometimes when you don’t have something to eat and you want to take drugs, sometimes you don’t have the drugs that you are supposed to take. Those are the challenges but at the hospital they are really trying to do what they can. There is a time when there was only one doctor but the other day I found that the doctors are two so I don’t know if they will maintain the two doctors or they will go back to using one doctor. We have only been having one doctor for long**

**Moderator:** What can we do to solve this like for example your case, you told me that sometimes you don’t take drugs, sometimes you forget or you find that there are no drugs at the hospital. Tell me what you can do as an individual to manage your blood pressure

**Respondent: What I can do to control blood pressure is the same problem that I told you about which is lack of money to buy drugs so I don’t know what I can do so that I don’t lack money to buy drugs or I don’t miss drugs when I go to the hospital and how I can do for me not to lack flour so that my kids can get something to eat coz I have to manage this alone now that I don’t have a husband**

**Moderator:** What is it that can be done differently at the hospital?

**Respondent: just making sure that drugs are available so that we don’t miss drugs when we go there. We should get drugs first then our task just becomes swallowing the drugs. The bad thing is that we don’t get drugs when we go there. They tell us to go buy and that becomes a problem when we don’t have money to buy the drugs**

**Moderator:** Ok, you also told me that sometimes there is only one doctor at the facility and your number is big that the doctor finds it hard to attend to you. What can we do to your health care providers at the hospital?

**Respondent: Maybe adding the number of doctors because there is only one doctor there and the other thing is ensuring that we always get drugs. We should be getting all the drugs, come with them home then we just take the drugs**

**Moderator: Ok, we are about to finish**

**Respondent: Ok**

**Moderator:** How has COVID 19 affected service delivery for hypertension patients in your community?

**Respondent: for us we just go to the hospital when our booking date comes. There is no any day that I have not gone to the hospital because of Corona. The doctor normally writes for us our clinic dates an when the date come we just go to the hospital but now we have to go very early because they don’t want many patients at the hospital**

**Moderator:** Ok, the last question, is there any other thing that you feel that we have not talked about in regards to high blood pressure that you feel that we should talk about it**?**

**Respondent: What I can say is that the drugs be availed and people to get food because we don’t eat all sorts of food, we only eat foots that we are recommended to eat but now getting money becomes a problem which make you have a lot of thoughts. The doctor tells us not to think but as a normal human being you cannot avoid thinking. You must think because you have kids, you have parents that depend on you yet you can’t help yourself. That’s the problem that makes our blood pressure to be high**

**Moderator:** Ok, we are done. Thank you so much for your time and the information that you have shared with me. I hope that we will use the information to make other people know the problems that you go through as hypertensive patients. Thank you so much.
