# Supplementary material for: A socio-ecological framework examination of drivers of blood pressure control among patients with comorbidities and on treatment in two Nairobi slums; a qualitative study
Source: PLOS Glob Public Health. 2023 Mar 10;3(3):e0001625. doi: 10.1371/journal.pgph.0001625 (PMC10021823; doi:10.1371/journal.pgph.0001625)
Supplement: S1 File — (ZIP) [file pgph.0001625.s001.zip › Community/KOCH-IDI-UHTNC-200720_001.docx]

**Moderator: {Name}**

**Code:** **KOCH-IDI-UHTNC-200720_001**

**Moderator:** This community has been identified to have a high burden of uncontrolled hypertension which is a leading factor to premature deaths and disability. I am trying to gather information about hypertension care in your community. To avoid hypertension related complications, it is recommended that people with high blood pressure can change their lifestyles in regards to diet, physical activities, smoking, alcohol consumption and using blood pressure medication. So tell me about your experience with having high blood pressure. Tell me about your experience with having high blood pressure

**Respondent: I am at a very critical stage in that I am not supposed to be angry or be told any shocking news. Getting drugs that can help me manage this condition has been a challenge**

**Moderator:** How often do you check your blood pressure?

**Respondent: I used to test after every two weeks before corona came but I have not tested again from the time corona cases were reported in Kenya**

**Moderator:** Where do you check?

**Respondent: There is a health facility**

**Moderator:** What’s the name of the facility?

**Respondent: {Name of the facility}**

**Moderator:** Do you keep record of your blood pressure measurements?

**Respondent: Yes I do but I don’t know where I kept my book. They write for us in a book and we come home with the books**

**Moderator:** Do you remember the measurements for the last check up?

**Respondent: It’s been long. I have forgotten**

**Moderator:** Was it ok or it was high?

**Respondent: It was getting better not like before when it was 180. It was getting better just that I can’t remember the last measurement**

**Moderator:** Has your doctor told you what your normal blood pressure target should be?

**Respondent: He told me that it is supposed to be around 135**

**Moderator:** Apart from high blood pressure, do you have any other condition?

**Respondent: I was told that I am asthmatic**

**Moderator:** How has have you been affected by this condition?

**Respondent: I have been affected to an extend that I can’t correct my kids as a parent because the blood pressure rises when you talk to them and they don’t listen and I also cannot do my work faster. That is how I have been affected**

**Moderator:** Can you tell me about your antihypertensive drugs like how do you take them and how many types do you have?

**Respondent: I was told to take one tablet per day**

**Moderator:** Do you know the name of the drug?

**Respondent: I don’t know madam but it’s a small white tablet**

**Moderator:** For how long have you been having high blood pressure?

**Respondent: From 2014**

**Moderator:** Is it the same tablet that you started with when you were diagnosed or it was changed?

**Respondent: It’s the same though there are times when I an given the yellow ones but my head aches when I take them but I don’t feel any headache when I take the white ones**

**Moderator**: Have you been using one tablet from the time you were diagnosed with high blood pressure?

**Respondent: The doctors say that it is getting better when I go for monthly check up but when we don’t find drugs then the pressure rises again by the time we go for the other clinic. That’s the problem**

**Moderator:** But the tablet has been constant?

**Respondent: Yes**

**Moderator:** Apart from using drugs, how else do you manage your blood pressure condition?

**Respondent: I try so hard to avoid things that can make me angry even though it is hard to manage**

**Moderator:** What about diet?

**Respondent: We were told to take a well-balanced diet but getting money is also a problem. We’ve been told to eat traditional vegetables in plenty but getting them is also a problem and if you find them the price I s always high so we end up eating the sukuma that we are advised not to eat. We just take what is available**

**Moderator:** What about exercise?

**Respondent: We have been told to do exercise and not just sit**

**Moderator:** Do you do the exercise?

**Respondent: Yes, I do but not as much**

**Moderator:** Have you ever used traditional medicines

**Respondent: Yes**

**Moderator:** Are you still using them?

**Respondent: It has been a while since I took them. Am just there, there is no medicine that I am using**

**Moderator:** Who do you see when you go to the health center for clinic?

**Respondent: We are always referred to doctor there**

**Moderator:** What can you say in regards to the way your doctor attends to you?

**Respondent: I have never seen anything that is wrong with him because he serves us well and he refers us to other people who give us advice on balance diet but it’s hard to eat as we are told because life is hard that we are forced to repeat the same meal but according to the doctor, we are old that we a are supposed to be changing meals, take fruits and eat vegetables in plenty**

**Moderator:** Have you sought treatment elsewhere apart from the Health Center?

**Respondent: No, I have just been going to this health center**

**Moderator:** What services do you receive when you go to the Health Center?

**Respondent: The services are not bad. They invite us well and tell us to sit and wait because everyone has his or her date as booked by the doctor. From there you go to the doctor who checks your blood pressure measurements and he directs you to go and collect the drugs**

**Moderator:** Do you buy your drugs or you get them for free?

**Respondent: We don’t buy drugs at the facility but sometimes we do buy from catholic fathers when they are not available at the facility**

**Moderator:** You told me that you used to go for blood pressure checkup after every two weeks

**Respondent: Yes**

**Moderator:** How often do you go for clinic?

**Respondent: The doctor just books a date and writes for us in the book**

**Moderator:** Do you have any problem in managing your blood pressure? Looking at you as an individual, you told me that you have financial problems and that’s why you can’t buy what you are advised to eat

**Respondent: Yes**

**Moderator:** Do you have any other thing that you can add to that?

**Respondent: No but if I had money then I would eat as advised but now that I don’t have money then I just cannot afford to eat as advised**

**Moderator:** Do you have insurance?

**Respondent: I don’t have yet**

**Moderator:** Looking at your age, is it a challenge in managing your blood pressure?

**Respondent: My age was ok when I was diagnosed in 2014 coz I had 50 something years then but it is becoming worse as the age increases. I am 60 years now and that’s why I keep of forgetting somethings. Thing are not doing fine**

**Moderator:** You also told me that you are asthmatic

**Respondent: Yes**

**Moderator:** Is it hindering you from managing your blood pressure?

**Respondent: I don’t know**

**Moderator:** What about the way you take your drugs? You told me that you are supposed to take one tablet daily

**Respondent: Yes**

**Moderator:** Is that hindering you from managing your blood pressure?

**Respondent: I don’t think but just like I told you there are times when we are given the yellow tablets that always affect me by causing me headache. I don’t have a problem with the white tablets**

**Moderator:** Do you use either alcohol or cigarettes?

**Respondent: No**

**Moderator:** How is your normal day, do you leave the house?

**Respondent: For now we are not leaving home, we just go to the shop and back**

**Moderator:** Looking from your family side, is there any hindrance in managing your blood pressure?

**Respondent: They can’t help me because they don’t have the ability**

**Moderator:** Looking at the environment around you, could there be any foods that are not quality and might hinder you from managing your blood pressure?

**Respondent: Lack of a good diet is also a contributing factor**

**Moderator:** Looking at your health care provider, does he have a problem in managing your blood pressure?

**Respondent: I have not seen any problem maybe when I go and find that drugs are not available but I am always given drugs whenever they are available and I am told to go get them from a chemist when they are not available**

**Moderator:** Are the clinic hours ok?

**Respondent: Yes, we are told to go there in the morning at 8:00 am and I don’t stay far from the facility and if you find that there another people who came before you then you que and wait to be served**

**Moderator:** How is the quality of care in that facility?

**Respondent: It’s not bad**

**Moderator:** What about advice?

**Respondent: I don’t have a problem with the advice that we get on diet but the challenge only come when you can’t get money to buy the food**

**Moderator:** Looking at the government policies, do they cause any hindrance in managing your blood pressure?

**Respondent: I don’t know if the problem is from the government or it’s just the health care providers who don’t want to serve us well**

**Moderator:** What can be the solution to the hindrances that you mentioned? You had talked about several challenges and I’ll read them for you

**Respondent: Ok**

**Moderator:** We talked about finances where you said that there are times that you don’t get drugs from that facility and you are but them and also you talked about getting money to buy food. What would be the solution to that?

**Respondent: The solution would be finding ways in which we can be getting money so that we can be able to get drugs and food easily. If you have money then you will be able to eat as the doctor wants you to eat and you will be able to get drugs**

**Moderator:** We also talked about age where you said that age is a contributing factor and it makes you to forget

**Respondent: Yes**

**Moderator:** What would be the solution to that?

**Respondent: Maybe if the doctor can tell me if there is a drug that I can take at my age that can help me not to forget or could it be the hypertensive condition that causes forgetfulness. I need specialists to help me on that**

**Moderator**: We also talked about your drugs whereby you said that there is a drug that has side effects on you

**Respondent: Yes**

**Moderator:** And there is one that is ok. What do you think would be the solution to that?

**Respondent: They are two tablets, a yellow one and a white one. It would be better if I the white one would be easily available on time**

**Moderator:** We also talked about the family. Sometimes your children can cause you problems that can lead to stress. What would be the solution to that?

**Respondent: it is hard because you cannot get rid of them. They are our kids and we stay with them. I don’t know what we can do maybe if I can get a doctor to inform them that I am not supposed to be angered because kids don’t understand what you are feeling. It would be better if I could get someone to advise them so that they can stop causing me anger**

**Moderator:** What about diet? You said that you can’t get good diet at the place where you stay. What would be the solution to that?

**Respondent: I can get the traditional vegetables that I am advised to eat by the problem is money. It would be easier if it was at my rural home where I could just get them from the garden. These vegetables are more expensive compared to 1kg of meat**

**Moderator:** Looking at drugs, you said that sometimes they are not available at the hospital, what would be the solution to that?

**Respondent: It would be better if the government could help us get drugs easily**

**Moderator:** Looking at the government

**Respondent: Yes**

**Moderator:** What would be the solution?

**Respondent: I said that it would be better if they could help us get drugs easily. They should treat us the same way they treat those patients with other diseases like HIV because it is very hard for HIV people to miss drugs**

**Moderator:** How has COVID19 situation affected how you get hypertension care in this community? You told me that you have not gone to your health facility since from the day cases of COVID were identified.

**Respondent: Yes**

**Moderator:** How has that affected you?

**Respondent: It has really caused me a hard time because my blood pressure keeps on increasing because sometimes we even fear going to the clinic because of fear that you can meet other people. We were told to avoid getting close to people especially people of my age of 60 years. That is the age that is mostly affected by COVID. That’s a challenge because we are told to stay at home not knowing what you will eat or drink. That’s the challenge**

**Moderator:** Is there anything else that you would want us to talk about in regards to hypertension?

**Respondent: I have nothing else apart from what I mentioned about getting drugs easily**

**Moderator:** Ok. Thank you for the time that you have given me and the conversation that we have had. I know this will help us in our research. Thank you very much and have a good day

**Respondent: You too**

**…END…**
