# Supplementary material for: A socio-ecological framework examination of drivers of blood pressure control among patients with comorbidities and on treatment in two Nairobi slums; a qualitative study
Source: PLOS Glob Public Health. 2023 Mar 10;3(3):e0001625. doi: 10.1371/journal.pgph.0001625 (PMC10021823; doi:10.1371/journal.pgph.0001625)
Supplement: S1 File — (ZIP) [file pgph.0001625.s001.zip › Community/KOCH-IDI-UHTNC-200720_002.docx]

**Moderator: {Name}**

**Code: KOCH-IDI-UHTNC-200720_002**

**Moderator:** This community has been identified to have a high burden of uncontrolled hypertension which is a leading factor to premature deaths and disability. I am trying to gather information about hypertension care in your community. To avoid hypertension related complications, it is recommended that people with high blood pressure can change their lifestyles in regards to diet, physical activities, smoking, alcohol consumption and using blood pressure medication**.**So tell me about your experience with having high blood pressure**.**Tell me about your experience with having high blood pressure

**Respondent: For me what am trying to avoid is that I don’t smoke cigarettes, I don’t take alcohol and I eat food as advised by the doctor**

**Moderator:** For how long have you been having high blood pressure?

**Respondent: Since 2007**

**Moderator:** So let’s say that it is like for the last 13 years

**Respondent: Yes**

**Moderator:** How often do you check your blood pressure measurements?

**Respondent: Once in a month**

**Moderator:** Where do you check?

**Respondent: At the Health Center**

**Moderator:** Do you record your blood pressure measurements?

**Respondent: Yes I have a book**

**Moderator:** What were the measurements the last time you checked?

**Respondent: It was not ok**

**Moderator:** Can you remember the number?

**Respondent: Let me check in my book**

**Moderator:** Ok.

**Respondent:** **It was 185/90 and it was in February**

**Moderator:** Ok, do you have any other condition apart from high blood pressure?

**Respondent: Yes, am diabetic**

**Moderator:** Has your doctor told you your target blood pressure measurement?

**Respondent: Yes**

**Moderator:** What did he tell you?

**Respondent: My BP should be 120/80**

**Moderator:** Ok, tell me about the antihypertensive drugs that you are using

**Respondent: For blood pressure I use one tablet enapryn 10mg per day**

**Moderator:** You are using only one tablet for blood pressure?

**Respondent: Yes**

**Moderator: Which drug did you start with when you were diagnosed with hypertension 13 years ago? Can you remember? Or you have been using the same drug**

**Respondent: I was calysprene (4:46)**

**Moderator: When did you start taking enapryne?**

**Respondent: Here is where I am given that …5:00-5:02… (Not clear)**

**Moderator:** I am asking, how long you been using enapryne

**Respondent: I have used it for the past two years now**

**Moderator:** Has your dosage changed from the time you started using it?

**Respondent: Yes, I have been taking the same**

**Moderator:** How has high blood pressure affected you?

**Respondent: It has affected be on drugs, sometimes I don’t have money and I cannot get the drugs at the public hospital**

**Moderator:** Apart from using medicine, how else do you manage your blood pressure?

**Respondent: I avoid many thoughts**

**Moderator:** You had also told me that you watch your diet

**Respondent: Yes**

**Moderator:** How do you watch your diet?

**Respondent: I watch my diet coz of diabetes**

**Moderator:** What about exercising?

**Respondent: Exercising is obvious, I must walk everyday**

**Moderator:** Have you ever used traditional medicine?

**Respondent: No, I don’t like traditional medicine**

**Moderator:** What else do you do to manage you high blood pressure condition?

**Respondent: Just walking and ensuring that I use medicine**

**Moderator:** Who do you see when you go to the health center?

**Respondent: Doctor**

**Moderator:** What can you say in regards to the way your health care provider is managing your blood pressure?

**Respondent: He serves us well**

**Moderator:** Have you ever sought care elsewhere?

**Respondent: Some times when I don’t get drugs there I usually go to {Name of the hospital}**

**Moderator:** What do they tell you there in regards to blood pressure?

**Respondent: They give me drugs that they have and write down for me to buy the ones that they don’t have**

**Moderator:** Which services do you receive when you go to the health facility?

**Respondent: Blood pressure measurement, Diabetes checks and they also advise me**

**Moderator:** Ok, You told me that you go to the hospital on a monthly basis

**Respondent: Yes. I must go to collect medicine every month**

**Moderator:** Do you have any problems in managing your blood pressure? Looking at you as an individual

**Respondent: Yes, I have a problem. Getting drugs is the main problem**

**Moderator:** Do you have insurance?

**Respondent: No I don’t have**

**Moderator:** So you have to buy drugs when you don’t get them at the hospital

**Respondent: Yes and if I don’t have money then I don’t take the drugs**

**Moderator:** Looking at your age, do you think that it’s a hindrance in managing your blood pressure?

**Respondent: Age is not a problem**

**Moderator:** You told me that you are also diabetic. Is that condition a hindrance in managing your blood pressure condition?

**Respondent: No**

**Moderator:** Looking at the way you take medication, you have antihypertensive, you have diabetes drugs, do you see any problem with that?

**Respondent: I just use the all together**

**Moderator:** You have told me that you normally walk

**Respondent:** Yes, and I also sell water so mostly I do walk

**Moderator: Looking at you family and the community, are they a hindrance in managing your blood pressure?**

**Respondent:** No, they are not stressing me

**Moderator: What of the food that is close to you that is not good? Do you use?**

**Respondent: No**

**Moderator:** Like potatoes

**Respondent: I don’t use**

**Moderator:** What of your health care provider at the health Centre, do you see like he is a hindrance in managing your blood pressure?

**Respondent: No. He has no problem**

**Moderator:** How is their quality oh treatment?

**Respondent: It is good that they give me drugs when they have and when they don’t have they do write for me to go buy and when they tell me to buy that when I go to {Name of the hospital}**

**Moderator:** Looking at the hospital, you told me that sometimes you don’t find drugs

**Respondent: Yes**

**Moderator:** Looking at the facility the way it is constructed, Is it enough for you?

**Respondent: Yea, it’s enough for us**

**Moderator:** If we look at the policy makers and guideline, do you see like there is any problem in regards to monitoring your blood pressure?

**Respondent: Do they have? We are normally given drugs and we are also advised on how we can live**

**Moderator:** Ok. What do you think can be the solution to the hindrances that you mentioned? You had told me many hindrances before so I’ll be mentioning one by one. On individual hindrances you told me that there is a problem on finance

**Respondent: Yes**

**Moderator:** What’s the solution for that?

**Respondent: Am disabled yet I don’t receive the money that the government released for the disabled**

**Moderator:** So what would be the solution to that?

**Respondent:** The government should take care of us coz personally I don’t have a job

**Moderator: You also said that sometimes you don’t have drugs at the hospital**

**Respondent:** Yes

**Moderator:** What would be the solution to that?

**Respondent: The government should provide drugs**

**Moderator:** We also looked at policies and you said that there are hindrances there, what would be the solution to that?

**Respondent: I don’t understand**

**Moderator:** You told me some issues on policy makers that hinder you from managing your blood pressure. What would be the solution to that?

**Respondent: They should bring us medicine to the hospital so that we are not told to go buy**

**Moderator:** How has COVID 19 affected delivery of hypertension care services in this community?

**Respondent: We don’t see the doctor when we go to the hospital; we just pick drugs and go back home. Like for my case you can see that the last time I was checked pressure was in February. We don’t even talk to the doctor; we just go to the pharmacy**

**Moderator:** Is there anything else that you would like us to talk in regards to pressure?

**Respondent: Just the drugs so that we don’t have stress. Stress contributes to that**

**Moderator:** Ok, thank you for your time and the discussion that we have had. I know that it will be of help to our study. Thank you very much

**Respondent: Thank you**

**…END…**
