# Supplementary material for: A socio-ecological framework examination of drivers of blood pressure control among patients with comorbidities and on treatment in two Nairobi slums; a qualitative study
Source: PLOS Glob Public Health. 2023 Mar 10;3(3):e0001625. doi: 10.1371/journal.pgph.0001625 (PMC10021823; doi:10.1371/journal.pgph.0001625)
Supplement: S1 File — (ZIP) [file pgph.0001625.s001.zip › Community/VIWA_IDI_UHTNC_200715_006.docx]

**Moderator: {Name}**

**Code: VIWA_IDI_UHTNC_200715_006**

**Moderator:** This community has been identified to have a high burden of uncontrolled hypertension which is a leading factor to premature deaths and disability. I am trying to gather information about hypertension care in your community. To avoid hypertension related complications, it is recommended that people with high blood pressure can change their lifestyles in regards to diet, physical activities, smoking, alcohol consumption and using blood pressure medication**.**So tell me about your experience with having high blood pressure**.**Tell me about your experience with having high blood pressure

**Respondent: Am not getting you**

**Moderator:** For how long have you been having high blood pressure?

**Respondent: For like 18 years coz the child that I gave birth to while having high blood pressure is 15 years now**

**Moderator:** How often do you check your blood pressure?

**Respondent: I check on a monthly basis**

**Moderator:** Where do you go to check?

**Respondent: I used to go {Name of the hospital} but since they closed the hospital nowadays I go for checkup at {Nam of the facility}**

**Moderator:** Ok, do you record your blood pressure measurements?

**Respondent: Yeah, I have a book**

**Moderator:** What was the reading the last time your blood pressure was measured?

**Respondent: I am not close to my book and I cannot remember**

**Moderator:** Were you told that your pressure was high or low

**Respondent: I was told that it was high but not very high**

**Moderator:** Ok, do you have any other condition apart from high blood pressure?

**Respondent: Yes, I have ulcers**

**Moderator:** Has your health care provider told you what your target blood pressure should be?

**Respondent: He just checks my measurements and tells me that it should not go beyond 120**

**Moderator:** Tell me about the drugs that you are using

**Respondent: I used to take two types but for now am only using one**

**Moderator:** You told me that you have been having this condition for 18 years, did you start taking these two types back then?

**Respondent: At first it was nerdin (4:15 not clear). Actually I started with four types of drugs then changed to two types and am now using using one**

**Moderator:** How do you take that one type that you are taking?

**Respondent: I take it once in the morning**

**Moderator:** How was your blood pressure when you started taking one tablet?

**Respondent: It is ok coz nowadays am not admitted when it high like before when I could be admitted for like two weeks in a ward because of high blood pressure. Nowadays it’s like I have known how to manage it**

**Moderator:** How has high blood pressure affected you?

**Respondent: It has affected me because I cannot go to work when my blood pressure is high; sometimes I am unable to walk and it also gets worse when I hear of any bad news about my family but through counseling I am able to manage to an extend that my blood pressure doesn’t rise even if I get any bad news**

**Moderator:** How are you managing your blood pressure apart from using drugs? You told me that you are currently using one type so apart from drugs, how else do you manage your blood pressure?

**Respondent: There are some things that I am not using**

**Moderator:** Like what?

**Respondent: I don’t use tea leaves,**

**Moderator:** What else?

**Respondent: There are many thing that I don’t use, do you know that there are other things that I am not using because of ulcers? I don’t take tomatoes, beans, red meat but I am allowed take white meat. I don’t take soda, food spices. My blood pressure went down when I stopped using all those things that I was told not to take**

**Moderator:** For now what do you take?

**Respondent: I take yellow beans, ugali, traditional vegetables, I don’t take sukuma wiki but I eat managu, terere and spinach**

**Moderator:** What about exercise?

**Respondent: I do exercise because I walk as I was told. I try to walk and avoid using matatu to {Name of a place}; there are times that I walk to {Name of a place} because my weight was not good and I was advised to reduce. I had started developing diabetes symptom but when I started working on my weight then the symptoms disappeared**

**Moderator:** Have you ever used traditional medicine

**Respondent: No, I have never**

**Moderator:** Who do you see when you go to the hospital?

**Respondent: I go direct to the doctor**

**Moderator:** What can you say in regards to way your health care provider is managing your blood pressure?

**Respondent: I am even thinking of changing the facility because the always ask me to pay for my blood pressure checkup when I go there with other conditions like malaria. I am thinking of changing because I don’t have that money. I use my NHIF card and I think my current hospital is not like {Name of the facility} coz at they used to advise me on what to eat unlike my current hospital where they don’t tell me**

**Moderator:** What were you told at {Name of the facility} in regard to blood pressure?

**Respondent: They used to do follow ups on me in regards to diet, exercise, and they could call to follow up whenever I missed my clinics and with my current hospital they don’t. I went for the other clinic and missed the clinic that I was supposed to attend last and they have not called me to know what happened though I went and bough my drugs elsewhere. It’s like they don’t follow up on their clients**

**Moderator:** What services do you receive as hypertensive patients when you go to that facility?

**Respondent: They don’t give me anything other than telling me to do exercise like at shalom where they could refer me to a different doctor when they find out that my blood pressure is high but there is no referral at my current facility, I am attended by the same doctor every time I go there**

**Moderator:** What of doing blood pressure measurement?

**Respondent: They do measure**

**Moderator:** Do you pay for drugs or they are sorted by the insurance?

**Respondent: We are told to add some money for us to get drugs and sometimes you don’t have money**

**Moderator:** Were you paying at {Name of the facility}?

**Respondent: I have never paid at {Name of the facility} apart from when I was supposed to so a scan in relation to ulcers but they used attend to me when it comes to other services**

**Moderator:** You told me that you normally go for clinic on a monthly basis

**Respondent: Yes**

**Moderator:** Are there any individual factors hindering you from managing your blood pressure? You told me that you have insurance and insurance could cover for everything at Shalom Hospital unlike at the current hospital where you have to buy some tablets

**Respondent: Yeah, they tell you to buy like for example this month I was told to add 800 shillings that I didn’t have. I told them to attend to the problem that I had and went home to find how I can attend to high blood pressure and I spent less than the 800 shillings on the drugs that I went to buy them at went to {Name of the house} because I know the drugs**

**Moderator:** Looking at your age, is it a hindrance in managing your blood pressure?

**Respondent: My husband has cancer and it’s like in the final stages, he can ask me to cook something and then hit me with what I serve him yet he is the one that asked for it, I think he is the one contributing to that as I based on how I was told at {Name of the facility} because that shocks me and causes me a lot of stress**

**Moderator:** You also told me that you have ulcers; do you think that it could be a hindrance in managing your blood pressure?

**Respondent: Yes, I have ulcers; there are a time when I vomit blood and that is why I was asked to do MRA at {Name of the facility} for them to see how the condition is**

**Moderator:** Is it a hindrance in managing your blood pressure?

**Respondent: Yeah, I think so**

**Moderator:** What about the way you take your drugs, could there be a hindrance there?

**Respondent: It is ok taking drugs but there are times that I have to be injected when I think so much and my blood pressure goes up and the tablets fail to work and I faint or fall down**

**Moderator:** How is your normal day, do you go out of the house?

**Respondent: I go out when am ok but not when my blood pressure is high or when I have ulcer pains like for now am just indoors coz of ulcers**

**Moderator:** You mentioned that from your family you think that your husband is causing you problem because of the condition that he has

**Respondent: Yeah, he has cancer and it’s like it is in the last stage because sometimes I give him food and he hits me with it and he also shouts at me when I ask him a question that might even be of help to him and this makes me think**

**Moderator:** What about food that are not good like for example chips or any other foods, do you take that?

**Respondent: I am forced to take especially for now that my business has gone down and I also cannot go without food because ulcers get worse. I just eat what is available I don’t have money**

**Moderator:** Looking at your health care providers, are they a hindrance in managing your blood pressure? You had said that you don’t get advice at the hospital that you go for clinic

**Respondent: yea, it’s not like the hospital that I used to go before, not even like St, Mary’s coz I used to go to {Name of the hospital} before but I changed to {Name of the facility} when {Name of the hospital} became bad and then {Name of the facility} was closed after it was found to be having Corona cases and that’s why I changed and started going to {Name of the facility} but I think of going back to {Name of the facility} because they have re-opened**

**Moderator:** You mean that the quality of service is not good at the facility that you are attending?

**Respondent: They just followed up on the drugs that I told them**

**Moderator:** Looking at the facility itself, do you get drugs when you go there?

**Respondent: I told you that when I go there with other condition like cancer, they only treat one and ask me to pay for the other one**

**Moderator:** Do you get all high blood pressure drugs there?

**Respondent: You only get them when you pay**

**Moderator:** Looking at the government policy, are they hindering you in managing your blood pressure?

**Respondent: They should be helping us. I heard that they help but I have never received any help from the government**

**Moderator:** What do you think can be the solution to the problems that you have mentioned? You mentioned several problems so I will be reading as you give me solutions to each problem.

**Respondent: Yes**

**Moderator:** About drugs you said that there are some drugs that you pay for from your pocket

**Respondent: Yes**

**Moderator:** What would be the solution to that?

**Respondent: It would be better if we can get a donor to help us on those drugs like for my case it is very hard because the drugs that my husband is using for cancer are also expensive. Sometimes I have to stay without drugs when I don’t have money and my blood pressure goes up when I stay without drugs to an extend that I fall down and end up being admitted in wards**

**Moderator:** You also said that ulcers are also a hindrance in managing your blood pressure, what would be the solution to that?

**Respondent: All those things are caused by thinking and you cannot avoid thinking as human being with kids and like for now am the wife and the husband because my husband has not been working from 2013. With such a situation I just have to think. I also have a kid who joined form one and this one really brought me down**

**Moderator:** You also told me that there are times that you are forced to stay at home

**Respondent: Yes, when my blood pressure is high**

**Moderator:** What would be the solution to that?

**Respondent: I am always unable to walk when my pressure is high, sometimes I fall down and due to that I am forced to stay at home**

**Moderator:** What would be the solution to that?

**Respondent: You must have everything for you to avoid thinking coz if you miss something then you just have to think and that’s when the blood pressure and ulcers become worse**

**Moderator:** On the family you mentioned that your husband is a problem, what would be the solution to that?

**Respondent: I have started to understand him; I just walk out when he throws his food and come back after some time when he is ok but initially I could ask him why he was pouring food but nowadays I don’t ask him because he gets worse when I ask him such a question. His doctor at { Name of the facility} advise me to be assuming and act like he has done nothing because he never does that willingly**

**Moderator:** You also told me that there are times when you eat foods that you are not supposed to eat, what would be the solution to that?

**Respondent: I am forced to take what is available when I don’t have money to buy food and I am supposed to take drugs**

**Moderator:** You also told me that you don’t like the way they attend to you at the facility that you go for clinic, what would be the solution to that?

**Respondent: I think the solution would be leaving that hospital and going to where used to go before**

**Moderator:** I was asking to know the solution in relation to those that are attending to hypertensive patient

**Respondent: They are supposed to follow up and guide us on how we are supposed to live but we cannot be the once telling the doctor what to do because he will think that you are commanding him and that’s why we just keep quiet and change the facility**

**Moderator:** What else can you do differently as a patient?

**Respondent: In regards to what?**

**Moderator:** In regards to pressure

**Respondent: I don’t like listening to many things when my blood pressure is high because I get angry easily. I don’t like being close to people who don’t understand me especially when my blood pressure is high**

**Moderator:** How has this COVID19 period affected hypertension care service delivery in your community? Remember that you had told me that you stopped going to {Name of the facility} because of COVID 19

**Respondent: Yes**

**Moderator:** How has it affected you?

**Respondent: It has affected me coz they have hiked fare and when you we go to the hospital other are told to wait outside as they attend to other patients making us take a lot of time before we are served**

**Moderator:** Ok, is there any other thing that you would like us to talk about in regards to high blood pressure?

**Respondent: I would like to tell you to talk to those people if at all you work with them because hypertensive patients become worse when they are shouted at. Doctors should understand hypertensive patients because hypertensive and cancer are almost the same**

**Moderator:** Ok, thank you for the time that you have given me and the conversation the we have had. It will really help us in our research. Thank you

**Respondent: Ok. Thank you**

**…END…**
