# Supplementary material for: A socio-ecological framework examination of drivers of blood pressure control among patients with comorbidities and on treatment in two Nairobi slums; a qualitative study
Source: PLOS Glob Public Health. 2023 Mar 10;3(3):e0001625. doi: 10.1371/journal.pgph.0001625 (PMC10021823; doi:10.1371/journal.pgph.0001625)
Supplement: S1 File — (ZIP) [file pgph.0001625.s001.zip › Community/VIWA_UHTNC_200715_007.docx]

**Moderator: {Name}**

**Code: VIWA_UHTNC_200715_007**

**Moderator:** This community has been identified to have a high burden of uncontrolled hypertension which is a leading factor to premature deaths and disability. I am trying to gather information about hypertension care in your community. To avoid hypertension related complications, it is recommended that people with high blood pressure can change their lifestyles in regards to diet, physical activities, smoking, alcohol consumption and using blood pressure medication**.**So tell me about your experience with having high blood pressure**.**Tell me about your experience with having high blood pressure

**Respondent: My experience with blood pressure is that one has first to accept him or herself that he or she is hypertensive for you to walk with that book. If you are eating a balanced diet, doing exercise, taking medicine and honoring appointments by going for clinics as required then you will not have any problem but when I miss on taking those drugs then I develop a different problem. I always feel good when I have those drugs even though there is still a challenge sometimes like one feels bad when there is no food. You feel like you are losing site and the legs become weak so it is always important that you eat foods that give your body energy for you to be able to take drugs**

**Moderator:** For how long have you been having high blood pressure?

**Respondent: For several years almost 7**

**Moderator:** How frequent do you check your blood pressure measurement?

**Respondent: I do check on a monthly basis**

**Moderator:** Where do you check your blood pressure?

**Respondent: Sometimes i check at {Name of the facility} sometimes at the health Centre or at {Name of the facility}**

**Moderator:** Do you record your blood pressure measurement?

**Respondent: Yes**

**Moderator:** What was you pressure the last time you checked?

**Respondent: It was 120, there is a time when it was 110 and there a month when it was 149**

**Moderator:** Do you have any other condition apart from hypertension?

**Respondent: It is just as I told you before**

**Moderator:** Can you repeat for it to be recorded?

**Respondent: I had told you that I am HIV positive**

**Moderator: Ok.** Has your doctor told you what your blood pressure target should be?

**Respondent: You mean normal blood pressure?**

**Moderator:** Yes

**Respondent: It is supposed to be 120**

**Moderator: Tell me about the antihypertensive drugs that you are using**

**Respondent: The ones that I am using?**

**Moderator:** Yes, you have been hypertensive for like 7 years; tell me about the drugs that you have been using

**Respondent: The drug that I am using for hypertension condition is…5:47-5:56… (Not clear)**

**Moderator:** How do you use them?

**Respondent: I take one tablet a day**

**Moderator:** Are you using the same drugs from the time that you were diagnosed with hypertension 7 years ago?

**Respondent: They were changed when I started using those other drugs. I was give HTC and that is the one that I am using**

**Moderator:** For how long have you been using this one?

**Respondent: I have used it for like 6 years**

**Moderator:** Is the dosage the same as it was from the day you started using it?

**Respondent: It has changed coz it was 170 something at that time and for now it has gone down**

**Moderator:** How has hypertension affected you?

**Respondent: It had really affected me coz I used to feel my head aching all the time, sometimes I could feel like my legs were becoming weak and my hands were also shaking**

**Moderator:** How else do you manage your blood pressure apart from taking drugs? You had told me about dieting, exercising and taking medicine. Is there any other thing that you can add?

**Respondent: The other thing is avoiding stress even though as a human being you have to encounter stress**

**Moderator:** Have you ever used traditional medicine?

**Respondent: No**

**Moderator:** You told me that you go to the health Centre monthly

**Respondent: Yes**

**Moderator:** Who do you see when you go there?

**Respondent: The doctor that I see?**

**Moderator:** Do you see a doctor or nurse?

**Respondent: I go straight to a doctor who prescribes medicine for me then I go to the pharmacy to collect my drugs but there is a specific doctor who measures our blood pressure but I don’t know his name**

**Moderator:** Are you attended by a doctor or a nurse?

**Respondent: Nurse**

**Moderator:** What can you say in regards to the way your doctor is managing your blood pressure?

**Respondent: All I can say is that she is good because she measures me to know how my blood pressure is**

**Moderator:** Have you ever sought treatment elsewhere away from your village?

**Respondent: Yeah, there is a time I went to {Name of the facility}**

**Moderator:** Why?

**Respondent: At that time there were no drugs at the health Centre**

**Moderator:** What were you told when you went there?

**Respondent: My blood pressure was measured, the doctor prescribed drugs for me and I went to buy them**

**Moderator:** What services do you receive you go for clinic?

**Respondent: The first thing is to do blood pressure check to know how it is then they give me drugs and teach me how I am supposed to eat and avoid stress so that I am not brought down by blood pressure**

**Moderator:** Do you have insurance?

**Respondent: What?**

**Moderator:** NHIF

**Respondent: No, I just applied the other day**

**Moderator:** Do you pay for your drugs from the pocket or you are given for free when you go for clinic?

**Respondent: I used to get them for free at the health Centre but I paid when I went to {Name of the facility}**

**Moderator:** Looking at yourself as an individual, do you have any problem with managing your blood pressure? Like you just told me that you have applied for NHIF and you also said that you buy drugs, do you have a problem with that?

**Respondent: You see like now there is no job and everyone is not ok that’s why I cannot get money to buy drugs**

**Moderator:** Is your age a hindrance in managing your blood pressure?

**Respondent: No**

**Moderator:** You also told me that you have other condition apart from high blood pressure

**Respondent: Yes**

**Moderator:** Is the condition a hindrance in managing your blood pressure?

**Respondent: The other condition?**

**Moderator:** Yeah, do you think that HIV is a hindrance in managing your blood pressure?

**Respondent: No**

**Moderator:** You had told me about taking drugs as directed by the doctor, do you do that?

**Respondent: Yes**

**Moderator:** Do you use either alcohol or cigarettes?

**Respondent: No**

**Moderator: How is your normal day? Do you leave the house?**

**Respondent: I do go out**

**Moderator:** And if we look at the family and the community side, do you see any hindrance in managing your blood pressure?

**Respondent: In my family?**

**Moderator:** Yeah, looking maybe at your kids if you are marred, jut your family

**Respondent: No**

**Moderator: Looking at the environment around you, are you using any foods that are not good?**

**Respondent: Yeah**

**Moderator:** Is it a hindrance in managing your blood pressure?

**Respondent: I normally feel some difference in my body when I take something that is not ok with me**

**Moderator: You said the your health care provider at the health Centre advices you when you go for clinics**

**Respondent: Yes**

**Moderator:** How is their quality of treatment?

**Respondent: Their quality of treatment is not bad and it is not very good**

**Moderator:** What about the hours that you are supposed to be there for clinic?

**Respondent: When one reaches there he or she must wait if at all the health providers are not yet in**

**Moderator:** Looking at the hospital, you told me that you are given drugs there; are there times when you don’t find drugs?

**Respondent: You are told to go buy**

**Moderator:** So there are times when you don’t find drugs

**Respondent: Yes**

**Moderator:** Looking at the government policies, are they a hindrance in managing your blood pressure?

**Respondent: The government has no problem because they have provided health care to us but there is still a problem because sometime the drugs are not available**

**Moderator:** What can be the possible solution to the hindrances that you have mentioned? You have many hindrances so I will be reading to you as you tell me the solution, like for example you had told me that you lack money to buy drugs, what do you think would be the solution to that?

**Respondent: Say for example if there was business or maybe am working or even if my kids’ work were doing well, then I will not be having any problem in managing my blood pressure**

**Moderator:** You also mentioned that sometimes you lack food, what would be the solution to that?

**Respondent: Lacking food is normal because at this time my work is not doing well and in the business that I am doing maybe I didn’t get enough money to buy good food, the solution is just to work hard**

**Moderator:** There are times that you eat foods that are not good for you, what would be the solution to that?

**Respondent: I would buy foods that am supposed to eat if I was able to but now since am not able to buy them then I will just eat what I can get**

**Moderator:** You said that sometimes you receive quality care at the hospital sometimes it is not of good quality, what would be the solution to that?

**Respondent: If the government could help on drugs and maybe food then most people would not be affected so much**

**Moderator:** You also talked about lack of medicine at the hospital, what would be the solution to that?

**Respondent: You have to work harder for you to get money to buy drugs coz if you speak for like 2 day without taking those drugs then you start getting sick which might lead to increase in your blood pressure**

**Moderator:** You said that there is a time that the government helps and there are other times that things don’t go well, what would be the solution to that?

**Respondent: Things go back to worse when there are no drugs sometimes leading to deaths coz this high blood pressure condition is not a good condition. It’s a condition that can lead to disability**

**Moderator:** We are almost done, what can you as a hypertensive patient do differently?

**Respondent: What I can do is ensuring that I take drugs as am supposed to, do exercise and work harder at my job because I have to buy those drugs when they are not available at the facility coz I know that those drugs are my life**

**Moderator:** What can your health care provider do differently in regards to hypertension?

**Respondent: He is supposed to give patients the best advice so that they can accept themselves with the conditions that they are in**

**Moderator: looking at the facility, what is it that can be done differently?**

**Respondent: What can be done is for example you go to the hospital and there are no drugs and the health care provider shouts at you and maybe the doctor tells you to go and sit at home if you don’t have money. That can bring a big problem to hypertensive patients**

**Moderator:** How has COVID19 affected delivery of hypertension care services in your community?

**Respondent: Pardon**

**Moderator:** This is a COVID19 period

**Respondent: Yes**

**Moderator:** How has it affected delivery of hypertension care services in your community?

**Respondent: This period of COVID19 has affected me because there are no drugs at the hospital because travelling has been burnt, maybe you are not allowed to move or travel or walk without putting on a mask then that one can affect**

**Moderator:** Is there anything else that you would want us to talk in regard to hypertension care services?

**Respondent: What I can say about high blood pressure is that we be with people that advice o for high blood pressure checkups because it’s a condition that any person can get without knowing then you come to hear that this person is dead or affected because most of the people don’t have knowledge about it and others fear it and they cannot accept the results even if they are tested**

**Moderator:** Ok, I appreciate so much for the time and the information that you have given me and I believe that the information will help us in our research

**Respondent: Thank you**

**Moderator:** Good day

**Respondent: You too**

**…END…**
