# Supplementary material for: A socio-ecological framework examination of drivers of blood pressure control among patients with comorbidities and on treatment in two Nairobi slums; a qualitative study
Source: PLOS Glob Public Health. 2023 Mar 10;3(3):e0001625. doi: 10.1371/journal.pgph.0001625 (PMC10021823; doi:10.1371/journal.pgph.0001625)
Supplement: S1 File — (ZIP) [file pgph.0001625.s001.zip › Community/VIWA-FGD-200807_1507.docx]

**Moderator: {Name}**

**VIWA-FGD-200807_1507**

**Moderator:** This community has been identified to have a high burden of uncontrolled hypertension which is a leading factor to premature deaths and disability. I am trying to gather information about hypertension care in your community. To avoid hypertension related complications, it is recommended that people with high blood pressure can change their lifestyles in regards to diet, physical activities, smoking, alcohol consumption and using blood pressure medication**.** So tell me about your experience with having high blood pressure**.** Tell me about your experience with having high blood pressure

**R1: It was in 2016 that I woke up feeling so weak and I tried to prepare some porridge but when I went to the hospital I was checked and I was diagnosed with blood pressure. I was told that the condition almost killed me and so I was given drugs and I came back home and later on I came back to the health Centre where I was checked again and I was told that it was the same thing. I have been taking drugs from 2016 but it is only this year when Corona came though I have been buying drugs. Sometimes I become weak though I don’t know the symptoms of this condition though when I go for checkup I do find it at 170, it goes down to 140 or 130. I just use my drugs but I don’t know if the drugs are the reason for my headache or could it because of the diabetes that I was checked. I wanted to know the symptoms of this disease, how it starts and how it can be treated**

**Mod:** We may talk about that as we continue

**R1: Ok**

**Mod:** Can R3 tell us his experience on having high blood pressure?

**R2: I am {Name}, I can’t say that I have experience on this disease, sometimes I feel sick and I go to the hospital thinking that I have Malaria, they try treating Malaria but it doesn’t get treated and after checkup they find that my blood pressure is high and so I get confused if it is Malaria or blood pressure and when I go to collect drug they have always been giving me different types of drugs and this makes me confused. I don’t know the best drug that u can buy in case I find that the drugs are expensive at the hospital and I want to buy from the chemist. On the other side, nowadays when I walk around people tell me that I have changed, I am becoming fat but when I examine my body I can’t say that I am fat, it’s like my body is swelling but I don’t know. At my age I am not supposed to feel flesh when I pinch myself, I should just feel the skin. I just don’t understand myself**

**Mod:** Can anyone else share his or her experience on having high blood pressure? R4?

**R4: I don’t know how I was found to be having this blood pressure condition just like the other man said. I used to take alcohol and sometimes I could wake up feeling like I cannot walk but I couldn’t tell what the problem was. I thought that it is was malaria and I decided to go to a hospital within the community and when I explained how I was feeling the doctor told me that I had typhoid. I was injected and given drugs and after sometime I decided to take alcohol again and with time the feeling came again. I couldn’t understand why this typhoid is not getting treated. I had to stop taking alcohol but I could still fell that I was weak and sometime I could feel like I even don’t want to rise up from bed. I went to {Name of the hospital} and stopped going to the one that I used to go before. I was checked and I was told that I have blood pressure condition and when I asked what blood pressure is and I was told that it was because of my many thoughts. I was given drugs and I paid almost 600 shillings but after some time I had the same feeling and when I went to the same hospital I was referred to {Name of the facility}. I thought of {Name of the facility} and my job, I knew that I might lose my job if I went to {Name of the facility} yet I have kids who don’t have a mother and they are not able to take care of themselves because they were still in school. I asked a friend who told me about a {Name of the hospital}, I went there and as they were attending to me, I was asked what I have been thinking. I couldn’t answer because I couldn’t tell what I was thinking about because I have kids and their mother died and I had to think because these kids depend on me. My blood pressure was fluctuating and I was told to continue taking drugs because I couldn’t stop taking drugs**

**Mod:** What’s the name of the hospital that you went to?

**R1: {Name of the facility}**

**Mod:** Can any other person share his or her experience? R6

**R6: With this condition you can’t tell if you are sick or not. Like for me I was just taking my kid for clinic and I found people being checked on their blood pressure and when mine was checked it was that it was high and by that time I was not feeling anything. The doctor asked me who I was with and I told him that I had brought my child for clinic. I was told to find something to eat then I take the drugs that I was given there and wait for two hours. I was being checked after every 30 minutes and after I was checked again and the doctor said that the pressure had gone down. I was given drugs and told to go home**

**Mod:** R5 kindly tell us your experience

**R5: I was diagnosed with diabetes at first but I did not believe that I am diabetic but I was not feeling like taking anything. I was just feeling thirsty and hungry and I was feeling weak though I was not taking diabetes drugs at that time. I started using drugs after like one or two years when the doctor told me that I was hypertensive and I should start taking drugs before I fall on the way because of high blood pressure. I used to feel dizzy even after taking drugs, that’s how it started. I had to stop taking some foods because I felt bad whenever I took them**

**Mod: Can R2 share her experience too?**

**R2: I am {Name}. This condition started when I was hospitalized at Name of the hospital} because of diabetes. My daughter died after I left {Name of the facility} and because of that I was taken again to {Name of the facility} where I was told that my blood pressure was high. I took drugs for like 2 years and when I went for checkup I was told that my blood pressure was ok. I stopped using those drugs and nowadays I use diabetic drugs. This condition worsens when one things a lot and everyone has to think**

**Mod:** R3. Tell us, for how long have you been having this condition

**R3: It has been a while, almost 4 years since**

**Mod:** R4. For how long have you been having this condition?

**R4: More than 10 years**

**Mod:** More than 10 years?

**R4: Yes**

**Mod:** R5?

**R5: 5 years**

**Mod:** R6?

**R6: 2 years**

**Mod:** R1?

**R1: 4 years**

**Mod:** I would like to know how frequent you go for checkup to know our blood pressure condition. R5 how frequent do you go for checkup?

**R5: With blood pressure you just can’t understand yourself, you just feel like you don’t want anything and then you decide to go to the hospital for checkup for you to know what could be the problem. You just feel like you don’t want either to sit or sleep and that’s when you decide to go to the hospital**

**Mod:** I would like to know the period that you take before you go for check up

**R5: I can’t tell the exact period but my body becomes unwell when I attend occasions and eat certain foods**

**Mod:** You mean that you go to the hospital at that time when you feel unwell

**R5: Yes, that’s when I go to the hospital for checkup**

**Mod:** R1, tell us the period that you take before you go for blood pressure check up

**R1: I go for check up every month because I take drugs that can take me for one month and then I go back when they are finished. The last time I was checked I was told that it was 130**

**Mod:** R2 tell us period that you take before you go for checkup

**R2: I usually go after 3 months when I go to collect drugs but I don’t take drugs when I find that it is ok**

**Mod:** R3 how often do you go for checkup?

**R3: Initially like 2 years ago when I was being treated in Nairobi, I used to go for check up whenever I could feel sick but I decided to go to a hospital located at my rural home called {Name of the facility} because I felt like I was not receiving any helped here. I was checked on everything and the doctor booked me for monthly clinics. I went there for like 1 year then Corona came and we were locked down. I have not gone there again for clinic though I do go to the hospitals around whenever l feels bad and when I go there they do check my blood pressure and am always told that it is high**

**Mod:** How frequent were you going for checkup before corona?

**R3: I used to go there after every month**

**Mod:** R6, how often do you go for checkup?

**R6: I go after 2 months**

**Mod:** Which hospital do you go to?

**R6: I go to the health Centre**

**Mod:** R5

**R5: I can go to any hospital, sometimes I go to the health Centre and other times I go to {Name of the facility} or {Name of the hospital}, it depends with the way I am feeling**

**Mod:** I would also like to know if we have any other condition apart from high blood pressure. Is there anyone who has any other condition apart from high blood pressure? R3 tell us the condition that you have

**R3: I have ulcers**

**Mod: For how long have you been having ulcers?**

**R3: For almost 4 years**

**Mod: Is there anybody else who has any other condition apart from high blood pressure?\**

**R2: I have been diabetic for like 5 years**

**R5: I am also diabetic**

**Mod: Is there any other person who has any other condition apart from high blood pressure?**

**R1: I always have a headache**

**Mod:** Have you ever explained that to your doctor when you go to the hospital for your clinics?

**R1: It started the other day. I have been feeling like that for the last two days. I would like you to tell me because I have been having this headache for the last 24 hours and I don’t know what the problem is.**

**Mod:** I can only advice you to share this information with your doctor coz he is the one that has been attending to you and so he can easily tell you where the problem is

**R1: Ok**

**Mod:** What do you want to say R4?

**R4: When you feel that just go to a hospital that is close to you for the doctor to check your blood pressure for you to know what the problem is. Don’t just say that it is Malaria, go to the hospital and you will be told the exact problem after you have been checked**

**Mod:** Have you had what R$ has said?

**R1: Yes**

**Mod:** It’s better for you to go to the hospital because they will examine and tell you what the problem is instead of you saying that you have Malaria

**R4: Just go to the nearby clinic for checkup. At the clinic you will be told if the problem is blood pressure or not and you will be given drugs**

**Mod:** I know that we all go for checkup. Has your doctor ever told you what your normal blood pressure target should be?

**R1: I was told that it is supposed to be between 80 to 120**

**Mod:** Over what? There always two numbers, one number is above the other one bellow

**R1: 80 should be the lowest and 120 the highest over 110**

**Mod:** R6, what did your doctor tell you

**R6: 120/70**

**Mod:** R5 what were you told?

**R5: It should be 120 or 110 over 80**

**Mod:** R4?

**R4: I can’t remember**

**Mod:** R3?

**R3: 120/70**

**Mod:** R2?

**R2: 120/80**

**Mod:** How many of us still remember their last blood pressure measurement readings when they went for check up

**R6: It was 143/90**

**Mod:** Is there any other person who can remember?

**R5: It was 116/80**

**Mod:** Any other person who can remember

**R1: Mine was 170**

**Mod:** 170 over what?

**R1: Over 100**

**Mod:** What about you R3

**R3: I cannot remember**

**Mod:** On to the next question where I would like to know the drugs that you are using to manage your blood pressure. Starting with R4, which drugs are you using?

**R4: I don’t know the names of the drugs that I am using but they always change my drugs when I go there and they find that my blood pressure is high or they give you a drug that you will take and wait for like 30 minutes then before you go back to be checked again before they prescribe drugs for you. They always change for you if they find that the ones that you are using are not effective**

**Mod:** How many tablets do you take?

**R4: I take 3 tablets**

**Mod:** What about you R3?

**R3: I use 4 types of drugs because there is one that I take a half a tablet, there is another one that I take 2 tablets and there is this one that I take 4 four day and then I stop. That one is just for urinating; I was told that it is used to remove something that I don’t remember**

**Mod:** How many drugs are you using R1?

**R1: I don’t know but I was given 3 packets**

**Mod:** How do you take the 3 packets?

**R1: I take in the morning and in the evening**

**Mod:** R5 can you tell us?

**R5: I take one tablet once a day**

**Mod:** Have you been using the same tablet from the time that you were diagnosed or they have ever changed

**R5: I used to take a half a tablet but later on I was told to take it full**

**Mod:** Is there anyone who has not told me? R6, how many tablets do you take

**R6: In the morning I take one tablet called…24:20… (Not clear) and there is another one called HTCZ that I take a half, so I normally take 1 and a half tablets per day**

**Mod:** You take 1 and a half tablets but two different types?

**R6: Yes**

**Mod:** For those that their drugs have changed, I remember that there is one who said that she started with a half a tablet but nowadays she takes one full tablet, there is another one who said that their drugs changed, can you tell me the reason that made the doctor to change for you your drugs? Is there anyone who was told the reason, R1?

**R1: The doctor just examined me and he gave me the drugs, that’s all**

**Mod:** Can anyone tell me the reason as to why their drugs were changed?

**R3: There was one that always made me feel headache whenever I took it and it was later on changed and I was given another one**

**Mod:** R5

**R5: My drugs were changed because both my blood pressure and my sugar levels were high and from when they change I have been ok. I don’t have a problem with that**

**R2:** Have your drugs ever been changed?

**R2: I was told to add the drugs that I do inject myself with when I was told that my sugar levels were high. I was told to go to the hospital on Thursday every week and then later on I went back to my normal clinic days when my sugar levels went back to normal**

**Mod:** The other question that I would like to ask is that I know that this condition has affected us in one way or another, true?

**In unison: Yes**

**Mod:** I would like to know how high blood pressure has affected your life. Everyone has been affected differently and so I would like to get opinions from everyone starting with R6. Please tell us how high blood pressure has affected you

R6: **How can I say?**

**Mod:** How has it affected your life?

**R6: It has affected me because I have to minimize my thinking and I am not supposed to be annoyed**

**Mod:** R5, how has it affected you?

**R5: It has affected me because of getting annoyed. My children are the reason as to why I get angry and that makes my blood pressure to rise. There is nothing else**

**Mod:** What do you mean when you say that it has affected you on getting angry?

**R5: I have a son who has been misbehaving and I tried talking to him but he was not listening to what I was telling him. He was later on arrested and that issue really affected me to an extend that I had to be admitted in a ward**

**Mod:** R4, please tell me how high blood pressure has affected your life

**R4: This condition comes from thinking a lot. My wife died as we were eating on one plate. We were using a lamp that had petrol and we didn’t know. She died before we reached at the hospital**

**Mod:** Sorry

**R4: I was left with my kids who were still young and after this shock that’s when I went to the hospital and I was told that I have blood pressure. This issue has affected me a lot and I have a child who takes alcohol. He always gets drunk whenever he gets money**

**Mod:** Sorry for what happened but I would like to know how this high blood pressure has affected your life

**R4: It has affected me. I have lost almost everything because I don’t have that energy that I need to work.**

**Mod:** What has changed looking at the time that you didn’t have high blood pressure and now?

**R4: I used to do farming but I had to stop because most of my money is used on medication and I have kids. I lost everything and now I just like a temporary life**

**Mod:** R3 please tell us your experience

**R3: It affected me because I used to do a lot of work because I work in the informal sector. I am a carpenter but nowadays I do get tired easily whenever I try to do that job. I can’t work like I used to work before. On the other side if something happens and we are told to run then I can’t run for a long distance because I will start breathing heavily but you couldn’t catch me when I was ok. I really get tired when climbing stairs. It has really affected me**

**Mod:** R2 can you tell us how blood pressure has affected your life

**R2: It has affected me because there are some jobs that I cannot do, I used to wash peoples’ clothes at a pay but nowadays I cannot do that. It has affected me a lot**

**Mod:** I know that you all use drugs to manage your blood pressure, R3 tell us, apart from using drugs, what else do you do to manage your blood pressure

**R3: What else do I say? I really try not to be angry because I know that anger is the beginning of all this**

Mod: Is there anybody who does something different to manage their blood pressure?

**R1: This disease is automatic like for example my neighbor collapsed and died while taking bath. They tell us to avoid thinking yet there is no any man or woman who can avoid thinking. We have to think on how our kids will go to school, what they will eat and how life will be and this condition can kill you at any time. We can just leave this place and you get information that I have died. In June my child told me that he has received a letter informing him to report to school, I just kept quiet and didn’t talk to him because I needed 60,000 shillings for this yet I did not have money. I later on told him to have faith that he will go to school. This condition has a lot of issues. With my job I can stay even for a week and with such a situation you just have to think**

**Mod:** R2, apart from using drugs, what else do you do to manage your blood pressure?

**R2: By eating what I am supposed to eat, I avoid thinking too much. That’s what I do to manage my blood pressure**

**Mod:** R5, apart from using drugs, what else do you do to manage your blood pressure?

**R5: I go to noisy place or I just talk to my fellow women and that help me forget the issues that might disturb my mind**

**Mod:** R6, do you have anything to mention in regards to that?

**R6: you are supposed to take a lot of water because water helps in regulating blood pressure and also avoid taking paper or coffee**

**Mod:** I have heard most of you talking about food, is there anyone who can mention something on exercise?

**R1: I know that there are people who do exercise by running in the morning but for me the work I do is just like doing exercise. Sometimes at work I do climb stairs, I carry heavy loads. That’s the exercise that I do**

**Mod:** Is there anyone else who want to talk about exercise?

**R3: I normally do exercise, with my work I most of the time you will find me seated as I carry people on my bike and I walk a lot when am not working. Like for now I just decided to come to this place walking. I prefer walking a lot**

**Mod:** Any other person who has any other opinion in regards to exercise?

**R4: At my work I can just stand or walk for 3 0r 4 hours without sitting but this makes me so tired to an extend that I can just sit somewhere and find myself asleep because of being so tired. I can leave my house for work and at work I don’t find time to sit, at around 11 or 12 is when ill find time to sit down.**

**Mod: R6, what can you mention in regards to exercise?**

**R6: For training one can either walk or run**

**Mod:** Which one do you do among the two?

**R6: I walk**

**Mod:** What about you R5?

**R5: I like doing my work by myself, I walk, like I even came here walking. I don’t have a problem with walking of doing my work**

**Mod:** Is there anyone who is using traditional medicine to manage or control blood pressure condition?

**R1: I have never used**

**R5: I have never used**

**R3: I have never used**

**R2: I have never used**

**R4: I have never used**

**R6: I have never used**

**Mod:** It’s like all of us have never use traditional medicine to manage blood pressure. Who do you see when you go to the hospital seeking hypertension care?

**R5: A doctor is always there. There is someone who checks blood pressure and for those that are diabetic are also checked on their sugar levels then we go to see the doctor**

**Mod:** Who is this other person?

**R5: He is there to check blood pressure**

**Mod:** Which hospital do you go to?

**R5: I go to Mareba**

**Mod: R3, who do you see when you go to the hospital?**

**R3: A doctor**

**Mod:** Is there anyone who sees a different person apart from a doctor?

**Respondents: *Silent***

**Mod:** Does that mean that we all see a doctor?

**Respondents: Yes**

**Mod:** What can you say about the doctor that manages your blood pressure?

**R4: I don’t have any input though my doctor told me that he does what he can based on what he knows. There is a time when I asked my doctor, can this condition be cured so that one can stop using this drugs and he told me that once you cannot stop using these drugs once you have started because you cannot avoid thinking and that’s what makes the blood pressure to rise and your heart can still be shocked. That’s what my doctor told me, he can’t do anything else other that prescribing drugs as directed**

**Mod:** R3, what can you say about the doctor that attends to you?

**R3: I trust the doctor that used to attend to me at {Name of the place} because I think that he used to attend to me better but not the doctor that is attending to me here. I told you that there is a time I was sick and I was given antimalarial drugs. I told him to check my blood pressure and he found that it was high. I am the one who told him the problem**

**Mod:** You have mentioned only one case, what else makes you think that the doctor at Mwea is better that the other one?

**R3: The doctor at {Name of a place} couldn’t treat any condition before doing tests and this other doctor just prescribes drugs when you tell him how you feel**

**Mod:** Ok. What about you R2. What can you say about the doctor that attends to you when you go to the hospital?

**R2: I have never seen anything that was wrong with him because he checks my BP, Sugar levels before I am directed to the doctor**

**Mod:** Thank you. Is there anyone who can share what he/she thinks about his/her doctor? Anything different from what we have heard

**Respondent: *Silent***

**Mod:** R3 said that he used to go to {Name of a place} but he has not gone there from the time Corona was announced to be I Kenya and you gave reasons that made you change like for example you talked of a doctor who just gives you drugs without performing some tests on you. Could there be any other reason that made you change the facility that you used to go to?

**R3: There is no reason. I just went to see my family in {Name of a place} where I fell and I was taken to that hospital in Mwea. I was tested everything for them to know my problem and after they were done testing I asked them why didn’t they test for HIV because I suspected that I could be having HIV and I was tested here and the results were negative. I asked them to test again so that I can confirm. They confirmed that I was negative positive though I was hypertensive and I also had ulcers**

**Mod:** R4, have you ever sought treatment elsewhere?

**R4: I have never gone elsewhere from the time I was diagnosed because they are the ones who diagnosed the problem**

**Mod:** You have never gone elsewhere?

**R4: I have never because I can’t go to another place. Can you go to a place where you just heard they just prescribe medication without even testing to know the problem? They can even give you drugs that are not meant to cure the condition that you have and it can worsen the condition**

**Mod: R6:** Have you ever sought care elsewhere?

**R6: No**

**Mod:** Where else can we get hypertension care within our community? Can we mention places where we can get hypertension care services? Let’s start with R6

**R6: {Name of the facility}, {Name of the facility}**

**Mod:** R5, where can you get hypertension care within viwandani?

**R5: I know of {Name of the facility}, {Name of the facility} or {Name of the hospital}. Only those ones]**

**Mod:** R4, where can you get hypertension care within viwandani community?

**R4: I cannot tell you because I have never changed. I had gone to another private hospital located in {Name of a place}, then I went to {Name of a hospital} and then {Name of afacility} where I was tested and diagnosed with this condition. I have never thought of changing**

**Mod:** Where did you say that you are currently going?

**R4: St, Mary’s**

**Mod:** R3 can you mention where one can get hypertension care services within this community?

**R3: I only know of private hospitals, I don’t know the other ones**

**Mod:** Like which one do you know?

**R3: Huduma bora**

**Mod:** R2?

**R2: I used to go to {Name of the facility} but I have been going to the health Centre from January**

**Mod:** Ok

**R1: I only know of the health Centre and that’s where I always go**

**Mod**: What services do you get when you go to th3ese facilities in search of hypertension care? For example R3 told us that they used to check you up and you have been going there for your monthly clinics

R3: Yes

**Mod:** Can you tell me the services that you used to get when you were going there for clinic?

**R3: Apart from the normal service?**

**Mod:** The services that you used to get in relation to hypertension

**R3: There was maternity service**

**Mod:** I was asking of services related to hypertension. Can R4 tell us?

**R4: You give out your card after reaching the hospital so that they can start attending to you, you sit and wait to be called in for blood pressure checkup and after that you are referred to the doctor where you are supposed to tell him if you have any other problem so that he can refer you for a test if at all the condition needs another test like stool test or diabetes checkup then you bring the results to him. That’s how it has been at the hospital that I go to**

**Mod:** Is there anyone who has something different from what we have been told by R4? What services do you get when you go to the health Centre R6?

**R6: I give them my book then I queue before I am checked by a nurse, and then I go to the doctor who prescribes for me drugs that I am supposed to take**

**Mod:** Is there anyone who has something different from what we have heard from R4 and R6?

**R1: Everyone that comes to the hospital has a different condition and we are all directed from the reception on where to go according to the condition that brought you to the hospital**

**Mod:** For now we are talking about hypertension. What services do you get when you go to the hospital for hypertension care?

**R1: I start from the reception then I go for blood pressure checkup then I take my results to the doctor and from there the doctor prescribes the drugs**

**Mod:** Ok. Is there anyone who has something different?

**R4: There have been changes at this time of corona. At first you are checked on corona when you reach the hospital. You can be denied access if you are found positive and if you are negative you will be allowed in and for example at my hospital you will be checked if there is anything that you are carrying and you are not supposed to go with it inside the hospital then you go to the reception where you give your card, from there you go for blood pressure checkup then you see the doctor and if you don’t have another problem then you will just be given a list of drugs that you are supposed to collect.**

**Mod:** Ok. For those who got to Mareba, do you pay for the services that we get or we do get the services for free

**R5: We are treated for free but many times we are told to go buy drugs. Some drugs are not available**

**Mod:** You get treated?

**R5: They check the blood pressure then we go to the doctor but when we go to pick the drugs we are told they are not available**

**Mod:** Ok. Anybody who has something different? R3, were you getting free health care services or you were paying at Mwea

**R3: I only heard that they offer free Tuberculosis care services but for the other services like blood pressure, they don’t charge blood pressure measurement and doctor’s consultation though you have to pay for drugs**

**Mod:** What about R4 who goes to {Name of the facility}, what can you tell us?

**R4: At St. Mary’s I pay for drugs though there those services that are different and you have to pay for them before anything. There is nothing that is offered there for free. You start with the card that you have to pay for before you go to see the doctor for blood pressure measurement or any other condition and in case you need to do any test then you will also pay for that**

**Mod:** R2, do you pay for the services that you get when you go to the hospital or you get then for free?

**R2: At {Name of the facility} you have to pay for a file and the again you have to pay for the drugs that the doctor prescribes for you**

**Mod:** Ok

**R2: But at the health Centre I don’t pay and that’s why I changed from {Name of the facility} to the health Centre**

**Mod:** So you stopped going to {Name of the facility}?

**R2: I no longer go there; nowadays I go to the health Centrebecause of that**

**Mod:** You had told me that you stopped going to {Name of the facility} because of corona

**R2: No, I stopped going there because sometimes I couldn’t get money for transport and file charges and I went to the health Centre when I knew that I can be treated for free**

**Mod:** Is there anything that you can add R1?

**R1: At first we used to get drugs for free at the health Centre but nowadays the doctor prescribes and when we go to collect drugs we are told that they are not available**

**Mod:** What challenges do you get in managing high blood pressure?

**R6: Sometimes the doctor prescribes drugs for me but I can’t buy all of them because I don’t have money so I end up buying daily dosages**

**Mod:** Is there anyone who has another difficulty?

**R5: Sometimes it’s difficult to choose between buying drugs and food especially for the diabetic and hypertensive patients yet both food and drugs are of equal importance for your body. If you miss taking drugs then the blood pressure will increase and it’s also a problem if you miss on food. Those are the challenges that we face**

**Mod:** R4, is there any other challenge you can mention?

**R4: The main problem is that with this condition you have to use money and its worse if you don’t have money. I am telling you that I have sold many things because of medication. There is no hospital that will treat you for free. They just prescribe and when you go to collect they tell you that drugs are not available. Its better you go to these mission hospitals where you can get drugs at a lower price instead of going to buy at the chemist where prices are high**

**Mod: Ok. R3, please tell us the challenges that you get in managing your blood pressure**

**R3: Luck of money especially at this time of corona. I do bodaboda business and the government ordered a curfew from7:00pm to 5:00am and this reduced our working hours because police used to chase us during curfew hours. We can only work during daytime yet other boda boda riders don’t want to see you parking at a stage that you don’t belong to. Those are the challenges that I face**

**Mod:** R2, what challenges do you face as you try to manage your blood pressure?

**R2: We are told not to eat the same food for two days and getting different foods is a problem**

**Mod:** Is there another problem that you have not mentioned before we move to the next question?

**Respondents: *Silent***

**Mod:** Is age a challenge in managing blood pressure?

**R3: Age is not a challenge**

**Mod:** Is there anyone who has a different opinion in regards to age?

**R5: I didn’t get you**

**Mod:** Am asking if you age is a challenge in managing your blood pressure

**R5: Yes, it can be a challenge**

**Mod:** Why do you say that?

**R5: I said that because the body loose strength to fight many things as we grow old**

**Mod:** Is there anyone who has something to add on what we have been told about age?

**R4: What I can add is that our bodies’ loose strength as we get old and it becomes worse when the diseases come in but you have to struggle with the age and the diseases because there is nothing you can do other than taking drugs. You can’t have the same energy that you had when you were young**

**Mod:** Is there anyone who has something to add on age?

**R6: I don’t think that age is a challenge because you can also get young people at the hospital suffering from this condition**

**Mod:** How many of us use NHIF cards to pay for drugs?

**Respondent:** ***Silence***

**Mod:** Is there anyone who has an NHIF card or any insurance?

**R4: Pardon**

**Mod:** Is there anyone who has an insurance card or NHIF

**R4: I do have it**

**Mod:** Have you ever used to pay for your drugs?

**R4: I have never used**

**Mod:** Why

**R4: I tried using it but I was told that they don’t take that because they don’t get money from insurance. I had to pay with my money to access treatment**

**Mod:** Is there anyone who has ever used an insurance card?

**R3: I have never used**

**Mod:** R2, have you ever used your insurance card at {Name of the facility}?

**R2: I don’t even have it**

**R1: I have never used**

**Mod:** For those who have other conditions apart from high blood pressure, like for example R3 you said that you have ulcers

**R3: Yes**

**Mod:** Is it causing any difficulty in managing your blood pressure condition?

**R3: I can say that it is a hindrance because there are many foods that I was told not to eat yet they are the foods that I liked eating**

**Mod:** Which foods were you told not to eat?

**R3: Sweet potatoes, sukuma, beans, such foods. I cannot mention cigarettes and alcohol because those are not good although God has helped me; I stopped using them because of this condition**

**Mod:** Is there anyone else who has something to add on this? R4 told me that you have ulcers. Is there any other person who has another condition apart from high blood pressure?

**R5: I am diabetic**

**Mod:** Is your diabetic condition a challenge in managing your blood pressure?

**R5: It is contributing because when my sugar levels are high then my blood pressure also rises. That’s how they are**

**Mod:** What can you say about that in relation to the drugs that you are using?

**R5: I don’t use the same drugs that I started with. I used to take one tablet a day, then two and nowadays I take 4 tablets**

**Mod:** Are the tablets meant for blood pressure or?

**R5: I take 4 tablets for diabetes, 2 tablets in the morning and 2tablets in the evening and I also have another one then again the antihypertensive tablet**

**Mod:** In total how many drugs do you take?

**R5: In the morning I take 3 tablets and 4 in the evening**

**Mod:** I wanted to know the types of drugs that you use, are they 7 different types or?

**R5: in the morning I take...1:10:22-14 (Not clear) and at night I take 2 … 1:10:27-33 (Not clear) and 1 HTCZ**

**Mod:** There is one who said that those who have kids have a challenge in managing blood pressure. Is there anyone who has anything to add in relation to how the family and community hinder them from managing blood pressure?

**R5: Everybody has his or her reasons**

**Mod:** Please tell us your reason

**R5: I get annoyed easily and my heart beats faster when I am annoyed that I can even fall down**

**Mod:** R6? What are the family or community factors that hinder you from managing your blood pressure?

**R6: Those days that I am supposed to pay my rent**

**Mod:** What happens on that date?

**R6: You have to think on how you will get money because maybe you only have half of the money and you don’t know where to get the rest of the money**

**Mod:** R1?

**R1: There are many family issues. You come home and find that your wife had an argument with the neighbors, my mother or my brother. At that point you don’t know where to start from because people may start saying that your wife is the one controlling you and it’s also another issue when you take the parents side. Those are the issues that make us start thinking because it can become worse if you don’t take a neutral stand. There must be conflicts within a family; it can’t just be peaceful always**

**Mod:** R3, are there any community or family factors that hinder you from managing your blood pressure?

**R3: There no hindrances within my family**

**Mod:** R2

**R2: You can’t avoid thinking when you are still on this earth because there will always be a reason for you to be angry**

**Mod:** R3 told us that there was a hospital that he used to attend where the doctor used to give him drugs without checking him, there is a time that he was just given antimalarial drugs. Can you tell me what your health care is doing or he is not doing that hinders you from managing your blood pressure?

**R4: This blood pressure condition was brought up by my children issues. I have two kids and one has a mental problem. I have tried taking him to the hospital we went to {Name of the facility} and we were told that we will be given drugs that he can take from home. At home the child meets his friends where they take drugs that make him loose his head. I would prefer him to be locked at {Name of the facility} because I can’t be ok because my mind is always engaged. He can receive better treatment at {Name of the facility} because at home he will still take alcohol and smoke bhang and there is nothing you can tell him at that point and this makes my blood pressure to rise**

**Mod:** Ok. We were talking about the doctors that attend to us. There is one who said that he is ok with what his doctor is doing. Do you have anything that you think that your doctor is not doing and if he did that then your blood pressure condition would be ok?

**R3: For private hospital they are just after money. I go to a private hospital and they attend to you and give me drugs because I pay for that but I am sure that it different with the public hospitals**

**Mod:** R6, what is it that can be done differently at the health Centre?

**R6: My doctor should advise me on what I should eat and what I should not eat**

**Mod:** R4, what would you want to be done differently at {Name of the facility}?

**R4: I would be ok if my children changed their behaviors or if they just go elsewhere because my brain will not be disturbed if that happens. I will not think a lot if that child would be attended to. They always disturb me yet they are grown up men**

**Mod:** What can we say about the quality of treatment that we get when we go to the hospital?

**R2: The treatment is not bad because they always advise us on what we can use and they do follow ups**

**Mod:** R3 tell us about the quality of care at the hospital that you used to go

**R3: It is ok because they always give me drugs and I feel better when I take the drugs and if the issue is blood pressure, it goes back to normal after taking the drugs**

**Mod:** R1?

**R1: All I know is that you will feel better if you just take in the instructions that you are given at the hospital. You might not be healed completely but your blood pressure will be controlled**

**Mod:** What can we say in regards to the working hours at the facilities that we go to for clinic?\

**R1: The time that we go in or the time that we check out?**

**Mod:** The time that you come in and to the time that you come out after you have been attended to

**R1: What I know is that at the health Centre it always depends with the number of people seeking care at the facility. Those that come late have to wait on a queue for those who came earlier to be treated. The doctors also need their time to prepare in how they will work and that consumes time too**

**Mod:** R3, what can you tell me about working hours at the facility that you attend?

**R3: Timings at the private hospitals are ok because they attended to you immediately when you reach the hospital though I had to set aside a whole day when I go for my clinic at Mwae. There is nothing else I could do on that day because I used to go there in the morning then we start with a prayer then we go for blood pressure checkup and then we all assemble to be advised by the doctor**

**Mod:** Ok. Anyone who goes to a different hospital? I have heard about the health Centre and {Name of a place} How are the facility working hours at {Name of the facility?

**R4: There are no problems at { Name of the facility} because the doctors report at the normal time. You will find the doctors there at 8:00am. We queue for treatment and I have never experienced any problem there**

**Mod:** Ok. Do we get enough information on high blood pressure at the facilities that we go for clinic?

**R6: I don’t know because I have never received any information**

**Mod:** R1, do you receive any information about high blood pressure at the facility that you go for clinic and is the information enough**?**

**R1: I have never received any information**

**Mod:** Let us hear of any other person who attends any other facility apart from the health Center

**R3: The information at {Name of a place} is ok because we are advised on what to eat, how we can live with this condition and how we can take care of ourselves**

**Mod:** Ok. R4, do you receive information about high blood pressure at {Name of the facility}?

**R4: Yes, we are told not to eat paper because it can lead to a rise in blood pressure because it makes the heart to beat faster but I have never been told about what am supposed to do or what I should eat**

**Mod:** We have national health policies that control supply of drugs, health facilities and they provide guidelines on how hypertensive patients can be attended to. You said that you don’t get drugs at the hospital. Is there anything else that we can mention about drugs?

**R1: There is shortage of drugs at the hospital and if you had planned to take drugs then you have to go without drugs for some time before you come to collect them later sometimes we just loose hope because we depend on the hospital to provide us with drugs**

**Mod:** What can we add I regards to unavailability of drugs at the hospital?

**R3: it would be better if these drugs were offered for free because we are always told that services at the public hospital are free but when one goes there you are told to go buy the drugs**

**Mod:** Are there any government policies that hinder you from managing your blood pressure condition?

**Respondents: *Silent***

Mod: Let us look at this question as we finish. On the challenges that we mentioned, there is one who talked about drugs, there is one who talked about finances and there is one who said that he has children who always disturb him and he finds it hard to talk to them. What can we do to solve this issue of drugs at the hospital?

**R6: The suppliers should ensure that they avail enough drugs**

**Mod:** You said that these drugs are expensive yet most of the time they write for you and tell you to go buy outside. What can you say about that? R5?

**R5: I can’t say anything because the government knows that we need drugs and they are the ones to confirm if all the hospitals have drugs**

**Mod:** Anyone else? R2?

**R2: I have nothing to say**

**Mod:** As we finish, you said at the health Centre there are many people who come seeking treatment so you have to queue, there is one who also said that it take him a whole day when he goes to {Name of aplace} for clinics, What is it that can be done so that you can be able to access care faster?

**R2: You can’t come at the hospital in the morning then the person who comes late be attended before you are attended to. You just have to queue and wait for your turn. The doctor attends to us as we come**

**Mod:** Is there anyone who has anything to add on that?

**Respondents: *Silent***

**Mod:** There is this Covid situation that has affected the whole world; I would like to know how this COVID 19 has affected your access to care at the facilities that you go to

**R3: It has affected me because I have never gone to {Name of a place} where I used to get good health care services. At {Name of a place} they are very strict and if they see someone from Nairobi, they send you to quarantine**

**Mod:** Ok. R1?

**R1: We fear going to the hospital because one is not allowed to go back home in case you are tested positive so I just decided to wait until the corona issue is over. For now I am just buying the drugs that am using**

**Mod:** Is there anyone else who has anything to add on how COVID 19 has affected how they get high blood pressure treatment?

**R5: It has affected me because I cannot go to those places that I used to go for work yet I don’t have any other alternative. I just sit because when I go there I don’t get money which makes me unable to buy drugs and food. There is no one who thought that it will reach to this extend. We thought that thing will get back to normal after one month. It has affected us enough**

**Mod:** R4, is there anything that you can add?

**R4: This thing has affected everybody. It has affected me because everything has hiked in prices. Transport charges have doubled and you can pay thrice the normal amount at the evening. You cannot visit any of your friends; you just have to stay at home. This thing has really impacted a lot on transport. It is very expensive to travel because the prices have doubled because in the matatus you just sit I person per seat so we have to pay for the seat that has not been used**

**Mod:** Ok. Is there anything that we have not talked about and you feel that we can talk about it in regards to high blood pressure?

**R1: Is there a way through which we can be helped as people who have this blood pressure condition?**

Mod: As APHRC, our main task is research. We do research to know how the situation is then we share the report with those that are responsible for them to make changes or make things better and if there is any other thing that you would like to understand better in regards to hypertension, You have a right to ask him any question or any information that you would want to know and it will be easier to seek clarification from the doctor on anything that you want to know better. This is the doctor that has been attending to you and he is the one who knows the problems that you have. We only collect information from you to know how the situation is in Viwandani or maybe Korogocho. After we collect all this information, we share with the policy makers who come up with policies that can make the situation better so that everyone can benefit

Mod 2: To add on that, we have understood that there is a problem, we note the problems like have done then we take the problem to the people responsible like at the hospitals that we go to or the government. We tell them what we have gathered so that they can solve the problems that are there so that everyone can get health care services. Have you understood?

**Respondents: Yes**

Mod: Thank you so much for your time and I would like to wish you a good evening as you go home. The information that you have given me will help us to better the current situation in our community. Thank you very much

**…END…**
