# Supplementary material for: A socio-ecological framework examination of drivers of blood pressure control among patients with comorbidities and on treatment in two Nairobi slums; a qualitative study
Source: PLOS Glob Public Health. 2023 Mar 10;3(3):e0001625. doi: 10.1371/journal.pgph.0001625 (PMC10021823; doi:10.1371/journal.pgph.0001625)
Supplement: S1 File — (ZIP) [file pgph.0001625.s001.zip › Community/VIWA-IDI-UHTN-200710_0535.docx]

**Moderator: {Name}**

**Code:** **VIWA-IDI-UHTN-200710_0535**

**Moderator:** This community has been identified to have a high burden of uncontrolled hypertension which is a leading factor to premature deaths and disability. I am trying to gather information about hypertension care in your community. To avoid hypertension related complications, it is recommended that people with high blood pressure can change their lifestyles in regards to diet, physical activities, smoking, alcohol consumption and using blood pressure medication. So tell me about your experience with having high blood pressure. Kindly tell me about your experience with having high blood pressure

**Respondent: Number one is that I am supposed to take drugs as instructed by the doctor, the second one is that I should eat foods that I am advised to eat by the doctor like for example I was told not to take sugar and to avoid excess salt in food and I am also supposed to do exercise. So far I can see that I am progressing well since the day that I was diagnosed with this condition and I don’t feel like I have a big problem. I was also told that I should avoid thinking too much**

**Moderator:** Ok, for how long have you been having high blood pressure?

**Respondent: For almost six years I think**

**Moderator:** Six?

**Respondent: Yes, I think so**

**Moderator:** How often do you check your blood pressure?

**Respondent: I normally go for clinic like for now I’ll go in August but I attend my clinic in a monthly basis**

**Moderator:** Next month?

**Respondent: Yes, but I was told to go after three months**

**Moderator:** Do you record your blood pressure measurements when you go to for your clinics?

**Respondent: Yeah**

**Moderator:** What was your last blood pressure reading?

**Respondent: I can’t get that book but I was told that it is normal**

**Moderator:** Ok, you told me that you don’t have any other problem apart from high blood pressure?

**Respondent: Yes but the only problem that I might be having is malaria but malaria is a condition that affects everybody but I don’t have any other condition**

**Moderator:** Ok. Has your doctor ever told you what your target blood pressure reading should be?

**Respondent: I think it should be below 140/90**

**Moderator:** Your doctor told you that it should bellow 140/90?

**Respondent: Yes**

**Moderator:** Ok, tell me about your antihypertensive drugs

**Respondent: I used to take HCT and another one called artenal**

**Moderator:** Has the number of medication changed from the day you were diagnosed with high blood pressure?

**Respondent: I was given three different types of drugs that I was swallowing one of each on a daily basis but for now am taking two**

**Moderator:** Ok, did the doctor tell you the reason as to why he did that?

**Respondent: I was told that it was because my blood pressure was neither very high nor very low, that’s the reason that I was given**

**Moderator:** Ok, how has high blood pressure condition affected your life?

**Respondent: Sometimes back, I could not see very well, I could also feel tired and sleepy while at work but when I followed my doctor’s advice on diet and exercising even though I am old I can see that God is helping me and am doing better**

**Moderator:** Apart from medication, how do you manage your high blood pressure condition?

**Respondent: There is nothing else apart from exercising and taking fruits, there is nothing else that I am doing to manage my blood pressure**

**Moderator:** You told me that you were asked to reduce on your sugar intake and you are doing exercise and reduce salt

**Respondent: Yes and I was told to reduce my meat consumption**

**Moderator:** Ok, who do you see when you go to the hospital?

**Respondent: I see a doctor called {Name}**

**Moderator:** What did she tell you, is she a nurse or?

**Respondent: I cannot tell because I go to the health Centre clinic managed by the city council so I don’t know if she is a nurse or a doctor but I can say that she is a doctor**

**Moderator:** You told me that you see a doctor when you go to the hospital

**Respondent: Yes**

**Moderator:** So what can you tell me about your health care provider in regards to how is she managing your high blood pressure condition?

**Respondent: When I go there, they take my blood pressure measurements, my weight and my height then I am told on how I am supposed to continue taking drugs**

**Moderator:** So what is your view on how your health care provider is managing your blood pressure condition?

**Respondent: I can say that she is doing well because I have never gone to another hospital and she told me that my blood pressure is controlled unless there is another way or if you can advise me on any other way, I’ll still accept your advice**

**Moderator:** So you told me that you don’t go elsewhere apart from the health Centre Clinic?

**Respondent: Yes, the health center**

**Moderator:** Ok. Where else can you get hypertension care in your community?

**Respondent: There is a clinic called {Name of the facility}, that’s where I used to go but I was advised to go to the health Centre because of treatment costs. I get free treatment at the health Centre**

**Moderator:** What kind of services do you receive at the health Centre?

**Respondent: I told you that when I go there the first thing they do is to check my blood pressure measurements, height then I go to the doctor who tells me my blood pressure readings, he give me drugs and then I leave. That’s all**

**Moderator:** You told me that you changed from access to The health Centre because they are giving drugs for free

**Respondent: Yes, exactly**

**Moderator:** Also that you are served better and the doctors at The health Centre advise you well on how your blood pressure should be

**Respondent: Yes**

**Moderator:** And you also told me that you go there on a monthly basis

**Respondent: Yes, I used to go there every month but when COVID19 came that’s when I was given drugs that could take me for three months**

**Moderator:** Is there any other hindrance in managing blood pressure apart from what you tome me that you are stressed?

**Respondent: No, there is no other, for me I just take medicine as instructed and running a little bit**

**Moderator:** As an individual, tell me what you can do differently to manage your blood pressure

**Respondent: According to what we are told, blood pressure is not a condition that can be treated; I was told that I will leave with it so it depends on how I am taking drugs. That’s what I was told and I know that I just need to take my drugs but you can inform me in case there is another way**

**Moderator:** Are there any communal problems that make you not be able to manage your blood pressure?

**Respondent: It can be there but I have never thought that way**

**Moderator:** What of your health care provider? Are there factors that hinder you from managing your blood pressure that are caused by your health care provider?

**Respondent: Based on what I was told, blood pressure cannot be treated but it can only be controlled so I have never thought of anything that my doctor can do to manage my blood pressure and so I have just decided to take my drugs**

**Moderator:** Is there anything that can be done differently at the facility for you to be able to get your hypertension care services better?

**Respondent: I don’t see but maybe if I go to a different facility like {Name} where I will be forced to pay but I have never thought of any other thing apart from going to the hospital**

**Moderator:** Is there a problem with the time that you go there for treatment?

**Respondent: There is no problem**

**Moderator:** What about drugs? Are there times when there are no drugs?

**Respondent: Yes, there are times that you can go to the hospital and the doctor tells you that there are no drugs or maybe you get one and you are told to go buy the other one. That challenge is there**

**Moderator:** How is the quality of treatment?

**Respondent: I can’t see anything that is bad because I have not tried somewhere else**

**Moderator:** Ok, what about the costs of treatment?

**Respondent: I told you that I go to a hospital managed by the city council called The health Centre. This is a public dispensary, and I am not charged when I go there but we are asked to buy if there other drugs that are not available**

**Moderator:** How is the patients’ treatment space at the hospital?

**Respondent: When we go for clinic like I told you that I will be going after three months, hypertension patients are always given first priority then the other patients are treated when we have left**

**Moderator:** What can we do to solve the problems that you have mentioned? You told me that there are times when there are no drugs. Please tell me about what can be done to solve that problem

**Respondent: When we miss drugs we are told that the hospital receives drugs from the government but if they have not received the drugs then us patients will not be able to get the drugs and we are forced to go buy but if the government has supplied I don’t see if they can deny us but because they have not received from the government then they will not give us**

**Moderator:** On individual factors, what can you do differently as an individual to manage your blood pressure?

**Respondent: I don’t see any other thing apart from taking drugs and following what I am told. That’s what I was told can be done to manage blood pressure but as an individual, I think I should avoid thinking because I was told that blood pressure goes high when I think so much so I try avoiding that. When I get annoyed at my family I just walk out and come back when I am ok**

**Moderator:** How has COVID19 affected hypertension service delivery in your community?

**Respondent: We used to go to clinic after every one month but now with COVID we were told that there should be no gatherings and that’s why we were given drugs that can last for three months so that we can go back after the three months because there are always many people at the hospital. That’s what we were told and we also know that we cannot sit close to each other because of COVID19**

**Moderator:** Is there anything else that you feel that we have not talked about and you feel that we should talk about in regards to high blood pressure?

**Respondent: Yes, I heard you asking me a question if there is anything that I as {Name} can do or if there is any other way that I can follow so that I can manage my condition completely. What can you tell me about that? Could be there any other way because for me what I was told by the doctor is that I can only do that by taking drugs, dieting which cannot be available always because sometimes there is no money. Tell me if there is anything else I can do to control my blood pressure condition**

**Moderator:** What I can tell you is that I can only refer you to your health care provider at The health Centre. When you go there after they have measured your blood pressure and advised you, it will be better for them to tell you why your blood pressure is not controlled and you shouldn’t fear your doctor, just ask her where the problem is and if the issue is drugs then they will tell you, if it is about lifestyle I think they are in the best position to tell you what you can do in regards to your condition

**Respondent: Ok**

**Moderator:** For me I can’t know because I have not been with you for a long period

**Respondent: That’s true**

**Moderator:** They are the ones who can tell you because they have been with you from the day that you were diagnosed, you have been going there, they have given you drugs and if at all you have any question it will be better if you went to your health care provider and asked her why she has been giving you three types of drugs and why she has changed to two for you to know. You must have that urge to ask coz its bot bad to ask; actually the doctor will see that you have that urge to know why your blood pressure is not ok. That’s all I can tell you

**Respondent: Ok**

**Moderator:** Any other question?

**Respondent: You asked me what I can do in regards to my community to manage my blood pressure**

**Moderator: I’ll just take you back to your health provider coz she is in a better position to tell you everything**

**Respondent: I will ask**

**Moderator: Thank you so much for taking part in this research that we are doing and we appreciate for your time**

**Respondent: Me to**

**Moderator: This will be listened to by those that are supposed to listen and address the gaps**

**Respondent: Ok**

**…END…**
