# Supplementary material for: A socio-ecological framework examination of drivers of blood pressure control among patients with comorbidities and on treatment in two Nairobi slums; a qualitative study
Source: PLOS Glob Public Health. 2023 Mar 10;3(3):e0001625. doi: 10.1371/journal.pgph.0001625 (PMC10021823; doi:10.1371/journal.pgph.0001625)
Supplement: S1 File — (ZIP) [file pgph.0001625.s001.zip › Community/VIWA-IDI-UHTN-200714_001.docx]

**Moderator: {Name}**

**Code:** **VIWA-IDI-UHTN-200714_001**

**Moderator:** This community has been identified to have a high burden of uncontrolled hypertension which is a leading factor to premature deaths and disability. I am trying to gather information about hypertension care in your community. To avoid hypertension related complications, it is recommended that people with high blood pressure can change their lifestyles in regards to diet, physical activities, smoking, alcohol consumption and using blood pressure medication. We will go to the questions, tell me how your experience has been in regards to having high blood pressure

**Respondent: That will be hard for me to explain based on the way you are talking**

**Moderator:** For how long have you been having high blood pressure?

**Respondent: It’s been a while since I was diagnosed with high blood pressure**

**Moderator:** Can you tell me the number of years?

**Respondent: It was diagnosed when I was having a little baby and I didn’t have something to eat at that time. That drug almost killed me**

**Moderator:** For how long have you been having high blood pressure?

**Respondent: From that time like 10 years. I stopped taking drugs and the condition made me weak and I stopped giving birth and started using my drugs but the drugs have made me weak, I cannot stand, one leg is not functioning and it is like a shock on one side but I am getting better from the time I started taking drugs again. It is not like before when I used to fall down all the time. I also don’t have teeth due to pressure condition that I have been having for more than 10 years**

**Moderator:** Where do you check your blood pressure?

**Respondent: At the health Centre. I went and my teeth were extracted because of pressure shock and I don’t have teeth on one side. They gave me drugs that I ate, and they have affected my teeth and I went back to the health Centre**

**Moderator:** How frequent do you go to the health Centre to check your blood pressure measurements?

**Respondent: For now I go after two months because of corona**

**Moderator:** Do you record somewhere?

**Respondent: No I don’t record**

**Moderator:** Can you remember your last measurement readings

**Respondent: I don’t know how to write and my doctor didn’t tell me, he just tell me that my blood pressure is low or if it is ok and he gives me the medicine**

**Moderator:** Has your doctor told you that a certain blood pressure measurement is ok?

**Respondent: I was measured at {Name of the facility} and I was told that my blood pressure was very high and they gave me drugs that I swallowed though they closed and I decided to go back to the health Centre.**

**Moderator:** When you go to clinic, have you ever been told what your normal blood pressure should be?

**Respondent: It is 180**

**Moderator:** Do you have any other condition apart from high blood pressure?

**Respondent: I have a problem with my stomach but I don’t know if it is related to pressure. I have been having this stomach problem for a very long period**

**Moderator:** You told me that you have been having high blood pressure for like 10 years

**Respondent: Yes**

**Moderator:** Have you been using medicine for the 10 years

**Respondent: No just this year, I used drugs at first and I used to feel weak and so I stopped taking because I didn’t have something to eat then. I might be thinking that the drugs made me weak yet it could be because I didn’t have food. That’s why I stopped**

**Moderator:** Which type of drug are you using for now?

**Respondent: For now I don’t know the name but I am using a drug that is in a box together with other small tablets**

**Moderator:** How many types of drugs are you using?

**Respondent: Am using two**

**Moderator:** Did you start with the two drugs when you were diagnosed with blood pressure?

**Respondent: No they are not the ones that I was given at first**

**Moderator:** Compared to that time and now, would you say that you drugs have increased or reduced?

**Respondent: They have reduced**

**Moderator:** How has this blood pressure condition affected you?

**Respondent: It has affected me, my legs are swollen, they are weak and I have headaches every now and then, I don’t feel well to an extend that I spend most of my time sleeping and I can’t do my work**

**Moderator:** Apart from taking drugs, how else do you manage your blood pressure condition?

**Respondent: I avoid noisy places and I also try not to get angry. I just walk pot when my kids quarrel**

**Moderator:** What about diet?

**Respondent: I try eating traditional foods, I was told not to take tea. There many things that I was told not to eat**

**Moderator:** Can you tell me?

**Respondent: I was told not to take sugar, alcohol, cigarettes. They are many things that I have been told not to get close to and I have already told you**

**Moderator:** What about exercise?

**Respondent: Yeah, I was told to do exercise. Something like that**

**Moderator:** Who do you see when you go to the health Centre?

**Respondent: I don’t know the name**

**Moderator:** Do you see a doctor or a nurse

**Respondent: I see a doctor who checks my pressure then I take my drugs and leave**

**Moderator:** What can you say in regards to the way he is managing your condition?

**Respondent: I feel good because I can now walk and I can eat. I am ok as per now**

**Moderator:** How is your doctor managing you?

**Respondent: He is ok. He serves me well. He checks my blood pressure, gives me drugs then I leave**

**Moderator:** Have you gone elsewhere apart from the health Centre clinic?

**Respondent: Yeah, I went and found out that they had relocated. I suffered for two weeks, and I decided to go back to the health Centre**

**Moderator:** Ok

**Respondent: Later on they sent me a message informing me that they have relocated to a another place that I should go but I didn’t follow up**

**Moderator:** Where else had you gone?

**Respondent: At {Name of the facility} where I was being served at first, but I went there and found that they had demolished the building because it was built on a road reserve. I didn’t know where they had relocated to and when I asked, and I couldn’t know where they moved to then I decided to go back to the health Centre**

**Moderator:** What kind of services do you receive at the health Centre?

**Respondent: Only high blood pressure care services. They checked my blood pressure measurement and gave me drugs. I just gave them a paper and told them that I used to go to a hospital that I don’t know where it was relocated to and they started serving me then**

**Moderator:** Do you pay for the drugs that you receive there or you are given for free?

**Respondent: No, they are just ok**

**Moderator:** Without payment?

**Respondent: Yes**

**Moderator:** You told me that you were told to go for clinic after two weeks due to Corona?

**Respondent: Yes, after two months**

**Moderator:** How often do you go when everything is normal?

**Respondent: Because of Corona we are just given drugs and we told to go back after to months**

**Moderator:** How were you going for clinics before Corona came?

**Respondent: I just went for one clinic and on that day and on the very day I found them telling people not to sit close to each other**

**Moderator:** Am asking how you going for clinics before Corona came?

**Respondent: It was the only clinic that I attended there the same day the doctor told us that Corona was in Kenya; I told you that access afya had relocated. We were told that there is another new condition and people should not sit close to each other**

**Moderator:** Are you advised when you go to that clinic?

**Respondent: We were not advised because of that disease, we were told to keep distance as we collected drugs and left**

**Moderator:** Do you have any problem in managing your blood pressure?

**Respondent: Yes, I have a problem with my stomach; there is also a high blood flow when menstruating**

**Moderator:** Looking at the drugs and the costs, do you have insurance card?

**Respondent: No, I don’t have**

**Moderator:** You are able to sort all the payments?

**Respondent: I don’t have that ability, but I try**

**Moderator:** Looking at your age, is it a hindrance in managing your blood pressure?

**Respondent: When you have kids at school it becomes a challenge in managing your age and the condition. You get shocked when you are told that your child needs something or school fees and the pressure goes up when you are shocked, that’s the problem that I have**

**Moderator:** You had told me that you take two types of drugs

**Respondent: Yes**

**Moderator:** Do you take them as they are supposed to be taken?

**Respondent: I take two at night and I go to bed because I cannot even stand after taking them**

**Moderator:** And you take them as instructed by the doctor?

**Respondent: Yes**

**Moderator:** Do you just sit in the house or you do go out?

**Respondent: I walk when am feeling well and if I dint feel well then I just sit but it is controlled not like before when I couldn’t walk. I just sit when I feel like am not ok**

**Moderator:** Looking at your family and your community are there any factors that are hindering you from managing your blood pressure?

**Respondent: There is a problem with my family especially my husband. I cannot take care of myself with my condition and sometimes my kid’s condition becomes worse. That’s the problem**

**Moderator:** What about food that is not good for you in relation to the family

**Respondent: No**

**Moderator:** Looking at your doctor or the nurses, are they hindering you from managing your blood pressure?

**Respondent: I have no problem but I was not advised. We just took the drugs and left without being advised**

**Moderator:** And he attends to you well

**Respondent: Yes, he attends to me well; he checks my blood pressure and gives me drugs before I leave**

**Moderator:** Do you get all the drugs when you go to the health Centre hospital?

**Respondent: I have taken drugs twice at the health Centre**

**Moderator:** Is the space at the health Centre enough for you?

**Respondent: The sitting space is ok. We always keep distance**

**Moderator:** Looking at the government, is it hindering you from managing your blood pressure?

**Respondent: The government I caring for me well, because I have not seen any problem. I just went there twice and I was given my drugs. I just don’t understand what you are saying**

**Moderator:** We want to look at the solutions to the problems that you have mentioned. I’ll read to you the challenges that you mentioned

**Respondent: Ok**

**Moderator:** You told me that your legs are swollen and you don’t feel well, what do you think would be the solution to that?

**Respondent: I don’t know what could be the problem with my legs. I just think that it is because of my blood pressure condition. I just have a problem with my stomach then the legs but I don’t know where the problem comes from**

**Moderator:** You also told me that you have a problem in getting money to buy food, what could be the solution to that?

**Respondent: There is a problem with my child and my husband has a problem that he cannot manage my problem so I just leave it to God because there is nowhere else I can take that problem**

**Moderator:** You also said that there is a problem with your family. What can we do to solve that?

**Respondent: You know I have a kid but if it was not for the kid then I would have left and moved to a place where I can manage. The issue is that I cannot leave the child; I just have to struggle because the time is not ripe for me to leave**

**Moderator:** Looking at you as an individual, what can you do differently to manage your condition?

**Respondent: For now I can say that I have controlled but I don’t know what the issue was before because I used to fall down every time. I cannot sit where people are quarrelling; I avoid getting annoyed because of blood pressure. I just sit down by the roadside when I feel dizzy and headache the time that I go out for a walk alone**

**Moderator:** What can your doctor at the health Centre do differently in regards to your blood pressure?

**Respondent: He did not tell us anything because of Corona. We were just checked and then we left**

**Moderator:** What can be done differently at the health Centre health center?

**Respondent: There are somethings that I don’t understand; your Swahili is very hard like for now I don’t get what you are saying**

**Moderator:** Ok, we are looking at your hospital at the health Centre

**Respondent: Yes**

**Moderator:** What can they do differently in relation to blood pressure?

**Respondent:** I cannot understand that

**Moderator:** How has COVID19 affected how you get your hypertension care services in your community? You told me that you were told to go to the hospital after two months because of Corona, how has that affected the way you have been receiving hypertension care services?

**Respondent: They serve me well. I just take my drugs and I leave. There are somethings that I don’t understand I can’t say a lie**

**Moderator:** I was asking how COVID19 affected how you get your hypertension care services or I put it this way, How have you been affected now that you have been asked to go to clinic after two months

**Respondent:** It is ok just okay because there is Corona and it is not our fault or the facility’s fault. They said that because of the regulations that we should keep distance. They had to give us different dates so that we don’t come close

**Moderator:** Is there anything else that you would want us to talk about in regards to high blood pressure?

**Respondent: No**

**Moderator: Ok**

**Respondent: There was nothing like talking to us on blood pressure, they just checked the blood pressure and asked to leave**

**Moderator:** Ok, thank you so much for your time and the information that you have given me and I hope that it will help us in our research

**Respondent: Ok**

**…END…**
