# Supplementary material for: A socio-ecological framework examination of drivers of blood pressure control among patients with comorbidities and on treatment in two Nairobi slums; a qualitative study
Source: PLOS Glob Public Health. 2023 Mar 10;3(3):e0001625. doi: 10.1371/journal.pgph.0001625 (PMC10021823; doi:10.1371/journal.pgph.0001625)
Supplement: S1 File — (ZIP) [file pgph.0001625.s001.zip › Community/VIWA-IDI-UHTN-200714_002.docx]

**Moderator: Catherine Kahare**

**Code:** **VIWA-IDI-UHTN-200714_002**

**Moderator:** This community has been identified to have a high burden of uncontrolled hypertension which is a leading factor to premature deaths and disability. I am trying to gather information about hypertension care in your community. To avoid hypertension related complications, it is recommended that people with high blood pressure can change their lifestyles in regards to diet, physical activities, smoking, alcohol consumption and using blood pressure medication. So tell me about your experience with having high blood pressure. Kindly tell me about your experience with having high blood pressure

**Respondent: It has really troubled me**

**Moderator:** For how long have you been having this condition?

**Respondent: For like 10 years**

**Moderator:** Where do you go to check your blood pressure measurements?

**Respondent: Up here at the stage**

**Moderator:** How often do you check your blood pressure measurements?

**Respondent: Once per month but when I feel like I have a problem I go once per week**

**Moderator:** Do you keep record of your blood pressure measurements?

**Respondent: No, I just leave once I have been measured**

**Moderator:** What were the measurements the last time you checked?

**Respondent: Maybe I confirm**

**Moderator:** Have you been told by your doctor your target blood pressure measurements

**Respondent: He told me that it is supposed to be 130/90**

**Moderator:** Ok, do you have any other condition apart from high blood pressure?

**Respondent: No**

**Moderator:** Do you use antihypertensive drugs?

**Respondent: Yes**

**Moderator:** Tell me about the drugs

**Respondent: I used to take the red one but it had complications on me, I could feel dizzy but am now using amroza H (3:17)**

**Moderator:** You told me that you have had this condition for like 10 years, did you start medication immediately or how was it?

**Respondent: I didn’t start immediately because I didn’t even know what it was. My head was aching at day time and at night then I decided to go confirm**

**Moderator:** When did you start taking drugs?

**Respondent: Not long ago, I started this year**

**Moderator:** You told me that there is this medicine that had complications on you and it was changed

**Respondent: Yes, I was given the drug at olive link then I could feel like it was not ok with me then I went to {Name of the facility} where I was given Amroza H**

**Moderator:** How many tablets have you been taking from the time you started taking that drug or how do you take it?

**Respondent: Once per day**

**Moderator:** Have you been taking it that was since you started taking drugs?

**Respondent: Yes, once per day**

**Moderator:** How has high blood pressure affected you?

**Respondent: It has affected me coz sometimes because I can’t do my daily work like as am supposed to do**

**Moderator:** How do you manage your blood pressure condition apart from using drugs?

**Respondent: Dieting, there is a way I was advised to eat. I was also told to take vegetables more and fruits. That is the reason as to why it balances and sometimes it comes to 128**

**Moderator:** What else?

**Respondent: I don’t work a lot, I was told to be walking for some time then I come back**

**Moderator:** How is your normal day?

**Respondent: It is not that good but at least**

**Moderator:** Do you leave the house?

**Respondent: Yeah, I do leave and go for a walk just like I was told to**

**Moderator:** Do you use traditional medicine?

**Respondent: No I have never**

**Moderator:** You told me that nowadays you go to {Name of the facility}

**Respondent: Yes**

**Moderator:** Who do you see when you go there?

**Respondent: I see a doctor**

**Moderator:** Is he a doctor or a nurse?

**Respondent: I don’t know if he is a doctor coz I can’t tell. It is a big hospital**

**Moderator:** What can you say about how your health care provider is serving you?

**Respondent: He is ok because since he gave me those drugs, there are times that my blood pressure reads 128 and I don’t feel headache**

**Moderator: What made you to start going to {Name of the facility**

**Respondent: ? There is a day that you were measuring us at Lunga lunga stage and I found out that my blood pressure was high despite the fact that I was on medication and then one of my friends advised me to go there**

**Moderator:** What services do you receive when you go to {Name of the facility}?

**Respondent: They just check my blood pressure readings, they used to check my headache condition but nowadays I no longer feel headache and they told me that I think so much and so they gave me drugs that can make me sleep**

**Moderator:** Are you given drugs for free or you do buy?

**Respondent: I do buy, what else can I do?**

**Moderator:** What about advice?

**Respondent: They counsel me and tell me to avoid thinking so much because I used to have a lot of problems before I was diagnosed with hypertension condition**

**Moderator:** How often do you go for clinic?

**Respondent: I go monthly**

**Moderator:** Do you have any problem in managing your blood pressure?

**Respondent: No**

**Moderator:** Looking at you as an individual, you told me that you buy your drugs

**Respondent: Yes**

**Moderator:** Do you have insurance?

**Respondent: No. I just try get like 200 bob then I go for drugs**

**Moderator:** Looking at age, is it a hindrance in managing your blood pressure?

**Respondent: No**

**Moderator:** What about the way you are taking medicine?

**Respondent: No**

**Moderator:** You told me that you normally leave your house

**Respondent: Yes**

**Moderator:** Do you use alcohol or cigarettes?

**Respondent: No, have never used them**

**Moderator:** Looking at your family, is it hindering you from managing your blood pressure condition?

**Respondent: I am the one who takes care of my self**

**Moderator:** Do you take the food that you are advised to take?

**Respondent: Yes, I try**

**Moderator:** Looking at your health care providers at {Name of the facility}, are they hindering you from managing your blood pressure condition?

**Respondent: No they are ok**

**Moderator:** How are the clinic hours there?

**Respondent: We are not many patients there because of the current situation. I have to call my doctor first to confirm if he is available**

**Moderator:** What about the drugs that you buy at {Name of the facility}, do you always get them?

**Respondent: Yes, there is a chemist there, sometimes the doctor tell me to go to the chemist when I don’t get drugs at the hospital**

**Moderator:** What about the space at the hospital, is it enough

**Respondent: It is not bad**

**Moderator:** Looking at the policies, do you see that there is a problem in managing high blood pressure patients

**Respondent: At {Name of the facility}?**

**Moderator:** Yes, on the government side

**Respondent: There is nothing that the government is helping us with because if I don’t have money to buy drugs then I’ll just stay without them and ask God to take care of me. They are supposed to give out hypertensive drugs for free just the same way they do with TB. We just ask God to take care of us when you cannot afford the drugs because they are expensive**

**Moderator:** What could be the possible solution to the hindrances that you mentioned? You talked about drugs that you go to buy at the chemist when you don’t get them at the hospital, what can we solve that?

**Respondent: You are the one to tell me how you can help me**

**Moderator:** I wanted to get your view

**Respondent: On drugs?**

**Moderator:** On money, what could be the solution to that? Because you have to buy drugs when they are not available at the hospital

**Respondent: I just give up when I don’t have money and my business can’t manage to provide the money that I want**

**Moderator:** What do you think would be the solution?

**Respondent: The government should help patients with hypertensive condition because they are the ones that die more**

**Moderator:** Ok, looking at you as an individual, what can you do differently?

**Respondent:** At the house

**Moderator:** In regards to blood pressure issue

**Respondent: There is nothing I can do**

**Moderator:** What about your health care provider?

**Respondent: Health care providers help me where they can**

**Moderator:** How has COVID19 affected health care service delivery to hypertensive patients in your community? You told me that you have to make a call to confirm if the doctor is available before you go to the hospital

**Respondent: Yes**

**Moderator:** How is that affecting you from receiving your health care service?

**Respondent: It has affected me because sometimes I have to walk to {Name of the facility} when I don’t have airtime and I just come back when I find that he is not available**

**Moderator:** Is there anything else that you would want us to talk about in regards to high blood pressure?

**Respondent: The way a person can assist himself like for example on high blood pressure. How can you advise us to do so as to manage our blood pressure?**

**Moderator:** Thank you for your time and thank you for the conversation that we have had and I know that it will help us in our research. Thank you

**Respondent: And if I may ask, where are you located currently**

**Moderator:** For now am at the office

**Respondent: Where Exactly?**

**Moderator:** At Kitusuru

**Respondent: Where is Kitusuru?**

**Moderator:** Kitusuru is in Kiambu County, it is not far

**Respondent: Kiambu County**

**Moderator:** Yes

**…END…**
