# Supplementary material for: A socio-ecological framework examination of drivers of blood pressure control among patients with comorbidities and on treatment in two Nairobi slums; a qualitative study
Source: PLOS Glob Public Health. 2023 Mar 10;3(3):e0001625. doi: 10.1371/journal.pgph.0001625 (PMC10021823; doi:10.1371/journal.pgph.0001625)
Supplement: S1 File — (ZIP) [file pgph.0001625.s001.zip › Community/VIWA-IDI-UHTN-200714_2253.docx]

**Moderator: {Name}**

**Code:** **VIWA-IDI-UHTN-200714_2253**

**Moderator:** This community has been identified to have a high burden of uncontrolled hypertension which is a leading factor to premature deaths and disability. I am trying to gather information about hypertension care in your community. To avoid hypertension related complications, it is recommended that people with high blood pressure can change their lifestyles in regards to diet, physical activities, smoking, alcohol consumption and using blood pressure medication. So tell me about your experience with having high blood pressure. Kindly tell me about your experience with having high blood pressure and how you have been from the day that you were diagnosed with high blood pressure

**Respondent: Its three months now since I was diagnosed**

**Moderator:** How have you been lining with this high blood pressure condition?

**Respondent: It is not good with me coz I was in a very bad situation but I went to {Name of the facility}**

**Moderator:** Is that where you were told that you are hypertensive?

**Respondent: Yes, but it is three months since I was told that**

**Moderator:** How often have you been checking your blood pressure measurements within the three months that you have been hypertensive?

**Respondent: I go to a hospital that is located after {Name of the facility} and I was told that I should go back for checkup once I finish taking my drugs but its two weeks now since I finished my drugs but I have never gone back for check up**

**Moderator:** Ok

**Respondent: I was planning to go back tomorrow for check up**

**Moderator:** Have you been recording your blood pressure measurements after you have been checked?

**Respondent: Yes**

**Moderator:** What were the measurements the last time you checked?

**Respondent: I can’t remember the last one; it is recorded on a card**

**Moderator:** They write for you on a card?

**Respondent: Yes, and I left the card there but I was told to go back after two weeks for checkup. I am planning to go there tomorrow**

**Moderator:** You told me that you only have high blood pressure condition?

**Respondent: Yes and my legs are also painful**

**Moderator:** Has your doctor ever told you what your target blood pressure should be?

**Respondent: The last time the doctor told me that it was low but he told me to go {Name of the facility} to be checked up on my legs issue**

**Moderator: Ok**

**Respondent: But I came to realize that this legs issue is brought up by the hypertensive condition**

**Moderator:** Ok, so the doctor has never told you what your target blood pressure should be?

**Respondent: No but I’ll be going there tomorrow**

**Moderator:** Ok .So how many tablets have you been taking in the last three months?

**Respondent: Three**

**Moderator:** Have they been reducing or adding your drugs for the three months as you have been going to the hospital?

**Respondent: It has been reducing**

**Moderator:** How many tablets did you start with?

**Respondent: Its only three months since I started taking drugs**

**Moderator:** How many are you taking now?

**Respondent: I don’t have drugs for now. I finished drugs and there is nothing that I am using**

**Moderator:** How has high blood pressure affected your life?

**Respondent: I can’t answer that**

**Moderator:** What is it that you can do or what have you been unable to do from the time that you were told that you are hypertensive?

**Respondent: I have been told to avoid somethings like I was told to reduce sugar, they are many things. I just did as I was advised by the doctor**

**Moderator:** What else do you do to manage your blood pressure apart from using drugs?

**Respondent: I don’t know how this condition is and that why I am planning to go there tomorrow so that they can tell me how I can stay and the way I’ll be taking drugs so that I can manage my blood pressure**

**Moderator:** You said that you want to go to the hospital that is after {Name of the hospital}?

**Respondent: Yes or {Name of the facility}. I go to those two hospitals**

**Moderator:** Who do you see when you go for clinic?

**Respondent: I see a doctor**

**Moderator:** What can you tell me about this doctor in regards to the way he is managing your blood pressure condition?

**Respondent: you mean the doctor?**

**Moderator:** Yes

**Respondent: I don’t know his name**

**Moderator:** You don’t have to know his name; I just wanted to know how he is managing your blood pressure condition

**Respondent: He gave me drugs and he is also the one who told me to go back so that he can see how I am progressing**

**Moderator:** You told me that you have been going to another hospital apart from the one that you go to, why did you go to {Name of the facility} and not where you used to go before?

**Respondent: I just decided to go to a hospital that is close to my place**

**Moderator:** So you thought of going there because it is close to you?

**Respondent: Yes, it is so close**

**Moderator:** Ok, what kind of services do you receive when you go to {Name of the facility}?

**Respondent: They give me drugs**

**Moderator:** What else?

**Respondent: Only drugs, I have never been injected, I just take the drugs**

**Moderator:** They only give you drugs?

**Respondent: Yes, just drugs then I pay and go home**

**Moderator:** What about teachings, have they given you any teachings in regards to blood pressure?

**Respondent: I was taught at the facility how I should live and what I am supposed to use**

**Moderator:** How often are you supposed to go there?

**Respondent: I was told to be going on the day that I am booked**

**Moderator:** From the time you started going, would you say that you go there monthly or weekly or how have you been going?

**Respondent: I was going there on a weekly basis**

**Moderator:** Every week?

**Respondent: Yes and I go there when I finish my medicine**

**Moderator:** So they give you drugs that can take you for one week then you go cack when you finish them?

**Respondent: Yes. And they exchange my drugs**

**Moderator:** What challenges have you come across as you try to manage your blood pressure condition?

**Respondent: You mean before I knew that I have this condition?**

**Moderator:** Yes

**Respondent: I was feeling bad, sometimes I used to feel like am falling down and other times I could feel like vomiting and that’s what made me to go to the hospital and that’s when I was told that I have this condition**

**Moderator:** Ok. What challenges do you encounter as you try to manage your blood pressure condition?

**Respondent: Maybe you tell me coz it is like I am confused**

**Moderator:** What are the individual factors that hinder you from managing your blood pressure condition?

**Respondent: For it to be normal?**

**Moderator:** Yes

**Respondent: Just tell me what I am supposed to do for my blood pressure to be normal**

**Moderator:** You told me that you are going for clinic tomorrow?

**Respondent: Yes**

**Moderator:** It would be better for you to ask your doctor and inform him about your condition for him to tell you and also advice you about hypertension, how you are supposed to stay, what you are supposed to eat because he is the one that will be attending to you all the time and you will be having questions every time you go there

**Respondent: Tomorrow**

**Moderator:** Yes, when you go there tomorrow

**Respondent: Ok**

**Moderator:** Now tell me about the cost of drugs and the cost of you attending clinics

**Respondent: The money that I use?**

**Moderator:** Yes

**Respondent: It is a lot**

**Moderator:** You told me that you are 50 years

**Respondent: Yes but I have used a lot of money since the day that I was diagnosed with this condition and I don’t have that ability**

**Moderator:** What about the way you take medicine? Are you able to remember or sometimes you do forget to take drugs?

**Respondent: I would buy if I had money but there are some drugs that they prescribe but I don’t buy because I don’t have money**

**Moderator:** I buy drugs in small portions because I don’t have money and there is nothing that I can do

**Respondent: Drugs cost like 400 shillings or 500 shillings and I have like 200 shillings at that time and so I am forced to buy drugs worth 200 shillings and I am left with a deficit of like a half a dose**

**Moderator:** What are the communal or family factors that might hinder you from managing your blood pressure condition?

**Respondent: Its better when one has money, there some questions that I cannot answer because I don’t have money. Sometimes I lack money to buy food like for now I just took porridge in the morning and I have not taken food. I have a lot of problems**

**Moderator:** Ok, looking at the health center, what are they doing that might hinder young from managing your blood pressure? You told me that there are times that you don’t get drugs and you have to buy

**Respondent: You just use what you have because you cannot use what you are told to use yet you don’t have money to buy**

**Moderator:** Ok

**Respondent: If I had money I would buy what the doctor advices me to use**

**Moderator:** You also told me that there is no one at the hospital to teach you about pressure

**Respondent: I will know what to do when I go there tomorrow**

**Moderator:** Ok

**Respondent: He will check me and tell me how I am fairing on then you call me later because I will go there tomorrow in the morning**

**Moderator:** What about the government? What is it that you think they are not doing that is hindering you from managing your blood pressure?

**Respondent: Some questions are hard for me to answer**

**Moderator:** What is it that the government is not doing and it is supposed to do so as to manage your blood pressure condition?

**Respondent: They should send me money so that I can go to the hospital and buy the things that I am advised to use. I am told to buy many things yet I don’t have money**

**Moderator:** Ok, you have said that they should send you money, what else do you think that the government should do?

**Respondent: Just what I told you. The government should send me money to buy drugs that are subscribed and foods that I am supposed to eat because I have a lot of problems**

**Moderator:** As a hypertensive patient, what can you do differently from what you are doing now so that you can manage your pressure?

**Respondent: I have many thoughts due to lack of money and food. Financial problems are the ones that make my blood pressure to rise since I don’t have money to use at home. I am told that hypertensive patients receive money but I have never received any**

**Moderator:** Who told you that hypertensive patients receive money?

**Respondent: Those patients that receive and personally I have never received any money for me to go to the hospital. I receive my help from my neighbors**

**Moderator:** Who told you?

**Respondent: People do tell me**

**Moderator:** At the place where you stay?

**Respondent: Yes. I was told that the money is sent to those people that are sick. I have been sick and I have not received anything yet and people move around writing names. I gave them my name and I have not yet received anything**

**Moderator:** Were you told the name of the person that has been sending them money? Where did they write their names?

**Respondent: They receive money. I have given my name to four people but I have never been sent anything**

**Moderator:** Where did the guys that were writing names came from?

**Respondent: There those that were coming from {Name of a place}, {A place}, {Name of a place} but I have not received anything from the time that I forwarded my name**

**Moderator:** I have never heard of that, actually you are the first person to tell me that there are people getting money and you have not received

**Respondent: Ok**

**Moderator: How has COVID 19 affected the way you as a hypertensive patient get your hypertension care?**

**Respondent: I just hear of it but I have not seen it or ever been infected by Corona. I have never tested positive the times that I have been going for tests**

**Moderator:** So it has not affected your hypertension care services like going to the hospital?

**Respondent: I used to go to the hospital where I was tested of COVID**

**Moderator:** Last question, is there anything else that you would want to talk about in regards to high blood pressure that you feel that we didn’t talk about?

**Respondent: For me I just want drugs because I was told that the pressure can go back to normal**

**Moderator:** So you only want drugs

**Respondent: I need to be helped with money because I don’t have money and the other one is that I continue with clinic so that my pressure can go back to normal**

**Moderator: Ok**

**Respondent: I want to go to the hospital tomorrow so that I can know how I am fairing on because its two weeks since I finished taking drugs and I have never gone back for checkup. I want to go so that I can be checked, be given drugs and advised on how I can live**

**Moderator:** Ok, thank you for your time for now and I hope that what you have told me will help us know where the problem is

**Respondent: Ok**

**Moderator:** Thank you

**…END…**
