# Supplementary material for: A socio-ecological framework examination of drivers of blood pressure control among patients with comorbidities and on treatment in two Nairobi slums; a qualitative study
Source: PLOS Glob Public Health. 2023 Mar 10;3(3):e0001625. doi: 10.1371/journal.pgph.0001625 (PMC10021823; doi:10.1371/journal.pgph.0001625)
Supplement: S1 File — (ZIP) [file pgph.0001625.s001.zip › Community/VIWA-IDI-UHTN-200721_004.docx]

**Moderator: {Name}**

**Code:** **VIWA-IDI-UHTN-200721_004**

**Moderator:** This community has been identified to have a high burden of uncontrolled hypertension which is a leading factor to premature deaths and disability. I am trying to gather information about hypertension care in your community. To avoid hypertension related complications, it is recommended that people with high blood pressure can change their lifestyles in regards to diet, physical activities, smoking, alcohol consumption and using blood pressure medication. So tell me about your experience with having high blood pressure. On to the questions, tell me about your experience with having high blood pressure

**Respondent: I just found myself with this condition and I can not deny**

**Moderator:** For how long have you been having this condition?

**Respondent: Like around 15 years**

**Moderator:** Where do you check your blood pressure measurements?

**Respondent: I just came there and went to {a place}**

**Moderator:** What do you mean when you say that you came there?

**Respondent: At your place**

**Moderator:** Can you mention the name?

**Respondent: {Name of the facility}**

**Moderator:** How frequent do you go for high blood pressure checkup?

**Respondent: Like after every two months**

**Moderator:** So you go for checkup after every two months?

**Respondent: Yes**

**Moderator:** Do you record your blood pressure measurements?

**Respondent: I used to have it but for now I don’t have it here**

**Moderator:** Can you remember your last blood pressure measurements

**Respondent: It was 87**

**Moderator:** Has your doctor told you what your normal blood pressure target should be?

**Respondent: He told me that it should be around 50**

**Moderator:** Do you have any other condition apart from high blood pressure?

**Respondent: No**

**Moderator:** Can you tell me about the drugs that you are using

**Respondent: I use the ones that I was given there and the ones that I was given at {a place}**

**Moderator:** Do you know them?

**Respondent: No**

**Moderator:** How do you take them?

**Respondent: Taking them?**

**Moderator:** Yes, how do you take them?

**Respondent: I take them in the morning and in the evening**

**Moderator:** How many drugs do you take?

**Respondent: They give me and I go back for other drugs once I have finished the ones that I had**

**Moderator:** Were you taking only one type or how many types were you taking?

**Respondent: One type**

**Moderator:** Have you been using the same drug for the 15 years that you have been having high blood pressure?

**Respondent: The drugs have been changing**

**Moderator:** When did you start using the drug that you are currently using?

**Respondent: In March**

**Moderator:** How has high blood pressure affected you?

**Respondent: You get angry when someone offends you but you just relax because you know your status**

**Moderator:** How do you manage your blood pressure condition apart from using medicine?

**Respondent: There is nothing else that I do**

**Moderator:** What about diet?

**Respondent: With diet I was told to avoid eating meat and eat terere, managu and such types of vegetables**

**Moderator:** What about doing exercise?

**Respondent: I do exercise**

**Moderator:** Who do you see when you go to {a place} or {Name of the facility}?

**Respondent: I met a certain Kamba lady there**

**Moderator:** Is she a doctor or a nurse?

**Respondent: She is a Doctor**

**Moderator:** What can you say in regards to the way she is attending to you?

**Respondent: She is just ok**

**Moderator:** Have you sought treatment elsewhere?

**Respondent: No but there is a day they came to my place of work, measured our blood pressure and they left**

**Moderator:** What did they tell you about your blood pressure?

**Respondent: It was low at that moment coz it was at 65 then it went back. It fluctuates**

**Moderator:** What kind of services do you receive when you go to a health center?

**Respondent: Like what?**

**Moderator:** For example when you go to {a place}, what kind of services do you get there?

**Respondent: I just go there, take drugs and come back**

**Moderator:** Do they check your blood pressure measurements?

**Respondent: Yea, they check our measurements then they prescribe for us drugs, we collect them then we leave the hospital**

**Moderator:** Do you buy the drugs that you collect there?

**Respondent: There is a time when we used to buy like for now I have gone there twice and I was told that there are no drugs so I should wait. I bought the last time I collected them**

**Moderator:** Are you advised when you go for clinic?

**Respondent: Yes, we were told to stop using cigarettes, alcohol, reduce salt intake and avoid taking Sukuma wiki and meat**

**Moderator:** Do you have any problem with managing your blood pressure? Like for you as an individual, you told me that you were asked to buy your drugs the last time you went for clinic. Do you have any problem with managing your blood pressure?

**Respondent: Money is the problem**

**Moderator:** Do you have insurance?

**Respondent: No**

**Moderator: Looking at your age, is it a hindrance in managing your blood pressure?**

**Respondent: No**

**Moderator:** What about the way you take drugs?

**Respondent: I like taking drugs and that’s why I asked you if I can come to collect them**

**Moderator:** And you don’t have a problem with that?

**Respondent: Yes, they don’t affect me**

**Moderator:** Do you use either alcohol or cigarettes?

**Respondent: No, I used to take alcohol but I have never smoked cigarettes**

**Moderator:** What about now, are you still taking alcohol?

**Respondent: No**

**Moderator:** How is your normal day? Do you leave the house for work?

**Respondent: Yes, I go once and we work for like 4 or 3 days in a week. We just work if we are picked**

**Moderator:** Looking at your family and the community, are they a hindrance in managing your blood pressure?

**Respondent: No, they are not a hindrance**

**Moderator:** And you told me that you don’t take these foods that are not good for you like nyama choma

**Respondent: I was told not to take that**

**Moderator:** So you don’t eat?

**Respondent: No I don’t**

**Moderator:** Looking at your health care provider, is there a hindrance in managing your blood pressure

**Respondent: There is no problem**

**Moderator:** Do you get quality care?

**Respondent: Yes, it’s of good quality**

**Moderator:** What about the time that you go for clinic?

**Respondent: It’s not bad**

**Moderator:** Looking at the hospital, you said that there are times that you get drugs and other times you don’t get

**Respondent: Yes**

**Moderator:** Is the space at the hospital enough for you?

**Respondent: Yes, it’s enough**

**Moderator:** Looking at the government policies, are they hindering you from managing your blood pressure?

**Respondent: No**

**Moderator:** What would be the possible solution to the hindrances that you have mentioned? You told me that sometimes you don’t have money to buy drugs, what would be the solution to that?

**Respondent: The problem is when I don’t get job but it would be better if there was a project that can be giving us money**

**Moderator:** Looking at you as an individual, what can you do differently in regards to blood pressure?

**Respondent: I don’t have any other thing**

**Moderator:** What about your health care provider?

**Respondent: She is ok**

**Moderator:** What about the health center, what is it that can be done differently?

**Respondent: I have not seen anything that is no good there**

**Moderator:** How has COVID19 affected the way you receive hypertension care services in this community

**Respondent: It has affected me because people don’t want to go to the hospital because they fear that they can be tested and found to be positive and there is no work**

**Moderator:** What else can you talk about in regards to blood pressure?

**Respondent: I just request that you help those people that have this condition even if you can be sending is 5000 shillings so that we can buy drugs when we don’t find them at the hospital**

**Moderator: Thank you for the time that you have given me and I believe that the information that you have given me will help us in our research. Thank you**

**Respondent: Ok**

**…END…**
