# Supplementary material for: A socio-ecological framework examination of drivers of blood pressure control among patients with comorbidities and on treatment in two Nairobi slums; a qualitative study
Source: PLOS Glob Public Health. 2023 Mar 10;3(3):e0001625. doi: 10.1371/journal.pgph.0001625 (PMC10021823; doi:10.1371/journal.pgph.0001625)
Supplement: S1 File — (ZIP) [file pgph.0001625.s001.zip › Community/VIWA-IDI-UHTN-200721_0459.docx]

**Moderator: {Name**

**Code:** **VIWA-IDI-UHTN-200721_0459**

**Moderator:** This community has been identified to have a high burden of uncontrolled hypertension which is a leading factor to premature deaths and disability. I am trying to gather information about hypertension care in your community. To avoid hypertension related complications, it is recommended that people with high blood pressure can change their lifestyles in regards to diet, physical activities, smoking, alcohol consumption and using blood pressure medication. So tell me about your experience with having high blood pressure. So tell me in short, tell about your experience with having high blood pressure

**Respondent: I used to smoke cigarettes and sometimes back and again I still used alcohol and cigarettes even as was using these drugs and I realized that my blood pressure was rising but when I stopped using alcohol and cigarettes that’s when my blood pressure became better. Most of the time my blood pressure is not high when it is measured. The only problem that I have is lack of heat but it’s not that bad but I can say that the drugs are ok with me**

**Moderator:** For how long have you been having this condition?

**Respondent: I didn’t have this condition when I was involved in an accident in 2013 but in 2015 is when my blood pressure became high but I was not using drugs and it got better when I started using the drugs. I take drugs on a daily basis**

**Moderator:** Where do you check your blood pressure measurements?

**Respondent: I normally check when I go for my leg clinic**

**Moderator:** Ok

**Respondent: But leave that, I always like checking my blood pressure coz I normally pay for blood pressure check at some place in town to know how my body is**

**Moderator:** What was the reading the last time you checked?

**Respondent: I checked my pressure two weeks ago and I am planning to go for checkup this coming week**

**Moderator:** What was the reading the last time you checked?

**Respondent: It was 168/100**

**Moderator:** 168/100

**Respondent: Yes**

**Moderator:** Ok

**Respondent: That what written in my book**

**Moderator:** There is no problem but it’s good for you to ask for explanation

**Respondent: Yeah, it was that way. Sorry, it was 160/101and the doctor told me that it is ok and it can get better if I continue taking drugs because it was very high before**

**Moderator:** Ok, were you told what your normal blood pressure target should be?

**Respondent: I didn’t ask him how it should be but it would be better if you told me**

**Moderator:** It would be better if you asked him coz personally I have never checked you and I don’t know how your blood pressure condition is

**Respondent: He told me that I am not worse off and I can be better if I continue taking drugs**

**Moderator:** Do you have any other condition apart from blood pressure and the accident that you were involved in in 2013

**Respondent: No**

**Moderator:** Tell me about the antihypertensive drugs that you are taking

**Respondent: I take one tablet daily very early in the morning**

**Moderator:** Has the number of your tablets reduced or increased from the day that you were diagnosed with high blood pressure?

**Respondent: I used to have a problem with drugs before because I was using alcohol while on medication but when I stopped using them, then my condition became better**

**Moderator:** So you have been taking one tablet from 2015?

**Respondent: Yes, a tablet daily**

**Moderator:** Ok

**Respondent: I was using them even that time when I was with you**

**Moderator:** Tell me how hypertension condition has impacted your life

**Respondent: Blood pressure can impact your life in many ways because it becomes worse when your blood pressure rises when you have another disease for example if you have flu it takes long to heal compared to the person who is not hypertensive and the issue with lack of body heat, I don’t know if I am the only one with this problem among many other problems**

**Moderator:** You told me that you take one tablet daily

**Respondent: Yes**

**Moderator:** Is there anything else that you do to manage your blood pressure apart from taking drugs?

**Respondent: I don’t like it when someone annoys me and I try so hard not to be angry. I also try not to sleep when my blood pressure is high; I always try to find something to keep myself busy so that I can distract myself from thinking**

**Moderator:** What about food?

**Respondent: My feeding is normal just that I avoid paper. Don’t like hot food**

**Moderator:** What about exercise?

**Respondent: I walk on foot when am on this side. I exercise through walking and carrying water upstairs**

**Moderator:** Ok, who do you see when you go for blood pressure checkup?

**Respondent: Even these young people have high blood pressure condition and I also find old people there. This condition can affect anybody**

**Moderator:** You told me that you travel from where you stay to this side for clinic

**Respondent: Yes**

**Moderator:** Where do you go for clinic?

**Respondent: I go to {Name of the facility} for clinic**

**Moderator:** Ok, how often do you go for clinic?

**Respondent: After two or three months but I check my blood pressure after two or three weeks**

**Moderator:** Alright, what can you tell me in regards to the way your health care provider is managing your blood pressure condition?

**Respondent: The negative side or which side?**

**Moderator:** In general, what can you say in regard to the way he is managing your blood pressure condition

**Respondent: I just pray for God to bless them because they are very nice people, they seek to know what is the issue in case they find out that your pressure is high and he tell you good things and what you can do and in the process the blood pressure goes down. Your condition becomes better if you follow their instructions**

**Moderator:** Have you ever sought care elsewhere apart from {Name of the facility}?

**Respondent: Yes**

**Moderator:** Where did you go?

**Respondent: I went to {Name of a place]**

**Moderator:** What were you told in regards to your blood pressure when you went to Makongeni?

**Respondent: They told me that my blood pressure is not very high and they asked me if there is another place that I go for checkup and I told them yes and I showed them the results. They encouraged me to continue that way**

**Moderator:** How are the services at the facilities that you go to? Kenyatta and Muranga

**Respondent: Their services are not bad**

**Moderator:** What about free services and blood pressure checkups and the advices they give you. You said that they talk to you when your blood pressure is high and they also advice you on how you are supposed to live. So how do you get your Antihypertensive drugs?

**Respondent: The drugs that I am using are not bad and they would have changed for me if they were bad**

**Moderator:** Do you buy your drugs or you just get them at the hospital?

**Respondent: They give me for free**

**Moderator:** Ok, have you ever gone there and missed drugs?

**Respondent: No, I have never missed drugs**

**Moderator:** What another problem do you get in managing your blood pressure?

**Respondent: Problem with my body?**

**Moderator:** Yes

**Respondent: I always think about my leg problem and that’s when my blood pressure rises. I always think of this metal that is supposed to be removed, my blood pressure and money and that’s when my blood pressure becomes high coz you don’t get money the same way you used hustle when you were ok**

**Moderator:** You told me that you never miss drugs when you go to the hospital?

**Respondent: I have never missed drugs both at {Name of a place} and {Name of the facility}**

**Moderator: Tell me about individual factors, you told me that your blood pressure rises when you think about your leg**

**Respondent: Yes**

**Moderator:** Tell me about the individual factors that can hinder you from managing your blood pressure

**Respondent: I have a lot of problems like getting food and I have a bad leg so hustling for becomes a problem; I also need transport money to and from {Name of the facility} when I go for clinic. For now I am supposed to be in Nairobi but I can’t go there because of this condition. It is worse for a person with high blood pressure to be infected with other conditions**

**Moderator:** You told me that you stopped using alcohol and cigarettes?

**Respondent: Totally**

**Moderator:** Tell me what are the families or communal factors that can hinder you from managing your blood pressure?

**Respondent: There is nobody disturbing me at home. My life is not bad here just that I am not healthy and I lack food**

**Moderator:** You said that you don’t have a problem with your health care providers? You said that they serve and you well

**Respondent: Yes, I have never seen anyone of them disturbing me and they also treat me well**

**Moderator:** What about the time that you take at the hospital? How long do you take?

**Respondent: We take like three minutes for consultation, 3 minutes for blood pressure checkup and the doctor can repeat measuring for like 3 times if he finds that your blood pressure is high so it can take 10 minutes**

**Moderator:** You also told me that you have never missed drugs

**Respondent: Yes, I have never missed drugs at any time that I have gone to the hospital**

**Moderator:** You also told me that you don’t take long before you are served whenever you go for clinic?

**Respondent: No**

**Moderator:** Ok. Asking about the government. What are they doing that hinders you from managing your blood pressure?

**Respondent: The government should try and provide these services free of charge coz like for my case, this leg causes my pressure to rise because you have to pay if you have other conditions even if I get antihypertensive for free. When you don’t have money to pay for treatment, you have other problems at home like problems with food. The government should take care of blood pressure patients. The blood pressure condition can go back to normal if you just take medicine when you don’t have any other problem**

**Moderator:** Ok, you told me some individual problems. What can you do differently compare to what you are doing to manage your blood pressure?

**Respondent: If the government can help me get removed this metal from my leg then my blood pressure wouldn’t be disturbing me and my life will be normal because I can’t hustle in this condition, if I was normal then then my blood pressure will be ok**

**Moderator:** You told me that you moved from Nairobi and decided to go stay at your rural home because of Cororna

**Respondent: Yes, I came here so that I can avoid being infected with another disease yet my leg issue is still disturbing me**

**Moderator: How has this corona period affected the way you get your hypertension care service? You told me that you went to your rural home because you fear COVID**

**Respondent: I go to a dispensary located close to my place**

**Moderator:** What’s the name of the dispensary?

**Respondent: It is called {Name of a place} dispensary**

**Moderator:** Ok, on to the last question. Is there any other thing that we have not talked about in regards to high blood pressure and you would want to mention?

**Respondent: I only want us to talk of how the government can help us people with high blood pressure and at least also help us on how we can get food. Only that**

**Moderator:** Thank you for your time and the information that you have given us. I think your information will reach those that are supposed to get it and it will help us change what we are supposed to change. Thank you

**Respondent: Ok**

**…END…**
