# Supplementary material for: A socio-ecological framework examination of drivers of blood pressure control among patients with comorbidities and on treatment in two Nairobi slums; a qualitative study
Source: PLOS Glob Public Health. 2023 Mar 10;3(3):e0001625. doi: 10.1371/journal.pgph.0001625 (PMC10021823; doi:10.1371/journal.pgph.0001625)
Supplement: S1 File — (ZIP) [file pgph.0001625.s001.zip › Community/VIWA-IDI-UHTN-200722_0058.docx]

**Moderator: {Name}**

**Code:** **VIWA-IDI-UHTN-200722_0058**

**Moderator:** This community has been identified to have a high burden of uncontrolled hypertension which is a leading factor to premature deaths and disability. I am trying to gather information about hypertension care in your community. To avoid hypertension related complications, it is recommended that people with high blood pressure can change their lifestyles in regards to diet, physical activities, smoking, alcohol consumption and using blood pressure medication. So tell me about your experience with having high blood pressure. Kindly tell me about your experience with having high blood pressure

**Respondent: I have been having this condition for like 5 years but I have not been able to do what I am required to do because I work as a shop keeper so most of the time I must be there selling even now I am at the shop so I don’t find time to do exercise. I would say that I only depend on medicine in managing my blood pressure but I find it hard to use those other means that can help me manage my blood pressure for example exercising but on diet you know the situation in Nairobi, we feed on the same food which is ugali and rice**

**Moderator:** How often do you check your blood pressure?

**Respondent: For now I don’t check though I am on medication, I am using drugs and I go for clinic after two months at {Name of the facility} and I always find my measurements to be ok because I use drugs**

**Moderator:** You told me that you normally go for checkup at after two months

**Respondent: Yes**

**Moderator:** What were the measurements reading the last time you checked?

**Respondent: The way it was**

**Moderator:** Yes, can you remember?

**Respondent: Even if I don’t remember I just know that it was at one twenty something over seventy**

**Moderator:** Do you record these measurements somewhere or how does it go?

**Respondent: I don’t record, it just depends on what I get at the clinic for example the doctor advises me on what to do if they find that my blood pressure is not ok**

**Moderator:** Has your doctor ever told you what your blood pressure normal target should be?

**Respondent: Yes**

**Moderator:** What did your doctor tell you?

**Respondent: He told me that it shouldn’t exceed 130 or 140 over 80 or 70**

**Moderator:** How many antihypertensive tablets are you taking?

**Respondent: I take 2 tablets**

**Moderator:** You told me that you have been having this blood pressure condition for 5 years

**Respondent: Yes, about**

**Moderator:** You just told me that currently you are taking 2 tablets. Has the number of your tablets been increasing or reducing in number?

**Respondent: At first I used to take more than two tablets even though I was going to another facility and not {Name of the facility}**

**Moderator:** Where were you going?

**Respondent: I used to go to {Name of the hospital}**

**Moderator:** What made you change from {Name o of the hospital to {Name of the facility}?

**Respondent: I reached a time that I lost trust with the doctors that were treating me at {Name of the hospital} because they were just checking my blood pressure and gave me prescriptions in case they found that it was high but at {Name of the facility} I would say they are more active, they check what could be wrong with the patient, my liver was checked at {Name of the facility} but at {Name of the hospital} I was just going to collect drugs and go back home**

**Moderator:** So, you mean that at {Name of the facility} they have been giving you advice and they do check you on everything apart from just giving you drugs?

**Respondent: Exactly and they also check other conditions that might come up due to high blood pressure but at {Name of the hospital} I was just going for blood pressure measurements, drug collection then I go home**

**Moderator:** How has blood pressure affected your life?

**Respondent: I didn’t get you; you mean how I got this condition or the effects?**

**Moderator:** The effects of blood pressure condition on you for the last five years like how it has affected your life

**Respondent: I can mention several effects like for example my relationship status with my wife has been affected. It’s like I am disabled**

**Moderator:** You told me that you cannot find time to do exercise, so apart from drugs, what else do you do to manage your blood pressure?

**Respondent: I do jog sometimes and I have also reduced eating some types of foods like cakes**

**Moderator:** Ok. You told me that you go to {Name of the facility} after every two months, who do you see when you go there?

**Respondent: I don’t see a specific person. I can go this time for clinic and this doctor and when I go there next time I’ll find another doctor**

**Moderator:** What can you say about the way they treat you? Is there any difference between the different doctors that attend to you?

**Respondent: I can say that it is not bad because there is record of how it was last time so the doctor goes by what is recorded in the file in regards to how the patient is fairing**

**Moderator:** Ok. You told me that you were going to {Name of the hospital} before you started going to {Name of the facility}

**Respondent: Yes**

**Moderator:** Have you ever sought for treatment elsewhere apart from {Name of the facility} and {Name of the hospital}?

**Respondent: Maybe before I knew that I had that condition. I had gone to these local hospitals located in the village like for example {Name of the facility} before I decided to look for a hospital of that kind of status**

**Moderator:** What made you change from going to {Name of the facility}?

**Respondent: It was at the initial stage and personally I felt that it was much serious and that’s why I decided to go to a hospital where I can be served better**

**Moderator:** Ok, tell me what kind of services do you get at {Name of the facility}? Like what do they do when you reach there?

**Respondent: First they take the blood pressure measurements then you see a doctor so that he can advise you on what to do in case your blood pressure condition is bad, he also ask questions about how you feel and that help the doctor to know what he can do and they take action if the patient complains**

**Moderator:** What about drugs like how do you get them?

**Respondent: They sell for us the drugs. They just prescribe and the patient can choose either to buy from them or any other place**

**Moderator:** Ok. You also told me that you receive advice from the health care providers that attend to you?

**Respondent: Yes**

**Moderator:** Tell me about the hindrances that you encounter as you try to manage your blood pressure condition

**Respondent: The challenge that I face is maybe the costs of acquiring drugs**

**Moderator:** Do you pay in cash or you have an insurance card

**Respondent: I pay in cash though I have an insurance card but it doesn’t cater drugs or outpatient cases**

**Moderator:** Ok. What about you taking drugs as directed by the doctor?

**Respondent: I try so hard to take the drugs in the morning and evening**

**Moderator:** So you take your drugs in the morning and evening?

**Respondent: Yes**

**Moderator:** Ok, what about your age?

**Respondent: I think I am 51 or 52 years**

**Moderator:** Tell me the individual factor that hinders you from managing your blood pressure. You had told me that sometimes the prices are high

**Respondent: The current situation is a challenge coz if you look at what has befell us as a nation and my blood pressure gets affected when I think about that**

**Moderator:** What are the family or community factors that hinder you from managing your blood pressure?

**Respondent: For now I don’t know of any**

**Moderator:** What do you think your health care providers at {Name of the facility} can do to manage your blood pressure?

**Respondent: Their efforts in trying to manage my condition are the best but maybe the problem is with me because of what I told you like maybe exercises even though I take drugs**

**Moderator:** How long do you take to be attended at the hospital?

**Respondent: It takes like 3 hours**

**Moderator:** How do you find that?

**Respondent: I normally close my job and go or maybe I leave my wife there**

**Moderator:** What do you think that the government is not doing and if they did it then your blood pressure would be normal?

**Respondent: Talking of the government I can say that the government has failed us because there many things that have happened to me in this slum. You can call it development to the people but you find that it is affecting you directly as an individual. When I say that I am referring to me and the other person. Generally I can say that the economy is contributing a lot**

**Moderator:** What can be the solution to the issues that you mentioned like foe example you talked about lifestyle issues and lack of money. What do you think would do as an individual to help you manage your blood pressure?

**Respondent: The solution would be like several things that maybe cannot be actualized because you find that I have many responsibilities. I cannot close my shop to go for exercise yet there are people waiting for me to provide at the end of the day so you just have to stay at the shop for you to get what my children can eat. You can’t stay the way you are supposed to stay because of not having enough**

**Moderator:** You told me that you don’t have any problem with {Name of the facility} Hospital where you go for your clinics?

**Respondent: Yes**

**Moderator: You also said that you are ok with their hours of operation and they treat you well, they give you information about blood pressure, you always get drugs**

**Respondent: That’s true**

**Moderator:** How has COVID 19 affected the way you get your hypertension care services at {Name of the facility}?

**Respondent: For now I can say that it has not affected me because I manage to go when I am supposed to go and they provide me with drugs or they write for me then I go to buy**

**Moderator:** For now the drugs that you have can take you for how long?

**Respondent: For now I have drugs that can last me for like one month**

**Moderator:** Ok, on to the last question, is there anything else that you would want us to talk about in regards to high blood pressure and you feel like we have not mentioned

**Respondent: I can only talk about the side effects that come with this condition because you can just say that its high blood pressure but there are other effects on the body like what I told you but I don’t know what causes that. I also ask myself if taking drugs daily can bring other effects other than being affected by that condition**

**Moderator:** So you think that you have to take a lot of drugs

**Respondent: Yes on a daily basis makes me think if there could be a long term effect. That’s a question that I always ask myself**

**Moderator:** It would be better for you to ask your health care provider about these drugs and for him to tell you why you are taking a certain type of drug and their side effects for you to know why you are taking them instead of just collecting your drugs then you leave

**Respondent: That’s true**

**Moderator:** Just go there because he is the one who knows the type of the drugs that you are taking and he has all the information

**Respondent: Ok**

**Moderator:** It would be better if you asked your health care provider to tell you the reason as to why you are taking two tablets yet the other person is taking one tablet and also ask about the side effects and the reason as to why you are taking these drugs for long. It’s good to ask so that you can know the type of tablets that you are taking and their side effects

**Respondent: True**

**Moderator:** Thank you for your time and the information that you have given me and I think that it will reach the people that it can reach so that we can change on what we have not been doing right and do much better on what we are doing right

**Respondent: Ok**

**Moderator:** Thank you

**Respondent: Ok**

**…END…**
