# Supplementary material for: A socio-ecological framework examination of drivers of blood pressure control among patients with comorbidities and on treatment in two Nairobi slums; a qualitative study
Source: PLOS Glob Public Health. 2023 Mar 10;3(3):e0001625. doi: 10.1371/journal.pgph.0001625 (PMC10021823; doi:10.1371/journal.pgph.0001625)
Supplement: S1 File — (ZIP) [file pgph.0001625.s001.zip › Community/VIWA-IDI-UHTN-200722_0845.docx]

**Moderator: {Name}**

**Code: VIWA-IDI-UHTN-200722_0845**

**Moderator:** This community has been identified to have a high burden of uncontrolled hypertension which is a leading factor to premature deaths and disability. I am trying to gather information about hypertension care in your community. To avoid hypertension related complications, it is recommended that people with high blood pressure can change their lifestyles in regards to diet, physical activities, smoking, alcohol consumption and using blood pressure medication. So tell me about your experience with having high blood pressure. Kindly tell me about your experience with having high blood pressure

**Respondent: My experience is just having problems with my body, sometimes my blood pressure goes up other times down but I am controlling by using drugs but the problem is that I usually feel headaches when I take the drugs and I also do urinate a lot. That’s the experience that I have had so far with blood pressure**

**Moderator:** Ok, for how long have you been having high blood pressure?

**Respondent: From 2008**

**Moderator: How often do you check your blood pressure?**

**Respondent: After every two weeks**

**Moderator:** Where do you go to check your blood pressure?

**Respondent: There is a facility called {Name of the facility} where I stay**

**Moderator:** Access

**Respondent: Yes, I pay 20 shillings to be checked**

**Moderator:** Do you record these pressure measurements every time you are checked?

**Respondent: They record in their book for follow up**

**Moderator:** What were the measurements the last time you checked?

**Respondent: It was 140/101 or 102**

**Moderator:** Do you have any other condition apart from high blood pressure?

**Respondent: No I don’t have any other condition**

**Moderator:** Have been told your target blood pressure by your doctor at access?

**Respondent: Yes, I was told**

**Moderator:** What did they tell you?

**Respondent: 120/90**

**Moderator:** How many antihypertensive tablets have you been taking from 2008?

**Respondent: Two**

**Moderator:** Have these tablets been increasing or reducing in number since the time you started taking them in 2008

**Respondent: They have been changing drugs whenever I tell them how I am feeling**

**Moderator:** Did the doctor tell you the reason as to why he has been changing your drugs?

**Respondent: The doctor said that they do change because am getting older and so they have to change because the other drug was not managing the condition well**

**Moderator:** How many times have you been taking the two tablets?

**Respondent: Once**

**Moderator:** In the morning or in the evening?

**Respondent: I take it in the evening**

**Moderator:** How has high blood pressure affected your life?

**Respondent: Maybe on expenses but again there are foods that I love taking but I was told not to take them. I really love eating meat but I was told not to eat but I eat once a week but it doesn’t hinder me from doing work. I still do my job**

**Moderator:** What else do you do to manage your blood pressure apart from taking drugs?

**Respondent: There is nothing that I do**

**Moderator:** You had told me that you changed your diet

**Respondent: They really advise me on my diet**

**Moderator:** You also mentioned that drugs are expensive

**Respondent: Yes**

**Moderator:** What about exercise?

**Respondent:** I don’t exercise a lot unless when I go to my home and I do some manual work

**Moderator:** What kind of job are you doing?

**Respondent: For now …7:00-7:04… (Not clear) that is what I operate**

**Moderator:** Who do you see when you go to access?

**Respondent: I do see the person who does the measuring and sometimes he refers me to s doctor in case he looks at my records and finds out that I am not doing well**

**Moderator:** What can you tell me in regards to the way your health care provider manages your blood pressure?

**Respondent: He responds well coz from the way we have been talking; I have no problems with the drugs that he prescribes**

**Moderator:** Have you ever gone elsewhere to seek care apart from {Name of the facility?

**Respondent: Yes, I went to {Name of the facility}**

**Moderator:** What were you told at {Name of a facility} in regards to blood pressure?

**Respondent: They took my blood pressure measurements and they referred me to a nutritionist who advised me on diet. I also go there to collect my drugs because there my employer has registered for our insurance with them and so I go there to collect my drugs**

**Moderator:** What services do you receive when you go to {Name of the facility} or {Name of the facility} apart from giving you medicine?

**Respondent: There is no other service not unless I go there with other conditions and they treat me but there are no other services**

**Moderator:** You told me that access is located close to the place where you stay

**Respondent: Yes, it’s close to where I stay**

**Moderator:** You told me that you pay for your drugs using insurance card

**Respondent: Yes**

**Moderator:** So it’s not a big expense or getting medicine is not a big problem

**Respondent: Yes, maybe when I have finished and I have not been given permission to go to {Name of the facility} so I am forced to come and buy them at {Name of the facility}**

**Moderator:** So you do collect your medicine at {Name of the facility} using insurance card when you have time and when you don’t have time you buy them at access

**Respondent: Yes**

**Moderator:** Do you have any other individual problems in regards to managing your blood pressure apart from the ones that you have mentioned

**Respondent: No, I don’t have any other problem apart from flu that is caused by the cold season**

**Moderator:** What are the individual factors that can hinder you from managing your blood pressure?

**Respondent: Lack of exercise and also the work that I do requires me to sit down and I think that contributes a lot. The doctor told me that I should be doing exercise. He also recommended that I be going to the GYM but for now it’s not possible**

**Moderator:** What about you taking medicine in time as directed by the doctor?

**Respondent: It is ok**

**Moderator:** So you don’t miss taking your drugs and you take them at the time that you should take them as instructed by the doctor?

**Respondent: Yes, I always take them at the required time**

**Moderator: What about other problems like taking alcohol and smoking cigarettes?**

**Respondent: No, I don’t have such problems**

**Moderator:** What are the family or community factors that might hinder you from managing your blood pressure?

**Respondent: I get little money and the expenses at home on my family hinder me from going to the gym**

**Moderator**: Are you able to get foods that the doctor advised you to take at the place where you stay?

**Respondent: Yes I have access but I might need to take some foods but you end up not eating those foods due to lack of money**

**Moderator:** Ok. And what are the health providers’ factors at the facilities that you visit either at {Name of the facility} or {Name of the facility} Or what are they not doing and you feel that if they did that then your blood pressure will be normal?

**Respondent: Personally I think they are ok**

**Moderator:** You don’t have any problem with them?

**Respondent: I don’t have any problem with them**

**Moderator:** How long does it take you from the time when you go to the clinic and the time that you leave clinic?

**Respondent: It takes me one hour at {Name of a facility} because they have many clients there but at {Name of a facility} it takes me 30 minutes because I go there in the evening when there are no clients**

**Moderator: You said that you receive nutrition teachings at {Name of a facility}**

**Respondent: Yes**

**Moderator:** What do you think are the government factors that if they did differently then your blood pressure would be normal?

**Respondent: For the government I would say that if their services were better like for example at {Name of the facility} then I wouldn’t go to where I am required to use money and I also wouldn’t buying because I would be going there to collect my drugs**

**Moderator:** What are the possible solutions to the individual, community, family and government factors that you told me? Starting with you as an individual, what would you do to manage your blood pressure?

**Respondent: For government?**

**Moderator:** No you as an individual, you told me that sometimes you don’t do exercise and other times you don’t have money to buy the food that your doctor advised you to take. What do you think you can do for you to manage your blood pressure?

**Respondent: Maybe I get an extra income so that my money can be enough for me and my needs but because I have financial problems then that’s why I am limited on getting some things and going to the gym. Extra income would be good because maybe I would be able to control my blood pressure**

**Moderator:** What do you think should be changed at the health center?

**Respondent: For the hospital they should bring medicine and make their services better. We would be going there but that’s why you see us going to private clinics**

**Moderator:** We are about to finish, how has COVID19 affected the way you receive hypertension care at the community?

**Respondent: The problem with COVID is that it’s really controlled so you see like the hospital where I am supposed to go**

**Moderator:** Pardon

**Respondent: COVID can make me not to go for medication like am supposed to but not that much**

**Moderator:** Ok. Like for now you have drugs that can last you for how long?

**Respondent: For now I have drugs that can last for one week before I go to look for other drugs**

**Moderator:** Is there any other thing that COVID has affected apart from you going for your drugs?

**Respondent: I don’t think**

**Moderator: Ok. On to the last question**

**Respondent: Ok**

**Moderator: What do you think that we have not mentioned and you feel like talking about in regards to high blood pressure?**

**Respondent: I think that you have covered all areas and I don’t think if there is any other thing that was left**

**Moderator:** Ok, thank you so much for your time and the information that you have given me and I think that it will reach those that are supposed to be reached so that we can better those areas that are not ok.

**Respondent: Ok**

**Moderator: Thank you so much**

**Respondent:**

**…END…**
