# Supplementary material for: A socio-ecological framework examination of drivers of blood pressure control among patients with comorbidities and on treatment in two Nairobi slums; a qualitative study
Source: PLOS Glob Public Health. 2023 Mar 10;3(3):e0001625. doi: 10.1371/journal.pgph.0001625 (PMC10021823; doi:10.1371/journal.pgph.0001625)
Supplement: S1 File — (ZIP) [file pgph.0001625.s001.zip › Community/VIWA-IDI-UHTNC-200712_004.docx]

**Moderator: {Name}**

**Code:** **VIWA-IDI-UHTNC-200712_004**

**Moderator:** This community has been identified to have a high burden of uncontrolled hypertension which is a leading factor to premature deaths and disability. I am trying to gather information about hypertension care in your community. To avoid hypertension related complications, it is recommended that people with high blood pressure can change their lifestyles in regards to diet, physical activities, smoking, alcohol consumption and using blood pressure medication. So tell me about your experience with having high blood pressure

**Respondent: My experience is that I didn’t have means to sustain myself and that’s why I developed high blood pressure**

**Moderator:** For how long have you been having high blood pressure?

**Respondent: I realized that I have pressure like 10 years ago actual I realized that I have high blood pressure after having it for 10 years**

**Moderator:** Where do you go to check your blood pressure?

**Respondent: I go to nearby facilities when I feel like my body is not ok but I go to the hospital in case I feel like I have not received enough treatment**

**Moderator:** How often do you check your blood pressure measurements?

**Respondent: I don’t taste every time but when I feel like my body is not ok like when I feel dizzy I know that my blood pressure is high then I go to check**

**Moderator:** Do you keep records of your blood pressure measurement?

**Respondent: Like I checked on Friday and I was told that my blood pressure is low though I had gone there because of a heart burn. I was told that my blood pressure was ok but the doctor found out that I had another problem**

**Moderator:** What were the measurements when you went for checkup on Friday?

**Respondent: It was 118/85**

**Moderator:** Has your doctor ever told you what your normal blood pressure target should be?

**Respondent: He has never told me what the normal reading should be but I always ask them questions about blood pressure and I was told that if the blood pressure is ok if the readings are 130 something**

**Moderator:** Which drugs do you take?

**Respondent: I don’t remember the name but I can text you the name later if I remember**

**Moderator:** How many tablets do you take daily?

**Respondent: I take one tablet daily**

**Moderator:** Have you been using the same drug for the 10 years that you have been having this condition?

**Respondent: I started with the same drug but I was taking a half of it but I started taking a full tablet when the doctor realized that my blood pressure was not going back to normal**

**Moderator:** Do you have any other condition apart from high blood pressure?

**Respondent: I am asthmatic and I also have a problem with my stomach and I have had operation two times but the problem has not stopped**

**Moderator:** How has hypertension condition affected you?

**Respondent: It affects me because sometimes I feel weak when my blood pressure is high and other times I just fall when walking or I support myself somewhere and when I feel ok then I continue walking**

**Moderator:** Apart from using medicine, how else do you manage your blood pressure? You told me that you are using one tablet

**Respondent: Yes**

**Moderator:** So apart from using that tablet, how else do you manage your blood pressure?

**Respondent: There is nothing else that I do**

**Moderator:** What about diet?

**Respondent: With food I don’t eat much because I have a stomach problem like I told you and sometimes I can take tea or even water for like 2 days when I feel like it has gas**

**Moderator:** What about exercise?

**Respondent: I find it hard for me to do exercise coz I can’t even jump because of the two operations that I have had before. I would really love to skip a rope but it’s hard for me**

**Moderator:** Have you ever used traditional medicine?

**Respondent: I have never**

**Moderator:** What else do you do to manage your blood pressure?

**Respondent: I just do my house chores then I feel much better but I don’t do any exercise or use any medicine apart from the tablets that I am using**

**Moderator:** Who do you see when you go to the hospital?

**Respondent: I just do buy drugs in the local clinics from the time the doctor prescribed for me at the hospital**

**Moderator:** When you go to there, do you see a doctor or a nurse

**Respondent: I see a nurse though I have never gone to the hospital from that time when I was diagnosed and told the drugs that I was supposed to be using. I just buy them from a local clinic**

**Moderator:** What can you say about your health care provider in regards to the way he is managing your high blood pressure condition

**Respondent: He serves me well because he still attends to me even if I don’t have money**

**Moderator:** Have you ever sought care elsewhere?

**Respondent: No**

**Moderator:** What kind of services do you receive when you go for clinic?

**Respondent: I depend with the condition that takes you there because if you go to the hospital with ulcers or blood pressure then the health care provider attends to that**

**Moderator:** What kind of services do you get when you go seeking hypertension care?

**Respondent: Sometimes they measure to check if it’s high or low and other times they just give medicine**

**Moderator:** Are you given drugs for free when you go for clinic?

**Respondent: We buy**

**Moderator:** What about advise? Does the doctor advise you?

**Respondent: No**

**Moderator:** Do they check your blood pressure measurements?

**Respondent: Yes and he files**

**Moderator:** How often do you check your blood pressure?

**Respondent: I check after every week**

**Moderator:** Do you have any problem with managing your blood pressure? Looking at you as an individual, you told me the issue of money. Do you have NHIF?

**Respondent: Yes**

**Moderator:** Does it help you in acquiring your blood pressure medicines?

**Respondent: I have never used it on hypertension because I had not activated outpatient. I just activated the other day**

**Moderator:** Ok

**Respondent: I activated in February**

**Moderator:** So you have always been paying from your pocket?

**Respondent: Yes**

**Moderator:** Looking at your age, is it a problem in managing your blood pressure?

**Respondent: I didn’t get you**

**Moderator:** Do you have a problem with managing your blood pressure? We are looking at the age

**Respondent: Yes, I have a problem coz there are times when I don’t have drugs and I can’t find the money to buy them**

**Moderator:** You also told me that you have ulcers and you are also asthmatic

**Respondent: Yes**

**Moderator:** Does that hinder you from managing your blood pressure?

**Respondent: I don’t have a problem if I found someone who attends to me**

**Moderator:** What about the way you take your drugs?

**Respondent: I don’t have a problem, but I will really appreciate if I found someone to assist me on drugs**

**Moderator:** What about your family?

**Respondent: What do I tell you about family?**

**Moderator:** I mean your community in managing your blood pressure. We are looking at what can hinder you from managing your blood pressure and now we are looking at the community

**Respondent: I don’t know if there is a problem or not because I didn’t understand anything there but I have a son who is asthmatic**

**Moderator:** What about diet? Because you said that you are asthmatic and hypertensive. Looking at your diet at home

**Respondent: There is no problem**

**Moderator:** What about your health care providers? Do you have any problem in managing your blood pressure? Looking at your health care providers

**Respondent: There is no problem**

**Moderator:** Do you get quality treatment? Or how do you see it?

**Respondent: I can’t tell you if it is bad or good because I told you that I do buy my tablets and when I don’t have money then I take them on credit but I can say that there is no big problem**

**Moderator:** So you buy the tablets when you don’t find them at the hospital?

**Respondent: Yes, I can go to the hospital that I have chosen for outpatient service but if I don’t find drugs there then I just buy**

**Moderator:** If you look at policies or we look at the government.

**Respondent: It’s ok, there is no problem**

**Moderator:** Have you ever been told about hypertension guidelines?

**Respondent: Visiting me?**

**Moderator:** Yes

**Respondent: I have never been told about that**

**Moderator:** What would be the solution to the hindrances that you mentioned? You told me that you buy drugs but for now you have NHIF that you have not yet used. What would be the solution to that?

**Respondent: The solution would be on medication because sometimes I find it hard to get medication and also getting food is a problem but I will really appreciate if you help me on that**

**Moderator:** Looking at drugs, you said that sometimes you buy them at the chemist especially when you don’t find them at the hospital. What would be the solution to that?

**Respondent: Ill also appreciate if I can get drugs**

**Moderator:** What else can you do differently in regards to blood pressure?

**Respondent: I can do business because I am employed and the work that I do is not sustainable because I just do casual house job and that why I told you that sometimes it’s hard for me**

**Moderator:** Do you leave your house or you just stay at home?

**Respondent: I do leave in search of casual house jobs**

**Moderator:** What do you think that your health care provider can do differently?

**Respondent: I don’t get you sister**

**Moderator:** When I say your health care provider I mean your doctor, what else do you think that he can do differently in regards to blood pressure?

**Respondent: I don’t understand that, let’s just skip that**

**Moderator:** Looking at your health care facility, what else can be done differently?

**Respondent: Health facility?**

**Moderator:** Yes, clinic

**Respondent: They should help us get drugs**

**Moderator:** How has COVID19 affected hypertensive service delivery in your community?

**Respondent: It has affected because I fear going to the hospital and that’s why we are buying drugs from chemists as we wait to see how this situation ends up. COVID has affected that side**

**Moderator:** Is there any other thing that you can talk about in regards to blood pressure?

**Respondent: What I can say is that blood pressure is a condition that has affected many people and it’s a condition that does not have treatment but treatment can only be effected by us by only accepting ourselves but if you don’t accept yourself then this condition will affect you because it’s a condition that you can know when it comes and when it goes and you also don’t know when the blood pressure rises or when it comes down. It’s your own responsibility to bring it down by controlling my life so that I can know how I can help myself in my life so that I can leave a good life**

**Moderator:** Thank you so much for the information that you have given me and I am happy with the information that you have given me and I know that it will be of help to the research that we are doing. Thank you so much and have a good day

**Respondent: Ok**

**…END…**
