# Supplementary material for: A socio-ecological framework examination of drivers of blood pressure control among patients with comorbidities and on treatment in two Nairobi slums; a qualitative study
Source: PLOS Glob Public Health. 2023 Mar 10;3(3):e0001625. doi: 10.1371/journal.pgph.0001625 (PMC10021823; doi:10.1371/journal.pgph.0001625)
Supplement: S1 File — (ZIP) [file pgph.0001625.s001.zip › Community/VIWA-IDI-UHTNC-200713_2056.docx]

**Moderator: {Name}**

**Code:** **VIWA-IDI-UHTNC-200713_2056**

**Moderator:** This community has been identified to have a high burden of uncontrolled hypertension which is a leading factor to premature deaths and disability. I am trying to gather information about hypertension care in your community. To avoid hypertension related complications, it is recommended that people with high blood pressure can change their lifestyles in regards to diet, physical activities, smoking, alcohol consumption and using blood pressure medication. So tell me about your experience with having high blood pressure. Kindly tell me about your experience with having high blood pressure

**Respondent: The drugs that am using are not of help because I always see that my blood pressure is high but I was told that the foods that I am taking are the reason as to why it’s not going down and maybe thinking too much. That’s the reason why my blood pressure is sometimes low and other times high. There also times when my sugar levels go down that I am forced to go to the hospital. My drugs were changed and I feel like the ones that I am using are making me feel better**

**Moderator:** Ok, when were your drugs changed?

**Respondent: They were changed in June**

**Moderator:** For how long have you been having high blood pressure?

**Respondent: From 2003 that’s when I knew. I used to feel a headache and I went to the hospital and that’s when I was told that I was hypertensive**

**Moderator:** How did you become diabetic?

**Respondent: I knew that I was diabetic later**

**Moderator:** So you contracted high blood pressure first then diabetes?

**Respondent: Yes contracted high blood pressure and I went to the hospital and the doctor confirmed that I was hypertensive and after sometime I went to the hospital where I was again told that I was both hypertensive and diabetic**

**Moderator:** Ok

**Respondent: So I go for the two clinics that is diabetic and blood pressure clinic and I use both antihypertensive and diabetes medicine**

**Moderator:** Ok. How often do you check your blood pressure?

**Respondent: I go for check up on a weekly basis**

**Moderator:** Where do you go to check your blood pressure?

**Respondent: I go to {Name of the facility} because it is the nearest facility**

**Moderator:** {Name of the facility}is located close to you place

**Respondent: Yes, at {Name of a place}or {Name of a facility}**

**Moderator:** Only those two

**Respondent: Yes, only those two**

**Moderator:** What was your last blood pressure measurement reading?

**Respondent: It was 182**

**Moderator:** Over?

**Respondent: I don’t know, I did not look**

**Moderator:** Ok, you also told me that you are diabetic

**Respondent: Yes, sugar levels were 19.3**

**Moderator:** Was that the last test that you had

**Respondent: I went again and I was told that it was 17**

**Moderator:** What did they tell you?

**Respondent: I was told to buy a machine so that I can be testing myself after they train me on how to do it. I was told that I can get the machine at 4500 shillings**

**Moderator:** Were you told that at {Name of the facility} or {Name of the facility}?

**Respondent: At {Name of the facility}**

**Moderator:** Ok, when you go to either {Name of the facility} or {Name of the hospital}, has your doctor told you what your target blood pressure should read?

**Respondent: Measurements**

**Moderator:** Yes

**Respondent: 130**

**Moderator:** He told you 130?

**Respondent: Yes, that’s how it should be. Around 130 to 140 there**

**Moderator:** Ok, how many antihypertensive tablets are you taking now?

**Respondent: I take 2 tablets that are 500mg in the morning**

**Moderator:** How many types are you taking?

**Respondent: I take 2 types of tablets for diabetes and two types for pressure**

**Moderator: So you take 2 types of tablets?**

**Respondent: I take 4, sorry 5. I take 2 measuring 500 mg, 1 luglo tablet and for blood pressure I take one pink tablet and a white one**

**Moderator:** So you take 2 tablets for hypertensive and 3 for diabetes

**Respondent: Yes**

**Moderator:** Have your blood pressure drugs been reducing or increasing from the day you started taking them?

**Respondent: Sometimes I go and find that they have reduced**

**Moderator:** Has the doctor ever told you the reason as to why they are reducing?

**Respondent: He told me that it’s because of the food that I am eating. Sometimes I take too much of a certain food when I am supposed to balance my diet**

**Moderator:** Ok, you told me that you take 2 antihypertensive drugs

**Respondent: Yes**

**Moderator:** From the time when you started taking them in 2013

**Respondent: I didn’t start taking them in 2013**

**Moderator:** Sorry, 2003

**Respondent: I started taking them last year when I realized that one of my legs was dying, it couldn’t step down. That’s when I was told that it’s because I was not taking drugs. I took long before I started taking those drugs**

**Moderator:** Why?

**Respondent: They had some side effects on me and so I stopped but nowadays I am taking them**

**Moderator:** When did you start taking them?

**Respondent: I started taking them last year**

**Moderator:** 2019?

**Respondent: Yes, 2019**

**Moderator:** How many tablets were you taking when you started medication in 2019?

**Respondent: I developed breast cancer**

**Moderator:** Sorry

**Respondent: And I used to take many types of drugs when I was admitted at the hospital and this made my blood pressure to be very high and I was told that I couldn’t undergo operation because my blood pressure was so high. The doctor said that my blood pressure has to be controlled before I undergo operation**

**Moderator:** Which year was you admitted?

**Respondent: I was admitted last year December**

**Moderator:** When did you know that you have breast cancer?

**Respondent: I knew about it in October last year. I went to {Name of the hospital} and I told them how I was feeling and asked to know what could be the problem. They referred me to German center for x-ray because they didn’t have a surgeon and you know they don’t tell you your problem. They just give you the image and tell you to take them back to your doctor**

**Moderator:** They gave you the images for you to take them to your doctor?

**Respondent: Yes**

**Moderator:** How has this hypertension condition affected your life?

**Respondent: I don’t feel very well, I always feel weak**

**Moderator:** Apart from taking drugs, what else do you do to manage your blood pressure?

**Respondent: I just use medicine so that I can feel better**

**Moderator:** What about dieting and exercising?

**Respondent: I just eat what I am supposed to eat but again I don’t get full when I eat what I have been told. There are times that I take fruits but I find them very sugary and so I am forced to find something that I can eat**

**Moderator:** Has the doctor advised you on what you can eat?

**Respondent: Yes, I was advised**

**Moderator:** Who do you see when you go either to {Name of the facility} or {Name of the hospital}?

**Respondent: I see a doctor**

**Moderator:** What can you tell me in regards to the way your doctor is managing your blood pressure?

**Respondent: They check my blood pressure measurements then they give me advice on food and then they give me drugs**

**Moderator:** How is this doctor managing your blood pressure condition?

**Respondent: He tell me about the foods that I am supposed to take**

**Moderator:** What else did he tell you apart from food?

**Respondent: He also told me that I am supposed to be doing exercise**

**Moderator:** Have you been doing the exercises?

**Respondent: I have not been exercising because I find it hard to walk**

**Moderator:** Why do you find walking hard?

**Respondent: I just feel weak and tired when I walk for long**

**Moderator:** You told me that you go to {Name of the facility} and other times you go to {Name of the hospital}?

**Respondent: Yes**

**Moderator:** What made you change from going to {Name of the facility} to {Name of the hospital}?

**Respondent: I used to go to {Name of the hospital} before {Name of the facility} came and its close**

**Moderator:** You said that {Name of the facility} is located closer to the place that you stay?

**Respondent: Yes**

**Moderator:** What kind of services do you get there?

**Respondent: I just go there to check my blood pressure and collect drugs**

**Moderator:** What services do you get there apart from checking your blood pressure and drugs collection?

**Respondent: Nothing else**

**Moderator:** You said that you go there once a week

**Respondent: Yes**

**Moderator:** What challenges do you encounter in managing your blood Pressure?

**Respondent: When I go there they do check my blood pressure and diabetes but the other day they asked me if I can but a machine that can help me test myself**

**Moderator:** And you also told me that you can’t take your drugs daily

**Respondent: That was way back**

**Moderator:** Ok, but for now you are taking the as you are supposed to

**Respondent: Yes, I am taking them now**

**Moderator:** As you are supposed to

**Respondent: Yes**

**Moderator:** What about the cost of drugs and cost of the services that you get at {Name of the facility}?

**Respondent: The drugs are expensive but not as compared to other facilities and there are others that are very strong**

**Moderator:** Do you use cash or you use insurance?

**Respondent: I do pay cash**

**Moderator:** What are the community or family factors that can hinder you from managing your blood pressure?

**Respondent: I have kids that I look after, I provide for them yet I don’t have a good income**

**Moderator:** What about the place that you stay and the foods that are available there?

**Respondent: Food is there. I buy and cook**

**Moderator:** So you buy and cook, you don’t eat those foods that are

**Respondent: I cook myself**

**Moderator:** What is your health provider doing that hinders you from managing your blood pressure?

**Respondent: Pardon**

**Moderator:** You said that you go to either {Name of the hospital} or {Name of the facility}

**Respondent: Yes**

**Moderator:** What do you think that they are doing that hinders you from managing your blood pressure?

**Respondent: They serve me well**

**Moderator:** And you also told me that they give you information on what you are supposed to know

**Respondent: Yes**

**Moderator:** What about the government, what do you think that they are not doing and you feel that it would be better if they did

**Respondent: The should be assisting people financially**

**Moderator:** You have told me all this problems about the government and you said that your health providers are attending to you well and you also mentioned your individual problems like lack of money, challenges in getting food and you also said that the drugs are slightly expensive

**Respondent: Yes**

**Moderator:** So what do you think we can do to solve these issues?

**Respondent: It would be better if you helped people with drugs**

**Moderator:** And what else?

**Respondent: And finances**

**Moderator:** As a hypertensive patient, what can you do differently to manage your blood pressure?

**Respondent: You know sometimes pressure comes because of problems, you have problems, you think of what to do**

**Moderator: Ok**

**Respondent: So problems causes these sometimes**

**Moderator:** What do you think that you can do differently? Apart from what you are doing now

**Respondent: It would be better if I had a business**

**Moderator:** So that your brains can be settled

**Respondent: Yes**

**Moderator:** What is it that can be done differently by your health care provider or at the health care center?

**Respondent: Maybe they stop charging much. They should just charge what I can manage to pay**

**Moderator:** How has COVID19 affected the way you get hypertension care services in your community or either at {Name of the facility} or {Name of the hospital}?

**Respondent: With this COVID there has been a problem with getting money. There are many problems**

**Moderator:** What about you getting high blood pressure care services?

**Respondent: I was given drugs that can last me for a whole month when I went there**

**Moderator:** What did they tell you after giving you drugs?

**Respondent: I was told to go back when I finish the drugs**

**Moderator: On to the last question**

**Respondent: Yes**

**Moderator: What do you think that we didn’t talk about in regards to high blood pressure and you feel like it would be better if we talked about it?**

**Respondent: Pressure has a lot of problems**

**Moderator: Ok, are you done?**

**Respondent: Yes, am done**

**Moderator: Thanks for your time and for giving me this information,**

**…END…**
