# Supplementary material for: A socio-ecological framework examination of drivers of blood pressure control among patients with comorbidities and on treatment in two Nairobi slums; a qualitative study
Source: PLOS Glob Public Health. 2023 Mar 10;3(3):e0001625. doi: 10.1371/journal.pgph.0001625 (PMC10021823; doi:10.1371/journal.pgph.0001625)
Supplement: S1 File — (ZIP) [file pgph.0001625.s001.zip › Community/VIWA-IDI-UHTNC-200714_2212.docx]

**Moderator: {Name}**

**Code:** **VIWA-IDI-UHTNC-200714_2212**

**Moderator:** This community has been identified to have a high burden of uncontrolled hypertension which is a leading factor to premature deaths and disability. I am trying to gather information about hypertension care in your community. To avoid hypertension related complications, it is recommended that people with high blood pressure can change their lifestyles in regards to diet, physical activities, smoking, alcohol consumption and using blood pressure medication. So tell me about your experience with having high blood pressure. Tell me about your experience with having high blood pressure

**Respondent: This condition started in 1999**

**Moderator:** 1999?

**Respondent: Yes, that’s when I was diagnosed with blood pressure after my last delivery. I was just going for my normal clinic and that’s when I was told that I have that condition**

**Moderator:** Ok

**Respondent: And I accepted that I had it**

**Moderator:** How often do you check your blood pressure?

**Respondent: I have just been buying drugs for the last two months**

**Moderator:** Where do you go to check your blood pressure?

**Respondent: I told you its two months since I last checked**

**Moderator:** Where have you been going before?

**Respondent: The health Centre**

**Moderator:** Do you record down your blood pressure measurements after you have been checked?

**Respondent: I have a book though sometimes I just go to buy drugs when I don’t find them there**

**Moderator:** Can you remember what your last blood pressure measurement was when you checked 2 months ago?

**Respondent: It was 140/96 and the doctor told me that it was not so bad**

**Moderator:** Do you have any other condition apart from blood pressure?

**Respondent: No**

**Moderator:** So you only have blood pressure condition

**Respondent: I was diagnosed the other day with diabetes but I am trying to control it by taking drugs and watching my diet**

**Moderator:** When were you told that you are diabetic?

**Respondent: Like 4 months ago**

**Moderator:** Ok. Has the doctor ever told you what your target normal blood pressure should be?

**Respondent: He told me that it should be 130**

**Moderator:** 130 over what?

**Respondent: 130/90**

**Moderator:** Have your blood pressure tablets been reducing or increasing in number from 1999 to now?

**Respondent: You know blood pressure condition; it keeps on going high and low according to the moments that you get annoyed and you cannot avoid challenges. That’s normal**

**Moderator:** How many tablets are you taking for now?

**Respondent: I take 2**

**Moderator:** And how many were you taking before?

**Respondent: I used to take 1**

**Moderator:** So they added you from one to two?

**Respondent: Yes, they added me another one**

**Moderator:** What was the reason that the doctor gave you for adding you another tablet?

**Respondent: To control the first one**

**Moderator:** How has blood pressure condition affected your life?

**Respondent: I cannot understand because my dad had it so I dint know if it’s from my clan or what**

**Moderator:** You mean your dad had it?

**Respondent: Yes but I cannot tell if it is normal or it is just a disease**

**Moderator:** Apart from taking drug, what else do you do so as to manage your blood pressure?

**Respondent: By just trying to avoid thinking too much**

**Moderator:** You told me that you are so keen on your diet?

**Respondent: Yes, I try so hard to eat what I have been advised to eat**

**Moderator:** What about exercise?

**Respondent: I don’t do exercise. I am old**

**Moderator:** What else do you do to control your blood pressure apart from taking medicine and being keen on your diet?

**Respondent: I do business and sometimes I do walk from Eastleigh to Likoni. I don’t just sit down**

**Moderator: Ok**

**Respondent: I am very active**

**Moderator:** You told me that you go to the health Centre?

**Respondent: Yes but its two months since I last went there**

**Moderator:** Who were you seeing when you used to go to the health Centre?

**Respondent: She is called {Name}**

**Moderator:** What can you tell me in regards to the way she was attending to your blood pressure?

**Respondent: She is a good doctor. She just prescribes drugs. Actually she is the one that prescribed the second tablet**

**Moderator:** Have you ever sought hypertension care elsewhere apart from the health Centre?

**Respondent: No**

**Moderator: You have never gone elsewhere?**

**Respondent: There is a time I went to {Name of the hospital} but I stopped going there because it is very far**

**Moderator:** What services do you receive when you go to the health Centre?

**Respondent: They just check my blood pressure and my weight**

**Moderator:** How are their checkup services and drugs?

**Respondent: They are ok**

**Moderator:** How do you get your drugs?

**Respondent: Sometimes they just write the names of the drugs for me to go and buy when they are not available**

**Moderator:** So sometimes you get them or?

**Respondent: Yes. Sometimes we get the drugs and when we don’t get them we just buy**

**Moderator:** Ok, you have drugs that you are using now

**Respondent: Yes, I have to but even yesterday I bought.**

**Moderator: Alright**

**Respondent: I love my life**

**Moderator:** What challenges have you encountered as you try to manage your blood pressure?

**Respondent: There is no problem**

**Moderator:** So you get drugs, food and everything is ok?

**Respondent: I just buy drugs when I cannot get those that are offered for free**

**Moderator:** You buy?

**Respondent: Yes**

**Moderator:** What are the individual factors that can hinder you from managing your blood pressure?

**Respondent: Sometimes you think so much when you face challenges but that’s normal as I told you**

**Moderator:** And you also said that you are not young

**Respondent: I have 62 years**

**Moderator:** What are the community factors that might hinder you from managing your blood pressure?

**Respondent: Every household has issues. There is a time when my husband was sick and that was a challenge and I also face challenges with kids every now and then**

**Moderator:** What are the factors at the health center that hinder you from managing your blood pressure?

**Respondent: They just try hard to help us when we are sick because they give us drugs and advise us to on how to use them**

**Moderator:** What do you think your healthcare provider is not doing that might help you manage your blood pressure?

**Respondent: She always quarrels when we go late for clinic**

**Moderator:** What about the information that she gives you in regards to blood pressure? Does she train you?

**Respondent: I have never seen**

**Moderator:** She has never trained you on blood pressure

**Respondent: There is a time we get some information from Red Cross**

**Moderator:** What about the time that you take at the facility?

**Respondent: We are told to report at 9 and leave the hospital once the doctors have finished with you**

**Moderator:** How long do you take?

**Respondent: Like one hour**

**Moderator:** So it’s not bad

**Respondent: It’s ok**

**Moderator:** What do you think that the government is not doing and you think that I would be better in managing your blood pressure if they did?

**Respondent: I can’t know about the government to I go to a public Hospital and we get drugs from Red Cross through the government**

**Moderator:** What would be the solutions to the problems that you mentioned? Like for you as an individual you told me that sometimes you don’t have money to buy drugs

**Respondent: I just work hard because I can’t just neglect myself and die. This condition is very bad just like disability**

**Moderator:** What is it that doctors can do to manage your blood pressure condition?

**Respondent: I do pray because even the drugs that they give us can only work through prayers**

**Moderator:** What is it that can be done differently at the hospital?

**Respondent: I don’t think of anything that can be done**

**Moderator:** How has COVID affected hypertension care delivery in your community?

**Respondent: It has affected me because I don’t get money like I used to**

**Moderator:** Pardon

**Respondent: It has affected me because my business has gone down yet that’s where I get my rent food and I feed my kids from that. I told you that my husband is sick so he has just been at home because he cannot go to work. I could manage before but my business has really gone down**

**Moderator:** You told me that you husband has TB?

**Respondent: Yes, he was sick but for now he is ok**

**Moderator:** On to the last question

**Respondent: Ok**

**Moderator:** What do you think that we have not talked about in regards to blood pressure and you feel like talking about?

**Respondent: I don’t know if there is a way you can help me**

**Moderator:** Mmmhhh

**Respondent: You can help me get drugs if it is possible**

**Moderator:** Ok. I have heard

**Respondent: Ok**

**Moderator:** Thank you for the time that you have taken to give me that information and as we finish, I would like to tell you that we will come back to inform you on the outcome and we will know what we can do

**Respondent: Ok**

**…END…**
