# Supplementary material for: A socio-ecological framework examination of drivers of blood pressure control among patients with comorbidities and on treatment in two Nairobi slums; a qualitative study
Source: PLOS Glob Public Health. 2023 Mar 10;3(3):e0001625. doi: 10.1371/journal.pgph.0001625 (PMC10021823; doi:10.1371/journal.pgph.0001625)
Supplement: S1 File — (ZIP) [file pgph.0001625.s001.zip › Community/VIWA-IDI-UHTNC-200715_005.docx]

**Moderator: {Name}**

**Code:** **VIWA-IDI-UHTNC-200715_005**

**Moderator:** This community has been identified to have a high burden of uncontrolled hypertension which is a leading factor to premature deaths and disability. I am trying to gather information about hypertension care in your community. To avoid hypertension related complications, it is recommended that people with high blood pressure can change their lifestyles in regards to diet, physical activities, smoking, alcohol consumption and using blood pressure medication. So tell me about your experience with having high blood pressure. Tell me about your experience with having high blood pressure

**Respondent: I know my blood pressure because I go for clinic every month**

**Moderator:** For how long have you been having this condition?

**Respondent: For 24 years if am not wrong because it started when I got my last born and he is 24 years now**

**Moderator:** You told me that you attend clinic monthly

**Respondent: Yes, every month**

**Moderator:** Where do you go for clinic?

**Respondent: I used to go to {Name of the facility} but nowadays I go to {the health Centre}**

**Moderator:** Do they check your blood pressure when you go for clinic?

**Respondent: Yes, they do check pressure and blood sugar level but for now I don’t think that it is bad because I am maintaining. There is change**

**Moderator:** Do you have a record of your blood pressure measurements?

**Respondent: I have my monthly book**

**Moderator:** What were the measurements the last time you checked?

**Respondent: 144/90**

**Moderator:** Do you have any other condition apart from blood pressure?

**Respondent: I have arthritis**

**Moderator:** You told me that you go to {the health Centre}

**Respondent: Yes**

**Moderator:** How do they measure your blood pressure?

**Respondent: They use a gadget**

**Moderator:** How often do you check your blood pressure measurements? Do you check when you go there monthly or

**Respondent: I do check monthly but I do go to a private clinic located closer to my place for checkup just in case I feel like it is not ok. They allowed me to be checking for free because my blood pressure can rise on weekend when {the health Centre} is closed. I have to check if it is high because it goes high when I take salty food or if I delay in taking drugs. I now know how to control**

**Moderator:** You said that you go to {the health Centre}

**Respondent: Yes**

**Moderator:** Has your doctor at {the health Centre} told you the normal blood pressure target?

**Respondent: The right measurement should be around 120 something or 130 around there but according to age I was told that 130 or 140 is not bad**

**Moderator:** Tell me about drugs

**Respondent: The drugs that I am taking?**

**Moderator: Yes**

**Respondent: I am taking HTZ 1*1 and nephidepine 1*2**

**Moderator:** You have told me that you have been having blood pressure for 24 years

**Respondent: Yes**

**Moderator:** How many tablets did you start with?

**Respondent: the doctor has been changing my drugs. I have taken very many different types. There those that used to make my legs swell after taking them and my leg could go back to normal whenever I stopped taking the drugs. I have used many types**

**Moderator:** For how long have toy been using the two tablets that you are using now?

**Respondent: Its now two months since I changed from Cardinal that I took for long before I changed to nephidepine. With HTZ I have really used it for long**

**Moderator:** Ok, how has high blood pressure affected you?

**Respondent: Very bad because there is a time I was not following. I did not have time to take drugs that I was even taken to…5:53… (Not clear) but this time I have controlled. It was really tough for me because of blood pressure and ulcers. As a mother sometimes it is so challenging**

**Moderator:** Apart from taking drugs, how else do you manage your blood pressure? You had talked about diet

**Respondent: I was told that I am not supposed to take a lot of salt and again I used to love meat so much but nowadays I do vegetables a lot but just a little meat because meat is good but not too much of it. I used to take a lot of salt and I think that’s what was affecting me so much**

**Moderator:** What about exercise?

**Respondent: exercising is also good. I do train, I normally do press ups in the morning and you know my work involves a lot of walking and also I prefer walking when going to the market like { Name of a place}**

**Moderator:** Have you ever used traditional medicine?

**Respondent: For blood pressure?**

**Moderator: Yes**

**Respondent: No. I have never used traditional medicine fir blood pressure**

**Moderator:** Who do you see when you go to {the health Centre}?

**Respondent: What’s the name of the doctor at {the health Centre}? Last time we were given drugs by Caro**

**Moderator:** Is she a doctor or a nurse?

**Respondent: She is a nurse**

**Moderator:** What’s your view in regards to the way your health care provider

**Respondent: All along I used to go to another place; I have only gone to {the health Centre} for 2 months so I even don’t know the doctor who serves me. I used to go to Athi River {Name of the facility}**

**Moderator:** For how long have you gone to {the health Centre}?

**Respondent: It is now 2 months since I started going to {the health Centre}**

**Moderator:** Ok. How do they attend to you when you go there?

**Respondent: Very good. They are really nice and the challenges that I used to get at {Name of the facility} was that I was supposed to go there after 2 weeks and I was supposed to use transport charges but at {the health Centre} they said that one month is ok and they gave me drugs that could take me for three months. It’s good for now coz I don’t have to pay for transport like for {Name of the facility} where I could finish my drugs after two weeks and I was forced to buy but its ok with {the health Centre} because I don’t lack medicine**

**Moderator:** Why were you going to {Name of the facility} before?

**Respondent: Because {Name of the facility} is my outpatient hospital**

**Moderator:** You have insurance?

**Respondent: Yes**

**Moderator:** What were they telling you at {Name of the facility} in regards to blood pressure?

**Respondent: They used to tell me that it was bad because I couldn’t get the medicine that I was using so they were giving me other drugs to try other times my blood pressure was low, my legs were swollen so they could tell me to try different drugs after two weeks**

**Moderator:** What kind of services do you receive at {the health Centre}?

**Respondent: I saw that it was ok there because they check everything. They can’t give you drugs without checking on you and again they have drugs and they give you medicine when they see that your pressure is doing well**

**Moderator:** Do they advise you?

**Respondent: Yes, they do teach us. There is a time I was with {Name of the hospital} patients. Sometimes we are called to attend training and counseling on the right thing that we are supposed to do or how we are supposed to live**

**Moderator:** You also told me that you have been going {the health Centre} for two months. Do you attend your clinic monthly?

**Respondent: Yes, I have a clinic next month. I am supposed to go for my clinic on October 10^th^**

**Moderator:** Ok. Do you have a problem in managing your blood pressure?

**Respondent: As for now?**

**Moderator:** Looking at you as an individual, you told me that it was hard for you to go to {Name of the facility} because of transport charges

**Respondent: Yes and it was hard for me because I used to finish my drugs within two weeks**

**Moderator:** Looking at your age, is it a problem in managing your blood pressure?

**Respondent: Pardon**

**Moderator:** Looking at your age, is it a problem in managing your blood pressure?

**Respondent: I don’t see any problem because I see that the service is ok**

**Moderator:** You told me that you have arthritis

**Respondent: Yes**

**Moderator:** Do you have any problem in managing your blood pressure. Looking at you as an individual, you told me that you have arthritis apart from blood pressure. Do you think that it is a challenge in managing your blood pressure?

**Respondent: I have arthritis but I no longer have ulcers**

**Moderator:** What about the way you take your drugs?

**Respondent: I don’t have any side effects**

**Moderator:** You don’t see any challenge in managing your blood pressure

**Respondent: For now I don’t have any problem**

**Moderator:** Do you use either alcohol or cigarettes?

**Respondent: No I don’t use any**

**Moderator:** From the family side, looking at the community and your family, do you see any challenge in managing your blood pressure?

**Respondent: My mother had blood pressure condition and that’s what killed her. We are 3 in our family who have this problem including my mum who passed away**

**Moderator:** Sorry. Looking at the environment where you stay, you had told me that you used to like meat a lot.

**Respondent: Yes**

**Moderator:** Are there any other foods that are not god and you are using them?

**Respondent: Pardon**

**Moderator:** Are you using foods that are not good?

**Respondent: With food we just eat what we can get. You cannot control what you eat here. I just eat what I get but I know that I am supposed to take a lot of vegetables but I eat what I get**

**Moderator:** Do you have any problem with your health providers in regards to the way he is managing your blood pressure?

**Respondent: I don’t have a problem with that**

**Moderator:** Are they attending to blood pressure patients well?

**Respondent: They are very ok**

**Moderator:** What of the information that they give you?

**Respondent: It’s ok**

**Moderator:** Looking at drugs, do you get all the drugs at the hospital?

**Respondent: We get them**

**Moderator:** There is noise there. Please move to a place where there is no noise

**Respondent: I am in the field nut you can now talk**

**Moderator:** How is the infrastructure at {the health Centre}?

**Respondent: It’s ok**

**Moderator:** Looking at the policy makers, do they have a problem in managing your blood pressure?

**Respondent: There is no problem**

**Moderator:** On the government side

**Respondent: There is no problem**

**Moderator:** Ok, what do you think can be the solution to the hindrances that you have mentioned? You told me that finance is one of the challenges

**Respondent: Yes**

**Moderator:** Can you give me a solution to that?

**Respondent: Solution to what?**

**Moderator:** Can you give me a solution to the financial hindrance that you mentioned in regards to the way you are managing your blood pressure?

**Respondent: On money issue?**

**Moderator:** Yes

**Respondent: Getting money to buy food is a challenge and sometimes it is a big challenge when you have a big family because you are not working**

**Moderator:** You also told me that you eat what you get. What do you think can be the solution?

**Respondent: pardon**

**Moderator:** What would be the solution to that?

**Respondent: I said that I must save for me to eat the right food because it is for my own life and it is much better when I have money. Money is everything**

**Moderator:** What can you do differently as an individual in regards to blood pressure?

**Respondent: What I am doing differently is the business that I am running so that I can be able to get money. I just cannot sit there**

**Moderator:** What about your health care provider?

**Respondent: Sometimes I go to afya center for training**

**Moderator:** What about at {the health Centre} health center, what is it that can be done differently?

**Respondent: We just go for training and we also refer others or teach them**

**Moderator:** How has COVID 19 affected the way you get hypertension care services in that community?

**Respondent: COVID19 has caused problems because I was a business woman. I used to transport some things to my rural home but for now I cannot**

**Moderator:** What about the way you receive your hypertension care services? How has it affected you?

**Respondent: It has affected me because I can’t balance. I have to think on how I will feed my kids because I used to give them good care when I used to get money but for now I can’t**

**Moderator:** Is there any other thing that you would like to talk about in regards to high blood pressure?

**Respondent: For now I don’t have, I think we have mentioned everything unless you tell me**

**Moderator:** I wanted to hear from your point of view

**Respondent: I just advice those who have this condition to follow what we are told and for me to keep my time because it is for my own good**

**Moderator:** Ok, thank you so much for the time that we have had and I am happy with the conversation that we have had and I know that it will help us in our research

**Respondent: Ok**

**…END…**
