# Supplementary material for: A socio-ecological framework examination of drivers of blood pressure control among patients with comorbidities and on treatment in two Nairobi slums; a qualitative study
Source: PLOS Glob Public Health. 2023 Mar 10;3(3):e0001625. doi: 10.1371/journal.pgph.0001625 (PMC10021823; doi:10.1371/journal.pgph.0001625)
Supplement: S1 File — (ZIP) [file pgph.0001625.s001.zip › Community/VIWA-IDI-UHTNC-200715_0558.docx]

**Moderator: {Name}**

**Code:** **VIWA-IDI-UHTNC-200715_0558**

**Moderator:** This community has been identified to have a high burden of uncontrolled hypertension which is a leading factor to premature deaths and disability. I am trying to gather information about hypertension care in your community. To avoid hypertension related complications, it is recommended that people with high blood pressure can change their lifestyles in regards to diet, physical activities, smoking, alcohol consumption and using blood pressure medication**.** So tell me about your experience or how you have been since the day that you were diagnosed with hypertension

**Respondent: I was diagnosed with blood pressure before diabetes and I have been taking antihypertensive for the last six years. I came to know that I am diabetic three years**

**Moderator:** So which one came first, diabetes or blood pressure?

**Respondent: I was diagnosed with hypertension first**

**Moderator:** Which year?

**Respondent: I can remember, maybe if I check my book later**

**Moderator:** Ok, there is no problem

**Respondent: Ok**

**Moderator:** So you were diagnosed with blood pressure then realized that you are diabetes later

**Respondent: Yes, I have been diabetic for three years and hypertensive for six years**

**Moderator:** You have had blood pressure for 6 years and diabetes for 3 years?

**Respondent: Yes**

**Moderator:** Ok. How often do you check your blood pressure measurements?

**Respondent: It was very high, it was 200 when I checked on Friday the other week and that’s why I was told to go for checkup daily. I have just checked now and I found that it was 195**

**Moderator:** Over what?

**Respondent: I don’t remember but I have been told that the bottom one was ok but the upper one was not ok**

**Moderator:** And you told me that you have been hypertensive for like 6 years?

**Respondent: Yes, six years**

**Moderator: Do you record somewhere when you go for blood pressure checkup?**

**Respondent: I was at my rural home and so I was not able to attend most of the clinics there but I normally go when I feel unwell and I have a book where I write in every time that I have been treated**

**Moderator:** And you said that you have been diabetic for the same number of year?

**Respondent: Yes**

**Moderator:** Has the doctor ever told you what your normal blood pressure target should be?

**Respondent: I don’t remember but he told me that it is supposed to be 130**

**Moderator:** He told you that it should be 130?

**Respondent: Yes, 130 but I don’t remember it should be over what**

**Moderator:** Tell me about the number of tablets that you have been taking for the last six years. How many tablets have you been taking?

**Respondent: I used to take my medicine 2 times a day**

**Moderator:** Just antihypertensive?

**Respondent: Yes, for hypertensive I used to take drugs in the morning and evening and when it got better I started taking drugs once per day**

**Moderator:** So you started taking once per day?

**Respondent: Yes. Only in the evening and later on it became worse and I was added other tablets so I was taking two tablets twice a day but it was again changed later. I used take drugs at mid-day, in the morning and in the evening. Thrice a day**

**Moderator:** Thrice a day?

**Respondent: Yes, per day. I took them that way for a while but currently I do take drugs twice a day**

**Moderator:** So you take drugs in the morning and evening only?

**Respondent: Yes, that’s not so bad**

**Moderator:** Did the doctor tell you the reasons that made him change?

**Respondent: My blood pressure was so high and it was not getting low. He gave me another tablet that I was told to put it under my tongue. It was so high on that day**

**Moderator:** What can you tell me in regards to the way blood pressure has affected your life?

**Respondent: It has affected me in many ways. I can’t work when my blood pressure us high. I work at a hotel and I have somebody to help me and I don’t do anything when I come back at tome. Someone has to bring me water so that I can take a bath and I don’t stay alone because there are times when my blood pressure**

**Moderator:** Apart from taking drugs, what else do you do so that your blood pressure can be at the level that the doctor advised you?

**Respondent: My condition worsens when I think so much or if i watch or hear anything**

**Moderator: What about food?**

**Respondent: I was told that I should be eating traditional vegetables, fruits and I can also take milk if I can find it, liver, beans, watermelon, oranges but in small portions**

**Moderator:** You were talking about you weight

**Respondent: I was weighing 95kgs**

**Moderator:** Then?

**Respondent: I am weighing 74kgs**

**Moderator: Oh, that’s nice .What else are you doing to control your blood pressure apart from reducing your weight and watching your diet?**

**Respondent: I had to reduce my weight because I was eating meat a lot and other oily foods but I stopped eating that. Nowadays I just eat liver when I can get it. I stopped using sugar, eggs that I used to take a lot. There are many foods that I don’t use nowadays**

**Moderator:** Who do you see when you go for your clinic?

**Respondent: A doctor**

**Moderator:** What can you say in regards to the way this doctor managed your blood pressure?

**Respondent: He checks my blood pressure measurements, I wait for five minutes and he checks my blood pressure measurement again then I leave**

**Moderator:** Have you sought care elsewhere apart from where you have always been going?

**Respondent: I have gone to many places. I go to another facility when I visit my rural home and I also used to go to at…is it called {Name of the Hospital} that is located in {Name of a place} village. I am not sure of the name but I will tell you if I remember. I used to go there for clinic before I went to my rural home and when I came back I started going to this facility that is close to where I stay**

**Moderator:** What do they tell you about your blood pressure condition in all the facilities that you have visited?

**Respondent: There are times when it is bad and sometimes it is ok. It is not bad always**

**Moderator:** You told me that the facility that you go to for clinic is located close to your place and it is easier for you to access services

**Respondent: Yes**

**Moderator:** Which services do you get when you go for blood pressure checkup?

**Respondent: I told you they check my blood pressure, sometimes they check my sugar levels and they also advise me to avoid thinking. They treat me well**

**Moderator:** What challenges do you face as you try to control your blood pressure?

**Respondent: I face a lot of challenges like buying drugs. I have never gone to register like other patients. I do send people to but me drugs at Mumbi house and when I don’t have enough money I just buy them within the village**

**Moderator: What about you taking medicine at the right time?**

**Respondent: When I get home I take shower first then I take my hypertensive and diabetes drugs then I wait for like 30 minutes then I eat and after am done with eating, I take milk then I sleep**

**Moderator:** Ok

**Respondent: I do sleep at 9:00pm**

**Moderator:** How old are you?

**Respondent: Am 62 years old**

**Moderator:** What are the family or community factors that might hinder you from managing your blood pressure? You told me that sometimes you get so many thoughts

**Respondent: Yes, I do have many thoughts, my business is not doing well and I have my grandchildren with me and I have to feed them so I must think. I also have one great grand child**

**Moderator:** What do you think that your care giver is not doing and if he did that then your blood pressure would be normal?

**Respondent: I don’t know because he is the expert. He tell me how I should live and he gives me drugs and advices me on how to take them**

**Moderator:** Do you receive training on hypertension when you go to the hospital?

**Respondent: Yes. We are trained**

**Moderator:** What about the time that you go for clinic? How is it?

**Respondent: We are not many patients at the clinic. I normally go there in the afternoon but when we have training I do go for clinic at 11am them I go back to the hotel**

**Moderator:** How do you get your drugs at the hospital?

**Respondent: I don’t get drugs there. You know that is a private hospital so for drugs I normally send somebody to buy me drugs at Mumbi house**

**Moderator:** What about the government? What can they do to help you manage your blood pressure?

**Respondent: They should give us drugs because like me I have children and grandchildren and so I have to think because sometimes we do lack like for now we there is corona and my job is not doing well. Sometimes we sleep hungry and i am taking medicine**

**Moderator:** What can you do as an individual to control your blood pressure?

**Respondent: If I get another job to do apart from running this hotel because I don’t like borrowing, I am used to hustling for myself. I can leave this hotel business if I can get some other job because fire even not good. I have nothing else to do for now and I don’t have someone to help me. I just have to go there**

**Moderator:** What do you think your doctor or your health care provider can do differently?

**Respondent: I don’t know. I just told you he is the expert. He is the one who checks me and he knows me very well but he never tells me. I can’t know and I can’t say that he has not treated me. Is that bad?**

**Moderator:** About the hospital you said that you would like them to give you drugs?

**Respondent: Yes**

**Moderator:** How has COVID19 situation affected how you get hypertension care in your community?

**Respondent: Am told that there those that get drugs but I have never gone there. I was told that they collect the drugs at the Health Center**

**Moderator:** You have never gone to the health Centre?

**Respondent: No**

**Moderator:** You told me that there are people who collect things from {the health Centre}

**Respondent: Yes**

**Moderator:** People with which high blood pressure condition or?

**Respondent: Those that have high blood pressure and diabetes get drugs there**

**Moderator:** They get the drugs for free or what were you told?

**Respondent: They get drugs for free**

**Moderator:** Ok, I have not heard that before, am getting it from you

**Respondent: I have never gone there**

**Moderator:** Do you know of anybody who gets drugs there for free?

**Respondent: Yes, I met one of them as I was coming from town to buy drugs and she asked me why I do buy drugs yet they are given drugs that can last them for 3 months at {the health Centre}**

**Moderator:** Is {the health Centre} Health Center a public hospital?

**Respondent: It is managed by the county council**

**Moderator:** Ok. On to the last question

**Respondent: Yes**

**Moderator:** What else do you think we can talk about in regards to blood pressure?

**Respondent: I don’t have another thing but I would like to ask a question**

**Moderator: Yes**

**Respondent: What causes people to feel weak and stagger when blood pressure is high?**

**Moderator:** It would be better if you told your health care provider about the problem because he is the one that started with you and he is the only one who can know where the problem is and am sure he notes all those things somewhere

**Respondent: I asked him and he told me that sometimes I get weak when I do heavy work, I can even fall down some time. He advised me to be sitting down when I feel that way and I continue walking when I feel good**

**Moderator:** Just follow what your doctor told you because I can tell you to do a different thing from what he told you yet he is the one that has been following you from the start. It wouldn’t be good if we say different things from what he has been telling you

**Respondent: Ok**

**Moderator: Thank you for your time and I appreciate for the information that you have given me about yourself**

**Respondent: Ok**

**…END…**
