# Supplementary material for: A socio-ecological framework examination of drivers of blood pressure control among patients with comorbidities and on treatment in two Nairobi slums; a qualitative study
Source: PLOS Glob Public Health. 2023 Mar 10;3(3):e0001625. doi: 10.1371/journal.pgph.0001625 (PMC10021823; doi:10.1371/journal.pgph.0001625)
Supplement: S2 File — (ZIP) [file pgph.0001625.s002.zip › Health Facility/KOCH_KII_HP_200531_1126272.docx]

**Moderator: {Name}**

**Respondent: Health Care provider**

**Code: KOCH-KII-HP-200531-1126272**

**Moderator: So I have a few questions that I have to read just to confirm what I had read to you**

**Respondent: Mmmmhh**

**Moderator:** So for these questions, everything that you heard me tell you just confirm by saying yes as I tick on my side. Ok

**Respondent: Ok**

**Moderator:** Personally I confirm that I have read and that you have understood the information sheet for the above study and you have had opportunity to ask questions and the questions you asked have been answered to your satisfactory

**Respondent: Ok**

**Moderator:** Ok or Yes

**Respondent: Yes**

**Moderator:** You understand that your participation is voluntary and you are free to withdraw from this study any time without being affected in terms of your legal rights

**Respondent: Yes**

**Moderator:** you understand that the data collected may be looked at by other individuals taking part in this study and you give permission for these individuals to have access to your data

**Respondent: Yes**

**Moderator:** You confirm consenting to this, to being audio recorded and you also consent that we use your data without, I mean while using your data we will use anonymized verbatim quotations

**Respondent: Yes**

**Moderator:** Are you happy for your data to be used for future research

**Respondent: Yes**

**Moderator:** So, Meaning that you agree to take part in this study

**Respondent: Yes**

**Moderator:** To start with, I have a few questions here that I want us to discus in relation to what I have told you

**Respondent: Mmmmhh**

**Moderator:** So, on to the first one. Just to explain a little bit

**Respondent: Ok**

**Moderator:** You understand the community that you are working with has been identified to have a burden of hypertension

**Respondent: Yeah**

**Moderator:** Which has been a leading risk to, leading risk to premature deaths and disabilities. So we are trying to gather information about provision of hypertension care in this community and am seeking to review your, I mean am seeking your views about uncontrolled hypertension in this community and factors driving to these high rates

**Respondent: Ok**

**Moderator:** So please tell me about hypertension in this community

**Respondent: First of all maybe I’ll define hypertension. Hypertension is high blood pressure that is caused maybe by stress**

**Moderator:** Yeah

**Respondent: Diet and maybe lack of exercise**

**Moderator:** Yeah

**Respondent: So in our community maybe hypertension is at high because people from this community are struggling to make a living that leading to stress so most suffer from the same high blood pressure because of being unable to manage stress**

**Moderator:** Could there be anything that you would like to add to what you have just said?

**Respondent: I think it’s just that**

**Moderator:** So, narrowing it down to your facility,

**Respondent: Yeah**

**Moderator:** Would you mind telling me about the hypertensive clinics in your facility, the facility that you are working with

**Respondent: In our clinic we don’t have specific clinics for these hypertension patients. We just attend to them on daily basis just as they come**

**Moderator:** So how do you go about it?

**Respondent: First of all clients are screened by doing vitals tests at the entry point in the facility**

**Moderator:** Yeah

**Respondent: One of the vitals is taking blood pressure by using the blood pressure machine**

**Moderator:** Mmmmhh

**Respondent: So those who got high blood pressure are isolated and put on medication and then follow up**

**Moderator:** Ooohh Yeah. And how do you do your follow ups?

**Respondent: By use of CHVs, we give them cards and then TCAs and then we do follow up by calling them to remind them that they are supposed to come to the clinic as soon as their medicine are done**

**Moderator:** Mmmmhh

**Respondent: Or any other thing that we need to do for them**

**Moderator:** Thank you. And as you do these within your facility, do you have any national guidelines that you follow as you do your daily duties in regards to hypertension?

**Respondent: Yes we do**

**Moderator:** So these guidelines, can I reach them or can I get to see them or how can I get them?

**Respondent: When you just reach at the facility you will just see them coz they are all over. The guidelines are in the outpatient part of the facility**

**Moderator: Ooooh**

**Respondent: Mmmmhh**

**Moderator: So the only way I can get them is by me coming to the facility, is that what you mean?**

**Respondent: Coming to the facility and I think we have a soft copy. I can as well send you**

**Moderator:** Ok fine. Ill appreciate if at all I can get the copy. The soft copy

**Respondent: on to the next question**

**Moderator:** Aaahh, do you see patients with hypertension and other conditions?

**Respondent: Yes we do**

**Moderator:** So what are…

**Respondent: Conditions… like mostly patients with hypertension most have diabetes**

**Moderator:** Mmmmhh

**Respondent: So you have to manage it too**

**Moderator:** Apart from diabetes, is there any other condition that you can mention?

**Respondent: I think just diabetes and maybe because of hypertension, their BMI is high, obesity, weight. These things yeah**

**Moderator:** And how do you manage these patients when they come to your facility?

**Respondent: How we manage patients with hypertension and other illnesses?**

**Moderator:** Yes, yes, yes

**Respondent: Of course we give them priority and we give them medication that someone with hypertension takes coz there are those medications that when you take and you have hypertension they can lead to diabetes and if you have diabetes can increase your blood pressure**

**Moderator:** Eeeehh

**Respondent: So we are so cautious and they are just treated like special clients**

**Moderator:** Mmmmhh

**Respondent: Other than those with only hypertension**

**Moderator:** Ohh, yeah

**Respondent: Yes**

**Moderator:** As a person that attend to these clients that have hypertension issues,

**Respondent: Yeah**

**Moderator:** what factors do you consider to be like good or bad in control of ahhh, blood control? I mean blood pressure control

**Respondent: There are defaulters**

**Moderator:** Yeah

**Respondent: That’s one factor, there are patients we book them for clinic for example a patient come today**

**Moderator:** Yeah

**Respondent: Turns that he has blood pressure, high blood pressure, you give them drugs and then you tell them to come back on another date**

**Moderator:** Mmmmhh

**Respondent: A client doesn’t come coz maybe they took the medicine ad feels that am good so they decide just not to come**

**Moderator:** Mmmmhh

**Respondent: So when they come back its just worse. So one factor is defaulters**

**Moderator:** Apart from defaulters?

**Respondent: Apart from defaulters, maybe transport, transport to come to the facility. Maybe is their due date to come to the facility, someone doesn’t have means to get to the facility.**

**Moderator:** Mmmmhh

**Respondent: Another factor is absence of drugs, sometimes we ran out of drugs and if you advice a client to get drugs maybe they ran out of money.**

**Moderator:** Mmmmhh

**Respondent: So they cannot find the drugs thus missing their doses**

**Moderator:** Mmmmhh

**Respondent: And maybe history of diabetes or cardiac problems and has high blood pressure**

**Moderator:** Mmmmhh

**Respondent: It’s very difficult to manage this patient**

**Moderator:** Mmmmhh

**Respondent: Yeah, coz there is drugs that you can give a patient with high blood pressure they can lead to problem with heart and already this patient has a problem with heart thus leading to management to be fatal**

**Moderator:** I have heard the negative or the bad habits of control of blood pressure, could there be factor that you can mention that are positive?

**Respondent: Positive?**

**Moderator:** The good factors that are associated with blood pressure

**Respondent: Management**

**Moderator: Yes**

**Respondent: Maybe the good factor is, the good factor that I can say is patients adhering to medication**

**Moderator:** Mmmmhh

**Respondent: Watching their diet and not defaulting on their return dates for clinics**

**Moderator:** Yeah. Is there any other thing you can add?

**Respondent: Blood pressure is not curable but you can manage it**

**Moderator:** Mmmmhh

**Respondent: So if clients know that it is manageable and just try to enlong the life span of this person**

**Moderator:** Mmmmhh

**Respondent: So for me to say that I have controlled this blood pressure**

**Moderator:** Mmmmhh

**Respondent: Every time the patient come back is reporting good news because they are doing what you asked them to do**

**Moderator:** Mmmmhh

**Respondent: Yeah**

**Moderator:** Apart from what you have mentioned on good and bad factors associated with blood pressure control, could there be anything else you would like to add in relation to that?

**Respondent: in relation to factors associated with blood pressure control?**

sure

**Respondent: I think am done**

**Moderator:** Mmmmhh

**Respondent: At my level am done?**

**Moderator:** What challenges do you encounter in provision of hypertension services?

**Respondent: Challenge number one, stock out of drugs**

**Moderator:** Mmmmhh

**Respondent: You have diagnosed this patient with hypertension**

**Moderator:** Mmmmhh

**Respondent: You have asked them to come back for their medication**

**Moderator:** Mmmmhh

**Respondent: Maybe during their returning there are no medications**

**Moderator:** Mmmmhh

**Respondent: Because of course they know these medications are free**

**Moderator:** Mmmmhh

**Respondent: You see that’s challenge number one, stock out of drugs**

**Moderator:** Yeah

**Respondent: Challenge number 2, work load**

**Moderator:** Mmmmhh

**Respondent: We don’t have specific dates for these clients**

**Moderator:** Mmmmhh

**Respondent: One, they just come**

**Moderator:** Mmmmhh

**Respondent: One, your return date depends with the day you were diagnosed or maybe when your medicine will be done**

**Moderator:** Mmmmhh

**Respondent: So you are coming to this facility maybe on a Monday and we have other services**

**Moderator:** Mmmmhh

**Respondent: So am overworked so am just telling you ahhh. Maybe am not even giving you that attention**

**Moderator:** Mmmmhh

**Respondent: So you are not getting good services from me coz am overworked**

**Moderator:** Mmmmhh

**Respondent: Workload, challenge number two**

**Moderator:** Mmmmhh

**Respondent: Another challenge is patients with other illness like I told you earlier**

**Moderator:** Mmmmhh

**Respondent: It is a factor and is a challenge too**

**Moderator:** Mmmmhh

**Respondent: You are supposed to be so cautious on which drugs you are giving this client for you to save them from hypertension and the other illness**

**Moderator:** Mmmmhh

**Respondent: And still another challenge is maybe patient perspective.**

**Moderator: Mmmmhh**

**Respondent: Patients just, for example a patient believe is a family thing**

**Moderator: Mmmmhh**

**Respondent: So if you give them drugs they just tell you that in our family my grand pa, may dad has hypertension so hypertension is not curable**

**Moderator: Mmmmhh**

**Respondent: So you are trying to manage this problem but the patient know that they just care for the …**

**Moderator: Ok**

**Respondent: Yeah**

**Moderator:** I understand that you have mentioned challenges in relation to patients’ ignorance, understaffing and under stocking of drugs and I would like us to talk also about… do you find working hours as a challenge?

**Respondent: As per now or? Yeah it’s a challenge**

**Moderator:** Yeah

**Respondent: For example you have a hypertensive client who is working and maybe for our facility we attend to these clients in the morning hours, one to two.**

**Moderator:** Mmmmhh

**Respondent: A patient comes at two when there is no a care giver,**

**Moderator:** Mmmmhh

**Respondent: Maybe yeah. She is told to come tomorrow coz there is no a caregiver coz he or she is late**

**Moderator:** Mmmmhh

**Respondent: And may be the patient was at work and he or she decide to pass by to check her level at the facility**

**Moderator:** Mmmmhh

**Respondent**: **So it’s also a challenge**

**Moderator:** Oooohh, and in regards to drugs or medication stock out,

**Respondent: Yeah**

**Moderator:** I understand that you have talked much about that

**Respondent: Yeah**

**Moderator:** Could there be anything else you would like to add in relation to that?

**Respondent: Unavailability of drugs**

**Moderator:** Yeah

**Respondent: Is also a challenge coz you are trying to control this blood pressure at least these patient do not come to the facility every now and often**

**Moderator:** Mmmmhh

**Respondent: So the other time patients come they get drugs, the other time you are sending them to buy and they don’t find the drug**

**Moderator:** Mmmmhh

**Respondent: They just sit back at home and maybe come back when the situation is worse and maybe when they come there are no drugs**

**Moderator:** Mmmmhh

**Respondent: That’s also a challenge. Unavailability of drugs at the facility**

**Moderator:** I understand that you had also mentioned an issue about work load and staff in your facility

**Respondent: Yeah**

**Moderator**: Is there anything you can add in s there anything you can add in regards to that workload

**Respondent: As in as a solution or as a challenge**

**Moderator:** As a challenge and you can also mention a solution to that

**Respondent: What I think about workload, for us we do daily clinics to these clients as much as they are available**

**Moderator:** Mmmmhh

**Respondent: So for workload I think we schedule for their special clinic. Like we just schedule like 3 days of the week where a client knows, even come one knows that from this hour to this hour we are attending to hypertensive clinic**

**Moderator:** Mmmmhh

**Respondent: Maybe that could help**

Moderator: ooohh…thank you

**Respondent: Yeah**

**Moderator:** On to these other question, we have these factors that contribute to uncontrolled hypertension to the patients that you attend to

**Respondent: Mmmmhh**

**Moderator:** And these factors can be categorized as either individual factors or patients’ factors, I mean factors caused by patients, others are caused by the community or family level, there are those that are caused by health system or the policy level perspectives

**Respondent: Mmmmhh**

**Moderator:** So I would like to get your opinion on all the perspectives that I have mentioned starting from individual level. I understand you have talked a lot about individual level where you mentioned about ignorance, the issue of costs, getting drugs

**Respondent: Mmmmhh**

**Moderator:** So is there anything you would say much in regards to individual factors that contribute to uncontrolled hypertension?

**Respondent: As per me?**

**Moderator:** Yeah

**Respondent: Factors that lead to uncontrolled hypertension, as per me I see maybe not scheduling the clinics for the clients as per the clinic**

**Moderator:** Yeah

**Respondent: Unavailability of drugs then defaulter tracing process by the CHVs**

**Moderator:** Mmmmhh

**Respondent: Yeah**

**Moderator:** On patients like the individual level, what would you say are the factors or what are they doing that contributing to uncontrolled hypertension

**Respondent: Maybe not watching their diet**

**Moderator:** Mmmmhh

**Respondent: Not coming for their clinics**

**Moderator:** Yeah

**Respondent: Defaulting medicine when they feel better**

**Moderator:** Yeah

**Respondent: Not doing exercise**

**Moderator:** Mmmmhh

**Respondent: Unable to manage stress**

**Moderator:** So when... You have mentioned stress, defaulters and other factors,

**Respondent: Yeah**

**Moderator:** Looking at the community level, at the community level, what are the factors that may contribute to uncontrolled hypertension?

**Respondent: Maybe myths and misconceptions**

**Moderator:** Pardon

**Respondent: Myths and misconceptions in the community about hypertension**

**Moderator:** Would you mind expounding further on that?

**Respondent: In the community of course people believe that hypertension kills, so someone is not supposed to be stressed when they find out they are hypertensive**

**Moderator:** Mmmmhh

**Respondent: So once someone is diagnosed with hypertension and of course he or she has ever heard someone say that if you have pressure then just start counting your number of days to live on this earth**

**Moderator:** Mmmmhh

**Respondent: So someone just have negative attitude towards maybe medication. Even if they come for clinics, back in the community they know that we are caring for the dying even if we are trying our level best**

**Moderator:** Ok, and at the health level, health system levels, what can you say about that?

**Respondent:** Health system level, maybe unavailability of the drugs and not prioritizing these clients

**Moderator:** Pardon

**Respondent: Unavailability of drugs and not giving these clients priority**

**Moderator:** Any other thing?

**Respondent: At the health facility level?**

**Moderator:** Yeah

**Respondent: Same workload, say you are alone in the facility and these clients need attention and you have other clients to attend to.**

**Moderator:** Mmmmhh

**Respondent: Maybe mothers in labor, others in ANC, children in clinic, so you are not giving these clients full attention. So that’s a factor that can also lead to uncontrolled hypertension**

**Moderator:** You have mentioned a lot on individual, community and health system, could you have anything that you can say at the policy level?

**Respondent: At the policy level?**

**Moderator:** Yes

**Respondent: Maybe according to the policies, according to the guidelines, we have policies on how to manage hypertension**

**Moderator:** Mmmmhh

**Respondent: Maybe there is something else I can add on, so someone is just following guidelines.**

**Moderator:** Mmmmhh

**Respondent: For example you were told; let me go out of the topic. You are told that you are supposed to resuscitate a baby for 30 minutes, if the baby is not responding pronounce them dead**

**Moderator:** Mmmmhh

**Respondent: But you can just go beyond that and maybe this baby will survive, same to hypertension, policies say, give these medication to these level and if and if the pressure, high blood pressure is not being controlled then that patient you just care for the dying**

**Moderator:** Mmmmhh

**Respondent: But you can do good more than what the policy says**

**Moderator:** Thank you for what you have given me, we are almost done but before we wind up, I understand that you have mentioned a lot of factors that can lead to uncontrolled hypertension but in your views, I would like we talk about possible solutions to the problems that you have mentioned. For example at individual level you mentioned the exercise, you mentioned defaulting and you mentioned dieting

**Respondent: Yes**

**Moderator:** What would you advice to be the possible solutions to such factors?

**Respondent: to such factors I would advise clients with hypertension to eat, as in under diet food less in sodium coz a lot of sodium increases water in the body**

**Moderator:** Mmmmhh

**Respondent: So blood is pumping so high in the arteries thus increasing the level of blood pressure**

**Moderator:** Mmmmhh

**Respondent: So for me ill advise them to eat food low in sodium that is salt, do a lot of exercises so that they can maintain their body weight so that when their BMI is calculated is not abnormal**

**Moderator:** Another solution?

**Respondent: That’s solution number one, another solution, maybe generally not as per patient**

**Moderator:** Actually you had given me factors that I have mentioned to you. You mentioned exercise, you mentioned matters on defaulting. Like I would like to know, how can we control this issue of defaulting?

**Respondent: Maybe we can use CHVs. When you get a client, first when you diagnose a patient with hypertension, you give then a CHV who will be calling them to know where they stay even locations and even if it’s possible they pick up their medicine for them which they are taking and to be reminding them of their clinics. For those who can’t make it to the clinic, they can take the medicine to where they can access them**

**Moderator:** Any other thing you would like to add in regards to that?

**Respondent: On defaulter tracing?**

**Moderator:** Generally, any other solution to what you have mentioned base on individual level

**Respondent: Maybe I can say generally, availability of drugs in the facilities coz these are patients with chronic illnesses that are not curable. So if they miss drugs they can just succumb to death coz of cause hypertension kills**

**Moderator:** Yeah

**Respondent: So they should have their drugs, like they should not miss drugs. Of course from the community people are, let me say the economy is poor and some of them don’t have white collar jobs so they just get that little amount of money from pocket to mouth**

**Moderator:** Mmmmhh

**Respondent: So if you don’t avail drugs to them then you will not help them**

**Moderator:** Oh yeah, and you mentioned something about myths and misconceptions that people understand within this community. How do we solve this issue?

**Respondent: By outreaches to talk about hypertension, causes, management and that to assure the community that if someone is diagnosed with hypertension, they can as well live as long as the other people so long as their diagnosis is well controlled by use of medication despite being a chronic illness**

**Moderator:** Ok. On to the next question, we understand that there are cases of COVID 19 that is all over the country, actually all over the world and it has been affecting different areas not forgetting the health sector. So as a health care provider, how do you say that this COVID 19 thing has affected your provision of care to hypertensive patients in your community?

**Respondent: It has really affected coz for now every facility everywhere in the whole world are prioritizing patients with COVID19**

**Moderator:** Mmmmhh

**Respondent: So people have forgotten to do the vital signs for other clients like hypertension clients for our case now. They are just screening those people for COVID19**

**Moderator:** Mmmmhh

**Respondent: There is just too much into the COVID19 other than these clients for hypertension. For example in our clinic what is being done is just temperature checking**

**Moderator:** Mmmmhh

**Respondent: for you to get a patient with hypertension you should do what? do blood pressure. So prioritizing clients with COVID19 has also affected hypertensive clients**

**Moderator:** Mmmmhh

**Respondent: Patients with chronic illness are being told that they are prune to COVID19. Right? So they are afraid of coming to the facility coz they know that they are eligible or they are exposed to this COVID19**

**Moderator:** Mmmmhh

**Respondent: So patients are failing to come back for their TCAs or even drugs coz they know that their immunity is low. They can contact COVID 19**

**Moderator:** And…

**Respondent: Other problem is still unavailability of drugs coz for now, clinics, ministry of health are just on PPEs of COVID19 everything, even the other drugs are now missing coz I think the whole money is just trying to buy PPEs, creating beds for COVID19 and they are forgetting that we have other clients like hypertensive who are having chronic illness**

**Moderator: When you say PPE what do you mean?**

**Respondent: Personal protective gears**

**Moderator: Oooh. Thank you. And how has this affected your hours of operation as a clinic?**

**Respondent: Maybe in Kenya, maybe in Kenya. I can quote about Kenya coz of the curfew so you are supposed to get to home earlier and get to work earlier, so if a client comes late and its time you leave you just tell them to come back tomorrow my time is done and I have to get home**

**Moderator:** And again you mentioned something on priorities according to the government like you said the government is concentrating more on buying drugs that are things that will help in controlling COVID19. Could there be other changes in priorities that you can mention

**Respondent: I think. Same, apart from the government buying those PPEs, what to use in the isolation rooms and beds, still at the hospital level, clients come; maybe he is an hypertensive client**

**Moderator:** Mmmmhh

**Respondent: He already knows there are no drugs or he already knows that we just isolating and triaging patient with suspected cases of CIVID19.**

**Moderator:** Mmmmhh

**Respondent: So these clients are missing a lot at the facility level. Everyone is concentrating on clients who are suspected cases rather than those with hypertension**

**Moderator: Mmmmhh**

**Respondent: Of course of late, most facilities maybe even not ours maybe in our sub counties are not doing blood pressure because they say that COVID 19 survives on surfaces and what have you**

**Moderator: Mmmmhh**

**Respondent: so every client that comes in you cannot sanitize the machine every now and then now and then so they are not doing it, so we are not getting even newly diagnosed clients. We are just concentrating on COVID19**

**Moderator:** Then i remember at some point you talked about the outreaches that should be done or the outreaches that you have been doing

**Respondent: Yeah**

**Moderator:** And I would like to know how these outreaches have been affected due to this COVID19

**Respondent: They have really been affected because the COVID19 thin is saying one meter apart, only 15 people. In the community we have thousands of people**

**Moderator: Mmmmhh**

**Respondent: So if we are not doing this then they are not getting the message home or the knowledge. It has really affected, you try just send a message to individual patient. You can’t even educate the other community because you are not supposed to be in gathering.**

**Moderator:** Ok, as we finish, could there be anything you would like to mention in regards to what we have just discussed hypertension

**Respondent: Hypertension**

**Moderator:** Mmmmhh

**Respondent: What I can say in a nut shell is that hypertension is a chronic and non-curable disease but it can be controlled to increase someone’s lifespan and it’s a silent killer**

**Moderator:** Mmmmhh

**Respondent: So with hypertension, those clients with hypertension should be handled with most care and given priority to save most lives. Yeah**

**Moderator:** So with your remarks we appreciate for what you have shared with us and I believe that this will be of great help actually in policy making and coming up with better ways on how the hypertensive care can be done and patients can be handled

**Respondent: Mmmmhh**

**Moderator:** I just appreciate for your time and I believe everything will be of help to us

**Respondent: Thank you**

**Moderator:** Asante Sana .Nice time

**Respondent: Same**
