# Supplementary material for: A socio-ecological framework examination of drivers of blood pressure control among patients with comorbidities and on treatment in two Nairobi slums; a qualitative study
Source: PLOS Glob Public Health. 2023 Mar 10;3(3):e0001625. doi: 10.1371/journal.pgph.0001625 (PMC10021823; doi:10.1371/journal.pgph.0001625)
Supplement: S2 File — (ZIP) [file pgph.0001625.s002.zip › Health Facility/KOCH_KII_HP_200625_002.docx]

**Moderator-{Name}**

**Respondent: Caregiver**

**Code:** KOCH-KII-HP-200625-002

**Moderator**: I’ll take you through the questions for the consent form, you confirm that I have read and you have understood the information for the above study and you have and the opportunity to consider the information, ask questions ad they have been answered satisfactorily

**Respondent:** Ok, I confirm that I have not read but heard

**Moderator:** Yes, sorry, I have read for you

**Respondent:** Yeah

**Moderator:** Yes, you understand that your participation is voluntary and you are free to withdraw at any given time without giving any reasons, without any of your legal rights being affected?

**Respondent:** Yes

**Moderator:** You understand that the data will collected during this study may be looked at by individuals relevant to this study and you give permission to access the information

**Respondent:** Ok

**Moderator:** You confirm consenting to be audio recorded and you also consent to use of anonymized verbatim quotations

**Respondent:** Yeah

**Moderator:** You are happy for your information to be used I future research?

**Respondent:** Yes

**Moderator:** You agree to take part in the above study?

**Respondent**: in he?

**Moderator:** You agree to take part in the above study?

**Respondent:** Ok yes

**Moderator:** Ok, now we shall go to our study, I am going to give you a brief explanation of our study

**Respondent:** Mhhh

**Moderator:** This community has been identified to have a high burden of hypertension which is the leading risk factor to premature deaths and disability. I am trying to gather information about provision of hypertension care in this community particularly for the patients on treatment and who have their blood pressure not under control. I am seeking your views on uncontrolled hypertension among those on treatment in this community and factors driving these high rates.

**Respondent:** Eeeh

**Moderator:** To our questions, we shall begin

**Respondent:** Eeeh

**Moderator:** Please tell me about hypertension care in this community

**Respondent:** Come again

**Moderator:** Tell me about hypertension in this community, the community that you are serving

**Respondent: Ohhh, how I take care of the hypertension problems**

Moderator: Yes

**Respondent: Eeeh…….most be drug based**

Moderator: MMmhh

**Respondent: Common drug that I like to use is called Nifidipine in combination with another one called Lasix**

Moderator: Mmmmhhh

**Respondent: that is normally what I lie to use**

Moderator: Mmhhhh

**Respondent: because they work very well**

Moderator: Ok. Do you have anything to add? About the care

**Respondent: Eeeehh another thing that I usually add is diet**

Moderator: ok

**Respondent: I advise them on good diet and good sleep but what I have come to find is that lack of sleep**

Moderator: Mmmmm

**Respondent: Lack of proper sleep contributes…yeah**

Moderator: Ok. In your facility, please tell me about hypertensive clinics in your facility

**Respondent: we don’t have a particular day for clinic**

Moderator: ok, Mmmhh

**Respondent: They just come randomly**

Moderator: Mmmhh

**Respondent: Yeah**

Moderator: Normally whenever you have your clients coming for the hypertensive clinics probably you said that you don’t have but what is the time that you normally access them

**Respondent: I just told you that it’s random. They just come any time.**

Moderator: Ok. So your facility works 24 hours

**Respondent: Yeah, we work 24 hours**

Moderator: Ok, normally when you get a case of high blood pressure, can you explain how you diagnose them

**Respondent: You know when they come, for example if he is a new patient, they have to go through the reception, pay what we call consultation fee**

Moderator: Mmmhh

**Respondent: Then they go to the next stage which is called…( Not clear)where they are taken their vitals that’s when I realize that the patient has got high blood pressure**

Moderator: Mmhhh

**Respondent: Of course the patient doesn’t know that he has got high pressure. He is………(Not clear)**

Moderator: Mmmmhh

**Respondent: Yeah**

Moderator: Ok. And **in your facility, do you have national guidelines for hypertension that you use**

Respondent: Eeeehhh, not the latest version. Yeah

**Moderator: Which version are you using?**

**Respondent: it’s an old one. A very old one**

Moderator: and can I be able to access it? Do you have it in soft copy or how can I access it?

**Respondent: Eeehhh. No**

Moderator: is it available in the clinic like booklet or something?

**Respondent:**

Moderator:

**Respondent: No no no, I don’t know where…I kind of misplaced**

Moderator: Ok

**Respondent: Yeah**

Moderator: Normally for these patients with hypertension, do they have other conditions?

**Respondent: Yeah, they normally come… in fact the hypertension is not the… I am going to talk about the new clients**

Moderator: Mmmhh

**Respondent: They normally don’t come with complains of hypertension**

Moderator: Mmmhhh

**Respondent: They come with other conditions and that’s when we realize that they have hypertension**

Moderator: Ok, and the ones that have hypertension, do they have other conditions too?

**Respondent: The ones who come or the contin…**

Moderator: Sorry

**Respondent: The ones who come on first visit?**

Moderator: The ones that you have been seeing

**Respondent: Which ones are you talking about?**

Moderator: The ones that you have been seeing, the hypertensive clients. Do they have other conditions apart from hypertension?

**Respondent: Yeah, like most of them yeah**

Moderator: Like which conditions?

**Respondent: The commonest one is urinary truck infection and**

Moderator: Another one?

**Respondent: Maybe….**

Moderator: Ok

**Respondent: Eeeeh**

Moderator: And when they have those conditions, do you have guidelines to help you manage patients with those conditions?

**Respondent: Eeehh**

Moderator: Pardon

**Respondent: Hallo**

Moderator: Hallo, Yes

**Respondent: Eeehh… Ok. What I normally I like is the HIs but I normally use what we have been trained on**

Moderator: Ok. We shall move to another question. What factors are associated with good and poor blood pressure control?

**Respondent: Again what did you?**

Moderator: What factors

**Respondent: What structures**

Moderator: What factors

**Respondent: ooohh factors**

Moderator: are associated with good and poor blood pressure control?

**Respondent: Eehhh.. As I have told you before,**

Moderator: Mmmmhhh

**Respondent: People who like …….(Not clear) Those are the ones who are mostly affected and as you know we are operating in the slum**

Moderator: Pardon, please be louder

**Respondent: I was saying that we operate in the slums**

Moderator: Mmhhh

**Respondent: And in the slums, the condition of living, you can get one roomed house that has got so many people in it**

Moderator: Mmmmm

**Respondent: And they are all sharing**

Moderator: Mmmmhhh

**Respondent: and so it becomes an issue**

Moderator: Mmhhh

**Respondent: and also the …..**

Moderator: Pardon

**Respondent: Hallo**

Moderator: Yes, I can hear you

**Respondent: Am talking about the noise from outside also contribute to luck of sleep**

Moderator: Mmhhhh

**Respondent: In my view lack of sleep is the major course**

Moderator: Ok. Do you have anything to add on the poor controlled?

**Respondent: Sometimes, of course diet is also an important cause**

Moderator: Mmhhh

**Respondent: Diet because some of the foods we eat is poorly cooked because sometimes is cooked in these outside kiosks and you know you can’t control the type of oil they cook with or the amount of oil that they put.**

Moderator: Mmhhhh

**Respondent: a lot of food that has been………… (Not clear)**

Moderator: Ok. And the good factors

**Respondent: what?**

Moderator: Good factors

**Respondent: The good factors is just the opposite of what I said**

Moderator: Mmmhh

**Respondent: Like people who have comfortable beds, maybe they sleep alone, maybe the family is not here is in the up country**

Moderator: Mmhhh

**Respondent: and they cook their own meals, those ones tend to have good control**

Moderator: Ok, anything to add there?

**Respondent: Eehhh**

Moderator: Do you have any more to add?

**Respondent: Eeeehhh, well, the normal stress of life**

Moderator: Mmmmhh

**Respondent: You know there are some people who don’t know to control stress. Yeah**

Moderator: Ok, let us go to another question. What challenges do you encounter in the provision of hypertension care services you provide to your patients with uncontrolled hypertension?

**Respondent: You mean the challenges I encounter?**

Moderator: Yes, while providing services to those clients with uncontrolled hypertension

**Respondent: The most immediate challenge is finance**

Moderator: Yes

**Respondent: Funding. You know …………. (Not clear) conditions they don’t have any form of insurances like NHIF that is also a challenge because they can’t afford**

Moderator: Mmmhhh

**Respondent: I think those are the most important ones**

Moderator: Mmmhh….any other?

**Respondent: Eeeehhh… you see some things like stress become uncontrollable, you know stress you can’t control**

Moderator: Mmmmhhh

**Respondent: Living in slums, luck of money, children are demanding**

Moderator: Mmmh

**Respondent: Yeah, all these**

Moderator: Ok. Do you have any other to add on the challenges?

**Respondent: maybe if I think off.**

Moderator: Do you have, about facility. About facility hours, do you have any challenge about it?

**Respondent: What?**

Moderator: Facility hours

**Respondent: Eeehhh**

Moderator: Facility hours**, Do you have any challenges about it in providing care to these patients?**

Respondent: No no no, here we provide care 24 hours

Moderator: Ok

**Respondent:** Security used to be an issue earlier but nowadays its not a big issue

Moderator: and what about medication

**Respondent: Eeehh**

Moderator: The medications

**Respondent: Medication, they have**

Moderator: Mmmhhh

**Respondent: some don’t have them**

Moderator: and the facilities, the medications for hypertension clients, are they available?

**Respondent: Yes, they are there**

Moderator: ok. And what about the employers work load, as in the care. When we talk about capacity building

**Respondent: Ehhhh**

Moderator: When we talk about workload, or how many employees are you? Is it enough for provision of care?

**Respondent: We are enough for the work load that new get. The only challenge is like now our income has gone down so maintaining the staff is also an issue**

Moderator: When you are prescribing drugs to these clients, coz we are talking about these clients are hypertensive and it’s uncontrolled. Do you face challenge in prescribing medicine to patients with hypertension?

**Respondent: What?**

Moderator: Do you face challenges in prescribing drugs to patients with hypertension?

**Respondent: As I told you earlier, it’s the finance**

Moderator: coz earlier

**Respondent: Finance is a big issue**

Moderator: Mmmmmm

**Respondent: Coz you find this patients are not only having hypertension, they also have other conditions**

Moderator: Mmhhh

**Respondent: so you cannot treat one and leave the other one**

Moderator: When we are talking about medicine prescription, let us see change of prescription like this client was on another drug and he wants to change, do you have some challenges on it with those kind of clients?

**Respondent: for me, funny enough, I have never had a challenge with changing medication**

Moderator: Mmhhh

**Respondent: The only thing that I look out for, is to see whether the have got other conditions that may lead to poor control**

Moderator: Mmmhh

**Respondent: some have other conditions like….. (Not clear) problem**

Moderator: Mmhhhh

**Respondent: So you have to take care of that also**

Moderator: and incase you are going to add medication, does it have a challenge or not?

**Respondent: like what kind of challenge?**

Moderator: now you are going to increase the, assuming the client was taking one tablet then probably you add another one on top, do you normally get challenges on that?

**Respondent: it all come with….(Not clear)**

Moderator: am trying to talk about your perspective as a health care provider. When you are prescribing, what brings challenges in concern with increasing the number of medicine?

**Respondent: I don’t see any challenge**

Moderator: and the strength of the medication?

**Respondent: no, no, no. the strength is not the issue**

Moderator: Mmmhh

**Respondent: the issue is other problems associated**

Moderator: ok **w**hen you talk of other problems associated, is like which one?

**Respondent: I have just told you other conditions like kidney problem**

Moderator: ok then, let me go to another question, what are the factors that contribute to uncontrolled hypertension in the patients you see and I am going to put it in levels. There is individual level, community or family level

**Respondent what are you talking about, try to speak louder please**

Moderator: hello, you can hear me?

**Respondent: Yeah yeah**

Moderator: Mmmmh. Are you. Hell. Can you hear me?

**Respondent: yes I can hear you.**

Moderator: Ok, let me repeat. What are the factors that contribute to uncontrolled hypertension in the patients you see? We shall put them in individual level perspective, community and family level perspective, provider perspective, health system level and policy level perspective

**Respondent: that’s a long long question. You know….**

Moderator: Am just reading through

**Respondent: can you break it down?**

Moderator: That’s what I am trying. Am reading the whole of it then we start breaking

**Respondent: Yeah**

Moderator: so I wanted you to internalize it. What are the factors that lead to uncontrolled hypertension in the patients that you see so we start with individual?

**Respondent: patients that I see?**

Moderator: Let me begin again

**Respondent: are you talking about the hypertensive patients that I see?**

Moderator: Yes, the hypertensive patients that you see who have uncontrolled

**Respondent: Yeah**

Moderator: So we are going to talk about individual factors. What are the factors that lead to uncontrolled hypertension on individual level, now the patient perspective?

**Respondent: I think this is a repeated question coz I have just answered that**

Moderator: Yeah coz at individual level you had answered me and told me about finance

**Respondent: Yeah**

Moderator: is there anything to add?

**Respondent: No, no**

Moderator: Ok. . What are the factors that lead to uncontrolled hypertension on community level, now we are at community and family level? What re the factors that could contribute coz you had talked earlier about the space

**Respondent: at the family level I think it’s the stress. You know if you have many people that stress you, it will affect you also.**

Moderator: ok, do you have any other thing to add?

**Respondent: No**

Moderator: At the provider’s level, the factors that contribute to uncontrolled hypertension? Provider level is like you yourself

**Respondent: Eeeeeee**

Moderator: Hallo

**Respondent: There I don’t see any issue**

Moderator: You don’t see any factors that contribute to it at the provider’s level?

**Respondent: No**

Moderator: Ok: at the health system level perspective, which factor that contribute to uncontrolled hypertension

**Respondent: None**

Moderator: Ok What about the policy level?

**Respondent: The policy**

Moderator: What factors might lead to uncontrolled hypertension in patients that you see

**Respondent: maybe on the policy, you know sometimes I find it a bit a challenge coz the people that formulate policies are not….. You know they formulate for a whole country or a whole region without considering that there are some aspects that are supposed to be…..**

Moderator: Mmmhhh

**Respondent: they do blanket policies. This blanket policies are the ones that brings about…… (Not clear)**

Moderator: mmmhhh

**Respondent: They should vary from one…. (Not clear) to another. They should be individual. Those policies**

Moderator: Ok. To the next question. What in your view would be the possible solutions to the challenges you mentioned

**Respondent: You are talking very slowly**

Moderator: Sorry. What in your view would be the possible solution to the challenges you mentioned. You had mentioned two challenges; finance and stress. So what do you think could be the solution to it?

**Respondent: For finance I don’t like this issue of “serikli saidia’”**

Moderator: Pardon

**Respondent: I really don’t like this statement of serikali saidia but sometimes you have to use it**

Moderator: pardon, please be louder, I can’t hear you

**Respondent: Am saying that there is a statement that I am not comfortable with**

Moderator: Which statement?

**Respondent: It goes like Serikali saidia**

Moderator: Aaahhh! On the finance part

**Respondent: Yeah, that is the statement that I am not comfortable with but sometime you have to use it**

Moderator: Mmhhh

**Respondent: If the government will implement we have like the universal health where by people who don’t have money are catered for**

Moderator: mmmhh

**Respondent: You know that will….(not clear) there is no other solution. you cannot force people to afford what they can not**

Moderator: Yeah

**Respondent: So if the government can chip in the better**

Moderator: ok. What about stress. How can it… what solution can we have

**Respondent: Mmmmhh**

Moderator: What about stress, the challenge you had talked about earlier. What are the solutions?

**Respondent: you know most stress emanates from finances. That’s what I believe**

Moderator: mmmhh

**Respondent: Most of the stress is caused by finance**

Moderator: Ok, do you have anything to add on solutions?

**Respondent: No**

Moderator: Ok this time we are in COVID 19 situation

**Respondent: yeah**

Moderator: yeah. We want to talk about how it has affected your provision of care to hypertensive patients in this community

**Respondent: what I have noticed is that people have got no money**

Moderator: Mmmhhh

**Respondent: that’s what I can add otherwise nothing has affected anything**

Moderator: Ok, has it affected your hours of operation?

**Respondent: no, we open normally**

Moderator: what of availability of hypertension medication?

**Respondent: There is no challenge with that**

Moderator: what about patients not coming

**Respondent: not..**

Moderator: How are patients’ appointments at this time?

**Respondent:**

Moderator: The patients when given appointment, are they keeping the appointments?

**Respondent: Yeah, yeah**

Moderator: ok. What about changing of priority coz of COVID19

**Respondent: Eeehh**

Moderator: Changing of priority

**Respondent: here we tell people to be safe. Both the patient and the health worker. That is the only additional item on the list**

Moderator: Mmmhh

**Respondent: yeah**

Moderator: and at the moment, Outreaches. Are you performing?

**Respondent: No, we don’t do outreaches**

Moderator: is there anything you would want us to talk about that we have not mentioned on COVID19?

**Respondent: The only challenge that we face is that sometime people come without masks**

Moderator: ooohh

**Respondent: and now we are forced to give them our own mask because we cant give them care without mask. Sometime even the masks they wear are of low quality. Others are women out with no laces to tie properly**

Moderator: mmmhh

**Respondent: you are forced to give them yours and of course thi mac with a cost**

Moderator: mmmhh. Ok. Is there anything you would like to talk about in regards to hypertension?

**Respondent: Oooohh. What I have noticed is that. I don’t know if its due to…. For now the cases have gone up a little bit**

Moderator: Mmhhh

**Respondent: I don’t know why the numbers have gone up a little bit. The cases of hypertension have increased that’s why I am saying that I am not sure why**

Moderator: oookk

**Respondent: Yeah**

Moderator: thank you so much for your time and I appreciate the information that you have given me and I believe that it’s going to be useful in the research that we are doing

**Respondent: ok**

Moderator: ok. Thank you so much

**End**
