# Supplementary material for: A socio-ecological framework examination of drivers of blood pressure control among patients with comorbidities and on treatment in two Nairobi slums; a qualitative study
Source: PLOS Glob Public Health. 2023 Mar 10;3(3):e0001625. doi: 10.1371/journal.pgph.0001625 (PMC10021823; doi:10.1371/journal.pgph.0001625)
Supplement: S2 File — (ZIP) [file pgph.0001625.s002.zip › Health Facility/KOCH_KII_HP_200627_0158.docx]

**Moderator: {Name}**

**Respondent: Caregiver**

**Moderator:** Kindly confirm that I have read and that you have understood the information sheet that I have read to you

**Respondent: Yes, yeah, Yeah. Ok**

**Moderator:** You have heard the opportunity to consider the information, ask questions and I have answered you to your satisfactory

**Respondent: Yes**

**Moderator:** You understand that the study is voluntary and you are free to withdraw at any time without giving any reasons and without any of your legal rights being affected

**Respondent: Ok**

**Moderator:** you understand that the data collected during this study may be looked at by individuals where it is relevant to your taking part in the study

**Respondent: Yes**

**Moderator:** You give permission for these individuals to have access to your data

**Respondent: Yes**

**Moderator:** you confirm consenting to be audio recorded and you also consent to the use of anonymized verbatim quotations

**Respondent: Yes**

**Moderator:** You are happy for your data to be used in future research?

**Respondent: Yes**

**Moderator:** You agree to take part in the above study?

**Respondent: Yes**

**Moderator:** So we start. Am going to read to you a few comments before we start

**Respondent: Yeah**

**Moderator:** And then we will start with the questions

**Respondent: Yes**

**Moderator:** So. This community has been identified to have high burden of uncontrolled hypertension

**Respondent: Yes**

**Moderator:** Which is a leading factor to pre mature deaths and disability

**Respondent: Ok**

**Moderator:** So am trying to gather information on the uncontrolled hypertension care in the service particularly among patients on treatment and who have high blood pressure which is not under control.

**Respondent: Ok**

**Moderator:** am seeking your views on hypertension among those on treatment in this community

**Respondent: Yes**

**Moderator:** And factors that are driving to tis high rates

**Respondent: Ok. There are so many factors that lead to, ok that will make a patient not to attend to health care services**

**Moderator:** Mmhhh

**Respondent: One of the factors that I have seen from where am working is the financial strains**

**Moderator:** Mmhh

**Respondent: Most of the hypertensive drugs that we have at the facility**

**Moderator:** Mmhhhh

**Respondent: To the local community they are a bit expensive**

**Moderator:** Mmhhh

**Respondent: So that’s the number one cause, so they feel like they don’t have money to come and buy the drugs**

**Moderator:** Mmhhh

**Respondent: they decide to just either use herbal medicine or just staying and just hope that the blood pressure will lower on its self**

**Moderator:** On your views, kindly tell me about hypertension in your community

**Respondent: Aaahh… about he number or..**

**Moderator:** Generally about hypertensive care in your community, in your facility

**Respondent: As for now there are many programs that are addressing hypertension in the community**

**Moderator:** Mmmhh

**Respondent: That’s in Korogocho and we’ve had some CHVs who give health talks to the community**

**Moderator:** Mmhhh

**Respondent: Who are referring patients to us from the community. They were given some blood pressure machines to be going around taking the blood pressures then they refer those that have high blood pressure to the facility**

**Moderator:** Ok

**Respondent: And that made us to have a high number of patients who are hypertensive**

**Moderator:** Mmhhh

**Respondent: But now the problem is that they were coming for the first time and most of them thought that the program was catering for them for their medicine**

**Moderator:** Ok

**Respondent: So most of them we saw them just once, twice and then they disappeared**

**Moderator:** Ok

**Respondent: So we have many people in the community who have hypertension who are not coming to the facility, so it’s either they are going to other facilities or they are just decided to stay at home**

**Moderator:** Kindly tell me about the hypertensive clinic in your facility

**Respondent: Currently we have just one day for the clinic where we attend to diabetic and hypertensive that is on a Thursday of every week**

**Moderator:** On Thursday from what time to what time?

**Respondent: It starts from 9 to 4 pm**

**Moderator:** 9 am to 4 pm?

**Respondent: Yes**

**Moderator:** So are there any guidelines for hypertension that you use in your facility?

**Respondent: Yes, we have the guidelines. We have the ones on the wall and pocket guidelines**.

**Moderator:** Ok

**Respondent: Yeah**

**Moderator:** Could you send me a copy of that?

**Respondent: Yes, I can**

**Moderator:** Ok, I’ll send you my email so that you can send me a copy of the guidelines

**Respondent: Ok**

**Moderator:** Do you see any patients with hypertension and other conditions?

**Respondent: Yeah. Other conditions like the infectious conditions or lifelong diseases?**

**Moderator:** Whichever, whichever conditions that you see with them

**Respondent: Most of the will come to, ok will attend our facility for other treatment**

**Moderator:** Yeah

**Respondent: And majority of them will not tell you that they have blood pressure until you take the measurement**

**Moderator:** Mmhhh

**Respondent: So they still attend the clinic**

**Moderator:** So which conditions do you see most?

**Respondent: In our facility?**

**Moderator:** Yes. For….

**Respondent: Mostly we**

**Moderator:** The ones who have hypertension and something else

**Respondent: Sorry**

**Moderator:** Am saying. What other conditions do you see your patients with? The ones who come with hypertension. Which is the most condition that you see frequently with hypertension?

**Respondent: With hypertension?**

**Moderator:** Yes

**Respondent: Mostly we get peripheral neuropathies, we’ve had a few cases of stroke and the others just come for the other diseases like skin diseases, typhoid, pneumonia and all that yeah**

**Moderator:** Ok. For this conditions that you see with hypertension, do you have other guidelines that you use for these patients?

**Respondent: Yeah, we have a cardiovascular guideline. I have a soft copy. Actually that’s the one that I will send you.**

**Moderator:** Mmhhh

**Respondent: It covers hypertension plus the complications plus relationship of hypertension and other disease**

**Moderator:** Ok

**Respondent: So it’s a wholesome guideline**

**Moderator:** That’s fine

**Respondent: Yeah**

**Moderator:** So what are the factors that are associated with good and poor blood pressure control?

**Respondent: Eeeehh… the factors ok. Ahh one thing is the, ok most of the factors are pointed towards patients.**

**Moderator:** Eeehh

**Respondent: one is patient’s willingness to take the drugs**

**Moderator:** Mmmh

**Respondent: If they are not taking drugs, they will not lower their blood pressure.**

**Moderator:** Mmmhh

**Respondent: Another one is the patient’s willingness to do the physical exercises and dieting.**

**Moderator:** Mmhhh

**Respondent: Most of them you will tell them about the exercise but they will ignore the exercise part and take the drugs or ignore the drug and exercise part and do diet. Most of them prefer to diet when they have hypertension because they believe that it will control their blood pressure**

**Moderator:** Mmhhh. So you’ve talked about patient’s willingness to take the drugs, diet and exercise. I think that fall into good control

**Respondent: Yeah**

**Moderator:** So what do you think are the factors that would lead to poor control of blood pressure?

**Respondent: Factors that would lead to poor control of blood pressure.**

**Moderator:** Yeah

**Respondent: One is the, what was the name? Compliance to medicine**

**Moderator:** Mmmmhh

**Respondent: Compliance to medicine. Generally is compliance to medicine and compliance to the physical exercises that we tell them to do and the diet.**

**Moderator:** Mmmmhh

**Respondent: if they comply with that then they are able to control their blood pressure**

**Moderator:** Ok. Anything else you would like to add to either good or poor?

**Respondent: Ok, the other thing that might make blood pressure not to, ok the patient not to control their blood pressure is if they are unable to access the hospital.**

**Moderator:** Mmmhh

**Respondent: If they stay far from the hospital and then they would like to come**

**Moderator:** Mmmhh

**Respondent: I have heard others who are coming from other counties like Kiambu County but they have settled for our clinic. Like they want to be coming to our clinic then sometimes they don’t have the transport fee to reach the hospital**

**Moderator:** Ok

**Respondent: Yeah, so that’s a factor that can make them not to come to collect their drugs and lead to uncontrolled blood pressure.**

**Moderator:** Ok. Have you ever found out what problems are there their facilities and the reason as to why they come to your facility for services?

**Respondent: I think is just how we handle our patients. Our clinic holds a few numbers**

**Moderator:** Mmhhh

**Respondent: Ok, we’ve distributed the number equally in the month so we don’t see a large number of patients. So we are able to communicate with our patients and we are able to get feedbacks from them when they come to clinic**

**Moderator:** So roughly let’s say that in a month how many clients do you book for yourself

**Respondent: in a month?**

**Moderator:** I’ll say on average 40

**Respondent: 40 in a month?**

**Moderator:** Yeah

**Respondent: And how do you contact them?**

**Moderator:** We usually have their phone numbers. We have these booklets which we issue to them and they contain their phone numbers and the phone number of the care provider

**Respondent: Ok. Thank you for that**

**Moderator:** So what challenges do you encounter in provision of hypertension care services you provide to your patients with uncontrolled high blood pressure?

**Respondent: Mmmhh. Ok I don’t know if this one applies**

**Moderator:** Mhhhh

**Respondent: There are some, most of the patients who are hypertensive or diabetic, most of them are elderly and sometimes trying to convince an elderly person to take the drugs or do exercises if you are much younger like the way I am**

**Moderator:** Mmmhh

**Respondent: They will pretend to listen but then again not do as you say, yeah. Again talking to such people is a bit of a problem.**

**Moderator:** Mmmhh

**Respondent: Again the other thing is language barrier**

**Moderator:** Mmhhh

**Respondent: You would want to communicate some information to then but then again they do not understand the Kiswahili or English and again you don’t understand their mother tongue or you are unable to communicate with them properly**

**Moderator:** Ok. So the clients that you see with uncontrolled high blood pressure. You have talked about the challenge you encounter as age.

**Respondent: Yes**

**Moderator:** In you clinic, what age do you see who have this uncontrolled high blood pressure?

**Respondent: Mmhh. I’ll say on average 60 years**

**Moderator:** 60 and above?

**Respondent: Yeah**

**Moderator:** What other challenges are you facing with your patients in regards to blood pressure control

**Respondent: The other thing is about; ok we are few in the clinic so most of the time you will find yourself attending to hypertensive clients and you are expected to attend to the other clients the same day**

**Moderator:** Mmmhh

**Respondent: So it’s some kind of being overworked**

**Moderator:** Mmhhh

**Respondent: You are being overworked so you wouldn’t give your best to the patients**

**Moderator:** Ok. Are there any challenges related to medication stock out?

**Respondent: Sorry**

**Moderator:** Are there any challenges related to medication such as stock out in your facility?

**Respondent: Most of the time we don’t have stock outs. It happens maybe once in a while but for hypertensive drugs we usually don’t have**

**Moderator:** Mmhhh. So like at the moment do you have any challenges with medicine stock out?

**Respondent: Currently what I can say about that our facility does not stock all the hypertensive drugs. They only stock the ones that are cheaper to the patients which sometimes is not effective to the patient**

**Moderator:** Mmhhh

**Respondent: You will find that you want to change medicine to you clients maybe from the first line to the second line and the patient has to go buy the second**

**Moderator:** Mmhh

**Respondent: They will be coming to the clinic, you attend to them, you talk to them, you do the blood pressure test to them then they have to go buy the drugs outside.**

**Moderator:** Mmhh

**Respondent: So that’s the other challenge**

**Moderator:** Ok. Do you have other challenges related to facility working hours?

**Respondent: Aaahh, our working hours are favorable because we start work at 8 and work up to 5 and most of the time it’s usually enough**

**Moderator:** Mmhhh

**Respondent: Yeah. Maybe now the problem is the number of clinic days that we have**

**Moderator:** Ok, you said you have how many clinics per month?

**Respondent: We have four, every week we have one clinic**

**Moderator:** Four clinics in a month?

**Respondent: In a month. Yeah**

**Moderator:** Specifically for hypertension and diabetes?

**Respondent: Yeah. And diabetes**

**Moderator:** What challenge do you face when prescribing medication to your patients with hypertension?

**Respondent: The challenges am facing when prescribing drugs?**

**Moderator:** Yeah

**Respondent: One thing I would say really is still the language barrier. I cannot explain to patients the side effects and when to come back**

**Moderator:** Mmmhh

**Respondent: And also for our patients who buy drug outside, you see now I have to do another prescription on top of the one that I had done in their book so that they can buy medication**

**Moderator:** Mmhhh

**Respondent: Most of them if they go to buy the medicine outside you tell them to come so that we can confirm they wouldn’t do that**

**Moderator:** Mmmhhh

**Respondent: So you are not sure if they are taking the right medication you prescribed**

**Moderator:** Ok. Are you having any challenge in changing prescription for the clients that you see?

**Respondent: Changing of drugs or prescription?**

**Moderator:** You see the clients that you are talking about that have uncontrolled high blood pressure

**Respondent: Yes**

**Moderator:** So I am thinking that they were on treatment previously

**Respondent: Yes**

**Moderator:** So do you have any challenges while changing their prescription when they come back?

**Respondent: Yes. Initially the challenge is when changing the prescription for those who have uncontrolled high blood pressure**

**Moderator:** Mmmmmh

**Respondent: You have to move higher from where we are. So if you move higher that is you go to the second level or the third line of treatment and the cost of drugs increases leading to a challenge in high blood pressure control because they will tell you that they can’t afford and that makes them not take the drugs**

**Moderator:** Mmmmhh

**Respondent: Yeah**

**Moderator:** Ok. What are the factors that contribute to uncontrolled hypertension in the patients that you see? There are few levels that we are going to talk about

**Respondent: Yes**

**Moderator:** The first one is from an individual or patients perspectives

**Respondent: Yeah**

**Moderator:** You talked about patient being unable to buy their medicines as a factor like financial constrains and talked about language barrier and you also talked about patients being unable to understand and take your word seriously. Is there anything you would like to add?

**Respondent: Yes. Unavailability of some hypertensive drugs in the facility**

**Moderator:** Yeah. Anything else?

**Respondent: Aaah...on our side or patient’s side?**

**Moderator:** Patient’s perspective

**Respondent: Patient’s perspective?**

**Moderator:** yeah

**Respondent: Aaahh, if I think of another one I will tell you in the course of the interview**

**Moderator:** Its ok, no problem. What of from the individual or family level perspective, what do you think would be the challenge?

**Respondent: At the individual level like I mentioned earlier, we have elderly clients that are hypertensive and those are the kind that need close attention and supervision while taking their medicine. They need someone closer take care of them**

**Moderator:** Mmhhh

**Respondent: We call them the call them care providers**

**Moderator:** Ok

**Respondent: Some of them have care providers at home, some of them don’t have. So that’s a factor.**

**Moderator:** Ok. Anything else you would like to add on the family level? You just mentioned that some of them don’t have care providers

**Respondent: Yeah, they don’t have care providers, which other. I’ll say financial constrains too. If they can’t afford the drugs then they won’t get the drugs**

**Moderator:** Ok. Anything else that you think you have not mentioned on the family perspective?

**Respondent: Mmmhh. Not really. I can’t think of anything**

**Moderator:** Ok. From the provider’s perspective, what do you think are the challenges that are leading to uncontrolled blood pressure?

**Respondent: The ones that take care of them while at home or the health care providers**

**Moderator:** Both, providers’ perspective. It is broad

**Respondent: One thing I’ll say is that on the part of health care providers I’ll say lack of training, lack of skills to attend to the patients. There are many things that are changing in regards to hypertension.**

**Moderator: Mmmhh**

**Respondent: The health care providers are not trained on the current guidelines of management**

**Moderator:** Mmmmhh

**Respondent: They are not able to manage the patients. Then the other thing would be insufficient staffing**

**Moderator:** Mmmhh

**Respondent: like now in our facility am the only one who deals with hypertension and if I am not around or when am on leave them most patients are given longer dates to come for their clinics**

**Moderator:** From the health system level perspectives?

**Respondent: From health system level?**

**Moderator:** Yeah. I’ll say just the unavailability of some hypertension drugs

**Respondent: Yes**

**Moderator:** Anything else from the health system perspective

**Respondent: Mmmhh**

**Moderator:** From the service delivery point, from the leadership, from the governance

**Respondent: Ooooh…you are talking broad. I thought you are only talking about our clinic**

**Moderator:** Those are a few examples am trying to give you so that you are able to tell me from your facility’s perspective

**Respondent: I think of like luck of support from our facility management**

**Moderator:** Mmmhh

**Respondent: We want to get enough support like stocking of drugs at the right time then we will be able to provide services the right way, and our leaders, the local leaders how they can support the hypertensive by organizing for training camps and mobilizing the community, giving health talks to the community through the community health workers**

**Moderator:** Mmmhh

**Respondent: I think that will improve the situation**

**Moderator:** From the policy level perspective meaning the guidelines, the people who give us the guidelines from the national and he state. What do you think are the challenges affecting the policy level perspectives that are leading to uncontrolled hypertension?

**Respondent: The only thing I can say about that is that when they are making their decisions, most of us are usually not involved, not like we need to be involved but come of our leaders like let’s say the clinic officers council, the nurses council they need to be involved so that the information reaches us if there are changes in the management.**

**Moderator:** Ok. We have talked about a lot

**Respondent: Yeah**

**Moderator:** So every level has its challenge and I am going to tell you the few things that you have told me and I would like to get possible solution to each problem

**Respondent: Ok**

**Moderator:** So from the individual and patient’s perspective, you talked about patients having language barrier and unavailability of drugs prescribed to them, you also talked about the patient above the age of 60 that have been predisposed to this

**Respondent: Yeah**

**Moderator:** What do you think are the solutions to individual perspective factors?

**Respondent: Oooohh… Ok. The possible solutions at the individual level**

**Moderator:** Yes

**Respondent: One is that they come, for those that have language barrier should come with their care providers so that we are able to relay the information through their care providers**

**Moderator:** Mmmhh

**Respondent: And then what else?**

**Moderator:** You talked about patients aged above 60

**Respondent: As for those, when we are giving health talks, I can also get a CHV who is elderly maybe to also talk at the same time. With that they will be convinced that someone experienced is also talking about the same thing. I can be doing health talks with a community health worker who is also elderly then that will solve that issue**

**Moderator:** Ok

**Respondent: Yeah**

**Moderator:** From the community and family level perspective you talked about some patients that don’t have health providers to take care of them and you also talked of financial constraints. What do you think are the possible solutions to these?

**Respondent: Aaaahh... Ok I think we can solve that through our CHVs, the health workers. They are the ones that can give the information to the community that if someone is hypertensive then they need a care provider when they go to the facility then those health care providers when they come to explain to them the importance monitoring how their patients are taking medicine**

**Moderator:** Ok

**Respondent: And them to also support the patients with some cash so that they don’t lack money to buy the drugs**

**Moderator:** Ok. From the providers perspective you talked about lack of skills for the providers and also you talked about staffing

**Respondent: Yeah**

**Moderator:** So what do you think would be a solution to that?

**Respondent: There should be more training for the health care workers on the current guidelines and management of hypertension and diabetes**

**Moderator:** Mmmh

**Respondent: Now if the management can add another health care worker who is equipped with knowledge to handle hypertensive clients then this I think will be good**

**Moderator:** Ok. From the health system you still talked about unavailability of medicine and you also talked about lack of support from the management system on your side.

**Respondent: Yeah**

**Moderator:** You also talked about them to organize training camps

**Respondent: Yes**

**Moderator:** I think that’s a solution that you meant for them to organize camps

**Respondent: sure**

**Moderator:** So what do you think can be the possible solutions to unavailability of medicine and lack of support from the management system?

**Respondent: I think on our management part, maybe to lias with some NGO who can be supporting the drugs for the… like for example we used to have an organization called Jack.**

**Moderator:** Ok

**Respondent: They used to subsidies prices of some medicine. Not all but a few were subsidized so the patients were getting them at a cheaper price because were buying them through the organization**

**Moderator:** Mmmhh

**Respondent: So I think if we can have such arrangements for the other drugs then I think the patients will be able to afford the medicine**

**Moderator:** This NGO that you are talking about, which year did it run to?

**Respondent: It ended last year**

**Moderator:** Last year

**Respondent: yeah**

**Moderator:** So now currently you are having your own drugs

**Respondent: Yeah. We are having our own drugs currently**

**Moderator:** Ok

**Respondent: From the policy level you talked about not being involved in making policies, what do you think would be the solution to that?**

**Moderator:** I don’t know what we can do about that, maybe if we can talk to our management.

**Respondent: Yeah**

**Moderator:** They are the people who responsible of reaching the right people so that we can be Involved when they are making those decisions

**Respondent: Yeah so like those seminars we go. Ok when they are making policies**

**Moderator:** Yeah

**Respondent: Our facilities be involved through our management so that we can be able to attend**

**Moderator:** Ok

**Respondent: It’s all about our management being more involved**

**Moderator:** Ok. We are almost done

**Respondent: Ok**

**Moderator:** Everyone is talking about COVID situation and how it has affected the country and the whole nation

**Respondent: Yes**

**Moderator:** How has this affected your provision of services to the hypertensive patients in your community?

**Respondent: What we have done as a facility, the decision we made since March is that those who have controlled blood pressure should be given longer dates so like they be coming to the hospital after two months or after 3 months**

**Moderator:** Mmmhh

**Respondent: And then those that have uncontrolled blood pressure, we are just monitoring them through the phone still they are coming after 2 or three months but now with them we are using the phone to monitor them.**

**Moderator:** Mmmhhh

**Respondent: But you see are not able to interact well and we are not able to know their level of blood pressure at that particular moment. It has changed how we used to attend to the patients but we are trying to the numbers of patients we are having at the facility at a given time**

**Moderator:** Ok

**Respondent: So they are not able to come monthly and we are not able to give one on one talk to them because of the situation. The other thing most of them are elderly are the ones that are more at risk they even fear coming to the hospital coz they fear being infected**

**Moderator:** Mmmhhh

**Respondent: some of them if you tell them to come after three months they still wouldn’t come because of the fear of being infected at the facility and then most of them are not going for blood pressure checks, so if they are buying medication from local shops, were are not able to tell if they are taking their medication as required. So you see it has changed a lot**

**Moderator:** Ok. This clients that you are saying have uncontrolled high blood pressure and you are managing them through the phone. Are you able to contact them or what happens?

**Respondent: I can say we are able to reach most of them like 90% and their care provider numbers. Maybe at times the patients have travelled to up country and the caregivers are around then we are not able to get the right information.**

**Moderator:** How has facility hours of operation been affected?

**Respondent: Initially when the curfew was at 7, we used to close the facility at 4 and when it was changed to 9 then we started operating normally**

**Moderator:** Ok. That’s good. What of the patients not coming for appointments

**Respondent: They are not able because of the fear of contracting the disease at the facility**

**Moderator:** Mmhhh

**Respondent: That’s one, it’s us who has given the longer dated so they don’t come because they don’t have appointments**

**Moderator:** Mmmmh

**Respondent: And those who come, now that the clinics have been pushed, some will just come before their clinic days so they just take their medications and go home because the ones who attends to them is maybe not around**

**Moderator:** What of the change in priorities of the patients that you see?

**Respondent: Change of priorities? In regards to hypertension?**

**Moderator:** Mmmhh. Yeah, in regards to hypertension

**Respondent: for now we are only giving attention to those who have uncontrolled high blood pressure and new clients, those who are still new on medication.**

**Moderator:** Mmhhh

**Respondent: Those are the ones that we prioritize for the clinic**

**Moderator:** Ok. What of the outreaches, are you having outreaches in your community at the moment?

**Respondent: Not really**

**Moderator:** OK

**Respondent: Currently we are unable**

**Moderator:** Ok. Is there anything else that you feel we’ve not talked about concerning COVID and how it has affected your care to hypertensive clients?

**Respondent: I’d say lack of support from the government. As a private institution we’ve not been supported in the right way with the PPEs, right information about COVID so we are not ready to deal with the COVID 19 so that means we are not able to increase the number of patients that we have**

**Moderator:** Ok

**Respondent: Yeah. so am asking if we can get support from the government or NGO like APHRC**

**Moderator:** Ok

**Respondent: If we can get support especially on the PPEs**

**Moderator:** Ok.

**Respondent: Yeah, we don’t have any**

**Moderator:** So in a nut shell, what if you found a COVID 19 patient at your facility, how would you handle that?

**Respondent: A suspected case?**

**Moderator:** Yes

**Respondent:** Ok. The PPEs that we have id the N95 mask. We bought a few because they are expensive

**Moderator:** Yeah

**Respondent: Now if we have a case, usually we are taking the temperature at the gate. So those patients will be suspected at the gate because we’ve trained our watch man to take the temperature and know where the cut off is.**

**Moderator:** Ok

**Respondent: So he is able to call the health care provider who is on duty that day and inform that this patient has high fever. Them from there if am called, the only thing that ill have is my mask**

**Moderator:** Ok

**Respondent: Then I’ll just keep the distance, talk to the patient then call relevant numbers**

**Moderator:** All right. On to the final question

**Respondent: Yeah**

**Moderator:** Is there anything else you would like to talk about in regards to high blood pressure and we haven’t talked about?

**Respondent: None that I can think of now**

**Moderator:** Thanks so much for your time Vincent. I was really productive. I appreciate and the information and I hope it will be of use to the community and patients who live with high blood pressure in the community

**Respondent: Thanks much for your time**

**Moderator:** If there is anything else you would like to talk about I can give you a number that you can call or you can call me later

**Respondent: That’s much better**

**Moderator:** Thank you

**….END….**
