# Supplementary material for: A socio-ecological framework examination of drivers of blood pressure control among patients with comorbidities and on treatment in two Nairobi slums; a qualitative study
Source: PLOS Glob Public Health. 2023 Mar 10;3(3):e0001625. doi: 10.1371/journal.pgph.0001625 (PMC10021823; doi:10.1371/journal.pgph.0001625)
Supplement: S2 File — (ZIP) [file pgph.0001625.s002.zip › Health Facility/KOCH_KII_HP_200627_2209.docx]

**Moderator: {Name}**

**Respondent: Health Caregiver**

**Code: KOCH-KII-HP-200627_2209**

**Moderator:** So… confirm that I have read and you have understood the information that I have read to you. You have had opportunity to consider the information, ask questions and I have these questions answered satisfactorily

**Respondent: Yes**

**Moderator:** Confirm that your participation is voluntary and you are free t withdraw t any time without giving any reason and without your legal rights being affected

**Respondent: Yes**

**Moderator:** You understand that the data collected in this facility may be looked at by individuals where it is relevant to your taking part in this study. You have given permission for these individuals to access your data

**Respondent: Yes**

**Moderator:** You confirm consenting to being audio recorded and you also consent to the use anonymized verbatim quotations?

**Respondent:** **Yes**

**Moderator:** You are happy for your data to be used in future research

**Respondent: Yes**

**Moderator:**  And finally, you are happy to take part in this study

**Respondent: Yes**

**Moderator:** Ok. Now is when we start the interview itself

**Respondent: Alright**

**Moderator:** There is a few information am going to read to you and then we start

**Respondent: Ok**

**Moderator:** In this community, ahh the community has been identified to have high burden of uncontrolled hypertension which is a leading factor to premature deaths and disability

**Respondent: Yes**

**Moderator:** So am trying to gather information about provision of health care services in the community particularly to patients on treatment and who have high blood pressure not under control. So I’ll be seeking your views on uncontrolled hypertension among those on treatment in the community factors that are driving to the high rates of patients having uncontrolled hypertension

**Respondent: Ok**

**Moderator:** On to the first question

**Respondent: Yes**

**Moderator:** Please tell me about hypertension in the community

**Respondent: Aahh. Ok. What would you like to know about hypertension in the community in particular?**

**Moderator:** Everything on hypertension. Anything that you feel important for you to tell me about hypertension in your community

**Respondent: It’s a big burden**

**Moderator:** Yeah

**Respondent: Of late it’s affecting all ages but more prevalent to the age of 30 upwards**

**Moderator: Ok**

**Respondent: Most people don’t know they have it but the majority come with complication**

**Moderator:** Mmhhh

**Respondent: And I would say that it’s a drain to people’s finances especially to those of low social economic levels**

**Moderator:** Mmmhh

**Respondent: Because of drugs and follow ups and investigations**

**Moderator:** Mmhhh

**Respondent: Yes**

**Moderator:** Ok, please tell me about the hypertensive clinics in your facility, now t facility itself

**Respondent: We have hypertensive clinic which runs once every week**

**Moderator:** Mmmhh

**Respondent: That is on Tuesday**

**Moderator:** Mmhhh

**Respondent: We currently have over 400 patients**

**Moderator:** Mmmhhh

**Respondent: We are in the process of starting a support group**

**Moderator:** Ok

**Respondent: Yes**

**Moderator:** These clients that you see every week, how do you divide them to make sure you attend to 400 patients?

**Respondent: What we do,**

**Moderator:** Mmmhh

**Respondent: What we do, we book them as they come**

**Moderator:** Mmmmhh

**Respondent: So there are those who are newly diagnosed, there are those who are already on treatment, there are those whose blood pressure has already been controlled**

**Moderator:** Yeah

**Respondent: And there are those whose blood pressure is uncontrolled**

**Moderator:** Yes

**Respondent: So we give them appointment depending on whether their blood pressure is controlled**

**Moderator:** Mmmhh

**Respondent: Whether they are newly diagnosed. Yes**

**Moderator:** Ok. So are there any national guidelines that you use for your clinic?

**Respondent: Yes**

**Moderator:** Eeehh, do you have it?

**Respondent: In soft copy**

**Moderator:** I can send you an email then you send it to me

**Respondent: I don’t think I can access it today being a weekend**

**Moderator:** No problem, that’s fine

**Respondent: Yes**

**Moderator:** I’ll send you my email then you will find time and send it to me on soft copy

**Respondent: I’ll see whether it’s possible coz we have a system which was developed by Nairobi County the Intel soft**

**Moderator:** Mmhhh

**Respondent: …. (Not clear)**

**Moderator: Mmhhhh**

**Respondent: I am not sure if I can send it from that but that’s where it is**

**Moderator:** Ok. Fine. So do you see patients with hypertension and other conditions?

**Respondent: Yes**

**Moderator:** What other conditions are you able to see?

**Respondent: Arthritis, asthma**

**Moderator:** Mmmhhh

**Respondent: Yes**

**Moderator:** And how do you manage these patients?

**Respondent:** **We manage the conditions they have other than hypertension**

**Moderator:** Mmmhhh

**Respondent: We also manage those other conditions**

**Moderator:** Mmmhh

**Respondent: Yes**

**Moderator:** So do you have other guidelines for these specific patients who have other conditions such as diabetes and asthma that you have mentioned. Do you have other guidelines that you use for them?

**Respondent: Other than, yes diabetes we have**

**Moderator:** Mmmhh

**Respondent: Again in the same system**

**Moderator:** Yeah

**Respondent: And… Yes**

**Moderator:** So, is it the same guideline or it’s a different guideline that you use

**Respondent: It’s a different guideline**

**Moderator:** Ok

**Respondent: For diabetes**

**Moderator:** Alright. So what are the factors associated with good or poor blood pressure control in your view?

**Respondent: In my view good blood pressure control means long life Eeehh less costly to the patient and also less costly to the health systems**

**Moderator:**  Mmmhh

**Respondent: And good quality of life**

**Moderator:** Mmhhh

**Respondent: Poor control means early deaths, complications, high costs to the patients and the systems**

**Moderator:** Mmmhh

**Respondent: And poor quality of life**

**Moderator:** Ok. Any other factors that you would like to add on either of the two

**Respondent: No**

**Moderator:** Ok. What other challenges do you encounter in provision of the health care services that you provide to your patients with uncontrolled hypertension?

**Respondent: The biggest challenge among the many that we have, I think the biggest will be drastic supply of drugs**

**Moderator:** Ok

**Respondent: Or unavailability of drugs throughout which means patients will be off drugs for some time because we don’t have the drugs to give them and they cannot afford to buy**

**Moderator:** Ok

**Respondent: We also have eeehh… we don’t have the capacity and the ability to do routine and follow up in investigations like kidney tests and the patient cannot be able to afford**

**Moderator:** Mmmhh

**Respondent: And where we refer them to do like at the level four they pay**

**Moderator:** Mmmhhh

**Respondent: One they don’t have transport to the facilities and two, if they are not able to find transport then they will not be able to pay for the test**

**Moderator:** Mmmhh

**Respondent: Three, diet**

**Moderator:** Mmmhh

**Respondent: These are people of low social economic status and most of the time they eat what they get now tailoring o feeding to their condition and again feeding their social economic status is a challenge**

**Moderator:** Ok

**Respondent: The other one especially in slums is use of drugs**

**Moderator:** Ok. Drugs such as?

**Respondent: Such as alcohol and cigarettes**

**Moderator:** Mmmhh

**Respondent: The other one is aaaa… I don’t know if I would call it ignorance**

**Moderator: Yeah**

**Respondent: Or refusal to accept**

**Moderator:** Mmmmhhh. They ignore in denial

**Respondent: Yes**

**Moderator:** Ok. What are the challenges you face with your patients in regards to high blood pressure. You have talked about patients income, you have talked about their diet being an issue, them not being able to manage their diet because of high cost of life and you have also talked about use of drugs, some of them use drugs when they are on treatment and you also talked about some of them not being able to have money to go pay for investigations

**Respondent: Yes**

**Moderator:** Do you think that there is anything else that you need to add there?

**Respondent: No**

**Moderator:** Ok. Fine. Are there any challenges related to your facility working hours?

**Respondent: Yes, I would say there is because we work from 8am -5pm and mostly that’s the time when they hustle looking for their livelihood**

**Moderator:** Yes

**Respondent: We don’t work on weekends and public holidays**

**Moderator:** Mmmhh

**Respondent: Yes**

**Moderator:** What’s the time for clinics on every Tuesdays?

**Respondent: Every Tuesday, let’s say from 8-1 but even during those other working hours and days if they walk in they will still be attended to.**

**Moderator:** Ok

**Respondent: Yes**

**Moderator:** You have also talked about that you have a big challenge on supply of drugs.

**Respondent: Yes**

**Moderator:** So when was the last time you were supplied with drugs, your hypertensive treatment drugs?

**Respondent: April**

**Moderator:** April this year?

**Respondent: Yes April this year but we still have stock but not all types**

**Moderator:** So you only have a few? What happens to the other drugs? What do you do?

**Respondent: We prescribe for them to go and buy**

**Moderator:** How about the capacity and the work load of your employees that provides the care. Is that a challenge?

**Respondent: A big one**

**Moderator:** Tell me about it

**Respondent: We are only two clinicians at the facility and I have been alone at the facility as a clinician since November last year but luckily I got a colleague this month**

**Moderator:** Yeah

**Respondent: We also have other than hypertensive clinics we also have other clinics live HIV CCC clinics, TB clinics, general patients work**

**Moderator:** Mmmhhh

**Respondent: So we have a shortage of basically all staffs, we have one nutritionist who is going through all those departments, we have one lab technologist, we have few nurses, we don’t have a pharm tech or a pharmacist, and we don’t have a physiotherapist**

**Moderator:** Mmmhhh

**Respondent: Majority of those patients want a physiotherapist**

**Moderator:** Alright. What are the challenges that you face when prescribing medication to patients with hypertension

**Respondent: Other than the fact that it’s not always that we have the drugs,**

**Moderator: Yeah**

**Respondent: At time you prescribe the drugs and they don’t take the drugs because they don’t have money to buy**

**Moderator:** Mmmhhh

**Respondent: Other time you stick on the guidelines and the same time you look at the status of the patient in terms of their financial ability**

**Moderator:** Ok. And do you have a challenge when increasing the number of medicine or changing prescription to the patients who are already on treatment or probably even when increasing the strength

**Respondent: No**

**Moderator:** Ok. So you are ok with increasing the number of medicine and changing the strength

**Respondent: Yes, we are trained**

**Moderator:** Ok. Fine. What are the factors that contribute to uncontrolled hypertension in the patients that you see? We are going to talk about a few levels starting with the first one which is Individual or patient perspective

**Respondent: Yeah**

**Moderator:** What do you think are the factors that contribute to uncontrolled hypertension on the patient perspective?

**Respondent: Poor adherence to treatment**

**Moderator:** Yeah

**Respondent: Yes**

**Moderator:** Anything else? You had talked about finances earlier

**Respondent: Yes. So finances will lead to poor adherence**

**Moderator:** Yeah

**Respondent: Coz if you can’t afford then you cannot adhere to medication**

**Moderator:** Mmmhhh

**Respondent: We also have life style. Like I said some are alcoholics, others smoke**

**Moderator:** Mmmmhh

**Respondent: Yes are, I wouldn’t say about sedentary lie in the slums**

**Moderator:** Yeah

**Respondent: But I would also say there are those who are not very active**

**Moderator:** Mmmhh

**Respondent: The other one would be diet**

**Moderator:** Yeah. From the individual**,** community family level perspective, what do you think would lead to uncontrolled high blood pressure in this community?

**Respondent:** Maybe poverty and lack of information empowerment

**Moderator:** Anything else?

**Respondent: No**

**Moderator:** Ok. From the provider’s perspective, what do you think would lead to poor blood pressure control?

**Respondent: What I would say is that maybe high workload which would affect the quality of care to client**

**Moderator:** Yeah

**Respondent: When you have a lot of patients you will also not have enough time with each individual**

**Moderator:** Yeah

**Respondent: Yes**

**Moderator:** Ok, anything else?

**Respondent: No**

**Moderator:** From the health systems perspective, what do you think would lead to high blood pressure control?

**Respondent: Erratic supply of drugs and commodities**

**Moderator:** Mmmhh

**Respondent: And also unavailability of routine follow up on investigations**

**Moderator:** Ok. From the policy level perspective?

**Respondent: That one I will pass**

**Moderator:** Ok fine. You’ve talked about a few things from different perspectives, so am going to need the possible solutions. I am going to mention what you’ve told me and you will be able at least to give possible solutions to the challenges you’ve mentioned

**Respondent: Yes**

**Moderator:** For example from the individual level you talked about poor adherence and finances being an issue to the people living in your community and the diet and also most of them not being active like moving around in their day to day life

**Respondent: Yeah**

**Moderator:** So what do you think would be the possible solutions to that?

**Respondent: One is creating awareness, community awareness through maybe media, house to house outreaches and also community health volunteers**

**Moderator:** Ok. From the community level you talked about lack of employment and you also talked about families living in poverty line. What do you think could be possible solution to the challenge?

**Respondent: Maybe supporting them in getting employment**

**Moderator:** Yeah

**Respondent: And maybe small businesses. Yes**

**Moderator: From providers perspective you talked about having a lot of work load and some of you not having enough time with your patients. What do you think would be the possible solution to that?**

**Respondent: One of them would be to increase health care workers**

**Moderator:** Mmmmhh

**Respondent: The other one would be to do capacity building through seminars and trainings and CMEs and OJTs, the other one would be… am not saying that it has not been happening**

**Moderator:** Yeah

**Respondent: Mentorship and support supervision**

**Moderator:** Yeah

**Respondent: Frequent like more frequent than it has been and motivation of staffs**

**Moderator:** Ok, so the policy level you said you will pass and that’s ok

**Respondent: Mmmmhh**

**Moderator:** So in your view

**Respondent: For the facility and health systems, ensure**

**Moderator:** Mmmmhh

**Respondent: That there is availability of drugs, make investigations available and affordable and if possible make them free**

**Moderator:** Ok

**Respondent: Yes**

**Moderator: We are almost done with the interview**

**Respondent: Yes**

**Moderator:** Just a few questions

**Respondent: Ok**

**Moderator:** So everyone is talking about the COVID situation that has affected the whole nation and affected the world

**Respondent: Mmmmhh**

**Moderator:** How has it affected the provision of care to your hypertensive clients?

**Respondent: It has really, first of all these are people with comorbidities so these are people at high risk**

**Moderator:** Ok

**Respondent: Two. First because of the economic meltdown because of COVID, it has affected them in terms of; majority of them cannot even afford food leave alone drugs**

**Moderator:** Yeah

**Respondent: Yes**

**Moderator:** Has it or how has it affected your hours of operation?

**Respondent: It has no affected really because we still operate within our normal hours of operation. Yes**

**Moderator:** And what of the patients how has it affected patients coming for appointments?

**Respondent: They are not coming they used to.**

**Moderator: Mmmmhh**

**Respondent: There is this fear that COVID is a… Hospitals are high risk areas for COVID contraction**

**Moderator:** Mmmmhh

**Respondent: So they are not coming like they used to. Majority of them are missing on appointments**

**Moderator:** You talked about you enrolled; you have over 400 patients booked in the clinic who come every Tuesday. Now since COVID situation started let us say in March when the first COVID 19 case was mentioned in Kenya. How are you seeing the turn out in hypertension patients since March?

**Respondent: It has gone down**

**Moderator:** So like roughly how many do you see in day on a normal day clinic

**Respondent: Infact what happens is we stopped doing clinics on Tuesday alone and we opened up** **to whole 5 days in a week to give them room to come in as they can**

**Moderator:** Mmmmhh

**Respondent: so we are seeing them every day since March**

**Moderator:** Ok. And these clients you are not seeing to the clinic and have been booked for clinic are you able to get them and how do you get them?

**Respondent: We actually don’t have a defaulter tracing mechanism for them**

**Moderator:** Ok

**Respondent: Yes**

**Moderator: You have talked about the current supply of drug being less. How has COVID 19 affected availability of anti-hypertensive medication?**

**Respondent: It has actually not because it is a it was before COVID19. The supplies have always been erratic but since April we’ve as some and we still have stock that would last us for maybe the next one month**

**Moderator:** Ok

**Respondent: Yes**

**Moderator:** Is there anything else that you feel you’ve not talked about the COVID situation that has affected the community?

**Respondent: No**

**Moderator:** on to the last question

**Resondent: Yes**

**Moderator:** Is there anything else you would like to talk about in regards to hypertension we haven’t talked about?

**Respondent: Eeeehhh… I wish it was given as much support as HIV and TB**

**Moderator: Thank you so much for your time and I hope the information that you have given me will be able each the people who will hear it and it will be able to change a few things and to correct and to add to whatever things that have to be added.**

**Moderator: Thank you**

**….END….**
