# Supplementary material for: A socio-ecological framework examination of drivers of blood pressure control among patients with comorbidities and on treatment in two Nairobi slums; a qualitative study
Source: PLOS Glob Public Health. 2023 Mar 10;3(3):e0001625. doi: 10.1371/journal.pgph.0001625 (PMC10021823; doi:10.1371/journal.pgph.0001625)
Supplement: S2 File — (ZIP) [file pgph.0001625.s002.zip › Health Facility/KOCH_KII_HP_200709_0207.docx]

**Moderator: {Name}**

**Respondent: Health Provider**

**Code:** **KOCH_KII_HP_200709_0207**

**Moderator:** Confirm that I have read and you have understood the information in the sheet of the above study and you have had an opportunity to ask questions and you have been answered to your satisfactory

**Respondent: Yes**

**Moderator:** You understand that your participation is voluntary and you are free to leave at any time without giving reasons without your rights being affected

**Respondent: Yes**

**Moderator:** You understand that the data being collected may be looked at by individuals taking part in this study and you allow these individuals to access your data

**Respondent: Yes**

**Moderator:** You confirm consenting to be audio recorded and you also consent using of anonymized verbatim quotations

**Respondent: Yes**

**Moderator:** You are happy for your data to be used in future research

**Respondent: Yes**

**Moderator:** And finally you agree to take part in the study

**Respondent: Ok**

**Moderator:** Am going to read a small statement and then we head right into the questions

**Respondent: Yes**

**Moderator:** This community has been identified to have a high burden of uncontrolled hypertension which is a leading risk to premature death and disability. I am trying to gather information about provision of hypertension care particularly to patients on treatment who have their blood pressure not under control, So I’ll be seeking your views on hypertension among those on treatment and the factors that are driving to these high rates. So in your view, kindly tell me about hypertension in your community

**Respondent: We are just going to talk about hypertension?**

**Moderator:** Yes. We are talking about hypertension among patients who are on treatment and their blood pressure is still not yet controlled

**Respondent: As far as the place where I work, they are seen by a clinician and I dispense the drugs. Most of the clients that we have here are elderly men and women aged between 60 and above and we almost get new cases on daily basis and the other cases we have got a clinic and the file is kept by the clinician there and I think we have approximately 350 to 400 cases. These are both of diabetic and hypertension that have got the file. Those are the ones we give clinics on specific days otherwise now because of this pandemic we are giving them return dates after maybe 3 or four months then we can dispense drugs which are available in our health facility**

**Moderator:** Kindly tell me about the hypertensive clinic in your facility, how it runs on a normal day

**Respondent: We have the outpatient book there whereby every patient who comes in and especially the grownups are taken the blood pressure and after being taken their blood pressure, they are ready to see the clinician. Now the clinician repeats the test and if he see that the blood pressure is still the same as the earlier, then he considers that patient to be a hypertensive case but also it depends with the clinician. There are patients who come with high blood pressure from the first time from the outpatient and they are immediately put on hypertensive drugs and also there other clinicians who give the start doses first and then they advise the patient or the client to be taken blood pressure alternatively for one week and after that if its persistent, they are put on drugs and they are booked for clinic. Others who have controlled or their blood pressure is not very high and also the age matters are put on lighter anti hypertensives like HTZ and then they are given a return date for checkup and when they come up and its showing that it’s not falling down, they are added some other drugs**

**Moderator:** So you said that they start with HTZ and then they are released to go home and when they come back when their blood pressure is not controlled then you have to add them other drugs

**Respondent: Yes**

**Moderator:** Ok. Are there any national guidelines that you are using in your facility for these types of patients?

**Respondent: Yes, there are there**

**Moderator:** Can you kindly send me a copy? I’ll send you an email so that you can send me if you have in soft copy

**Respondent: Ok. I’ll send you. In fact I think I have it in my WPC**

**Moderator:** Ok, I would like to have a copy of that

**Respondent: Ok, I’ll send you**

**Moderator: Ok.** Do you see patients with hypertension and other conditions apart from hypertension itself?

**Respondent: Most of the patients we have they have a combination of hypertension and diabetes**

**Moderator:** Mostly diabetes, in your clinic, what is the other condition that they come with apart from diabetes any other?

**Respondent: Apart from diabetes?**

**Moderator:** Yeah

# Respondent: Because most of them are the elderly people, they come complaining of rheumatism. For example they have got others have got PUK. Peripheral Ulcerative syndrome. They complain of arthritis and Peripheral Ulcerative syndrome

**Moderator:** So these patients that you say have these other conditions apart from hypertension like diabetes, rheumatism, arthritis, do you have other guidelines for these patients’ treatment?

**Respondent: I think we have the guidelines for hypertension and diabetes**

**Moderator:** Ok. What factors do you think in your view are associated with good blood pressure control?

**Respondent: I think one of the biggest challenges that we have that suppresses the control of blood pressure and maybe diabetes is the shortage of drugs. Sometimes we are not supplied with enough drugs, others we are supplied with this type and maybe is a combination 3 drugs, they miss two and get one, Most of the patients here you see are slum dwellers, they are elderly people who are not economically stable**

**Moderator:** Alright. That means that you are trying to say that some of them cannot afford the drugs?

**Respondent: They cannot**

**Moderator**: So those are the factors that are leading to poor blood pressure control, now am trying to find out what are the factors associated with good blood pressure control. What are the good things required for good blood pressure control?

**Respondent: Actually we had tried to form a support group late last year and I had managed to convince I think 44 clients so that we can at least so that we can approach apart from the government, maybe any other NGO who can supply us with drugs. I registered more than 40 clients but later on most of them registered then they disappeared, you ring then, you cannot trace them. So by the beginning of next month I had decided to trace them so that we can try to get assistance from an NGO or a well-wisher so that maybe they can supply us with enough drugs**

**Moderator:** Ok. What are the challenges do you think you encounter in terms of provision of these services with the patients with uncontrolled hypertension

**Respondent: First challenge is that most of them when they are prescribed the drugs, maybe the combination which we don’t have, most of them don’t buy because when they come back and you ask them did you buy these, they always say that they don’t have money. So they just tell me, give me the one that is available. Sometimes I go to the clinician and tell him to change the regime of the drug which are available as long as they don’t have a side effect to that patient because when the government supplies like now the last lot of drugs that we got was on April**

**Moderator:** And do you still have drugs right now or what happened

**Respondent: like last time they supplied with those drugs in diabetes part of it, they never supplied any oral drug. So they just supplied us with insulin which we have, hypertension they tried a bit because they had given us 3 types of anti-hypertensive drugs which we are now happy that when they come they don’t go back empty handed and we still have a little stock remaining**

**Moderator:** So what are the challenges that you are facing with your patients in regards to blood pressure control? You said that some of them are not able to buy the drugs, you also talked about the shortage on your side, the shortage on drugs. What other things do you think channeling to this blood pressure not to be under control

**Respondent: There are some patients especially men who have already been diagnosed and when they come here, maybe it is their first time they come here, some of them don’t know if they are hypertensive you see when you are taking history or maybe when they come, they have already taken alcohol. You know you can immediately know someone who is drunk and smelling alcohol. Then when you try to take history you find that he takes alcohol and maybe cigarettes and every time he comes to attend clinic you see that he is drunk. So you see someone is taking drugs when he is taking alcohol and those other cigarettes, it’s very hard to control blood pressure**

**Moderator:** Ok. Sawasawa, How about capacity of workload on the employees in your care, you have told me that you are a nurse and you still dispense drugs in the pharmacy. How is the workload to employees in your facility?

**Respondent: Ok, now, recently we had only one clinician but at least at the beginning of I think June another clinician was posted here. So we have two clinicians and now they are helping one another. At least now we are not badly off because one can see ordinary outpatient cases especially the time of clinic and the other one see the ones who are due for the clinic**

**Moderator:** So any challenge when prescribing drugs to patients with hypertension?

**Respondent: As far as, me I don’t prescribe but the challenge is that sometimes the clinician might not be around, maybe the combination of antihypertensive drugs, you see somebody maybe have been prescribed 3 drugs like HTZ, nifedipine and maybe enapril, you see these combination you have to decide the combination of ant hypertensive drugs and am not well conversant with that. Like combination of amerodopine, there is amerodopine, enapril, nefidipene, HTZ, all those drugs combination**

**Moderator:** You said that the clinicians are the ones who prescribe these drugs and you have had an encounter with them and when the patients come to you they come with a prescription from the clinician. Have you had an issue with the clinicians having to change prescriptions or increase the strength of these drugs or probably even prescribing the drugs to these patients?

**Respondent: Sometimes when they are prescribed, a patient may come with the prescription, then when am issuing the drug, he or she tell me that when I was given last time I had bad feelings. There are side effects like the nifedipine has side effect of headache. That’s the time I go back to the clinician to explain that the patient is complaining about that particular drug then he can change to another line of the drug which does not have side effects**

**Moderator:** Ok, so what are the factors that, we are going to talk about these on different levels, What are the factors you think they are contributing to uncontrolled hypertension from a patient’s perspective. What would a patient come and tell you that this is what is happening and that’s why I have uncontrolled high blood pressure

**Respondent: Most of them just talk of unavailability of the drugs and the other thing I have already told you it is drunkerdness**

**Moderator:** Ok. And you have also talked about poverty and you also talked about some of them not having enough knowledge on their disease

**Respondent: Yes**

**Moderator:** Now from the community level and family level perspective, what do you think are the factors that contribute to uncontrolled hypertension in these patients?

**Respondent: I think most of the clients whom I see here, I think age factor is contributing. Most of them are women and not men. In our clinic, most clients are old women, obesity is few cases. You see someone is very big. I know like three of them which can be a contributing factor but most of them here I can say it is old age although we have few younger generation especially women who are referred from the FP clinic**

**Moderator:** Ok, FP meaning family planning?

**Respondent: Yes. There are some who are referred from that place and some of them continue to be taking the drugs when they are booked in the clinic**

**Moderator:** Ok, so these patients that you are telling me they are of old age, how are you able to communicate to them about hypertension and their treatment?

**Respondent: It’s a problem explaining to them and especially when you come across somebody whom you are not speaking the same language although some of them are brought by their children but those who come alone, understanding is very hectic. You see an old woman coming at the pharmacy; you explain to her how to take the drugs then after that she goes back to the clinician until the clinicians comes to the pharmacy so that both of you can explain to the old woman or maybe there are some other languages which you don’t understand. You have to look for an interpretor**

**Moderator:** So from the providers perspective, from you yourself as a health care provider, what challenges do you have that you think contribute to uncontrolled hypertension

**Respondent: Me I think the main challenge here is unavailability of drugs**

**Moderator:** Alright. So from the health system, from the level of managerial, from the level of administration of the facility, what do you think are the challenges contributing to uncontrolled hypertension?

**Respondent: That question does not apply the same answer that I have given you unavailability of drugs although ok**

**Moderator:** From the provider I wanted you as the health care provider to explain to me the factors that are contributing to uncontrolled hypertension you as a health care provider then the health system means from the managerial, administration and anything to do with the hospital levels. Level 4, level 3, level 2

**Respondent: Ok, here at the facility we have community health volunteers and most of them are in contact with the clients and sometimes they trace the patients who have been absconders of the clinic. You know here we are at the slums, most of them as I told you I had started a support clinic, I enrolled more than 40 clients; we were meeting every Thursday after two weeks. The first two meetings the attended very nicely then after that because I have a leader who is a community health worker who was mobilizing all the clients, they started coming 4 or 5 when we scheduled a meeting and when you talk to them they are very attentive, they say it’s a very good idea but attendance they don’t come so infarct now am almost despairing**

**Moderator:** Don’t despair, it’s a good thing that you are doing. On the policy level perspective, the people who make these policies that we have and the people who make decisions about the channeling of the information and equipment and everything, so what do you think are the challenges from that level that would cause or make these patients have uncontrolled high blood pressure. What do you think is a problem there?

**Respondent: I think like first quarter we got some drugs like I had told you and now we have ordered some drugs from KEMSA. They told us to order and we made sure that hypertensive and diabetic drugs we ordered in large quantity because most of the drugs that go out of stock after the consignment of the drugs which are supplied they are those drugs because if you see somebody who has hypertension and is taking 300 hypertensive drugs. Maybe is taking one drug two times, you see for one month you are giving 60 tablets and as I had told you earlier we have many cases of hypertensive so they get out of stock within 2 to 3 months. So this time we tried to minimize the other drugs that are not very necessary and I hope they are going to supply us what we had ordered. A very good quantity**

**Moderator:** Ok. We have talked of many challenges that you have had and what do you think are the possible solutions to each problems that you are facing. Now from the individual level you told me poverty, lack of knowledge, alcoholism. What do you think we could do better?

**Respondent: I think what we can do is to lias with the CHVs. I think they are supposed to be more active so that they can go inside the village and we can also enhance daily health talks especially during the clinic time I think if maybe this pandemic will get over because now we are giving them appointments of 3 to 4 months and we issue them with the drugs that we have because of the congestion. Our health center is very small and there always congestions during normal times**

**Moderator:** Ok. From the community level you talked about some of them being elderly and some of them not being able to come for the clinics because of their age and you also talked about some of them being overage

**Respondent: Language barrier**

**Moderator:** What do you think we could do better for these challenges to go down?

**Respondent: We have been trying very hard for**

**Moderator:** What do you think we could do differently to have solution to challenges like language barrier and some of them being brought by their children, some of them being obese you have mentioned that previously, what do you think we could do?

**Respondent: I think we can now take advantage of CHVs because we have several of them although they try very hard**

**Moderator:** Ok. From providers perspective you’ve said defaulter tracing has been an issues and you also mentioned that you had a program that had started and it became an issue since you were not able to reach some of the people you had enrolled. So what do you think we could do differently in tracing their clients?

**Respondent: We have contact numbers of most of our patients in our files, I think we can restart and try to organize ourselves. We can try maybe during clinic time talk to them and tell them that we are planning something for them then we can call a meeting and then we can give them extensive health education**

**Moderator:** From the health system and policy level I think you talked about one thing that the system not being able to supply enough drugs for your facility and some of them having specific type of drugs and some not being available for a very long time. So what do you think we could do differently for this problem?

**Respondent: I think maybe if we can have a well-wisher or a donor who can supply us with drugs that cannot be supplied by KEMSA and they are prescribed here. I think we can feel very good**

**Moderator:** Ok. How has this COVID situation affected you hypertensive patients in this community?

**Respondent: Especially now we are not conducting the clinic day and whenever any patient visits us he or she is put at list along TCAs to come back**

**Moderator:** Sorry, you are saying

**Respondent: When somebody finishes the drugs and come because we do not have a specific clinic day now, they are put to a TCA to come maybe after 3months or 4 and we make sure with the drugs that we have them here, we give them drugs for at least 3 months and then we tell them to come and collect when they get finished but when they come they are taken the BP and they are done sugar**

**Moderator**: And what about the hours of operation in your facility, how has COVID affected that?

**Respondent: Before the COVID situation we used to have so many outpatients in fact the clinician used to see more that 80 outpatients now they are coming at around between 50 and 60 and the hypertension cases have been isolated now you see maybe 5 but those come according to the availability of drugs. If they have finished the drug they come and collect the drug**

**Moderator:** Ok. Are you having challenge with patients not coming for clinic appointments?

**Respondent: Initially they were coming in a good number but as I told you they are not being given the return date. We are giving them the drugs then we tell them to come when they finish the drugs we don’t want to give them a clinic day because if we give them a clinic day they will come and congest here. So far we have not gotten any case where by the blood pressure has raised abnormally; I think they are faring well**

**Moderator:** Ok. Have you had any outreaches in this current situation?

**Respondent: We have not had any outreaches for the NCDs but other outreaches that I have seen here are family planning and CWC**

**Moderator:** Is there something else that has been affected with COVID and we have not mentioned?

**Respondent: So far I will say that we have not witnessed any case although we had two cases that were referred to Mbagathi I think it was last week and the other sometimes back.**

**Moderator:** What was the issue?

**Respondent: The one that was referred earlier I think was negative and the one who was referred to Mbagathi last week she was a lady and I don’t know her fate. Otherwise we are being supplied with the basic commodities like the PPEs; Masks, sanitizers, and other few PPE gadgets that we can manage ourselves if we get a very ready case presented with symptoms of CIVID**

**Moderator:** So we can gladly say that you are prepared for every COVID situation that would come to your facility

**Respondent: Yes, we are prepared coz every case we suspect we have got numbers whereby we are supposed to ring to the sub county like the one am telling you for last week we just rang the ambulance and it came and took the patient to … so if we get a case we really depend on the sub county and when they come they just pick the patient**

**Moderator: We are on the last question, is there anything else about hypertension that you feel we have not talked about and you feel that we should talk about it?**

**Respondent: What we have not mentioned I thing is the complications of hypertension whereby a patient can come presenting with very alarming high blood pressure maybe a case of referral. Someone can come with a very elevated pressure. Sometimes somebody is just walking and he is not showing ant symptoms. A patient like that is supposed maybe to be even admitted in the ward. Sometimes maybe the ambulance, me I see the ambulance maybe sometimes take a lot of effectiveness when there is maybe an emergency for example maybe the maternity they should come very fast. Someone can come just walking and you refer to Mama Lucy. You tell them that this blood pressure of yours is very high and maybe you need to be admitted in a ward. You refer the patient and maybe because the patient is not feeling anything they might not go there. He or she says that she is not feeling anything. System of referral I think that one is a challenge**

**Moderator:** Ok. Anything else?

**Respondent: Maybe somebody is brought by the relative then you tell them that the BP is very high and they should go to Mama Lucy then the relative start talking about the ambulance but you see we only get a good response from the ambulance for maternity cases**

**Moderator:** Ok, so you are trying to say that for the maternity cases the ambulance is as quick as possible but for patients with hypertension is not as

**Respondent: When we have a patient with high blood pressure, they are given start doses and kept here for like 3 hours and then after that the patient is referred though we don’t call ambulance for those cases. Let me be clearer**

**Moderator:** Ok. Thank you for your time and I appreciate the information that you have given me and I hope that it will reach the ears that should hear and some changes and some of the information you have told me will be able to make a difference. Thank you very much for your time

**Respondent: Ok**

**…END…**
