# Supplementary material for: A socio-ecological framework examination of drivers of blood pressure control among patients with comorbidities and on treatment in two Nairobi slums; a qualitative study
Source: PLOS Glob Public Health. 2023 Mar 10;3(3):e0001625. doi: 10.1371/journal.pgph.0001625 (PMC10021823; doi:10.1371/journal.pgph.0001625)
Supplement: S2 File — (ZIP) [file pgph.0001625.s002.zip › Health Facility/VIWA_KII_HP_200626_001.docx]

**Moderator: {Name}**

**Respondent:**

**Code: VIWA-KII-HP-200626-001**

**Moderator:** Ill begin with the certificate of consent, I am going to read to you and you confirm yes or no

**Respondent: Yeah**

**Moderator:** I confirm that I have read and you have understood the sheet and you’ve had the opportunity to consider information, ask questions and your questions have been answered satisfactory

**Respondent: Yes**

**Moderator:** You understand that your participation is voluntary and that you are free to withdraw at any time without giving any reasons without any of your rights being affected?

**Respondent: Of course yes**

**Moderator:** You understand that the data collected during this study maybe looked at by individuals where it is relevant to your taking part in this study. You give permission for these individuals to have access to your data

**Respondent: It is yes**

**Moderator:** You understand that the data collected during this study maybe looked at by individuals where it is relevant to your taking part in this study. You give permission for these individuals to have access to your data

**Respondent: Yes**

**Moderator:** You confirm consenting to be audio recorded and you also consent to the use of anonymized verbatim quotations

**Respondent: Yes**

**Moderator:** You are happy to your information to be used in future

**Respondent: Yes**

**Moderator:** You agree to take part in this study

**Respondent: Yes**

**Moderator:** Ok, we shall begin. Am going to give you a brief explanation also for the study. This community has been identified o have high burden of uncontrolled hypertension which I th leading risk factor to pre mature deaths and disability. I am trying to gather information about provision of hypertension care in this community particularly for patients on treatment and who have their blood pressure not under control. I am seeking your views on controlled hypertension among those on treatment in this community and factors driving to the high rates I am going to begin my questions

**Respondent: Yeah**

**Moderator:** Please tell me about hypertension care in this community

**Respondent: As per the facility or as per the community?**

**Moderator:** Per the community

**Respondent: Per the community the hypertension is negligence**

**Moderator:** Mmmmhh

**Respondent: Because they rarely understand or they rarely know, Most of them rarely know that they are hypertensive unless they present themselves to the health care**

**Moderator:** Mmmmhh

**Respondent: Unless the present themselves to the health care or the hospitals around the community whereby they are diagnosed with hypertensive but before that they rarely understand they are hypertensive unless there is a family backtrack**

**Moderator:** Mmmmhh

**Respondent: Yes**

**Moderator:** What about the ones who are already diagnosed, how is their care

**Respondent: The ones that are diagnosed we put them on… those that are diagnosed understand their mediation as per the time**

**Moderator:** Mmmmhh

**Respondent: And there are cases where we have to change the medication because there are some that don’t respond to their hypertensive status**

**Moderator:** Mmmmhh

**Respondent: So we have to keep on changing and keep on monitoring as per the cases**

**Moderator:** Mmmmhh

**Respondent: For instance in my facility we have records, whereby we give them either cards whereby we usually monitor their BP readings**

**Moderator:** Mmmmhh

**Respondent: On daily basis or weekly basis**

**Moderator:** Ok

**Respondent: And in case we find out that they respond well**

**Moderator:** Mmmmhh

**Respondent: We either reduce the medicine or we withdraw. But the cases whereby they rarely respond, we have to look it further**

**Moderator:** Mmmmhh

**Respondent: Either through more examination**

**Moderator:** Mmmmhh

**Respondent: Yeah**

**Moderator:** Ok, in your facility, can you tell me about the hypertensive clinic

**Respondent: We have… most of the time we rarely have hypertensive clinics but the clinics we usually do them at ANC levels because most of the cases come with ANCs**

**Moderator:** Mmmmhh

**Respondent: And incase there is that factor that has been long,**

**Moderator: Mmmmhh**

**Respondent: Is when we give clinic as per the weekly or monthly basis**

**Moderator:** Ok**.** When you say ANC level, what do you mean?

**Respondent: They are mothers who are pregnant**

**Moderator:** Mmmmhh

**Respondent: And in the pregnancies they come and we diagnose that they are hypertensive in pregnancy**

**Moderator:** Mmmmhh

**Respondent: Now when we find out that there is hypertensive status in pregnancy, we have to undertake the measure that are relevant so that they don’t end up with either pre mature , giving birth pre maturely of they don’t eeehh. The foetus is not affected either directly or indirectly**

**Moderator:**  Ok

**Respondent: Yeah**

**Moderator:** In your case you say that you don’t have a clinic day but you

**Respondent: Yes, we don’t have ANC clinic day sorry hypertensive clinic day**

**Moderator:** Mmmmhh

**Respondent: But whenever we recognize one**

**Moderator:** Mmmmhh

**Respondent: Or some cases**

**Moderator:** Mmmmhh

**Respondent: We usually do impromptu clinics**

**Moderator:** Mmmmhh

**Respondent: Whereby we look at especially two to three cases and they come for a CME concerning management**

**Moderator:** Ok

**Respondent: Yeah**

**Moderator:** And when you get such a client, how do you diagnose the high blood pressure

**Respondent: Eeeeehh, diagnosis we usually use the BP readings**

**Moderator:** Mmmmhh

**Respondent: And we don’t undertake only one reading**

**Moderator:** Mmmmhh

**Respondent: We have to undertake several reading for instance if it’s so high,**

**Moderator:** Mmmmhh

**Respondent: We have to take first reading; we give like 2 to three aaahhh 20 to 30 minutes**

**Moderator:** Mmmmhh

**Respondent: We repeat to know whether its faulty or ok**

**Moderator:** Mmmmhh

**Respondent: and in case we find out that the readings are still high**

**Moderator:** Mmmmhh

**Respondent: We have to undertake the third reading**

**Moderator:** Ok

**Respondent: And after the third reading is when we ask for the history if the patient has ever had a high….if the patient has been hypertensive**

**Moderator:** Mmmmhh

**Respondent: Or he or she doesn’t know. There after we have to tell her that since we see the case look more like hypertensive,**

**Moderator:** Mmmmhh

**Respondent: We have to be checking on and off. Every now and then**

**Moderator:** Ok

**Respondent: Yeah**

**Moderator:** Ok. And in your facility, do you have, when you are managing these patients, do you have a national guidelines that you use?

**Respondent: Yeah, we have a national guideline**

**Moderator:** Mmmmhh

**Respondent: We have a national guideline that we use**

**Moderator:** Mmmmhh

**Respondent: Yes**

**Moderator:** is it available? Can I have a soft copy?

**Respondent: A photo copy unless**

**Moderator:** Or a soft copy

**Respondent: Unless I were to go and look out**

**Moderator:** Mmmmhh

**Respondent: There is one**

**Moderator:** Ok Then I think I will follow it later

**Respondent: Yeah**

**Moderator:** when you are having these clients, the hypertensive clients, do they have other conditions?

**Respondent: There are cases that we find out that some are both hypertensive and diabetic**

**Moderator:** Mmmmhh

**Respondent: And others as I had said earlier**

**Moderator:** Mmmmhh

**Respondent: In our settings, many rarely understand that they have hypertensive conditions**

**Moderator:** Mmmmhh

**Respondent: Now is only us who understand them**

**Moderator:** Mmmmhh

**Respondent: And by monitoring them time again**

**Moderator:** Mmmmhh

**Respondent: is when we come up with conclusion that they have other conditions or other underlying conditions**

**Moderator:** Mmmmhh

**Respondent: Yeah**

**Moderator:** Apart from diabetes, any other condition?

**Respondent: Eeehhh, I have seen one. There was one. There are some that have are seropositive.**

**Moderator:** What do you mean by are seropositive?

**Respondent: HIV positive**

**Moderator:** Ok

**Respondent: And in the course of being HIV positive, they are hypertensive**

**Moderator:** Mmmmhh

**Respondent: There are others that present in similar conditions, some have PTB**

**Moderator:** What do you mean by that? Can you expound?

**Respondent: Pulmonary tuberculosis**

**Moderator:** Ok

**Respondent: There are others that have either sarcoma or cancer**

**Moderator:** Mmmmhh

**Respondent: And very many other factors**

**Moderator:** Ok. And how do you manage this kind of patients?

**Respondent: Eeeehh. We usually look at the records because most of the time when you f9ind one who is seropositive or HIV positive**

**Moderator:** Mmmmhh

**Respondent: They are on other medication**

**Moderator:** Mmmmhh

**Respondent: When they are under other medication, we have to look at drugs that will not interact with other medicine or that don’t … we have to look for drugs that if at all they are reactive or sensitive**

**Moderator:** Ok

**Respondent: Or we look at if they are using other drugs at what drugs are they using and if they will react with this other medicine**

**Moderator:** Ok.

**Respondent: Yes**

**Moderator:** And these patients who have other conditions that we have talked about, diabetes, HIV or PTB or cancers, do you have guidelines for them?

**Respondent: Automatically, HIV there are guidelines**

**Moderator:** Mmmmhh

**Respondent: With diabetic we’ve got guidelines**

**Moderator:** Mmmmhh

**Respondent: But when we talk about cancer**

**Moderator:** Mmmmhh

**Respondent: It’s a secondary factor or a tertiary factor whereby we have seek further medical advice from other higher institutions**

**Moderator:** Ok. Let’s go to another question

**Respondent: Yeah**

**Moderator:** What factors are associated with good and poor blood pressure control?

**Respondent: Factors?**

**Moderator:** The factors that affect good and poor

**Respondent: Blood pressure control**

**Moderator: Yes.**

**Respondent: We usually look at the salt intake**

**Moderator: Mmmmhh**

**Respondent:** **Body weight**

**Moderator:** Mmmmhh

**Respondent: And cholesterol levels, because when one has a higher cholesterol level,**

**Moderator:** Mmmmhh

**Respondent: The possibility of having hypertension status is high**

**Moderator:** Mmmmhh

**Respondent: When there is a higher intake**

**Moderator:** Mmmmhh

**Respondent: It affects the hypertensive status**

**Moderator:** Mmmmhh

**Respondent: And when one is overweight or obese, automatically it affects the pressure**

**Moderator:** Mmmmhh

**Respondent: So we have to look at the guidelines on how to advice as per the BMI**

**Moderator:** Mmmmhh

**Respondent: And how they are to take care of their pressure and whatever**

**Moderator:** Ok

**Respondent: Yeah**

**Moderator**: Do you have more to add on the poor blood pressure control?

**Respondent: poor control is just negligence**

**Moderator:** Mmmmhh

**Respondent: Many do come and when you test them you tell them you know you are hypertensive, they never take it into consideration**

**Moderator:** Mmmmhh

**Respondent: Whenever you book them for a clinic, they rarely come**

**Moderator:** Mmmmhh

**Respondent: Or whenever you tell them why don’t we be monitoring on timely basis**

**Moderator:** Mmmmhh

**Respondent: They take it as a, they just ignore all together. That is a poor way of managing how we find it very difficult to manage them**

**Moderator:** Mmmmhh

**Respondent**: **Because many tell you that, I have never had such a case or they, anyway there is just ignorance and negligence**

**Moderator:** Mmmmhh

**Respondent: Its one of the poor ways that make them very hard to manage their hypertension**

**Moderator:** Mmmmhh

**Respondent: Yeah**

**Moderator:** And can you give me some good factors of blood pressure control?

**Respondent: Good factors of blood pressure control?**

**Moderator:** Mmmmhh

**Respondent: Eeehh, one is medication. Hallo**

**Moderator:** Yes

**Respondent: Am I still in the**

**Moderator:** Blood pressure control

**Respondent: The pressure control most of the time they usually take medication**

**Moderator:** Mmmmhh

**Respondent: We have to ensure that eeehh; they take low salt in their diet**

**Moderator:** Mmmmhh

**Respondent: We have to ensure that there is a little fat in their food so that the cholesterol levels don’t go up**

**Moderator:** Mmmmhh

**Respondent: And exercising time and again**

**Moderator:** Mmmmhh

**Respondent: Yeah**

**Moderator:** Do you have any to add?

**Respondent: Eeeehh, anything to add?**

**Moderator:** Yes

**Respondent: We look at these other factors for instance when one is taking, whoever is HIV positive**

**Moderator:** Mmmmhh

**Respondent: They have to take their medicine on time**

**Moderator:** Mmmmhh

**Respondent: Their medicine on time and we have to ensure that they don’t, there is how do I put? There is this education, more education on timely basis because lack of education concerning their status automatically will affect the hyper, the BM**

**Moderator:** Ok

**Respondent: Yeah**

**Moderator:** Ok, let’s go to another question. What challenges do you encounter in provision of the hypertension services that you provide to your patients with uncontrolled hypertension

**Respondent: Repeat**

**Moderator:** What challenges do you encounter in provision of the hypertension services that you provide to your patients with uncontrolled hypertension?

**Respondent: The challenge that we usually get is poor response to medication**

**Moderator:** Mmmmhh

**Respondent: And there are cases whereby we have to undertake cholesterol levels tests**

**Moderator:** Mmmmhh

**Respondent: And you understand that the tests are expensive as per the community**

**Moderator:** Mmmmhh

**Respondent: And since they are unable to provide them and we are unable to meet their whatever**

**Moderator:** Mmmmhh

**Respondent: You will find out that the cholesterol levels we won’t get it in in time**

**Moderator:** Mmmmhh

**Respondent: So that will affect because whenever you send one to take either LFTs cholesterol or whatever, they rarely bring back the results**

**Moderator:** Mmmmhh

**Respondent: There are cases where we have to understand what is the course especially echo. There are cases whereby we have to take echo tests or echo examination. Eeehh. When you send them for the echo they rarely come back**

**Moderator:** Mmmmhh

**Respondent: Those are some of the challenges we usually meet especially with the exorbitant prices on the test**

**Moderator:** Mmmmhh

**Respondent: Yeah**

**Moderator:** Any more you can add?

**Respondent: Eeeehh, I had talked of medicine**

**Moderator:** Mmmmhh

**Respondent: I have talked of…**

**Moderator:** Cholesterol levels

**Respondent: Higher prices in the tests**

**Moderator:** Mmmmhh

**Respondent: And I think the financial background of the families**

**Moderator:** Mmmmhh

**Respondent: Eeehh, And many don’t believe that, rarely believe that hypertensive is treatable**

**Moderator:** Mmmmhh

**Respondent: Yeah**

**Moderator:** Ok. And when we talk about your facility hours, does it have any challenge to you?

**Respondent: Facility**

**Moderator:** Hours

**Respondent: Hours**

**Moderator:** Mmmmhh

**Respondent: Eeeehh, automatically at facility level we must have challenges because**

**Moderator:** Mmmmhh

**Respondent: Maybe today I may have a clinic at the ANC clinic or a maternity case where by a mother can develop hypertension when she is expecting or she is giving birth**

**Moderator:** Mmmmhh

**Respondent: Automatically we will have challenges because we have to, it depends the mother has presented in what stage**

**Moderator: Mmmmhh**

**Respondent: When the mother comes in second stage and she is hypertensive, automatically you will find out that we have as per the facility we have to take emergency measures whereby we may affect the other clinics or departments**

**Moderator: Mmmmhh**

**Respondent: Because we have to look into it that the mother delivers well and the mother is stable and as per the staffing you will find out that it will affect other provisions**

**Moderator: Ok**

**Respondent: Yeah**

**Moderator: And earlier you had talked about second stage, can you elaborate more**

**Respondent: Second stage is when a mother comes fully dilated, she comes directly from home when she is fully dilated.**

**Moderator:** Mmmmhh

**Respondent: When we cannot refer, you cannot refer,**

**Moderator:** Mmmmhh

**Respondent: You have to undertake the situation on the ground**

**Moderator:** Mmmmhh

**Respondent: You have to take all the resources that you have**

**Moderator:** Mmmmhh

**Respondent: To manage her**

**Moderator:** Mmmmhh

**Respondent: So that you don’t lose the mother and at the same time the child**

**Moderator:** Mmmmhh

**Respondent: Or the mother is rarely affected directly or indirectly through the stage**

**Moderator: Mmmmhh**

**Respondent: When we talk of second stage as a difficult stage**

**Moderator: Mmmmhh**

**Respondent: Is whereby you don’t refer unless otherwise. Unless you have an ambulance around or any other factors**

**Moderator: Mmmmhh**

**Respondent: Yeah**

**Moderator: What about medication for hypertensive**

**Respondent: medication in hypertension we are always ready**

**Moderator: You don’t have any challenge on it**

**Respondent: Yeah. There are a few challenges because you will find out that maybe you didn’t expect such a situation**

**Moderator: Mmmmhh**

**Respondent: And as per the emergency state you find out that maybe you had replenished a certain drug**

**Moderator: Mmmmhh**

**Respondent: Without eeee, any way there are cases where we are always ready but we find out that maybe for instance magnesium sulphate you don’t have it**

**Moderator: Mmmmhh**

**Respondent: And you expected to have it**

**Moderator: Mmmmhh**

**Respondent: So now you have to look into it that the mother doesn’t do into Eclampsia or Pre Eclampsia**

**Moderator: Ok**

**Respondent: Yeah**

**Moderator:** You had talked earlier about workload to the employees, you said that there is a time you have to take them to. For instance you had talked about a mother who had come to and you have to work on him, on her. So do you have challenges to deal with work load on employees?

**Respondent: The challenges are always there,**

**Moderator:** Mmmmhh

**Respondent: Workload of employees depend on day to day routine**

**Moderator:** Mmmmhh

**Respondent: Because there are cases where you will find out that maybe you have three nurses or two nurses on duty and one or two clinical officers and one pharmacist**

**Moderator:** Mmmmhh

**Respondent: Maybe you know the procedure has to be that whenever you have to refer any patient**

**Moderator:** Mmmmhh

**Respondent: Hallo**

**Moderator:** Hallo, yes, I can hear you

**Respondent: Whenever we have to refer any patient, the patient has to be accompanied by a nurse**

**Moderator:** Mmmmhh

**Respondent: Now you find out that maybe there are emergencies that will come on when the nurse on duty has gone for a referral or has taken a patient for a referral**

**Moderator:** Mmmmhh

**Respondent: Now you have to undertake both the nursing cases and the clinical cases on your own**

**Moderator:** Mmmmhh

**Respondent: Now in that instance, incase another case occurs along the way, automatically you will have challenges**

**Moderator:** Ok. We are going to talk about prescription of medication

**Respondent: What? Prescription**

**Moderator:** Yes, let me tell you

**Respondent: Yes**

**Moderator:** Challenges you face when prescribing medication to patients with hypertension based on changing prescription .Do you have challenges with that?

**Respondent: Eeeehh. With prescription, we don’t have a challenge with that**

**Moderator:** Mmmmhh

**Respondent: We have guidelines unless once in a while**

**Moderator:** Mmmmhh

**Respondent: When you give prescription as per the guideline and the response is poor**

**Moderator:** Ooohh

**Respondent: That’s when you have to look for challenges to see maybe what’s maybe countering the**

**Moderator:** Mmmmhh

**Respondent: Or there are other patients who however much they have been on such a drug, maybe the patient is not your patient**

**Moderator:** Mmmmhh

**Respondent: The patient has been in such a hospital or a different hospital where by the have never changed the medication for him or her**

**Moderator:** Mmmmhh

**Respondent: You will find it very hard to confirm to him or her that you have to change this medication because the response is poor**

**Moderator:** Mmmmhh. And what about increasing the number of **medication**

**Respondent: Increasing the number of medication?**

**Moderator:** Mmmmhh

**Respondent: We usually increase provided they don’t counteract**

**Moderator:** Ok

**Respondent: There are cases whereby in hypertensive status you have to give more than two or three medications at the same time**

**Moderator:** Mmmmhh

**Respondent: Instead of one**

**Moderator:** Mmmmhh. The patients have no challenges on it. They are ok

**Respondent: The ones that I have been handling never have any challenges**

**Moderator:** Ok

**Respondent: Except only one case or two cases where your patient say that there are certain medicine that give me a severer headache**

**Moderator:** Mmmmhh

**Respondent: Now you have to look at it to understand why is it causing headache**

**Moderator:** Ok

**Respondent: Yeah**

**Moderator:** And what about when increasing the strength?

**Respondent: The strength of the medicine?**

**Moderator:** Mmmmhh

**Respondent: Eeeehh, we usually increase, for instance this patient has BP of more than 190 and he or she is on Nifidipine**

**Moderator:** Mmmmhh

**Respondent: That is 40mg OD; you have to increase it to BD instead of OD**

**Moderator:** Mmmmhh

**Respondent: Yeah**

**Moderator:** When you talk about **OD and BD, can you elaborate**

**Respondent: OD means you give one in a day, BD means you give twice in a day**

**Moderator: Ok**

**Respondent: TID means we give thrice in a day**

**Moderator: Ok**

**Respondent: and FID four times**

**Moderator: Ok**

**Respondent: So we usually look at if at all incase instead of giving one a day, there are cases where you find that, there are patients where you find that in the morning**

**Moderator: Mmmmhh**

**Respondent: Their blood pressure is ok**

**Moderator: Mmmmhh**

**Respondent: And they rise in the evening**

**Moderator: Mmmmhh**

**Respondent: And others you find that in the evening their pressure BP is ok and they rise up early in the morning**

**Moderator: Mmmmhh**

**Respondent: Now you have to look at what are the factors that are making it rise**

**Moderator: Eeeehh**

**Respondent: So you have to ensure that if it is in the morning you have medication to cover the morning BPs**

**Moderator: Ok**

**Respondent: Yeah**

**Moderator: Ok, we can go to another question. We are going to look at them on levels. We shall begin on individual level or patient perspective**

**Respondent: The factors that…**

**Moderator: What are the factors that contribute to hypertension to the patients you see at individual level?**

**Respondent: At individual level if I were to look at a factor,**

**Moderator: Mmmmhh**

**Respondent: one of the factors is alcoholism**

**Moderator: Mmmmhh**

**Respondent: At individual level**

**Moderator:** Mmmmhh

**Respondent: Eeeehh. Smoking**

**Moderator:** Mmmmhh

**Respondent: It may increase**

**Moderator:** Mmmmhh

**Respondent: Poor diet intake will increase**

**Moderator:** Mmmmhh

**Respondent: That’s on individual level**

**Moderator:** Mmmmhh

**Respondent: And most of the time you will find out that there is that hereditary factor**

**Moderator:** Mmmmhh

**Respondent: Hereditary factor may affect some because one will tell you that you know my mother, my father, my parent are hypertensive, so my case is not different from them**

**Moderator: Mmmmhh**

**Respondent: Yeah**

**Moderator: Anything to add?**

**Respondent: Eeeehh, I talked of poor diet**

**Moderator: You have talked about poor diet, smoking, alcohol, hereditary factor**

**Respondent: Hereditary factor and weight gain or weight loss**

**Moderator: Mmmmhh**

**Respondent: Yeah**

**Moderator: Ok**

**Respondent: And there are other mental. Let me talk of mental problems**

**Moderator: Mmmmhh**

**Respondent: For instance in this hypertensive status you will find out that one is hypertensive; both HIV positive has either a case of sarcoma or cancer**

**Moderator:** Mmmmhh

**Respondent: You will find out that they affect either directly or indirectly**

**Moderator:** Mmmmhh

**Respondent: Because many look at it like however much I take medicine at the end of the day am going to die**

**Moderator:** Ok. What about if we talk about their sex or age?

**Respondent: Age**

**Moderator:** Mmmmhh

**Respondent: Age is a factor because most of the time you find that advanced age will affect the hypertensive**

**Moderator:** Mmmmhh

**Respondent: But I think it’s rarely a factor because most of the time we diagnose**

**Moderator:** Mmmmhh

**Respondent: it’s rarely a factor because most of the time we diagnose the HB you will find that come at the age of 35 and above**

**Moderator:** Mmmmhh

**Respondent: Anyway my cases I have never found. Very few cases are less than 35**

**Moderator:** Ok

**Respondent: Yeah**

**Moderator:** What about when we talk about community and family level, what factors contribute to uncontrolled hypertension in the patients you see?

**Respondent: Community and family level, you will understand that stigma is one of them**

**Moderator:** Mmmmhh

**Respondent: Many are in stigma and they are in denial**

**Moderator:** Mmmmhh

**Respondent: When one is in denial and in stigma, you will find out that the stigma from the community will cause it,**

**Moderator:** Mmmmhh

**Respondent: Or some, some due to those underlying factors that we talked off, they have to travel very far so that the community does not understand them very well**

**Moderator:** Mmmmhh

**Respondent: For instance there is one that is HIV positive and also hypertensive**

**Moderator:** Mmmmhh

**Respondent: They won’t come to the facility around around due to stigma**

**Moderator:** Mmmmhh

**Respondent: They have to go very far but in the process it may affect either directly or indirectly because if we undertake roles as per the community**

**Moderator**: Mmmmhh

**Respondent: We will take earlier measures than when one has to travel a long distance to know the BP readings**

**Moderator:** And when you talk about traditional beliefs

**Respondent: You know I don’t believe in such things**

**Moderator:** Mmmmhh

**Respondent: But it may affect. Traditional beliefs may affect depending on the community or the tribe**

**Moderator:** Mmmmhh

**Respondent: There are tribes that have a lot of traditions; others will say that since am going to a facility, in case I don’t find a lady to look at me as a health care provider**

**Moderator:** Mmmmhh

**Respondent: I won’t accept**

**Moderator: Mmmmhh**

**Respondent: For instance take to it that we are only male,**

**Moderator: Mmmmhh**

**Respondent: Will they meet their challenges or whatever they have to undertake? One will say that if they are treated by a make or a female they can get better.**

**Moderator:** Mmmmhh

**Respondent: You know that’s a traditional belief**

**Moderator:** Mmmmhh

**Respondent: Yeah**

**Moderator:** And on provider perspective, you are the provider yourself

**Respondent: Yeah**

**Moderator:** Provider perspective, what are factors that contribute to hypertension in the patients you see

**Respondent: Come again**

**Moderator:** We are talking about the factors that contribute to hypertension in the patients you see, now at provider’s perspective. That is you

**Respondent: Provider, me as a whatever?**

**Moderator:** Mmmmhh

**Respondent: On the side of provider I think it’s just the attitude**

**Moderator:** Mmmmhh

**Respondent: The attitude will affect because you will find there are other providers me not, me inclusive**

**Moderator:** Mmmmhh

**Respondent: We would look at such patients with a low grade or we undermine or we just look at them with a very limited perspective**

**Moderator:** Mmmmhh

**Respondent: You will find out maybe this was, this is a life threatening case and the provider says that its ok, it will just be fine, it will just be ok**

**Moderator:** Mmmmhh

**Respondent: Yea**

**Moderator**: Ok. What about at health systems level?

**Respondent: Aaaahhh, health system as per the facility or per globally**

**Moderator:** Facility as we talk about service delivery, health force, drugs, the information, finance

**Respondent: Most of the time you will find out that at the health facility level you will find out that there are cases whereby**

**Moderator:** Mmmmhh

**Respondent: There is less provision as per the administration in terms of drugs,**

**Moderator**: Mmmmhh

**Respondent: Certain drugs because of their pricing**

**Moderator:** Mmmmhh

**Respondent: There are cases where you find certain drug does very much well**

**Moderator:** Mmmmhh

**Respondent: But some cannot be provided in bulk because of the pricing**

**Moderator:** Mmmmhh

**Respondent: Secondly it depends on who usually take medicine at what time**

**Moderator:** Mmmmhh

**Respondent: You will find out for instance like there is a patient who has been taking a certain drug for a period of time**

**Moderator:** Mmmmhh

**Respondent: So whenever he or she come for the drug at the facility will find that always the medicine is not there**

**Moderator:** Mmmmhh

**Respondent: Now when you find out that the medicine doesn’t come or it is not in position every time and again**

**Moderator**: Mmmmhh

**Respondent: You will find out that it will affect the response to the treatment of the patient**

**Moderator:** Ok. What about the policy level. When you talk about

**Respondent: Policy level?**

**Moderator:** Yes. Nationals

**Respondent: Nationally?**

**Moderator:** Yes. Guides, how about the state, the local laws and the guidelines

**Respondent: At the policy level, I usually take to it that you know most of the medicine that we have now**

**Moderator:** Mmmmhh

**Respondent: Most medicines are generic drugs**

**Moderator:** Mmmmhh

**Respondent: We cannot reach the original drugs because of the prices or the prices are very high**

**Moderator:** Mmmmhh

**Respondent: Now when it comes to nationally there must be a new outlook on the way this medicine comes in**

**Moderator:** Mmmmhh

**Respondent: And the cases where we find out that there is a very good drug, you know it’s very good, the composition is very good but no response**

**Moderator:** Ok

**Respondent: How do we go about it in such a case?**

**Moderator:** Mmmmhh

**Respondent: Where by you find they talk of contraband, you fail to understand is it contraband**

**Moderator:** Mmmmhh

**Respondent: is it the medicines that are not responding**

**Moderator**: Mmmmhh

**Respondent: Or are these the contraband where by you are told this is good and whenever you give to the patient there is no response**

**Moderator:** Mmmmhh

**Respondent: What do we do? How do we go about it**

**Moderator:** Ok

**Respondent: Hallo**

**Moderator:** Halloo, yes I can hear you

**Respondent: The other factor is that nationally they keep changing the guidelines**

**Moderator:** Mmmmhh

**Respondent: You know when they keep changing the guidelines, Ooohh, you need to use these before a term ends you find they have changed**

**Moderator:** Mmmmhh

**Respondent: The guidelines keep on changing and with no proper training we will remain in the other decade where we were using this and there are new guidelines**

**Moderator: Ok**

**Respondent: Yeah**

**Moderator:** Another question, we are almost done

**Respondent: It was long**

**Moderator:** Just hold on a bit

**Respondent: This one needs a big salary (laughing)**

**Moderator**: In your view, what would be the possible solutions to the challenges you have mentioned. You had mentioned about poor response to medication. What solution do we have for that?

**Respondent: Now solutions**

**Moderator:** On the challenges you have mentioned

**Respondent: if I were to look at the solutions**

**Moderator: Mmmmmhh**

**Respondent: Already you have the solutions with you**

**Moderator:** Mmmmhh

**Respondent: Because we have to start nationally**

**Moderator:** Mmmmhh

**Respondent: We have to start with national solutions**

**Moderator:** Mmmmhh

**Respondent: We come to community solution**

**Moderator:** Mmmmhh

**Respondent: Individual solutions**

**Moderator:** Mmmmhh

**Respondent: And health care solution, health providers solutions**

**Moderator:** Mmmmhh

**Respondent: Now when we look at nationwide solutions,**

**Moderator**: Mmmmhh

**Respondent: We have to have regular trainings concerning the guidelines**

**Moderator:** Mmmmhh

**Respondent: New guidelines**

**Moderator:** And how to go about it

**Respondent: Good provision of good medicine**

**Moderator:** Mmmmhh

**Respondent: And especially since hypertension is a risking factor, they have to look at it that for instance it’s causing a lot of deaths,**

**Moderator:** Mmmmhh

**Respondent: The medicines for TB are free, the medicines of HIV are free, and why don’t they make the medicine for hypertension free as well?**

**Moderator:** Mmmmhh

**Respondent: That’s one of the factors nationally**

**Moderator:** Mmmmhh

**Respondent: When we come to community level, the community must have regular education concerning hypertension**

**Moderator:** Mmmmhh

**Respondent: Because for instance I was talking about my rural outfit especially viwandani**

**Moderator:** Mmmmhh

**Respondent: In Viwandani you will find out that these people are hypertensive and they rarely know that they are hypertensive**

**Moderator:** Mmmmhh

**Respondent: Why do they give them education?**

**Moderator:** Mmmmhh

**Respondent: To understand that this disease is a killer disease and it can cause paralysis and whatever. So we have to have regular education concerning hypertension**

**Moderator:** Mmmmhh

**Respondent: Eeeehh, at the local levels, barazas chief barazas, community health care workers, community health…they are called CHEWs?**

**Moderator:** Yes

**Respondent: To keep on teaching the whatever and there must be a way were by enough machines, enough BP machines ore on the ground**

**Moderator:** Mmmmhh

**Respondent: Whereby these people must be regular, the regular measure or testing**

**Moderator:** Mmmmhh

**Respondent: Testing BP doesn’t cost a lot**

**Moderator:** Mmmmhh

**Respondent: I guess that will be one of the approach to counter the whatever**

**Moderator:** Mmmmhh

**Respondent: As per the community we must remove the stigma**

**Moderator:** Mmmmhh

**Respondent: Because when one, we must be able to tell the community at large that the when we take HBP readings it doesn’t mean that they are sick**

**Moderator: Mmmmhh**

**Respondent: And it’s not associated to other diseases such as HIV and whatever**

**Moderator:** Mmmmhh

**Respondent: That is one of factors**

**Moderator:** Mmmmhh

**Respondent: Coming to the health we as health providers**

**Moderator:** Mmmmhh

**Respondent: We must have meetings; we must have diaries we must have… for instance if I were to take a good example**

**Moderator:** Mmmmhh

**Respondent: I worked in Kiambu**

**Moderator:** Mmmmhh

**Respondent: In Kiambu we had regular clinics**

**Moderator:** Mmmmhh

**Respondent: every month for hypertension**

**Moderator:** Mmmmhh

**Respondent: there was a day every month or twice a month where the local communities were called to have free testing for hypertension and whatever**

**Moderator:** Mmmmhh

**Respondent: And in case you found out that you are hypertensive there must be a follow up**

**Moderator:** That’s per the facility

**Respondent: I think we need time to tame the administration not only in my facility and how do we enhance that**

**Moderator: Mmmmhh**

**Respondent: we can only enhance through regular teachings, regular CMEs and whatever**

**Moderator:** Mmmmhh

**Respondent: Eeeehh, and there must be diaries or there must be registers for the hypertensive**

**Moderator:** Mmmmhh

**Respondent: And in case there is a register we usually have CME and the administration at large should be open to the patient**

**Moderator:** Mmmmhh

**Respondent: Whereby the patients are enlightened and told what to do next**

**Moderator:** Mmmmhh

**Respondent: Not just in my facility but all facilities at large**

**Moderator:** Mmmmhh

**Respondent: Coming to individual levels, individual level is all about education**

**Moderator:** Mmmmhh

**Respondent: Educating them how they can help themselves in case on how to control pressure**

**Moderator:** Mmmmhh

**Respondent: Either through the diet, exercise and whatever**

**Moderator:** At individual level you had talked about poor response to medication and you had talked about cholesterol level and echo, affordability and you had talked about finance. What are the solutions to them?

**Respondent: You know when it comes to finance, how do they get a place where they are cheaper**

**Moderator:** Mmmmhh

**Respondent: Like for instance echo usually take eight to nine thousand**

**Moderator:** Mmmmhh

**Respondent: Now, can they afford?**

**Moderator:** Mmmmhh

**Respondent: I guess no**

**Moderator:** Mmmmhh

**Respondent: Cholesterol levels, the reading of cholesterol is between from twelve and twenty five hundred**

**Moderator:** Mmmmhh

**Respondent: are they able to meet the whatever**

**Moderator:** Mmmmhh

**Respondent: My guess is no**

**Moderator:** Mmmmhh

**Respondent: Now on an individual level there must be a way to assist there**

**Moderator:** Mmmmhh

**Respondent: When it comes to diet**

**Moderator:** Mmmmhh

**Respondent: Diet I guess that everybody is in a way whereby they can control the intake and they understand their BMI**

**Moderator:** Mmmmhh

**Respondent: Because when they come for BMI test, we can undertake the BMI**

**Moderator:** Mmmmhh

**Respondent: Whereby we tell them this is risky. I think that’s cheaper**

**Moderator:** Response to medication

**Respondent: Response to medication is either they take their medication regularly or they take quality medicine that will help out**

**Moderator:** Mmmmhh

**Respondent: Or in case there is any change,**

**Moderator:** Mmmmhh

**Respondent: An individual must understand that this medicine is not working for me**

**Moderator:** Mmmmhh

**Respondent: And health provider X or Y has told me to change the medication or the medication may be higher than I expect**

**Moderator:** Mmmmhh

**Respondent: They should have a way to control this through their pockets. That is in an individual level**

**Moderator:** Ok

**Respondent: Yeah**

**Moderator:** On another question. Currently we are in COVID 19. How has this affected your provision if care to patients in your community

**Respondent:** Very high

**Moderator:** Mmmmhh

**Respondent: It has affected greatly**

**Moderator:** In terms of hours of operation, how is it?

**Respondent: Our hours of operation we are operating at 24 hour basis so we have not been affected**

**Moderator:** Mmmmhh

**Respondent: But the curfew has affected especially how the patients have been attending their clinics is low**

**Moderator:** Mmmmhh

**Respondent: But our hours have never been affected directly or indirectly therefore others have been affected indirectly**

**Moderator:** Mmmmhh

**Respondent: But the way we have to manage our patients is minimal**

**Moderator:** Mmmmhh

**Respondent: Because many know that by such a time many hospitals will not be open**

**Moderator:** Mmmmhh

**Respondent: They don’t understand that we are a 24 hour system therefore we can work all the time**

**Moderator:** Mmmmhh .The availability of drugs for hypertensive at this time?

**Respondent: The availability of drugs at this moment depends on the way I had put it**

**Moderator:** Mmmmhh

**Respondent: The cheaper drugs we have them in provision all the time but when it comes to expensive drugs we usually prescribe to the patient**

**Moderator:** Mmmmhh

**Respondent: With education we usually advise them that this drug maybe better for you**

**Moderator:** Mmmmhh

**Respondent: So you have to ensure that you take it and the moment or time you take it you must give us the result**

**Moderator:** Mmmmhh

**Respondent: or you come by so that we know whether they are responding or not**

**Moderator:** Ok. And at the moment, what about prioritizing?

**Respondent: Priorities?**

**Moderator**: Mmmmhh

**Respondent: Drugs over food?**

**Moderator:** Changing of priorities because of the COVID19. The care, hypertensive care

**Respondent: You know there is no that direct contact**

**Moderator:** Mmmmhh

**Respondent: We rarely take the direct contact because we don’t know**

**Moderator:** Mmmmhh

**Respondent: You never understand who is positive and who is negative**

**Moderator:** Mmmmhh

**Respondent: Now, keeping distance has affected greatly because**

**Moderator:** Mmmmhh

**Respondent: Just take for instance the way we have to take our readings**

**Moderator:** Mmmmhh

**Respondent: You know reading can’t take one and a half meter apart**

**Moderator:** Mmmmhh

**Respondent: Now how do you risk yourself/**

**Moderator:** Mmmmhh

**Respondent: You must risk whoever is in contact or you must go the reverse way**

**Moderator:** Mmmmhh

**Respondent: You must use the back side**

**Moderator:** Mmmmhh

**Respondent: You will find that it’s challenging anyway**

**Moderator:** Mmmmhh

**Respondent: As per the COVID we have to take precautions**

**Moderator:** Mmmmhh

**Respondent: Because you may think that it is hypertension and in the process you find out it’s not hypertension**

**Moderator:** Mmmmhh

**Respondent: Yeah**

**Moderator:** At this time, what about outreaches?

**Respondent: Outreach?**

**Moderator:** Mmmmhh

**Respondent: Outreach is completely out of**

**Moderator:** Mmmmhh

**Respondent: It is no, completely no**

**Moderator:** Ok

**Respondent: We cannot use outreach because there is no association around allowed**

**Moderator:** Mmmmhh

**Respondent: So we have just to wait for our patients just in the facility**

**Moderator:** Ok

**Respondent: Yeah**

**Moderator:** Is there anything else that has been affected during this time and we have not talked about COVIDD 19?

**Respondent: Directly COVID19 or without COVID 19?**

**Moderator:** Within this CIVID 19, the care, is there any other thing you would want to talk about?

**Respondent: Eeeehh, on which grounds?**

**Moderator:** At this time we are talking about COVID 19

**Respondent: Yeah**

**Moderator:** How you have been taking care of hypertensive care in that community. Is there anything else you have left out that you would want to talk about?

**Respondent: I gave everything unless I were to remember at a later stage**

**Moderator:** Ok

**Respondent: I guess I have given you everything I knew or I know**

**Moderator:** Ok

**Respondent: Yeah**

**Moderator:** As we wind up, is there anything else that you would like to talk about in regards to hypertension

**Respondent: In regards to hypertension, I’ll talk of you**

**Moderator:** Mmmmhh

**Respondent: You APHRC, you have to look in to it that the community is well endorsed I concerning hypertension**

**Moderator:** Mmmmhh

**Respondent: Because to me I understand that nearly everything at national and all levels has been catered for**

**Moderator:** Mmmmhh

**Respondent: You understand that this hypertension is a disease that can kill**

**Moderator:** Mmmmhh

**Respondent: It can cause a lot of damage, how do you. You should come up with a portfolio or at least a way out**

**Moderator:** Mmmmhh

**Respondent: so that the community at large**

**Moderator:** Mmmmhh

**Respondent: Especially education wise, they have to understand**

**Moderator:** Mmmmhh

**Respondent: This old age, there must be a way they can be reached because old age in society they don’t come to hospital**

**Moderator:** Mmmmhh

**Respondent: And since you have a way of reaching the community**

**Moderator:** Mmmmhh

**Respondent: Either through your outreach methods**

**Moderator:** Mmmmhh

**Respondent: You should find a way of reaching these old aged**

**Moderator:** Mmmmhh

**Respondent: Eeehhh, what else, what else**

**Moderator:** Mmmmhh

**Respondent: I think just education and making this old aged just understand how they can be assisted directly or indirectly**

**Moderator:** Ok

**Respondent: By your either APHRC or via the government**

**Moderator:** Ok

**Respondent: Yeah**

**Moderator:** Ok. Thank you so much for your time

**Respondent: Yeah**

**Moderator:** The contribution that you have given me on this research is going to be very useful

**Respondent: Yeah**

**Moderator:** Thank you, have a good day

**Respondent: Same**

**…End…**
