# Supplementary material for: A socio-ecological framework examination of drivers of blood pressure control among patients with comorbidities and on treatment in two Nairobi slums; a qualitative study
Source: PLOS Glob Public Health. 2023 Mar 10;3(3):e0001625. doi: 10.1371/journal.pgph.0001625 (PMC10021823; doi:10.1371/journal.pgph.0001625)
Supplement: S2 File — (ZIP) [file pgph.0001625.s002.zip › Health Facility/VIWA_KII_HP_200626_0102.docx]

**Moderator: {Name}**

**Code: VIWA-KII-HP-200626-0102**

**Moderator**: Confirm that I have read and that you have understood the information that I’ve read to you and you have had the opportunity to ask information and I have answered you to your satisfactory

**Respondent**: **Yes**

**Moderator**: So you are advised to say yes or no.

**Respondent: Yes**

**Moderator:** Confirm that I have read and that you have understood the information that I’ve read to you and you have had the opportunity to consider the information, ask questions and I have answered you to your satisfactory

**Respondent: Yes**

**Moderator:**  You understand that your participation is voluntary and that you are free to withdraw at any time without giving any reason and without any of your legal rights being affected

**Respondent**: **Yes**

**Moderator**: You understand that the data collected during this study may be looked at by individuals where it is relevant to your taking part in this study. You give permission for these individuals to have access your data

**Respondent**: **Yes**

**Moderator**: You confirm to be audio recorded and you also consent to use the anonymized verbatim quotations

**Respondent:** **Yes**

**Moderator**: You are happy for your data to be used in any future research

**Respondent**: **Yes**

**Moderator**: You agree to take part in the above study

**Respondent**: **Repeat**

**Moderator:** You agree to take part in the above study

**Respondent:** **Yes**

**Moderator**: Ok, I am going to read to you a few questions and I will head into the questions.

**Respondent**: **ok**

**Moderator**: I am going to read to you a few questions and then you will be able to answer. So this community has been identified to have a high burden of uncontrolled hypertension which is leading risk factor to premature death and disability. So I am trying to gather information about the provision of hypertension care in this community particularly to patients on treatment and who have their blood pressure not under control. So I’m seeking your views on uncontrolled hypertension among those on treatment in this community and factors driving these high rates. So onto the first question kindly Please tell me about hypertension care in your community.

**Respondent: Ok, hypertension in short is the high blood pressure, then we have the normal which is 120 over 90 and the elevated blood pressure which ranges from 130 and diastolic above 89 and now we have high blood pressure where there are clients who come systolic above 139 and diastolic above 90, so in my facility right now where I am practicing, I have been experiencing such clients. Some have been attached tom medication (Not clear)**

**Moderator:** Hello, {Name of the Respondent}, Can’t hear you clearly.

**Respondent: I am saying I’ve been also experiencing apart from the outpatient hypertension clients, I have been also …. (Not clear) hypertension in pregnancy I hope you hear me**

**Moderator:** your network isn’t so clear

**Respondent: Let me move to a far much better place**

**Moderator:** Yeah

**Respondent: I** **am saying in terms of the pregnant mothers, I have been putting them on medication until delivery and I do follow up for one month and they turn to be ok, outpatient we have hypertensive but the owner of the facility prefer us doing follow up in medical outpatient bigger facility like Mbagathi or Mama Lucy so we have been especially because they deal with NHIF client we’ve been putting them on under hypertensive and then we fallow them through MOPC we refer them to Mama Lucy or Mbagathi ,so that is the information I can give you about hypertension that is what we have been getting clients but is measures we have been taken. And for pregnant mothers whom we have put on medication and it seems the blood pressure continues to be high we refer them to Mama Lucy for follow up**

**Moderator:** Ok, so your facility usually just handle hypertension just for outpatient’s services alone

**Respondent: No we do general treatment but we don’t specialize on ‘’nini’’ because I understand that BM and hypertension they need to be done follow up that is why we refer them but if we get them we stabilize them we refer for follow up**

**Moderator:** Ok so please tell me about hypertensive clinic in your facility.

**Respondent: What?**

**Moderator:** The hypertensive clinic you have said you do not have a hypertensive clinic in your facility.

**Respondent: Yes, we do not have as a whole facility that is why we refer.**

**Moderator:** Kindly give me the National guidelines; are there any National guidelines in your facility that you follow for hypertension?

**Respondent: No right know I don’t know, I don’t have**

**Moderator:** What do you usually use to treat your patient you have found at your clinic?

**Respondent: at our facility we use initially use I have Enapril that’s what I use to ANC mothers who are pregnant with hypertension and then for the outpatients, what we have is nefefipine but then before we used to have the chlorothiazide there some information that it has got some side effects it was cause that it was banned Some am reluctant in using it but in my case I get some clients who come from private facilities and they are under it. I maintain them on it but I refer them. So my case I am using enapril and chlorothiazide**

**Moderator:** Do you see client with hypertension and other conditions in your facility?

**Respondent: As combination of, there is some time I had a hypertensive client and also she was diabetic and it was like she was done follow up in a private facility what I did I referred him to be booked at Mama Lucy for MOPC but currently I had a client for a newly diagnosed diabetic client and still referred to Mama Lucy**

**Moderator:** So apart from diabetic which other condition having been seeing that are co-relating with hypertension

**Respondent**: **Apart from DM I don’t think I’ve experienced any other condition related to hypertension, ok there is renal condition I remember there is a boy who came who had hepatitis and that I believe is early this year and was January I referred because there was time he was diagnosed with hepatitis and I felt it is much better to refer for follow up from the facility where he was being managed. Still I got another client who had hepatitis sometimes later years back ago but still I referred. I got another client who had, I thought it was a renal condition because he was anemic so I referred, to Mama Lucy for further evaluation**

**Moderator: Ok** so you have mentioned of diabetes and hepatitis and renal, this client you said you refer to Mama Lucy most of the time for follow up and MOPC Do you have any other guidelines for this specific patients you refer like the ones you have said diabetes with hypertension or hepatitis with hypertension

**Respondent**: **I** **don’t have any guidelines to be sincere**

**Moderator:** How long have you not had guidelines from?

**Respondent: Since I came here whatever guidelines I have in this facility it is about malaria because there is time I went for the training of malaria and pneumonia for under-five I have guidelines for malaria and the under 5 about diarrhea and vomiting so for hypertension , DM I don’t have any guidelines**

**Moderator:** Ok on the third question what are the factors that are associated with good and poor blood pressure control

**Respondent: One thing it is about if the client is on medication and isn’t taking the drugs well. That’s one thing, the other thing is tracing of the clients as in if I don’t have enough that is insufficient information about the blood pressure can also lead to that also communication barrier can lead to that and also diet in terms of nutrition because currently we have been having a nutritionist who have been volunteering at our place so every time in getting such clients either diabetic or hypertensive in the clinic I refer them so that he tell them about diet what we are supposed to use and what we are not supposed to use**

**Moderator:** you have talked about client defaulting, you don’t have information for the client for you to be able to trace them, and you also talked about communication barrier between you and clients that you have

**Respondent: And also insufficient knowledge about hypertensive in terms of the clinician that is our part**

**Moderator:** And all these you have told me are falling in the category of poor blood control pressure, what are the factors that are associated with good pressure control.

**Respondent: Factors that are associated with good pressure control, one thing the client must know about their lifestyle like this who smoke if you keep on smoking then, so if stop smoking that means you are doing good. If you do some exercise you are doing good and also healthy diet like a lot of salt or things like if you maintain healthy diet also you are doing good in terms of your blood pressure. If you take your medication well**

**Moderator:** Ok taking medication well, healthy diet, exercise and knowledge about your lifestyle.

**Respondent: Yes**.

**Moderator:** Anything else you would want to add to that.

**Respondent: I don’t think whether I have something for right now**.

**Moderator:** Ok thank you for that. So what challenges do you encounter in provision of health care services to the patient that you see with uncontrolled blood pressure?

**Respondent: One thing the client might be lacking enough knowledge on what is hypertensive because some you refer them and they come a month later, they have not gone where you sent them another thing consultation because am clinician we don’t have people like MO, you know sometime you find if you someone to consult it would be able to attend well without referring and also some client when you put them on medication and they take find their blood pressure is stabilizing they stop taking the medication and also availability of drugs though now it is a thing something that need follow-up .I believe the reason behind having the availability of drugs because we don’t try follow up clinic at our place.**

**Moderator**: Currently, you have talked about there is no consultation the client don’t have knowledge and client immediately they walk out of your facility and you’ve said you have issues also stock out of drugs. Do you have any challenges with your facility hours?

**Respondent: Challenges in terms of what?**

**Moderator:** Your facility hours

**Respondent:** **No we don’t have because this is 24 hours facility**

**Moderator:** Ok, you work from what time to what time?

**Respondent: From 8-6**

**Moderator:** That is for the people who come for morning shift

Respondent: Yes

**Moderator:** and the people who come from night come from 6 to?

**Respondent: Still 8 in the morning**

**Moderator:** You’ve said that you’ve had stock out on medication, how are you handling clients at the moment?

**Respondent: In terms of what?**

**Moderator:** You’ve said that you don’t have drugs at the moment, how are you handling your clients at the moment that came for a refill?

**Respondent: Right now I give what is available and I refer, as I was saying we don’t have a variety of ant hypertensive because we don’t run those clinics but have something available to just stabilize them before we refer.**

**Moderator:** And then how about capacity of workload on the employees that are providing care. Do you have any challenges with that?

**Respondent:** **No we don’t have challenge.**

**Moderator:** So the challenges that you face with prescribing medication to patients with hypertension kindly tell about that, do you have challenges when you prescribing medication to you clients?

**Respondent:** **Yes I have coz I don’t have guidelines so sometimes i may have to call my colleagues who are working in government facility to verify what am supposed to do.**

**Moderator:** What of with clients who already in treatment do you have challenges in changing of prescription?

**Respondent: Yes because I am not who put them on the treatment and blood pressure are fluctuating, so for me to change the regime it may be very difficult because I Have to consult someone who put them on medication of whom Don’t know**

**Moderator:** Do you have a challenge in increasing the number of medication and also the strength?

**Respondent: Yes because I don’t have the guidelines the current for that**

**Moderator:** Ok thank you for that. What are the factors that contribute to uncontrolled hypertension in the clients that you see? I am going to give you the breakdown on the levels that I would want you to break down to for example in an individual or patient perspective what do you think are the factors that contribute uncontrolled hypertension .You had mentioned early about the clients not having knowledge for the medication they take and their lifestyle, kindly give me more of that

**Respondent: Ok, one thing in terms of dosage of the drug, there you may find that the client is taking under dose another thing in terms of financial some client some patient clients are not using NHIF and even if you prescribe they may lack money others may not understand sometime you may prescribe if you write 1x3 maybe they may not understand what you are writing and also the client may also sometime in terms of kind of work they are doing ,there are also those who will feel as if they are over burden to carry their medication to their place of work,**

**Moderator:** You have talked about having challenges from the clients, communication barrier sometimes you write something they don’t understand and then you have said about the kind of work they are doing they aren’t able to take their drugs and also you have talked the burden of pills from the patient’s perspective. so anything you want to add to that?

**Respondent: Even also the side effects of the drugs the client is using because in hypertension there are some drugs which client either to feel drowsy, dizziness or side effects so they might fear taking the drugs because of the side effects**.

**Moderator:** Ok and from the family or community level perspective, what factors do you think might contribute to uncontrolled hypertension in patients that you see

**Respondent: One thing is about social economic status where am living, it’s a place where people are of low social economic status. many clients you find they are smoking , taking alcohol and you find that for someone to withdraw from smoking and taking alcohol is not that easy**

**Moderator:** Anything else you would like to add?

**Respondent: For now no.**

**Moderator:** The family level I was talking about, do you have challenges like in the environment level? The tradition beliefs that the client may have told you

**Respondent: Repeat yourself**

**Moderator:** am saying in the community level, perspectives I would like to give you a few examples, so from the family level, means the tradition believes the client have the environment and probably certain thing, Do you think they have an effect in uncontrolled hypertension

**Respondent: For that I may lack information to give you for that.**

**Moderator:** Ok that is fine. And from the providers perspectives what are the challenges that are leading to uncontrolled hypertension?

**Respondent: I will still repeat again the guideline. You know the guideline will give us about the first line second line and the combination of the medication, so lack of enough knowledge about the current management of hypertension is it that is what I can talk about providers.**

**Moderator:** Anything you would to add?

**Respondent: And also other thing which may in term of site we provider we support the initiation of you know clients come already when they have been initiated, they had been put on medication so for you to change the medication you don’t have the guideline is another thing which also would affect our side as providers**

**Moderator:** Ok, so from the health system level perspective, what do you think could lead to uncontrolled hypertension?

**Respondent: The follow up, Client follow up**

**Moderator:** and then you have also talked about you don’t have a specific clinic for you patients.

**Respondent: Yes.**

**Moderator:** You also talked about you do not have someone to consult

**Respondent: Yes**

**Moderator:** You also talked about you have few drugs

**Respondent: Yes**

**Moderator:** So anything else you would want to add on that?

**Respondent: For now I can say no.**

**Moderator:** Now from the policy level perspective, policy level means the people who give you the guide lines, what do you think are the factors that are bringing this high blood pressure to be uncontrolled?

**Respondent: As in from?**

**Moderator**: From the policy level perspective from the national or the government level what do you think is leading to uncontrolled hypertension in your facility?

**Respondent: Maybe if could be in health facility could arrange things like seminars we can be taught about that and also may find a way on how the facility could be helped in linkage to hypertension that I believe it can help us Moderator**: Anything you would want to add to that

**Respondent: For now?**

**Moderator**: So what do you think are the possible solutions for the challenges you have mentioned are many and we would still go to the same things that you told me about the individual community health system and policy. So on the individual level you have talked about under dose some client are coming when the already have under dose they have no finances , they have communication barrier, the kind of work they probably don’t make them take their drugs and then you have talked about the burden of pills and you also mentioned some clients don’t want to continue taking their medication because of the side effects that they have .So what do you think could be a possible solution to some of the challenges that I have mentioned?

**Respondent: As on the solution, one thing we have guideline for the management of hypertension. Medication for hypertension to be availed and then coordination in terms of consultation .We may be coordinated with people who can consult and also the health talk for the hypertensive clients**.

**Moderator**: The guideline you are talking about for the guideline that you are saying that it will be provided I think that falls under provider perspective and policy level perspectives so that this clients can be able to get their information on individual level I would want to know, this is now from the patient think perspectives. What do you think would be the possible solution from them, have no finances ,some of them have communication barrier between you and them and you also talked about burden of pill and side effect of the medication, What do you think would have possible solution would you have for those problems you have told me about ?

**Respondent: One thing we need the guideline for the management of hypertension, medication for hypertension to be availed, coordination in terms of consultation. We be coordinated with people who we can consult and also health talks for the hypertensive clients**

**Moderator:**  The guidelines that you are asking to be provided I think they falls under the providers perspective and policy level perspective so that these clients are able to get their information. So from the individual level, I would want to know. This is now from the patients’ perspective. What do you think would be the possible solution to the things you mentioned from individual perspective which you said clients lack finances, communication barrier, and burden of pills and also the side effects of the medications? So what do you think would be solution to those problems that you had told me about?

**Respondent:** **In terms of finance maybe my suggestion is that because hypertension is very common in a country, if the government can provide either free medication for the hypertension, or drugs which have no price. I think it can help .and also in terms of health education to clients about hypertension and what are the complication of hypertension because most of them lack the information of complication of hypertension things like stroke you find yourself you have a kidney failure because of blood pressure that you have been living with it without being managed ,and if they could be taught about the risk factors which can lead to hypertension like alcoholism smoking things like that ,and if they could be educated about healthy diet to prevent hypertension.**

**Moderato**r: Ok that is fine from the community and family level perspectives you talked about the social economic status of your client and also talked about your clients mostly not being able to leave taking alcohol and smoking. What do you think could be the possible solution to that?

**Respondent: That is what I was saying there could be a facility, in terms of nutrition I believe it can help. Yes that one can help. If we could be having people, that are the people who that is the community health worker if they can participate in educating the community because there are people who are living in the community and there are hypertensive and they don’t know the repercussions of continuing taking alcohol and smoking.**

**Moderator**: From the provider perspectives we talked about no guidelines and then you also talked about lack of knowledge for the people who are already taking care of hypertensive clients and you also talked about you are not so sure about initiation of the prescription that you to start with what do you think would be a possible solution to that?

**Respondent: That’s why I was talking about attending the facility level can be able to be assisted in terms attending any attending CME about hypertension, if there are manuals guidelines for current management of hypertension if they can be provided and in terms if there is a way we can be touch with the health facility whom we refer our clients so that we may be able to do some follow up. There could be better communication between us and the facility and the referral Facility level , if we can be having a communication with clinician or the physician whom we are referring our clients that is our follow up for our clients**

**Moderator:** From the health system perspectives you talked about no fallow up of which we already discussed that you should be having fallow up for your clients from whenever place you are sending them to and then you talked about that you don’t have a specific clinic for these hypertensive clients and you also talked about that you don’t have sufficient drugs for clients that you see. What do you think would be the possible solution to that?

**Respondent: If the facility could be able to put some special case clinic like because it is not about hypertension only, I told you this is a health center and is not a big facility but if it could be able to establish such cases of special clinic like hypertension clinic, diabetic clinic even for ophthalmology and ENT we don’t have. Those we just refer. So is the facility was able to establish some of those special clinic that could be better.**

**Moderato**r: What are the possible solutions for policy level, you’ve talked that you don’t have seminars, and said there is no linkage to the facility like you don’t get the guidelines themselves you are not called to any training .What do you think could be the possible solution for that?

**Respondent: I have told I believe that is the question I have answered for provision, in short if the facility is able, it can provide whatever we are lacking**.

**Moderator:** So a question for example in a day how many of the clients of patients who have hypertension do you see?

**Respondent: Let me say they are not many and is not every day it is sometime. You find in a month you have encountered either 3 or 4 clients in a month**

**Moderator:** In a month?

**Respondent**: **Yes but in every month I encounter 1, 2 or 3.**

**Moderator**: So now everyone is talking of COVID situation and how it has affected the world and the country, so how has the current COVID situation affected provision of care from your community with hypertensive client?

**Respondent**: **Sometimes, in terms of examination of client because of maintaining that one meter. So in terms examination close contact**

**Moderator**: Anything else?

**Respondent**: **And also still you may find that client although they are hypertensive they might fear to come to hospital because they have the information like we as health workers we are the most vulnerable when it comes to COVID 19 so we might lack clients because they fear to come to the facility .**

**Moderator:** What of the hours of operations, has it affected your current situation in your facility?

**Respondent**: **Not but not in terms of the clients, that one I can talk in term when there was curfew but now that the curfew has been extended to 9pm I don’t think it could in terms of hours it could have affected because by 7pm everybody was supposed to be in the house, so whenever they come out because of insecurity, there are security failed to understand them whether they are going to hospital but for now because the curfew was extended don’t think there any problem.**

**Moderator**: What about the availability of antihypertensive medication, has it affected?

**Respondent**: **Yes the availability it has affected because there is a client we got and believe it was yesterday and he has been going to private health facility and he was on low losartan so what we did , we advised him to be followed having level 5 hospital like either Mbagathi or Mama Lucy that was what we did. He was on low losartan, we didn’t have and we were not sure if he was supposed to be on that losartan in terms of guideline.**

**Moderators:** So what of changes in priorities for your client that you see for hypertension. How has the COVID situation affected that?

**Respondent:** **That one I am not able to answer.**

**Moderator:** And what of outreaches in the facility.

**Respondent**: **How has affected what?**

**Moderator**: How COVID has affected the current situation of COVID has affected the provision of care of your hypertensive patients in the community in terms of outreaches.

**Respondent**: **in terms of what?**

**Moderator**: outreaches are doing any outreach or what is happening current?

**Respondent:** **We aren’t doing any outreaches**

**Moderator:** So we are almost done, is there anything else that you think that we have not covered about COVID situation that need to be handled?

**Respondent**: **For now I can say no.**

**Moderator**: Is there anything else you think we have not discussed about regards to hypertension that need to be handled?

**Respondent:** **Yes in terms of investigation. The only thing at our place that we are able to do in terms of hypertension is only urinalysis you know there are there are some investigation like electro ride [urea and creatinine kidney infection test we are not able to do so in terms of investigation and also when we look at the financial it doesn’t also affect the drug it affects also in terms of investigation. You find that the investigation are informed in terms of hypertension are very expensive so find that client cannot afford that.](https://acutecaretesting.org/en/articles/urea-and-creatinine-concentration-the-urea-creatinine-ratio)**

**Moderator:** Thank you very much for your time and I think we have talked in in extensive about the uncontrolled hypertension in your community and I hope that whatever you have told me will be of help to the community and all of us So thank you for your time and you have a good afternoon.

**Respondent: And also if I may ask.**

**Moderator**: yes

**Respondent**: **I** **never got the department you told me. It is what department by the way I never got?**

**Moderator**: Am working with APHRC, African population health research center.

**Respondent**: **Ok**.

**Moderators:** Thank you

**Respondent:** **you are welcome**

**…END…**
