# Supplementary material for: A socio-ecological framework examination of drivers of blood pressure control among patients with comorbidities and on treatment in two Nairobi slums; a qualitative study
Source: PLOS Glob Public Health. 2023 Mar 10;3(3):e0001625. doi: 10.1371/journal.pgph.0001625 (PMC10021823; doi:10.1371/journal.pgph.0001625)
Supplement: S2 File — (ZIP) [file pgph.0001625.s002.zip › Health Facility/VIWA_KII_HP_200630_0306.docx]

**Moderator: {Name}**

**Respondent: Health provider**

**Code: VIWA-KII-HP-200630-0306**

**Moderator**: Confirm that I have read and that you have understood the information sheet for the above study.You have had the opportunity to consider the information, ask questions and have had these answered satisfactorily.

**Respondent**: **Yes**

**Moderator**: You understand that your participation is voluntary and that you are free to withdraw at any time without giving any reason, without any of your legal rights being affected

**Respondent**: **Yes**

**Moderator**: You understand that the data collected during the study may be looked at by individuals where it is relevant to your taking part in this study.You give permission for these individuals to have access of your information

**Respondent**: **Yes**

**Moderator**: You confirm consenting to be audio recorded and you also consent to use the anonymized verbatim quotations

**Respondent:Yes**

**Moderator**: You are happy for your information to be used in future research

**Respondent**: **Yes**

**Moderator**: You agree to take part in the above study

**Respondent**: **Yes it is ok**

**Moderator**: Thank you very much for that and I am going to read to you a small information and I will head into the questions.

**Respondent**: **Ok**

**Moderator**: This community has been identified to have a high burden of uncontrolled hypertension which is leading risk factor to premature death and disability. I am trying to gather information about the provision of hypertensive care in this community particularly for patients on treatment and who have their blood pressure not under control. So I will be seeking your views on uncontrolled hypertension among those on treatment in this community and factors driving these high rates. Please tell me about hypertension care in your community.

**Respondent**: **Inmy view we teach them about nutrition, how they are supposed to conduct themselves and how they are supposed to adhere medication, but when you see various ways the pressure is not controlled. Some patients are on denial, second one they don’t adhere to drugs may be is lack of funds. Others they ignore, they come when pressure is severe.**

**Moderator:** Ok**,** whatabout the hypertensive clinic in your facility?

**Respondent: We have some days when they come but most don’t make it.Challenge is because of work, somepeople are working from Monday to Monday so they don’t manage although we allow them to come on their specific time when they are available because you can’t deny them the services. Another challenge again others when you give them certain date to come they don’t show up. Maybe they are supposed to come that date they come with excuses. We have some challenges but we have specific days but we do follow up with them.**

**Moderator:** On a normal month let’s say a month like this, how many clients do you have that are booked for the clinic?

**Respondent: Forthe pressure?**

**Moderator:** Yes

**Respondent: We have ten specific**

**Moderator:** How many days in a week do you have these clinics?

**Respondent: We normally have once a month**

**Moderator:** How do you find the specific dates to book like once a month? Like how do you find the specific day to book?

**Respondent**: **Because of COVID 19, we discourage people to come now and again to the facility. Again another thing is because these people availability is difficult when you give more days in a month they might not come so we give drugs which extend one month but for the person who is newly enrolled with pressure, that person we give twice a month or three days a month depending with the condition of that person**

**Moderator:** Are there national guidelines for hypertension that you use in your facility?

**Respondent: Wehave one but it is the old one**

**Moderator:** So could I have a copy of that I can send you my email later you send me a copy of that?

**Respondent: It is ok**

**Moderator**: Do you have patients with hypertension and other conditions?

**Respondent: Yeswe have both with hypertension and diabetic**

**Moderator:** And how do you manage these patients?

**Respondent: Whatwe do, we manage both but when a person has a severe condition we do refer.**

**Moderator:** So which are the most cases that you see mostly with hypertension**,** what other conditions apart from diabetes do you see?

**Respondent: May be they have those who are HIV positive**

**Moderator:** Mostly in your facility which ones do you see?

**Respondent: Diabetic one**

**Moderator:** For these patients we are talking about that have hypertension and other conditions, you have mentioned about hypertension and diabetes, hypertensive and HIV, are there any different guideline for these specific patients?

**Respondent: No**

**Moderator**: You use the same guideline?

**Respondent: No**, **wedon’thave**

**Moderator**: What factors are associated with good and poor blood pressure control? Let’s start with the factors that are associated with good control

**Respondent: First of all adherence of drugs, observingweight, use of family planning because use of hormonal family planning will affect the pressure, another thing is about nutriti**on **athome**

**Moderator:** Anything else you think you have left out in the good, you have talked about adherence, exercise, family planning on women and nutrition?

**Respondent:** Again the environment, you might find in a family is staying with a man who have conflict or war. It can make a person, can affect

**Moderator:** You mean stress or something like that?

**Respondent:** **Yes**

**Moderator:** What factors do you think are associated with poor blood pressure control?

**Respondent:** is opposite, poor adherence of drugs other use herbal for medication and other family planning. When you tell a person not to use this family planning method

**Moderator**: Which one specifically are you talking about?

**Respondent:** **Like depo**

**Moderator:** Depo, meaning?

**Respondent: Deporovela. The ones on pressure have to use the IUCD, devices but they don’t want that one**

**Moderator:** In your views, what are the reasons as to why they refuse IUCDs that you talked about?

**Respondent: I think is the myths. Others say that that thing can disappear in the body**

**Moderator:** You have talked about poor adherence, herbalmedicine. Have you ever found clients in your clinic that are on treatment and still on herbal medicine?

**Respondent:** **Yes**

**Moderator:** How do you handle that?

**Respondent: I try to talk to them and counsel them, I tell them the disadvantages of using those**

**Moderator:** How do you find their blood pressure mostly when on herbal medication and they are on treatment?

**Respondent: Remember those people come when their blood pressure is high, things when are worse and takes time for patients to disclose they are not using medication maybe they are using herbal or other things**

**Moderator:** What challenges do you encounter in the provision of the hypertension care services you provide to your patients with uncontrolled hypertension? You had mentioned there is poor adherence; some patients don’t come for clinic when you book them and also others are ondenial. Is there anything else you want to add?

**Respondent:** **For people in denial is difficult to follow up because we are in slum area, even other people don’t have phones and so tracing them is difficult**

**Moderator:** Do you have any other challenge related to facility working hours?

**Respondent:** **We don’t have a challenge as I told you earlier; sometimes others don’t get permission from where they are working.Thats why we give them room for them to come at their effective time and date**

**Moderator:** At your facility, how are working hours?

**Respondent: We work 24 hours**

**Moderator:** What are challenges that you are facing with patients in regards to blood pressure control, you talked about ignorance, denial, nutrition, lack of adherence. Anything else you think that would really effect on blood pressure control?

**Respondent: No**

**Moderator:** Are there any challenges related to medication such as stock out in your facility?

**Respondent:** **Yes sometime you might find a patient has come unfortunately we didn’t have that drug but what we normally do to avoid that ,a person is supposed to come when drugs are remaining three or four for three days. If a person comes today and we don’t have that in pharmacy, we look a way so that we can get that drug**

**Moderator:** What do you do in this case, like how do you manage to get medication for these clients on that day?

**Respondent: So when patients come He/She has three days, we take that period to organize for that patient to have those drugs**

**Moderator:** So how about capacity or workload of employees providing the care of hypertension services. Tell me about that

**Respondent: We have one clinician who deals with them during the day but for example we have others at night but we have few staff. When they come because of the time they get, some come at night, they will be seen with a certain clinician and if there is a condition that is more comprehensive then that patient is referred to the person who is entitled to do all those things.More so for example if they need tests at night or lab tests, then that patient is referred to the day staff.**

**Moderator:** Do you have any other challenges guessing that the person that have been given to talk prescribes medication at some point to clients with hypertension. Do you have any challenge when prescribing medications to clients with hypertension?

**Respondent: No we don’t have any challenge because a patient is given specific drugs in case of any changes there is a record. If any change is made you consult the person incharge of that area or refer the patient to the person incharge**.

**Moderator:** For example I have a client who has been on treatment and their blood pressure isn’t controlled and the have come to the facility and their blood pressure is still not controlled, do you have any challenge in changing prescription or increasing the strength?

**Respondent:** No

**Moderator:** You have talked about consultation, prescription. Whom do you contact for example at night?

**Respondent: We have a clinical officer**

**Moderator:** Working in the same facility?

**Respondent: Yeah**

**Moderator:** So there are factors we are going to talk in different levels, what are the factors that contribute to uncontrolled hypertension in the patients you see from the patient perspective? I think we had talked about these earlier, I will remind you a few and you will add a few factors that contribute to hypertension on the patients you see. We had talked on the patient perspective that some are on denial, some don’t adhere to drugs, some don’t come to clinic and some are on herbal medicine when on medication that you had given them, you also mentioned that some of them have stress. Anything else you will want to add?

**Respondent: No**

**Moderator:** On the second level from community and family level perspective, what do you think would be a factor that contributes to uncontrolled hypertension?

**Respondent: I can say is the denial because some of them they don’t accept at all and also herbal because others have myths that if you take ABCD you should. For example if you take lemons or those ABCD they can heal. Those are the things I can say**

**Moderator:** From the providers perspective, what factors that contribute to uncontrolled hypertension?

**Respondent: Is adherence again and other underlying conditions likediabetes, if the patient is stressed, lack of funds because if the patient doesn’t have money He/She might not get drugs he/she is supposed to get**

**Moderator:** From the provider’s perspective what I meant is you as the health provider, what factors that lead to uncontrolled hypertension in the clients you see or what do you think are the things that affect you leading to hypertension not being controlled

**Respondent: Maybe not giving right drug, maybe you have given under dose, it might cause that one, and other thing is failure to be keen on follow ups on client**

**Moderator:** From health system level perspective, what do you think has contributed to these uncontrolled hypertension? I mean hospital level from the whole system, what do you think would lead to this high uncontrolled pressure

**Respondent: Maybe the Prices, lack of follow ups, maybe changing of staffs might cause that one and stock out of drugs**

**Interviewer:**So now from the policy level perspective I mean by people who give us guideline and people who make these guidelines and the rules that we are supposed to follow as facility. What do you think from that perspective would be able to have an effect on uncontrolled high blood pressure?

**Respondent: One, lack of updates, availability of the guideline**

**Moderator:** You mean in-availability?

**Respondent: Yes**

**Moderator:** Anything else?

**Respondent: Again assistance of materials and some drugs**

**Moderator:** So we have talked about so many levels of perspectives that are leading to uncontrolled hypertension so I would like to get the possible solution of each level. I am going to tell you whatever we have talked about and you are going to give me the possible solutions to the challenges that you have mentioned. We started with the individual perspective and talked on clients in denial, some not adhering to drugs and you have also talked about lack of funds and ignorance, someof them not being able to come to the facility, what do you think are the possible solutions to these?

**Respondent:I think one, community to use Community health workers to have link with the community so that this issue of adherence can be sorted out and tracking of patient can be sorted**

**Moderator:** You talked about patient being on denial, how do you think?

**Respondent: Using community health worker to counsel them because person on denial can’tappear to hospital. So using that link you can get these patients easily and you continue talking to that patient until the patient accepts**

**Moderator:** On issue of finance, what do you think we would be able to do on that?

**Respondent: For finances, the prices are supposed to be looked at and reviewed so they can afford. For the patients who can not afford drugs for the whole month, they can be given for three days or five or ten days**

**Moderator:** Family level perspective we talked about family being on denial, herbal medication used at the same time when using medication from facility and you also talked about some of them havingmyths that the hypertension would go if they take concoctions. What do you think would be possible solution to that?

**Respondent: For me I can go back to Community health worker because they are staying with these people in the community and they tend to trust these people, so if you talk to them and they go link them in the facility I think we can solve this, again about herbal they are supposed to be told disadvantages of those drugs so that when a person is using the herbal medication she knows the side effects of that medication because the people that give them the herbal medication they are not telling them the side effects, they are only telling them the advantages**

**Moderator:** From the provider perspective you talked about some people who are prescribing and not giving the right drugs and not keen on follow up, what do you think ?

**Respondent: Updates to be done and continuous updates and findings**

**Moderator:** From the health systems you talked about prices on medication on the higher side and lack of follow ups and change of staffs members and stock out of drugs. What do you think would be possible solution?

**Respondent: Isto make a budget, know the number of clients you have and after that you budget for those people, another thing …(Not clear) should be looked at, you give maybe a questionnaire so that this patients can give their feelings about the clinic then you come up with solutions. If they say about the prices then go and check the prices so that everyone can be comfortable**

**Moderator:** From the policy level you talked about lack of updates and in availability of guidelines and no materials to use and some drugs not able to reach your facility. What do you think would be a possible solution?

**Respondent: To give updates**

**Moderator:** So everyone is talking about Covid -19, it has affected the world, Kenya and affected us in a great way. How has the current COVID-19 situation affected your provision of care to hypertensive patients in this community?

**Respondent: Due to Covid-19 people are fearing to go to hospitals because they may get a patient who is infected so they don’t come. Another way what they normally do because they have taken medication for long so they know the drug they decide to go to chemist and ask for medication. It means the number has declined because of the COVID**

**Moderator:** You have had said that you have booking of ten clients in your facility, currentlylet’s say from the month of March to now how many clients have you been seeing roughly?

**Respondent: There is a dropout, sometimes for example last month was six and when you try to do follow up some are at home because of the curfew others don’t know where they are**

.**Moderator:** Are you unable to reach them?

**Respondent: Others their phones are not working, you know the problems we have in this slum us that a person can give you this number this time and that number is not working**

**Moderator:** Has the COVID situation affected hours of operation in your facility?

**Respondent: Sometime a shift was starting from 8-6, we changed to 7-5**

**Moderator:** let’s say for example today you are having a clinic for hypertensive, has it been rescheduled or what has happened on clients on hypertension? You still book them the same day and see them through the stipulated time?

**Respondent: Yes**

**Moderator:** How has COVID affected availability of antihypertensive medication?

**Respondent: It hasn’t affected**

**Moderator:** What about change in priorities on patients that you see with the current situation?

**Respondent: Isthe same**

**Moderator:** Any outreaches you are holding for these clients with hypertensive issue in your facility?

**Respondent: No**

**Moderator:** Is there anything you feel we have not talked about COVID situation with hypertensive clients in your community?

**Respondent: No**

**Moderator:** We have talked about hours of operation being changed and it hasn’t affected availability of antihypertensive medication and you talked about patients not coming for clinics most of them and booked you also talked about there is no change in priorities from clients you see and there is no outreaches you are holding at the moment, anything else apart from that?

**Respondent: Nothing**

**Moderator:** On to the last question, is there anything else you feel you have not talked about in regards to hypertension and not mentioned?

**Respondent:Ithink I have talked about all of them**

**Moderator:** Thank you very much for your time and I hope whatever the information you have given will reach people who should hear it and it will be able to make change for patients and community at large who have this problem of hypertension. Thank you very much for your time someone will get back to you much later.

**Respondent: Thank you**

**Moderator:** Have a good day.

**…END…**
