# Supplementary material for: A socio-ecological framework examination of drivers of blood pressure control among patients with comorbidities and on treatment in two Nairobi slums; a qualitative study
Source: PLOS Glob Public Health. 2023 Mar 10;3(3):e0001625. doi: 10.1371/journal.pgph.0001625 (PMC10021823; doi:10.1371/journal.pgph.0001625)
Supplement: S2 File — (ZIP) [file pgph.0001625.s002.zip › Health Facility/VIWA_KII_HP_200701_0043.docx]

**Moderator: {Name}**

**Respondent: Health Provider**

**Code: VIWA_KII_HP_200701_0043.**

**Moderator:** Confirm that I have read to you the information sheet about the study and I have given you and opportunity to asks questions and your questions have been answered you to your satisfactory

**Respondent: Yes I confirm**

**Moderator:** You understand that your participation is voluntary and you are free to withdraw from the study without any of your legal rights being affected

**Respondent: Yes**

**Moderator:** You understand that the data collected during the study may be looked at by individuals where it is relevant and you also give permission for these individuals to access your data

**Respondent: Yes**

**Moderator:** You confirm consenting to be audio recorded and you also quoted using anonymized verbatim quotations?

**Respondent: Yes**

**Moderator:** You are happy for your data to be used in future research

**Respondent: Yes**

**Moderator:** And you also agree to take part in the study

**Respondent: Yes**

**Moderator:** Ok, am going to read to you a small statement then we head direct to the questions. So this community has been identified to have a high burden of uncontrolled hypertension which is a leading factor to premature deaths and disability so am trying to gather information about the provision of hypertension care in the community particularly to patients on treatment and who have blood pressure not under control. I’ll be seeking your views about hypertension among those on treatment in this community and factors that are driving to these high rates, So in your view, kindly tell me about hypertension in your community

**Respondent: Hypertension is very common so we give patients their review dates depending on their blood pressure how it is after checking it if it is high, is it coming to normal? So it depends with that whether the patient will be coming after one week or after one month or after 3 months. We have drugs sometimes and sometimes we don’t have them and so when we don’t have drugs some patients they won’t buy and when they come back it’s very high, you try to normalize it but even after doing that the patients says that he or she doesn’t have money to buy and sometimes it makes you to refer them because it’s not going down, you refer them then when they come back you still find that it’s still not normal. Others are alcoholic; dealing with alcoholics is very tough. It’s hard to manage it for those who use alcohol and the elderly who live alone but for the young people who are working is not very hard**

**Moderator:** Kindly tell me about the hypertensive clinic that you run in your facility

**Respondent: In our facility hypertension is every Thursday and every Thursday the newly diagnosed maybe if they come and be diagnosed on Monday, we tell him to come on Thursday but if at all they are the regular patients, we book them after a month but it will be on a Thursday**

**Moderator:** Ok, so how many clinics do you have in a month?

**Respondent: In a month we have two major but at least every Thursday we have a clinic for the newly diagnosed patients because we get at least a client in every week and you ask them to come on Thursday and you need to see them at least every week to check if their blood pressure has gone back to normal but to see a large number is twice in a month**

**Moderator:** Ok. Roughly how many clients do you have booked in your facility?

**Respondent: We book 70 70, the whole group they are around 150, 160**

**Moderator:** OK, so how do you diagnose high blood pressure?

**Respondent: We take the blood pressure using the blood pressure machine and we look at the diastolic one and if its above 90, maybe a patient comes when the blood pressure is 91, 92,93,94,95, we do a serial BP check. We do serial BP check daily for five days then after the five days you check how the diastolic blood pressure is behaving and if at all it is above 90, you try to investigate by talking to the patient and if the patient is alcoholic you advise to stop, if he or she smokes, you advise to stop, you advise on weight loss, you advise on exercise, salt intake and the use of oil then you keep on checking the patient for a month and if at all there is no change you start on medication**

**Moderator:** You talked about when doing the serial BPs and you find the blood pressure is between 90-95. So where do you get this information from? Are there guidelines that you use in your facility?

**Respondent: At our facility we don’t have one but the time I went for a CME seminar that’s when we were told that you don’t start the drugs immediately, you need to do a serial blood pressure check for five days**

**Moderator:** In your facility, do you see patients with hypertension and other conditions

**Respondent: Yeah**

**Moderator:** Kindly what conditions do you see mostly in your facility?

**Respondent: The ones that go along with hypertension?**

**Moderator:** Mostly the patients you see what conditions are they presenting with apart from hypertension

**Respondent: We have diabetes, HIV, pneumonia, TB, Diarrhea, peptic ulcer diseases such**

**Moderator: so these clients you see with TB, diabetes pneumonia are there guidelines that you use for them?**

**Respondent: HIV we have a guideline, TB we have a guideline, pneumonia for the adults we don’t have a guideline but for the children we have a guideline, malaria we have a guideline**

**Moderator:** The guidelines for the other conditions you have them at the facility?

**Respondent: We have then even if it’s not all the guidelines but at least we have them. For the common diseases like malaria we have them but we don’t have for hypertension and diabetes**

**Moderator:** What factors do you think are associated with good and poor blood pressure control in your view?

**Respondent: One is the one that I have told you. If at all the patient is alcoholic it’s very hard since he or she can’t stop taking alcohol immediately. The other one, for now we have drugs but when a patient is diagnosed with blood pressure they ask you will I buy food for my kids or antihypertensive drugs so that one is another challenge, the other one is the old age. Explaining to that old woman that she is required to take medicine daily because her pressure is high, there are those who will not even remember their return date when they are booked. So that is hectic and when you ask her to come with a guardian or a treatment buddy she will tell you that they are working and they don’t have time to come**

**Moderator:** The things that you have told me are about the poor blood pressure control, what do you think are factors that might lead to good blood pressure control?

**Respondent: If at all the patient can agree to stop taking alcohol, we have those who accept and say that they will not take alcohol again, for those one the pressure normalizes without problem, If at all an old lady comes and you ask her to bring along the her guardian and she agrees to bring them and they agree that they stay together and she will be giving her the medicine as you have instructed, she will be attending her clinics, if they do the investigations like the ones that are supposed to be done after every six months then it becomes nice but if the patient comes alone and she is old, then there is nothing that you can do there**

**Moderator:** Ok. On to the next question. What are the challenges you encounter while providing hypertensive care services to the patients with uncontrolled hypertension. You have talked about some of them being alcoholics; you have talked about change of priorities now that some parents are saying that they have to buy food for their children instead of them buying the drugs and you have also mentioned some of them being elderly and not being able to come to the clinic by themselves. What are the other challenges that you encounter

**Respondent: Another challenge is our laboratory is not well equipped to test other things like urethritis and you know hypertension causes kidney failure so we are supposed to do it after six months. Without that lab here, you send a patient tom Mbagathi or Mama Lucy; they will come and tell you they were asked to pay 1600 shillings which they don’t have and they say that they will do the tests when they get money. As a health provider, you will keep on telling they for two years and the patient will never do that and maybe their pressure is always high, today is low, tomorrow is high. It keeps on changing. So lack of equipment is another thing. For the drugs, sometimes you have them, sometimes you don’t have them. Sometimes you can even go for six months without any drug**

**Moderator:** Currently do you have any supply of the drugs?

**Respondent: Yes, we have them. As per today we have them**

**Moderator:** Are there any challenges related to facility working hours in your clinic

**Respondent: No, facility working hours we come at 8:30 and we leave at 5:00. So we have a plan. They come at 8:00, we start at 8:30 and by around 12:00 or 1:00 they are gone. That one is not a challenge**

**Moderator:** Any challenges related to medication such as stock outs in your facility?

**Respondent: Yeah, just like I told you. Sometimes we have medicine sometimes we don’t have but from last year August we have not had an issue of stock out though we didn’t get all the drugs but we have some**

**Moderator:** How about the capacity or workload of the employees providing this care?

**Respondent: For staffs we don’t have them, I am alone. Only one clinical officer attending to them and attending to other clients**

**Moderator:** So when you are not at the clinic that day, what happens?

**Respondent: They are not seen**

**Moderator:** They wait for you?

**Respondent: They will come the other Thursday**

**Moderator:** Ok. Do you have other challenges you face while prescribing drugs to patients with hypertension

**Respondent: It becomes a challenge when you prescribe and maybe the medicine is not available here. Maybe you wanted the patient to use 10mg and she is given 5mg and instructed to take 2 tablets at a go at the chemist shop yet the patient is used to taking 1*1 and so when you meet him or her she will tell you that she decided to be taking just one like she is used to but now you see that’s an under dose? That’s the challenge there but now as time goes you inform them that there is 5mg and 10mg so that when she is given 10mg she knows that she is supposed to take 1*1 and when she is given 5mg drug she is supposed to take 2**

**Moderator:** So these patients that you see with hypertension, do you see any challenge while changing their prescription, the ones who are already on medication

**Respondent: Yes, but it’s not a big challenge coz when you change you tell the patient I have changed them and if he or she goes when they are not in our facility and the but in a chemist, other come back to confirm if the ones they bought are the ones that are required off but others don’t come back to confirm, I will just meet them when they come for the other clinic**

**Moderator:** What are the factors that contribute to uncontrolled hypertension to the patients that you see? We are going to talk on different levels and the first level will be the individual level or the patients level perspective. You have talked about so many previously. You talked about alcoholism, like some of them are alcoholic, some of them change of priorities, some are elderly, and some don’t take whatever you tell them very seriously. So anything else

**Respondent: Ignorance, they say that that disease is meant for the old people. By the way do you know that we have the young aged around 20, 30 years with hypertension? They say it’s a lie, they say that the disease is meant for the old people. They even stop coming here and start going to other places but finally they come back and they tell you that they have decided to start medication**

**Moderator:** So roughly what age group do you see mostly that have hypertension?

**Respondent: Mostly it’s between 30 and 80**

**Moderator:** Ok. From the community or family level perspective, what do you think are the problems that lead to this uncontrolled hypertension?

**Respondent: From the family we can say that there is kind of stress. Stress, maybe people are very busy that they forget about their parents, others forget about themselves because they are very busy looking for money, another reason could be that they are just tired of taking this drugs daily. They even tell us and ask if they can skip a day**

**Moderator:** They feel burdened

**Respondent: Yeah, the other thing is lack of support. Some families don’t support their loved ones because you can see an elderly person who has kids but she can’t be brought for medication. From the community we can say lack of information the same as ignorance**

**Moderator:** So from the providers’ perspective, what do you think are the factors that lead to uncontrolled hypertension?

**Respondent: From me I think staffing coz if am not around then they will not go home with drugs, the patient will have to leave their other duties to come here again and they will get tired. So staffing, testing equipment. That’s all**

**Moderator:** Ok. From the health system level, what do you think are the factors?

**Respondent: From health system let them provide drugs. Let them provide equipment and drugs**

**Moderator:** From the policy level, what do you think are the factors?

**Respondent: They should bring guidelines and policies. What else did you ask about?**

**Moderator:** We had talked about health system

**Respondent: For the health system let them train the community**

**Moderator:** Ok. Policy level, anything else you would like to add? You had said guidelines and?

**Respondent: And policies**

**Moderator:** Alright, in your view, we are going to go back with the same method on the possible solutions that you think are going to help with this hypertension. On the individual level you talked about alcoholics, change of priorities among the clients you see, they decide to buy food instead of buying drugs, you’ve talked about elderly who come to clinic and they are alone and you are unable to give them information and them to remember their clinics and about ignorance, What do you think are solution on that side

**Respondent: Counseling, maybe through counseling you will be able to educate them more**

**Moderator:** Yeah, anything else?

**Respondent: No**

**Moderator:** From the family level you talked about some of them having stress, some of them having busy schedules to come for the clinics and you also talked about some of them having poll burden issues and lack of support from their families, what do you think are the possible solutions

**Respondent: counseling and let them know about the treatment buddy. One of the family members is supposed to come with the patient for them to know how they are supposed to take care of that patient. For the patients, counseling can also alleviate stress**

**Moderator:** From the providers’ perspective, what do you think are the possible solutions? You talked about staffing and lack of equipment for you to do some tests at your facility, so what do you think are the possible solutions to that

**Respondent: The government to provide staff and also to give people seminars. Regular ones. Yeah**

**Moderator:** Health system you also talked about lack of staff and training, equipment not being there and sometime you also have shortage of drugs. What do you think we could do to alleviate that?

**Respondent: They should provide the equipment and drugs**

**Moderator:** Policy level you also talked about, you mentioned that your facility doesn’t have guidelines and also some policies are not being put down in your facility. What do you think we could do for that?

**Respondent: They should make sure that every facility has a guideline**

**Moderator:** Ok, we are almost done with the interview. On to the next question, everyone is talking about COVID and the whole world country is shaken with the COVID 19. How has the COVID situation affected your provision of service to patients with hypertension in your community?

**Respondent: They have fear, the don’t come but we haven’t lost one. We only lost one of diabetes but for the hypertension we haven’t lost one but they don’t come regularly like before and when they come they always ask us to hurry up for them to leave**

**Moderator:** They are afraid of coming to the hospital?

**Respondent: Yeah, they have panic**

**Moderator:** And how has it affected the hours of operation in your facility

**Respondent: We come at 8:30 but at 3:00 we are closed**

**Moderator:** You mean by that time you have seen all the clients

**Respondent: Yeah, I normally attend to all of them**

**Moderator:** So how about availability of anti-hypertensive drugs. How has COVID 19 affected that?

**Respondent: We have all the drugs; they also brought another bunch last week**

**Moderator:** What about outreaches

**Respondent: From may we have been supported to do six outreaches by Red Cross. They supported for six week but now it had stopped**

**Moderator:** Are these outreaches for hypertension specifically or it’s general?

**Respondent: It was general but we were able to capture some around 6 or 7**

**Moderator:** And how about change of priorities from the patients perspective. How are you finding that?

**Respondent: As per the COVID?**

**Moderator:** Yeah

**Respondent: Patients have decided not to come to the hospital; they think that at the hospitals is where CORONA is but I think as per now, like today I have seen many returning but they are saying that there is no CORONA because they have not died**

**Moderator:** So they don’t believe that the situation is there

**Respondent: Yeah**

**Moderator:** Is there anything that you feel that we have not talked about concerning COVID ab hypertensive clients

**Respondent: I don’t think there is any but to the working class, you know now they are not going, they are not working like they used to work before so they are still complaining. There is another one who was diagnosed with hypertension for the last two years and he is 37 years old. He decided to say the truth that he was not using medicine and when we told him that we should do a series blood check, he told me that he has been told the same for the last two years my blood pressure has always been high but even if I start medication now, where will I be getting money yet there is no any? They are not working**

**Moderator:** So it has been a big challenge, yeah

**Respondent: Yeah**

**Moderator:** On to the last question. Is there anything else we have not talked about in regards to hypertension that you feel that we need to talk about?

**Respondent: As per hypertension, it is on the rise, every week we must attend to a patient with high blood pressure, every week there must be a new client so it is on the rise. Maybe its stress, we also fail to understand and the patients’ are of age 30 and 40. Initially we used to know that it is for the old, so this hypertension seems to be on the rise**

**Moderator:** Ok. Thank you so much for your time and I appreciate the information that you have given me and I hope that it will reach the people who should hear it and will make changes and improve on whatever we are supposed to do

**Respondent: Ok**

**Moderator:** Thank you

**…END…**
