# Supplementary material for: A socio-ecological framework examination of drivers of blood pressure control among patients with comorbidities and on treatment in two Nairobi slums; a qualitative study
Source: PLOS Glob Public Health. 2023 Mar 10;3(3):e0001625. doi: 10.1371/journal.pgph.0001625 (PMC10021823; doi:10.1371/journal.pgph.0001625)
Supplement: S2 File — (ZIP) [file pgph.0001625.s002.zip › Health Facility/VIWA_KII_HP_200703_0034.docx]

**Moderator: {Name}**

**Respondent: Health Provider**

**Code:** VIWA-KII-HP-200703_0034

**INT: So you confirm that you have read and you have understood the information of the above study and you have had the opportunity to consider information asked questions and answered to your satisfactory**

RESP: Okay, yes

**INT: You understand that your participation is voluntary and you are free to withdraw at any time without giving any reason**

RESP: Okay

**INT: And without any of your legal rights being affected**

RESP: Okay

**INT: You understand that data collected in this study will be looked at by individuals where it is relevant for you taking part in the study**

RESP: Please come again

**INT:** I’m saying, you understand that the data collected during the study **may be looked at by individuals where it is relevant to you taking part in the study…**

RESP: Okay, yeah

**INT: And you give permission to these individuals to access your data?**

RESP: Okay

**INT: And you confirm consenting to be audio recorded and you also consent to use anonymized vertabim quotations?**

RESP: Come again

**INT: You confirm consenting to be audio recorded and you also consent the use of anonymized verbatim quotations**

RESP: Yes

**INT: And you are happy for your data to be used in future research?**

RESP: My data?

**INT: Yes, to be used in future research**

RESP: Data, you mean the information you will get from me?

**INT: Yes**

RESP: Yeah, okay yes

**INT: And finally you agree to take part in the above study?**

RESP: Okay yes

**INT: So I’m going to read to you a small statement and then we’ll head right into the quesions**

RESP: Okay

**INT: This community has been identified to have a high burden of uncontrolled hypertension which is a leading risk factor to premature death and …**

RESP: Talk a bit louder, I have a hearing problem, my phone has a problem

**INT: Okay, so I am saying this community has been identified to have a high burden of uncontrolled hypertension which is a leading risk factor to premature death and disability. So I’m trying to gather information about the provision of hypertensive care in the community particularly to patients on treatment who have their blood pressure not under control. So I’ll be seeking your views on uncontrolled hypertension among those on treatment in the community and the factors that are driving to these high rates**

RESP: Okay

**INT: So, in your view kindly tell me about the hypertensive care in the community**

RESP: The hypertensive care in the community is good but there are some challenges. Sometimes there are stock outs in the facility that affects the clients so much, if they know that there are no medications they don’t come for them. Another thing is alcoholism, it also affects because they forget their TCAs and the clinic days are steady but you find that you can give a patient some appointment but if he or she still has some drugs at home she will not come for the appointment, she’ll tell you “I still had medicine”. And then the age also, you know most of them are 50’s and above, so the age challenge

**INT: Okay, so tell me about the hypertensive clinic that you have in your facility**

RESP: Pardon

**INT: Tell me about the hypertensive clinic that you have in your facility**

RESP: We usually have hypertensive clinics every Thursday and two major ones twice a month

**INT: Which days are these?**

RESP: Mostly on Thursdays, the doctors will spread you but every Thursday we have HCP* clinics and then two major ones in a month

**INT: Okay so kindly tell me last week on…Thursday was yesterday. Kindly run me through the clinic day how it was yesterday**

RESP: Yesterday?

**INT: Yeah yesterday was Thursday**

RESP: You want the number of patients?

**INT: Hmm just generally tell me how the clinic runs from morning till when it finishes**

RESP: On a clinic day the clients come at around 8.00am then they are seen, they are given their medications and after that they go back home

**INT: Okay so how do you diagnose high blood pressure from your facility?**

RESP: We screen all the patients coming to the clinic

**INT: Okay by screening how do you do that?**

RESP: The patients who come to the clinic who are sick we have to take their vitals

**INT: Vitals meaning?**

RESP: Blood pressure, temperature…that’s where you can find the new cases through screening

**INT: Okay and in your facility are there any national guidelines for hypertension that you use?**

RESP: No

**INT: So what do you use at your facility to diagnose these hypertension cases?**

RESP: To diagnose?

**INT: Yes**

RESP: The history of the patient, blood pressure machines, the (unclear 00:06:19)

**INT: Okay so do you see any patients with hypertension and other conditions in your facility?**

RESP: Yeah

**INT: What types of conditions are these?**

RESP: Mostly diabetics and in rare cases pregnant mothers and those who just come with other conditions when checked you find out that they also have hypertension

**INT: Okay so these patients that you are telling me have diabetes and hypertension and the pregnant women that you are telling me that some of them you find that they have hypertension, how do you manage these patients?**

RESP: They are managed at the clinic but in severe cases especially the ANC mothers…

**INT: ANC meaning?**

RESP: The pregnant ones we refer them to Mama Lucy because we don’t have the wards here especially mothers developing pre-eclampsia

**INT: Okay and are there any guidelines that you are using to these specific patients with hypertension and other conditions?**

RESP: Pardon

**INT: Are there any guidelines you are using to manage these patients with hypertension and other conditions?**

RESP: Other conditions?

**INT: Yeah you’ve said there are patients you see that have diabetes and hypertension and pregnant women with hypertension. Are there any guidelines that you use, specific guidelines for these patients with other conditions that you are using in your facility?**

RESP: There’s no specific guidelines but there is this book called ‘*Daily Activity Register for Diabetic and Hypertension Patients*’ that one guides us because it has a place for for example, TB screening

**INT: Okay and kindly tell me about the factors that are associated with good blood pressure control**

RESP: Factors that are associated with good blood pressure control?

**INT: Yes**

RESP: Health education on high blood pressure, the diet, follow up

**INT: Okay, anything else?**

RESP: Monitoring them every now and then and creating awareness in the community through health education

**INT: Okay and what do you think are the factors that are associated with poor blood pressure control?**

RESP: Unavailability of drugs in the facility sometimes because when there are no drugs they don’t come aside from that some clients are alcoholic and cannot follow up. Others like young people if they have high blood pressure they tend to be desperation they tell you “will I be able to take all these drugs throughout my lifetime?” The fear of taking drugs

**INT: Okay and in your facility what kinds of challenges…**

RESP: Another, you know blood pressure is never associated with pain so the client will tell you “why should I take drugs and I am okay I am just feeling a little headache, such things

**INT: Okay in your facility do you have any challenges that you encounter in provision of care?**

RESP: Pardon

**INT: Are there any challenges that you encounter in provision of hypertensive care services to the patients with uncontrolled hypertension?**

RESP: Challenges?

**INT: Yes, you talked about stock out in the facility, you talked about some of them forget their clinic days and you also talked about some of them being alcoholic. Is there anything else that you have a challenge with these patients with uncontrolled high blood pressure**

RESP: No, apart from the one you have mentioned maybe sometimes staff

**INT: Okay what about the staff?**

RESP: Sometimes we are so few

**INT: Okay and what challenges are there…are you facing with patients with regard to generally blood pressure control in your facility?**

RESP: Challenges?

**INT: Yes**

RESP: Come again

**INT: What are the challenges you are facing with your patients in regard to…**

RESP: Let me just say challenges is only with few patients who don’t want to follow instructions, the attitude of the client sometimes like this one asking you “I cannot take drugs throughout my life and I’m not feeling bad” or “I don’t want to be put on drugs” such minor minor arguments

**INT: Okay and are there any challenges related to the facility working hours?**

RESP: No

**INT: What time do you work from?**

RESP: The challenges is with…sometimes our clinical officer has great challenges for example now she is the only one in the clinic. One clinical officer…on a clinic day for example if it’s a major clinic day we can see up to 70 clients coming for PMH so that comes to challenges of sometimes staffing. (*Chuckles*) but that is beyond this

**INT: Okay and do you have…you’ve told me that you have a clinician who does prescribing of medications and seeing the clients who come at your clinic. Do you have challenges when prescribing medications to the patients with hypertension?**

RESP: Now the clinical officer?

**INT: Whoever prescribes the drugs to the patients**

RESP: No, she doesn’t

**INT: Okay so these clients come to the facility and they get their drugs and the prescriptions are okay?**

RESP: Yeah

**INT: Okay so any problem with capacity or workload of employees providing the care of hypertension?**

RESP: Pardon

**INT: I’m asking are there any challenges in the capacity or workload of employees who are providing the care of hypertension?**

RESP: Just some minor

**INT: Which is?**

RESP: Hmm?

**INT: Which is?**

RESP: The ones I have said about

**INT: Okay that’s fine, so we are going to talk about factors that are contributing to uncontrolled hypertension in the patients that you see but we are …**

RESP: Factors?

**INT: Factors that contribute to uncontrolled hypertension in the patients that you see but we are going to talk about it in different perspectives. So the first one is the patient or individual perspective like how the patient feels about being diagnosed with uncontrolled hypertension. So what are the factors that contribute to this uncontrolled hypertension among the patients? You’ve talked about alcoholism, you’ve talked about age factor, and you also talked about some of them having desperation asking if they’ll take their drugs for a lifetime and you also talked about some of them forgetting their clinic days. Anything else you’d like to add to that?**

RESP: Nutrition, you see most of our NCD clients are people…are elderly. Most of them are not working so also poverty, sometimes when there are no drugs in the hospital if you prescribe for them to go and buy they never buy they’ll keep on coming to you asking “have the medications come sister?”

**INT: Okay and from the community and family level perspective what do you think are the factors contributing to uncontrolled hypertension?**

RESP: Maybe some are…lack of knowledge. Lack of knowledge

**INT: Anything else?**

RESP: And poverty considering the population of those who are…

**INT: Hello**

RESP: Hello

**INT: You were saying something**

RESP: I said social economic factors considering that most of them are elderly and unemployed

**INT: Okay, from the providers’ perspective, from your perspective now what do you think are the factors that are leading to uncontrolled hypertension**

RESP: From?

**INT: Your perspective, the providers’ perspective**

RESP: I have said it, maybe if they default or …am I understanding the question well?

**INT: I’m saying, we are talking about the factors that are contributing to uncontrolled hypertension in the patients that you see and we have said that we are talking about this in different perspectives, we’ve talked about the individual perspective, we’ve talked about the family level perspective and community. Now we are at the providers’ perspective, you as a health care provider, what are the factors that are leading to uncontrolled hypertension in the patients you see?**

RESP: Factors that are leading to uncontrolled hypertension?

**INT: Yes, yes**

RESP: Patients not taking the drugs well, stock-outs, patients defaulting

**INT: And from the health system level, the management and everything that includes the health system level …**

RESP: Pardon

**INT: I’m saying from the management team and the health system level what do you think are the factors that are contributing to uncontrolled hypertension?**

RESP: Sometimes unavailability of the drugs the stock outs…

**INT: Anything else?**

RESP: Inability of the…some of the…most of the patients to afford the drugs

**INT: Okay cost of drugs yeah?**

RESP: Yeah cost of drugs

**INT: And from the policy level, the people who give us these guidelines and the people from the management team, what do you think are the factors that are contributing to uncontrolled hypertension?**

RESP: Factors that the?

**INT: That are leading to uncontrolled hypertension from the policy level**

RESP: To create more awareness, give health education on management of care of patients with NCDs…

**INT: Okay so we’ve talked …**

RESP: And guidelines for trying to track if there is any change

**INT: Okay guidelines updates yeah?**

RESP: Updates yeah. Especially from my experience the education on NCDs is never rampant like the HIV, you know HIV everybody knows about it but when it comes to NCDs it seems that nobody cares as in it’s not highlighted as much. (*In Swahili*) There’s not as much education on it like those other things that are highlighted …now that corona has come it is being highlighted, or malaria but these ones for NCD there is no one who is concerned with them

**INT: Okay you are free to talk any language it’s not specifically that you speak English. The one that you feel comfortable is okay. So we’ve talked about those different levels and you’ve given me the challenges that you are encountering in different levels so we’d want to talk about the possible solutions in the same same way that we’ve talked about. So now from the individual level you talked about poverty, you talked about alcoholism you talked about nutrition- people having problems with their nutrition. So what do you think are the possible challenges that we should do…**

RESP: Pardon

**INT: What do you think are the possible solutions for these challenges that you’ve talked about?**

RESP: Solutions?

**INT: Yeah for the individual level**

RESP: It’s the government to provide enough, the policy makers to give more education to health care providers and the health care providers to give more health education to the consumers, to the patients, and the guidelines to be provided to those who are providing care

**INT: Okay and from the family level…**

RESP: Maybe if the government can make sure that the elderly get those drugs or if they can be sent to even some little money for medication or they just be put for something to make sure that they get those whatever

**INT: Okay so everyone is talking about COVID, COVID-19 the whole world, Kenya…so how has the current situation of COVID affected the provision of care for hypertensive patients in your community?**

RESP: Previously, many had been afraid but then we had the community health workers to create awareness and there were…several outreaches were carried out so they are coming, it hasn’t affected them so much. They were given health education through outreaches

**INT: And has it affected the hours of operation in your facility?**

RESP: Pardon

**INT: Has it affected the hours of operation in your facility?**

RESP: For the first three weeks of COVID…for the first one month of the COVID it affected because we used to work in shifts but it was cancelled now it is not affected. And then during that month we were giving them long TCAs so that they should not keep coming and overcrowding the place. Say TCAs of three months

**INT: And how about the availability of the medications in your facility?**

RESP: Now we have most of the drugs except one which most of them use, NoGluc. It has been out of stock for so long

**INT: For like how long now?**

RESP: Hmmm?

**INT: For how long since the last time you ordered?**

RESP: For around 4 months now

**INT: Okay and is there anything else that has been affected with COVID and we’ve not talked about?**

RESP: No

**INT: Okay, so on to the last question is there anything else you want to talk about in regards to hypertension and we’ve not discussed in this topic?**

RESP: Pardon

**INT: Is there anything else you want to talk about in regards to hypertension and you feel we’ve not talked about it?**

RESP: No

**INT: Okay thank you very much for your time and someone will get back to you later and I hope the information you’ve given me will be able to reach…**

RESP: Pardon

**INT: I hope the information that the information you’ve given me will be able to reach the people who should hear it and we make our guidelines be as straight as possible to help the community and the country at large. Okay thank you so much for your time**

RESP: Thank you

…End…
