# Supplementary material for: A socio-ecological framework examination of drivers of blood pressure control among patients with comorbidities and on treatment in two Nairobi slums; a qualitative study
Source: PLOS Glob Public Health. 2023 Mar 10;3(3):e0001625. doi: 10.1371/journal.pgph.0001625 (PMC10021823; doi:10.1371/journal.pgph.0001625)
Supplement: S2 File — (ZIP) [file pgph.0001625.s002.zip › Health Facility/VIWA-KII-HP-200701-0035, 0426, 0425.docx]

**Moderator: {Name}**

**Respondent: Health care provider Viwandani**

**Code: VIWA-KII-HP-200701-0035, 0426, 0425**

**Moderator:** So confirm that I have read and that you have understood the information sheet from the above study and you have had the opportunity to consider the information, ask question and you have been answered to your satisfactory

**Respondent: Yes it is confirmed, I have read it in the question**

**Moderator:** Ok, you understand that your participation is voluntary and you are free to withdraw anytime without giving any reason and none of your legal rights will be affected.

**Respondent: Ok, I understand that**

**Moderator:** You understand that the data being collected during the study might be looked at any individual where it is relevant in taking part in this study and also agree that the permission you are giving to this individual to access your data

**Respondent: I confirm**

**Moderator:** You confirm consenting to the audio recorded and also consent the use of anonymized verbatim quotations.

**Respondent: No problem with that**

**Moderator:** And you are happy that your data may be used in future research

**Respondent: That’s nice**

**Moderator:** And then lastly you agree to take part in the above study?

**Respondent: Yea**

**Moderator:** Ok I am going to read to you small information about the study again and then we head right to the questions

**Respondent: Ok**

**Moderator:** This community has been identified to have a high burden of uncontrolled hypertension which is a leading risk factor to premature death and disability. So I am trying to gather information about the provision of hypertensive care in the community particular to patients with high blood pressure not under control, so I will be seeking your views on uncontrolled hypertension basically to patients who are on treatment and factors that are driving to this high rate.

**Respondent: Ok**

**Moderator:** Yeah, so unto the first question tell me about hypertensive in the community

**Respondent: Pardon, what say about the hypertension?**

**Moderator:** I would request you to get a place which is a bit quiet so you can be able to hear me well.

**Respondent: I am really training where I am working in “kijiji” so I am near the residents, so I am really trying to get a single room where I can be alone**

**Moderator**: Ok, I will try to be audible enough

**Respondent: Yeah try that please**

**Moderator:** So please tell me about the hypertension care in the community

**Respondent: Hypertension care in the community first I think as per our clinic, like our clinic is more based in the community and I think we encounter some patients coming to our clinic with hypertension on taking history most of them are brought about by poverty. Most of them either directly or indirectly but there are some which are being caused by things like diet but the most is poverty where some history cause I recall there is a record we have. Very minimal cases we have for the pregnant mothers in labor like maternity cases but most of them disappear maybe one or two cases tends to persist for around six months but when taken well care of disappears.**

**Moderator:** Ok

**Respondent: Yes**

**Moderator:** So at the moment you have mentioned that you work at a health facility, please tell me about hypertensive clinic in the facility you work in

**Respondent: The clinic I don’t understand the question well**

**Moderator:** I want to get to know if how hypertensive clinic in your facility works

**Respondent: Ooh, how it works**

**Moderator:** yes

**Respondent: Ok in our facility we have outpatients and mostly is done by CO and most of weekends we don’t have CO so it is the nurses and sometime I get to be called and actually I am the nurse in charge and most of the time when we have difficult sometime I chip in to assist so like generally it is managed by CO from Monday to Friday, weekend we have nurses but in case of anything in I am called to chip in for assist**

**Moderator:** Ok, so do you have a specific day for hypertensive clinic in your facility?

**Respondent: Not really, we don’t have specific day for hypertension, everyday anytime they come in we look at them because we don’t have a file for this hypertensive client that we are following. So once a client comes in the first thing and the triage department we refer to take the pressure, the BP and once we realize the BP are high we tend to take this patient under care for 2, not two weeks. When monitoring the BP like daily monitoring drug like------05:51----------- so if the BP tends to respond to medication we declare this this patient not hypertensive but if it persists then we have to refer this kind of patient to a well facility where there are hypertensive patients for other checkups and follow up.**

**Moderator:** Ok, now you have talked about you don’t have specific day for clinic they walk in as you see them and depending on their severity is when you refer and see how to manage them so how do you confirm this patient has hypertension?

**Respondent: We confirm by taking blood pressure with BP machine so when the BP pressure we checked systolic and diastolic. When the systolic is above 140 and diastolic is 91 and above so that sometime systolic tend to elevate bit immediately when a patient comes in with a different complain or condition they just come in cause we are trying by all means not to detain our client not to sit for long waiting in the waiting bay so you find that when a client comes go directly to triaging department so at time you find out the systolic pressure are bit elevated but when we realize that we tend to give them some more time like can you rest for thirty minutes then we take pressure again. So once we repeat some tends to go down some resist like they retain their BP tends to be so such clients are the ones that we start now the follow up bit like daily monitoring then we book them on those medication we have.**

**Moderator:** Ok

**Respondent: Yes**

**Moderator:** So at your facility do you have any national guideline for hypertension that you use?

**Respondent: Not really, mostly we have the MOHs like in the bigger hospitals where we can consult, we consult from them but we don’t have national guidelines that we are using currently.**

**Moderator:** So you have talked about also you see hypertensive patients and I would like to ask, do you see any patient with hypertension and any other condition?

**Respondent: Like with hypertensive and other conditions, yes**

**Moderator: Which conditions are these?**

**Respondent: Like some come with DM or diabetes so are normal conditions like typhoid, ok we have encountered with malaria but it is not that often, we have had conditions like asthmatic but in a very rare condition, encountered some patients who are obese some patient who come are obese that is. So I am just some number of conditions I might not highlight at the moment.**

**Moderator:** So these patients you are talking about have hypertension with diabetes, hypertension with typhoid, hypertension with malaria, how do you manage these patients specifically?

**Respondent: Ok, these patients who come in with hypertension for example with typhoid we have to treat the underlying cause like a patient come with hypertension and typhoid we will learn we will then physically they didn’t know that their pressure are elevated, it is something that we confirm we get or realize when we take the BP . So we need to explain to the patient that your BP pressure are not the normal one and so after having heard the complains the patient comes with we tend to explain like when the pressure are high it also cause something like headache so at time we tend to explain to them that this pressure can also lead to this kind of headache which persist or whenever you feel you are feeling are having headache you better come and check your BP. So when they come with those conditions for example I have diagnosed that this patient is typhoid and temperature has elevated. I will have to treat typhoid at the same time I will have to put this patient on hypertensive drug.**

**Moderator:** So you have said you don’t have a national guideline for hypertension in your facility, do you have a national guideline specifically to these patients who have other conditions?

**Respondent: The specific national guideline for these other condition like we have CME**

**Moderator:** CME meaning?

**Moderator: C**ontinuous Medical Assessment or education, so whenever we have CME, at time we get the lectures or there is a term her.

Moderator: The small pamphlets that you are given?

**Respondent: We get the small pamphlets at some time they are provided from the sub county that is Makadara at time most of the time whenever we had CME it is only that currently we are having that challenge of COVID 19 so we find it a bit hard to gather for the CME but we still have CME, so whenever we have CME we are updated on current guidelines on how to manage patients and then we have to ask questions. Before we used to do like this and how you are telling us to do this what has made us to do like these or what has made it change? As usual you know the medical field is dynamic everything is not constant. So most of the guidelines we use mostly we find from CME very vital for us.**

Moderator: So unto to the third question, what are the factors that are associated with good and poor blood pressure control?

**Respondent: Factors that are associated with poor?**

Moderator: Yes let’s start with poor.

**Respondent: Like when these patients I don’t know how they want me to answer this like.**

Moderator: Factors that are associated let me just read the question again probably you can get it well. So what are the factors that are associated with good and poor blood pressure control that is the question, so now I would want you to give me the factors that are associated with poor control of blood pressure?

**Respondent: For example a patient is hypertensive and is poorly controlled**

Moderator: Yes.

**Respondent: So the factors that are associated with, one is if we have is hypertensive and is poorly controlled, it can lead to cardiac arrest because of the fast beating of the heart which can lead to collapse of the patient. This patient can collapse due to that. It can also lead to factor of the veins and arteries because that is the part where we have blood running flowing through. Then sometime we can also have the water retention in the body because, if it is poorly controlled the same time I think we can also experience is that patients can lose their lives. But if it is nicely controlled.**

Moderator: Good control?

**Respondent: Good control, I mean these patients still can maintain, they can continue with his or her normal routine and nothing can help can she can continue with her normal life as used.**

Moderator: Anything else you would want to add to either the good and poor control.

**Respondent: Maybe if I remember one talk about I will let you know**

Moderator: It is fine, so unto the fourth question. What challenges do you encounter when you are providing hypertension care services to the patients that you see with uncontrolled hypertension?

**Respondent: The challenges, one is adherence to the medication. Some clients when we tend to give them a follow up date some don’t turn up and if you try to call, because we have a register where we record the name addresses, so if we try like patients that we have had the pressure era not behaving normally we tend to follow them up closely, because if it persists we need to refer these patients to a facility where she or he can get good management. So when we call most of the time they tend to answer like they are dizzy, looking for money so one is poor adherence to medication that is a very big challenge that we have been going through, some are allergic to such some type of medication so you give them these medication they tell you, oh am allergic to these, I’ve been using this and I remember once I reacted to this, so I can’t use it and then you find that in a clinic we are in we don’t have a lot of drugs have ---18:12 Think those are most what we have got, so you find that when a patient tell you he or she reacts to this you feel to now know which medication can you prescribe to this patient, so such we tend to refer and again when you are trying to refer, the patient because I Don’t know they are used to our hospital so at the same time when you try to refer the patient tell you “ooh, there am going to queue for long and if I go for like in the morning I will come back in the evening I shall have wasted a lot of time just how they may feel if you refer the patient some of them fail to go to the referral facility only to come back when pressure are too bad. So these are the some of the challenges we under go.**

Moderator: So the challenges you are facing with the patients in regards to blood pressure control, you have talked failure to go for referral saying they will queue for long some of them are in denial that they high blood pressure.

**Respondent: Some are also in denial.**

Moderator: You have talked about some of them not being able to adhere to medication, and also you have talked that sometime you have challenges with not being able to prescribe drugs because you don’t have all of them.

**Respondent: Yes we don’t have the variety and at some time, some also poverty comes in. Some because we are in private facility, they have to buy some so some with the cost some cannot afford**.

Moderator: Are there any challenges related to your working facility hours?

**Respondent: Our working facility hours challenge no. There we don’t have because, we are 24 and we work in shift, so don’t think if there is any challenge there.**

Moderator: So are there any challenges related to medication such as stock out in your facility?

**Respondent: Yea, initially we didn’t have that challenge like too much but currently because of the low turn up of the patients, so sometime and then of late we had of delay payment by NHIF because we are NHIF accredited so sometime we are so much dependent on NHIF payments sometime for our medication supply so we find out that at some time we order for less drugs compared to initial so sometime you find that the client we have because they have NHIF patients and not cash so you find that we treat , no payment so we run short of finance to order for other clients, but when we get the little that, we have we always make sure because we are trying, remember we are private so we are trying to give quality services compared to some public hospital where they go queue for long so we are trying all our best to make all our patients feel comfortable bit we have challenge currently of medication.**

Moderator: When is the last batch that you got for your medication stock?

**Respondent: The date we received?**

Moderator: Currently do you have any medication at your facility that will last you?

**Respondent: Currently we have some few stocks and actually we are making some** **plans to order next week but one**.

Moderator: So how about the capacity or workload of employees providing this care of hypertension. Do you have any challenges with that one?

**Respondent: That one we cannot fail to have. The workload is too much remember we are in slum and they come out a lot of challenges with workload because I told you it is a private and you know this employees sometime it is hectic. The staff are not enough like that is, sometime you can do work and get tired so but you have to do it because it is if not you there is no one to do and the patient has come. You have to strain to make sure your patient is served.**

Moderator: So if you have talked about most of that time during the week you usually have clinical officer at your facility who see your patient and when you are not at the facility during the weekend the nurses are there managing patients, so do you have any challenge when you are prescribing medication to patients with hypertension.

**Respondent: Not really like there I said that sometime when you are prescribing medication to hypertensive the only challenge we have you know before you prescribe the medication you to confirm with the patient” are you allergic to any kind of medication” and if yes, you have to ask which one and then you need to take history. So at time you find the kind of medication that you wanted to prescribe is falling under the drugs that he or she is allergic to and so when you try to check on other you can give that isn’t allergic you find that you are out of stock with the drug so at time it becomes an issue of you to prescribe such medication for he such unique patients.**

Moderator: So let’s say you have a client who is on medication and has come with hypertension, and you are here for change of prescription do you any challenges with that?

**Respondent: Not really because if this patient is on medication, actually then this patient might be having the card and medication that he or she is on, so if this patient wants to change the medication that is on side Don’t think if there is any problem on changing of medication just only if I don’t have that kind of drug.**

Moderator: What about the increasing of the number of the strength of the medication, do you have any challenges with that?

**Respondent: Ok there are some at time because of the severity. Depending on the severity some time when you want to know which dose should I give out and I feel like I have any challenge, because of the national guidelines that I don’t have at some time I have to call because we have the most at time we call other CO working in other hospitals so sometime if you find we call them and consult from them what is supposed to be done.**

Moderator: What are the factors that contribute to uncontrolled hypertension in patients that you see? We are going to talk about in different levels. So now from the patient’s perspective you had told me earlier about adherence, denial, allergic to medication some of them don’t have drugs for all of them, for patients that you see, you also talked about the referral system you have. Some of them talk that they queue there for long and you also talked about poverty. Anything else you would want to add to that?

**Respondent: For now I don’t think there is anything I can add**.

Moderator: From the community and family level perspective, what do you think are the factors that are contributing to uncontrolled hypertension?

**Respondent: There are some things like, you know when you are financially u stable, am now talking from the family level, when you are financially unstable you will go through a lot of challenges remember you are a mother, father and you are the bread winner of that family and at the same time you are living in Nairobi most of our clients don’t there residing places, like they dint have they homes, most of them are renting, so then there is job insecurity. Job is not that adequate that we can say that you have sure bet, so a lot of stress come in because you have to think of what are my family going to eat so like I talk of family conflict comes in you find out that somebody is talking alone , a patient come eating in the waiting bay. That you are just saying maybe a patient haven’t engaged them now that you have here a bit knowledge on psychology you will need try to get the information what could be wrong with your patient when now you are with the patient in the cracking room or consultation room that is so mostly poverty has really affected according to the population that we have at the family level and then there are some people who are hyper reactive. They react so much they, how can I put it?**

Moderator: Temperature?

**Respondent: Something like that, so you find that when this person is a kind of annoyed, in any way they tend to react too much and you know when they react too much, blood pressure tend to rise because of the heart beat which has increased and such thing. So some mostly poverty. We have stress too much is leading too that and those with hyper reaction.**

Moderator: From the providers perspectives, your perspective now what do you think are factors that are contributing to uncontrolled hypertension?

**Respondent: My perspective like, on my side now. What can lead to poorly controlled?**

Moderator: yes

**Respondent: Like as health provider if I fail my duties ,my services I don’t do them according to the way I am supposed to do because some of them negligence, there is something I ought to have done but I failed to do like, I one I have realized that I have a patient, this patient is hypertensive, I need to make sure that this patient is followed up, this patient takes medication, If there is any challenges with taking medication which can lead to poor adherence of medication I need to talk to this patient, if it is something to be referred and so you find maybe sometime if I failed to do my part ,ok I don’t see the patient alone we have a team so you find that maybe someone has seen patient for long or many patient and so this patient feels exhausted now we are human being, you feel exhausted and the patient come mostly these patient, you know we are human being and the same time as I have just said and I am dealing with a fellow human being each and every person will have his or her personality. If you find that your personality can’t ripe and the same time you are exhausted, you will find that doing something which wasn’t supposed to be done or you may just fail, take the medication you prescribe” ooh you had pressure, you prescribe medication and tell go buy the medication from the pharmacy go home, so you don’t yell these patients so that you are supposed to come for follow up, so these needs to be doing monitoring, daily monitoring .If you see it is this you have to do this .So such are the challenges that as health provider tend to have but I think we are trying our level best to be human enough.**

Moderator: from the health system level perspective what do you think are the factors that might lead to uncontrolled hypertension?

**Respondent: Like when you are hypertensive.**

Moderator: Yes

**Respondent: There is a kind of food that you are supposed, I think if I’m on the right track?**

Moderator: Health system means

**Respondent: There is some food that you are not supposed, diet that if you are hypertensive you are not supposed to eat. Like salt intake, sugar things like that. So you find that if these patients can’t the diet is not looked at, it is poor it can** **lead to poor uncontrolled hypertension, you tell a patient don’t do this you know the patient don’t understand that if they continue with their normal diet health wise it can lead to bad and other things health wise, when you are too much obese you have more chances of developing hypertensive because of the lumen of the veins of the arteries that comes in the thicken, so the narrow part of blood flow so such thing which will make the heart to pump at higher rate you know that when the heart pumps at that rapid rate it takes to elevate the blood pressure, so when you are obese, so when you don’t look at your diet well health wise such things can lead to and don’t do exercise, your work is eating meat and relaxing only, such things health wise tend to cause hypertension.**

Moderator: Now I meant from the health system level. It means that structure of the health itself. I meant by the people who give you medication, who supply you with equipment, the people who supply you with drugs. So what do you think are challenges that you will encounter that are leading to this kind of uncontrolled blood pressure from the health system?

**Respondent: That’s what you meant?**

Moderator: yes that’s what I meant

**Respondent: Sorry, I think from that you will see we are employees, we are employed and I think there is exemption from either the government facility because of the supply mostly currently as we talk patients are really running away from public facility because they say even if you are an NHIF if you are not cash patient you are told even a Panadol go buy. So at time you find that you are told to buy, you don’t have that money, the deduction are done in the NHIF, you so at times you find it a bit hard with the supply so sometime a health facility provider you prescribe a medication just the other thing I have told you and that thing is not fair or you look at hypertensive patient you say according to severity this one need this kind of HTZ wherever. So you find that according to severity you look at the patient you say this one need this kind of medication, that one you have, it is going to be a failure from the health provider person, so those are some of the challenges that at times can up from the health service provider.**

Moderator: From the policy level perspective, the people who give you the guidelines on what to follow and what to do. What do you think are the challenges that you encounter in that contribute to uncontrolled hypertension?

**Respondent: From the policy level, so far I don’t think if there is one that I can talk of from the policy level for that guideline I think you mean that.**

Moderator: yes

**Respondent: I don’t think so far we have had any challenge that can lead to control of hypertensive because I think of the calls that we have been making maybe to consult on the guidelines and everything most of them have worked for us like for me .So I don’t think If I have any challenge from there that I can talk of.**

Moderator: So those levels we have talked about I would like to get the possible solution from those challenges like for now, individual perspective you talked about adherence, you also talked about patient being in denial, others being allergic to medication other medication that you want to prescribe and you also talked about some of them having poverty, some of them are being obese and lack of proper diet and exercise, so what do you think are the possible solution to these challenges?

**Respondent: Is a bit hard, there are some challenges that are manageable but some are a bit hard like when somebody is poor, poverty you know you can’t get your money from pocket and then give every patient that come that cannot afford to buy drugs and like for the ones who are obese, adherence to the medication you will tend to get information, you will have your time with this patient. If this patient adherence to medication is not good, you will need to get as health provider you need to take time with this kind of patient, sit down get acts stories with this patient you know when you open up you create a good rapport with your patient they will tend to open up and tell you what could be the reason like for example we have patient on HIV , you know some are discordant and the same applies to these management of hypertensive, some patients you will find that they don’t want to disclose to others that I am hypertensive and maybe the spouse or any other person. So it become a bit harder for this person to be taking his or her medication because, he is trying to hide when taking this medication. At time you find that they lack that time to hide themselves so that sometime they don’t even take the drugs. Sometime the storage of medication matters a lot where the drug was stored was destroyed or something like that, it is not stored according to the standard. So there is damage to medication so when you talk to your clients if it a spouse or it is environment that may causing this patient to have this kind of hypertension, once the patient opens up to you it is now the time of the health provider person to take time maybe to get if they can manage to go visit the family, let them open up they talk but with the consent from the patient. You first need to get the consent from the patient so that you can carry on with your services.**

Moderator: From the family level perspective you talked about some of them having change of priority and thinking about their families and not their health themselves, you also talked about job insecurity for most of them and some having stress and some having temper that they are not accepting they have this kind of condition. What do you think are the possible solution for that?

**Respondent: Like this one for job insecurity, the solution for this are like once there is a job security because there problem is job insecurity.so when you create that opportunity, that job so this person is not idle ,is stress free, this person will not have kind of pressure.so this person has something to do, can generate something small maybe to sustain the family, that stress can that can cause this pressure I think will fade away at the same time if this person is highly tempered you tend to talk with this person like this person needs counseling. And during counseling you will be talking a lot of thing that leads to this person to raise them back so you train the patients on what are things to do when you see such things which can make you go through that, what can you do?, can you walk away?, do you fight to make it or when you have something disturbing your mind should you cry that you get away from it, so you look at what possible causes you treat the cause but you see now it is bit hectic like in our situation. If it is a job insecurity, we get to create a job for someone because usually when it comes money issues it is difficult so it is bit hard now to solve but you tend to walk to your patient when you have faith that it is not you alone that is going through this, many people are going through this but they are surviving, just accept the situation, do this during this time and you will get well**

**Moderator:** From the provider’s perspective you talked about negligence and some being exhausted when they are taking care of patients, what do you think would be a possible solution to that

**Respondent: Ok if I may start with exhaustion, like if you are too tired now you see the solution to that one is to make sure you are well staffed. Staffing is good that At least one can work from maybe morning to maybe noon like then another person comes is you are relieved bit you go rest wait maybe for tomorrow duties and something like that but if you see now if you come to work at 8 and leave at 6 and during this time you’ve been with patients. Every patient with their problem you know we have brains at times you get exhausted and feel like you want to sleep so stop that is to make sure that you are well staffed, you are not understaffed so the other question was about negligence?**

**Moderator:** Yeah

**Respondent: Negligence I think is all about talking with currently like when you are a leader you see when you neglect a patient they will not just walk away like that, this is something that they will have raise an issue and automatically it will follow that channel it reaches somebody who can solve the problem. Like currently I am the in charge and the patients feel she/he is not satisfied with the services she/he got, some walk away quarreling some come asks for the office of the in charge, administration, director so when they go there they take their problems automatically that person who did that will be called and as usual if you neglect something or show negligence in the line of duty that one you just need to do away with that person because when we are dealing with lives here. So the issue of negligence we don’t condone. You neglect our patient you suffer the consequences, if you feel you are sick or tired you call for help.**

**Moderator:** From the health system level you talked about some patients that you see are running away from the public facilities so what do you think would be a possible solution for that?

**Respondent: The possible solution is because the issue is queuing and you see queuing in public facility most people go there because of cash, some don’t have that money you see public facilities are subsidized services I mean so most of them are affordable so majority go there for services and automatically you see if majority got there it means you need a number of people or services providers also to attend for these number so if the service providers are understaffed there the queue will**

***Second recording***

**Respondent: The patients so that que which makes them ran away is bridged at least they can get there, get services and go to their jobs so that they don’t wait for long and again the supply with drugs I think we need to make sure that there is drug in their facility so that our patients wherever they go like especially a NHIF patients are really crying. Some people are really crying, some people are regretting and wishing to exit from NHIF and join any other insurance because the poor sometimes pay bills on medication including panadol. You are told to go buy, so which stock do they have. So supply of medication they should make sure that there is drug for patients who go in.**

**Moderator:** Ok and we have almost done, so how has the current situation affected the provision of care for hypertensive patient in your community?

**Respondent: The COVID currently has made people to shy away a way from the hospital, that’s the fact. People think that even if you are suffering from any other condition, not even having any sign of COVID that this people they will rather go to a chemist, explain to anybody who is there. Am calling anybody because most of the chemist sometime, you will find the person who sits there is not a doctor, so they go and explain what is happening with them. They take their medication and walk home until it becomes more severe that’s when they approach to the hospital. So COVID has made people shy away too much to the hospital. People don’t want to go to hospital because they think if there go, the hospital they will be tested for COVID -19 and are afraid to be taken to isolation, quarantine so people they mostly shy away and sorry if you are hypertensive and remember these people aren’t going to the hospital, they are sick there is no job, they go back home, the pressure keeps on going high so the situation is getting worse unit it becomes severe then they now approach with somebody, now accompanied, so the patient who is supposed to come early, diagnosed early and helped earlier is waiting until is severe accompanied by somebody maybe this person can’t walk, they are really** **sick. So, COVID has really done a lot** **to make our patients suffer.**

**Moderator:** Has it affected the current hours of operation from your facility?

**Respondent: Yes, to some extent, like when we had the curfew starting at 7 it affected a lot because, you see that they had to leave earlier because they said that there those essential services but police don’t want to hear about essential services, by the time you have produced your identity, you have already been harassed, you find that, people are trying to leave from the work as early as 3pm depending from where you work and where you live that is depending with these bans so nowadays after curfew was extended to 9pm, I think we have ample time to work and do other things, take reports and also patients have time to come to hospital early when curfew was starting at 7pm, there were times when patients could come and tell you that they have been beaten by the police on their way so people are getting afraid, most of them stay at home because they are afraid, how would they walk, they will meet with police and so on. So it resulted to many things and it has affected working hours a lot.**

**Moderator:** What of your availability of antihypertensive medication for your client?

**Respondent: I think I said that when we are starting, I said that availability if medication we have what we can use to sustain our patients and is severe that we can’t with our facility we need to refer but we have our medication that we used to get unless until we had the program of maybe having hypertensive patient like clinics day for the hypertensive patient from there then you see we will able to get like all drugs for hypertension. But coming still for the drugs and management that are currently doing I think we are good.**

**Moderator:** What of the outreaches in your community?

**Respondent: Of late like, I don’t want to lie. Since I came we haven’t done any outreach for the hypertensive but we had outreach for general where we could take BP, we test for sugar, and we have done one since**

**Moderator**: Since the COVID situation has started you have done?

**Respondent: Since the COVID situation that was your question, we have not had any outreach**.

**Moderator:** Is there anything else that has been affected that you have not talked about apart from whatever we have discussed.

**Respondent**: **Anything that has been affected?**

**Moderator:** Yeah, with the COVID situation we have not covered**.**

**Respondent: With the COVID that we have talked, life has been difficult, I don’t see anything that we have not talked of.**

**Moderator:** So we are onto the last question, is there anything else you’d want to talk about in regards to hypertension that you have not mentioned?

**Respondent: Anything that I would want to talk regarding hypertension, I think hypertensive is a serious condition and it makes one to lose his or her life very** **easily if not controlled so I just think that something needs to be done like government wise and private hospital people need to have an outreach for our patients like in the community we are we need to ensure that we align the information that is supposed to be aligned to the people, people are ignorant about their condition, like people are not aware, they lack information to know when they should go to the hospital, should they go to the hospital when they are only sick or should they go to the hospital for checkup and at what intervals, so people need to be educated to get information but of which we lack a lot of facility, finance to boost that, so it is all about the information that people need to have if people get the information. I think everything will just be ok. Hypertensive being controlled well continue with their live. Some will be cured because some sick patients will be healthy hypertension and they say “I stopped medication, am ok, I’ve been doing this, followed advices and they get cured so it is all about the information that that we need aligned on the patients and the community where we are studying so that they get to be informed.**

**Moderators:** So thank you very much for your time, and am thinking the information you have given will be able to reach people who should hear it and it will help us as community and country in general. Have a good day.

**Respondent: Yes too thank you.**

**…END…**
