# Supplementary material for: A socio-ecological framework examination of drivers of blood pressure control among patients with comorbidities and on treatment in two Nairobi slums; a qualitative study
Source: PLOS Glob Public Health. 2023 Mar 10;3(3):e0001625. doi: 10.1371/journal.pgph.0001625 (PMC10021823; doi:10.1371/journal.pgph.0001625)
Supplement: S3 File — (ZIP) [file pgph.0001625.s003.zip › Policy makers/MKD_KII_PDM_200702_0216.docx]

Moderator: {Name}

Respondent: Senior Medical Officer

Code: MKD-KII-PDM-200702_0216

**Moderator:** You confirm that I have read and you have understood the information sheet fore above study. You have had the opportunity to consider the information, ask questions and have had this answered satisfactorily

**Respondent: confirmed. I need an email of what you have just read, you just read to me**

**Moderator:** Mmmmhh

**Respondent: I need to look at it and digest it properly**

**Moderator:** Yeah. Fine

**Respondent: Ok**

**Moderator:** You understand that your participation is voluntary and that you are free to withdraw at any time without giving any reasons without any of your legal rights being affected

**Respondent: Yes**

**Moderator:** And you understand that your data collected during this study may be looked at by individuals where it is relevant in you taking part in the study and you have given permission to these individuals to access your data

**Respondent: Yes**

**Moderator:** You confirm consenting to being audio recorded and also consent to the use on anonymized verbatim quotations

**Respondent: Yes**

**Moderator:** You are happy for your data to be used in any future research?

**Respondent: Yes**

**Moderator:** And you agree to take part in the above study finally

**Respondent: Yes**

**Moderator:** Ok. So on to the questions. We have noticed that we have several challenges in uptake of hypertension which includes physical, structural, policy and financial challenges. So I’ll be seeking your views on uncontrolled hypertension particularly among those who are on treatment.so in your view, what are the challenges in the access and uptake of hypertension care in the community you serve?

**Respondent: Aaahh, currently I will say the first challenge especially in this season of COVID19 is transport for the patients to access health care**

**Moderator:** Mmmhhhh

**Respondent: People use different means like matatus and so on**

**Moderator:** Mmmmhh

**Respondent: So currently now because of the physical distancing and the number of people a matatu can take**

**Moderator:** Mmmmhhh

**Respondent: The fares have gone up**

**Moderator:** Ok

**Respondent: An especially like in Viwandani an informal settlement**

**Moderator:** Mmmhhh

**Respondent: People will tell you that I didn’t have fare to come to the hospital**

**Moderator:** Mmmmhhh

**Respondent: that’s when they have missed their appointments or they had to go to work. You know there is no work**

**Moderator:** Ok

**Respondent: So someone says that they would rather, I actually even had a case**

**Moderator:** Mmmmhh

**Respondent: They would rather go hustle to get something for the family to eat instead of coming for the appointment**

**Moderator:** Ok

**Respondent: So that has also been a challenge**

**Moderator:** Mmmhhh

**Respondent:** And then another thing with hypertension it also goes with diet

**Moderator: Yeah**

**Respondent:** So the client will tell you that I am willing to take my medication but I don’t have food

**Moderator:** Yeah

**Respondent: Like the proper meal that am supposed to take**

**Moderator:** Yeah

**Respondent: So those are some of the challenges**

**Moderator:** Ok. What of the physical number of public facilities in the area

**Respondent: Public I Viwandani we just have one**

**Moderator:** Mmmmhh

**Respondent: The population is quite big. Actually that’s why it has been a very big challenge coz Viwandani is a very big physical area**

**Moderator:** Mmmmhh

**Respondent: And of course the population is also quite high compared to the other wards in Makadara**

**Moderator:** Mmmmhhh

**Respondent: But we also find that we only have one public facility, a health center in Viwandani**

**Moderator:** Ok

**Respondent: Yeah**

**Moderator:** And what of the policies. Are there any clear guidelines for hypertension care in the facilities in your community?

**Respondent: The guidelines are there**

**Moderator:** Mmmmhh

**Respondent: Like when do we diagnose hypertension, when do we start medication and how do we scale up or scale down**

**Moderator:** Mmmhhhh

**Respondent: So the guidelines especially the clinical ones are in place**

**Moderator:** Ok

**Respondent: Yeah**

**Moderator:** So any financial challenges in allocation for hypertension care?

**Respondent:** From my health facility point of view

**Moderator:** Yeah

**Respondent: We can’t have a specific allocation for example NCDs that is non-communicable diseases of which hypertension falls under**

**Moderator:** Ok

**Respondent: I think we all, how do I put it**

**Moderator:** Mmmmhh

**Respondent: All health care combined**

**Moderator:** Yeah

**Respondent: Again you see we are battling a lot of communicable diseases**

**Moderator:** Mmmmhhh

**Respondent: So the non communicable ones are eeehh. We don’t give them the emphasis that they deserve because we are overwhelmed already**

**Moderator:** Ok

**Respondent: Our emphasis is more on communicable ones like you have seen of late, the non-communicable diseases like the hypertension and diabetes are going up**

**Moderator:** Ok

**Respondent: Yeah**

**Moderator:** Are there any challenges in terms of expertise in health care provider and equipment’s for blood pressure monitoring?

**Respondent: At the facility level I would say no**

**Moderator:** Mmmmhhh

**Respondent: The clinicians are there but the challenge we have is, how do I put it**

**Moderator:** Mmmmhh

**Respondent: I think it would be good to have just for example a hypertension clinic or something like that but now the challenges are the numbers**

**Moderator:** Ok

**Respondent: We have a shortage of staff that makes it difficult to allocate that this clinician will specifically sit at the hypertension or diabetes clinic**

**Moderator:** Mmmhhh

**Respondent: So it’s just an outpatient so they just come there. It doesn’t matter whether you have hypertension or malaria or you are coming with. I don’t know if you are getting what am saying**

**Moderator:** Mmmmhhh

**Respondent: We don’t have a specific day for a specific clinic being seen by a specific clinician**

**Moderator:** Ok

**Respondent: Yeah,** **now those are the challenges that we have**

**Moderator:** And any challenges relate to the facility working hours?

**Respondent: Facility working hours, initially yes we had a challenge because of the curfew hours of 7pm**

**Moderator**: Yes

**Respondent: So we were forced to open by 8 and by 3 we close so that our health workers are able to get home on time**

**Moderator:** Yeah

**Respondent: And even the patients are able to get home in time**

**Moderator:** Ok

**Respondent: But now since the review to nine o’clock we are seeing even the numbers increasing**

**Moderator:** Yeah

**Respondent: Since we have gone back to our normal 8-5 hours**

**Moderator:** Mmmmhhh

**Respondent: The only challenge we have especially with the Health Center is that we don’t operate during the weekends and we t operate at night**

**Moderator:** Mmmmhhh

**Respondent: So it’s just from 8-5**

**Moderator:** Ok

**Respondent: So incase a patient gets hypertensive emergency at night or need medication or a checkup over the weekend it’s not possible**

**Moderator:** Ok. Any challenges related to medication stock out in the facility?

**Respondent: Yes there are there, sometimes we get stocked out**

**Moderator:** Ok. So how about

**Respondent: And then the other challenge in medication you find that there are these, I don’t know how to call them**

**Moderator:** Mmmmhhh

**Respondent: The combinations**

**Moderator:** Ok

**Respondent: There are some medications that you feel s clinicians that they are better but now what we have in stock is not that**

**Moderator:** Yeah

**Respondent: So it’s either patients go out of their way to buy yet most of them can’t afford, so they just take what we have**

**Moderator:** Ok. you had talked about you’ve had staffing challenge and I think how about the capacity work load to the employees that you have at the facility providing this care

Respondent: The capacity of course they are being over stretched

**Moderator:** Mmmmhh

**Respondent: Because of the numbers**

**Moderator:** Ok

**Respondent: Because if you are few and you are supposed to see 50or 100 patients in a day**

**Moderator:** Mmmmhh

**Respondent: Those are quite many**

**Moderator:** Mmmmhhh

**Respondent: if we had two or three clinicians distributing those numbers then I would be better in terms of are we giving quality care**

**Moderator:** Yeah

**Respondent: Especially a facility like {Name of the facility}which is the only health center in Viwandani**

**Moderator:** Mmmmhhh

**Respondent: The numbers have increased in terms of what they are used to handling**

**Moderator:** So what is your opinion can be done to these facilities to alleviate the access and uptake. We are going to talk about these in different perspectives and we are going to start from the patient’s perspective

**Respondent: Mmmhhh**

**Moderator:** What are the challenges you think will be alleviated in the access and uptake for uncontrolled hypertension care?

**Respondent: I think first of all we need a lot of health education to the patients because you see hypertension is not like malaria where you will have a headache or pain**

**Moderator:** Mmmmhhh

**Respondent: Hypertension come in more less very… it’s asymptomatic and by the time am having maybe ahead ache that means that my blood pressure is really high**

**Moderator:** Mmmmhhh

**Respondent: Because of that people wait until they feel pain for them to go to the hospital**

**Moderator:** Yeah

**Respondent: and even when they are on medication, because the patient is not in pain**

**Moderator:** Mmmmhh

**Respondent: They may not adhere and when they find that their blood pressure is maybe the reason as to why they came to the hospital and then you find out that the hypertensive was the leading**

**Moderator:** Mmmmhhh

**Respondent: Of course once you control the blood pressure the headache will go away**

**Moderator:** Ok

**Respondent: So the challenge on the patients side is that when the head aches has gone then they feel that they don’t need to continue with the medication**

**Moderator:** Mmmmhhh. Ok

**Respondent: So we would really need to talk to these patients educating them especially on the complication coz if the blood pressure is uncontrolled then you are putting your organs at risk because of that high blood pressure**

**Moderator:** Mmmmhh

**Respondent: Then you end up with kidney failure, heart failure or those other complications that come with hypertension**

**Moderator:** Ok

**Respondent: Yeah**

**Moderator:** At the community of family level perspective, what do you think we could do to alleviate the access and uptake challenges of uncontrolled hypertension?

**Respondent: I think involving the family members because if for example a spouse is aware that you are hypertensive and this are the complications**

**Moderator:** Mmmmhhh

**Respondent: And you are supposed to be on medication, the will always check or you become accountable to them**

**Moderator:** Yeah

**Respondent: I think that’s something that can be done to improve so that the up taking is better and also not just to… Like for example when I give a patient an appointment to come for clinic next week, I don’t know I should contact the next of kin I don’t know just get someone to walk with**

**Moderator:** Mmmmhhh

**Respondent: So that if I can’t see the patient I can contact their wife, son or daughter just to remind them of their appointment**

**Moderator:** Ok

**Respondent: Yeah**

**Moderator:** And from the providers perspective, what do you think we could do

**Respondent: I think the follow up is important just to keep you know like remind them of their appointments especially for blood pressure if they can take their blood pressure machines at home**

**Moderator:** Mmmmhh

**Respondent:** Nowadays we have the portable ones that you can easily have in your house

**Moderator:** Mmmhhh

**Respondent: You can have like come up with ways in which a patient can get back to you or maybe give them a log where they can log in their blood pressure every day and if they can’t afford the machine**

**Moderator:** Mmmhhh

**Respondent: We have even labs and these small small chemists in the estate where you can just go and pay 20 bob and your blood pressure is checked**

**Moderator:** Mmmmhhh

**Respondent: Yeah, just having a log on your patients to check on them regularly**

**Moderator:** What of from the health systems level perspective. What do you think we could do?

**Respondent: Health system perspective I think the biggest challenge is staffing, equipping the facility with equipment that are required, medication, having a variety where clinicians can … (not clear) from**

**Moderator:** Mmmmhh

**Respondent: The other thing is as much as we are putting efforts on communicable diseases**

**Moderator:** Mmmmhh

**Respondent: just make the same noise concerning the non-communicable ones**

**Moderator:** Ok.

**Respondent: Yeah**

**Moderator:** What of from the policy level perspective

**Respondent: policy I think I’ll say the same thing like the health system level. They should come up with policies that don’t just focus on communicable diseases but also non communicable ones**

**Moderator:** Mmmmhh

**Respondent: And then also provide data like a data base. I think also our data base is not very good.**

**Moderator:** Mmmmhh

**Respondent: It’s very easy for me to say that there is this number of people dying from malaria like every year but it’s not easy for me to get the number of people dying from hypertension for example like every year**

**Moderator:** Ok. Everyone is talking about COVID19, you are also talking about COVID, the nation is talking about COVID and you have mentioned that it’s affecting transport for the people who are coming for treatment in the facility

**Respondent: And even the numbers co we are discouraging crowding**

**Moderator:** Yeah

**Respondent: Initially when the pandemic started, we were discouraging people from coming to the health center and we encouraged our clinicians to give long term return dates so that patients who were on follow up weekly were given medications that would last them for like a month so that they don’t need to come like every other week unless they have a complication**

**Moderator:** Mmmmhh

**Respondent: Or something new comes up**

**Moderator:** Ok

**Respondent: That’s how COVID has affected us but now we are slowly coming back to normal. The clinics are now running the same way they were running**

**Moderator:** Mmmmhh

**Respondent: We have not got back there but slowly we are getting there**

**Moderator:** Ok On to the final question, is there anything else that you feel like we have not talked about hypertension care and you feeling like we should talk about?

**Respondent: Hypertension care is also an aspect of lifestyle change and not just medication and I feel lie sometime we are not very keen. I don’t know if it is because we are physicians so we just want to prescribe.**

**Moderator:** Mmmmhh

**Respondent: I don’t know if... We are always fast to put patients on medication again you see hypertension is more or less like a life time disease where by when you start medication it has to take you a long time before we actually say now you can stop this**

**Moderator:** Yeah

**Respondent: I think we are not doing well in terms of giving people advice. We are not giving them education on lifestyle change**

**Moderator:** Mmmmhhh

**Respondent: Like actually you can normalize your pressure just by… coz most of them it comes in because of lifestyle**

**Moderator:** Mmmmhh

**Respondent: so if you exercise regularly, your diet contains certain … You know mount of maybe carbohydrates and fats and things like those**

**Moderator:** Mmmmhh

**Respondent: You are able to contain your diet and exercise; your pressure can actually normalize**

**Moderator:** Yeah

**Respondent: And maybe even at some point get off the drugs like completely**

**Moderator:** Ok

**Respondent: I think that is one aspect that we’ve never been very active on which can actually really help. It will help in terms of burdening coz again taking a pill every day like one pill, 2 pills every day becomes a challenge to many people**

**Moderator:** Ok

**Respondent: So if you can give them an option that maybe not immediately but over time you can get off the medication I you follow a certain routine or lifestyle like walk more, sit less, take the stairs instead of using lifts. You know those small small lifestyle changes**

**Moderator:** Yeah. So thank you so much for your time {Name}. We appreciate for your time and hope that the information you have given us will help us make change in policies that we make and help the community at large and am really appreciating your participation in this.

**Respondent: You are welcome. I hope the information I gave was relevant**

**Moderator:** It was

**Respondent: Ok**

**…End…**
