# Supplementary material for: A socio-ecological framework examination of drivers of blood pressure control among patients with comorbidities and on treatment in two Nairobi slums; a qualitative study
Source: PLOS Glob Public Health. 2023 Mar 10;3(3):e0001625. doi: 10.1371/journal.pgph.0001625 (PMC10021823; doi:10.1371/journal.pgph.0001625)
Supplement: S3 File — (ZIP) [file pgph.0001625.s003.zip › Policy makers/NRB_KII_PDM_200629_2302.docx]

**Moderator: {Name}**

**Respondent: Medical Officer**

**Code: NRB-KII-PDM-200629_2302**

**Moderator:** Confirm that I have read an you have understood the information sheet for the above study and you have had the opportunity to consider the information and ask questions that I have answered you to your satisfactory

**Respondent: Ok**

**Moderator:** You understand that your participation is voluntary and you are free to withdraw at any time without giving any reasons, without any of your legal rights being affected

**Respondent: Yes**

**Moderator:** You agree that the data collected during this study may be looked at by individuals where it’s relevant to your taking part in this study and you give permission for these individuals to access your data

**Respondent: Yes, I agree. Of what importance is the study to me?**

**Moderator:** To you as a policy maker you will be able to understand where the challenges are and probably make changes or add to something that you think is not working at the moment

**Respondent: Are you going to share the results of the study there after?**

**Moderator:** Yes

**Respondent: Ok**

**Moderator:** Yeah. You confirm

**Respondent: I don’t want to just give the information; I would expect that once you complete the study**

**Moderator:** Yeah

**Respondent: You also share with us**

**Moderator:** Yeah. We will

**Respondent: Hope your findings work**

**Moderator:** We will do that

**Respondent: Yes**

**Moderator:** You confirm consenting to be audio recorded and you also consent to the use of anonymized verbatim quotations

**Respondent: It’s ok**

**Moderator:** And you are happy for your data to be used in future research

**Respondent: No problem at all**

**Moderator:** And you agree to take part in the study lastly

**Respondent: Yes**

**Moderator:** So am going to read to you a small statement and then now we can start with the questions

**Respondent: Ok**

**Moderator:** There are several challenges in access and uptake of hypertension which include physical, structural, policy and financial challenges

**Respondent: Physical…**

**Moderator:** Physical, structural, policy and financial challenges

**Respondent: Mmmhhh**

**Moderator:** So am seeking your views on uncontrolled particularly to the people who are on treatment. On to the first question

**Respondent: Eeehh**

**Moderator:** In your view, what are the challenges in access and uptake of hypertension care in the community you serve?

**Respondent: the biggest hindrance is actually lack of regular screening and monitoring**

**Moderator:** Yeah

**Respondent: You realize that patients who have been diagnosed don’t do self-monitoring and they are not monitored in a health facility**

**Moderator:** Ok

**Respondent: So that definitely affects the uptake care**

**Moderator:** Ok

**Respondent: The other thing is that more often than not**

**Moderator:** Mmmmhh

**Respondent: There is the prohibitive and I think that will fall under financial. There is the prohibitive cost on medication which is quite challenging in informal settlement commonly because the medication themselves are quite expensive**

**Moderator:** Yeah

**Respondent: So that also hinders the control of hypertension**

**Moderator:** Ok

**Respondent: In terms of physical and structural we also realize that there is no adequate space for physical activities**

**Moderator:** Mmmmhh

**Respondent: An where there’s space there is the issue of security**

**Moderator:** Mmmmhh

**Respondent: Especially in the informal settlements**

**Moderator:** Ok

**Respondent: Coz you expect them maybe to do a morning jog which might not be very feasible due to surety reasons in those areas**

**Moderator:** Mmmmhh

**Respondent: And also the way the settlements are, there are o spaces where people can do physical activities**

**Moderator:** Ok

**Respondent: Maybe that’s all. Policy, one of the other things that affect this**

**Moderator:** Mmmmhh

**Respondent: Our health facilities in the same areas the health providers do not have eehh…they are not equipped very well in terms of skills for them to be able to manage the same conditions**

**Moderator:** Ok

**Respondent: Yes**

**Moderator:** On the physical part, what could you tell me about the number of facilities you manage in your area?

**Respondent: In Korogocho we only have one public health facility that is {Name of the facility)**

**Moderator:** Yeah

**Respondent: And in Viwandani it’s only {Name of the facility} in the vicinity. So it’s just one facility that caters for the whole population**

**Moderator:** Ok. And on the policy level

**Respondent: Mmmmhh**

**Moderator:** Kindly tell me about the guidelines for hypertension care

**Respondent: There are guidelines that are available for hypertension in the facility and we have also conducted some training and dissemination of the same**

**Moderator:** Yeah

**Respondent: So the … (Not clear) is quite… (Not clear) and we even have… (Not clear) and the standard of operation procedures on how to manage the condition**

**Moderator: Mmmmhh**

**Respondent: The only gap would be like I mentioned earlier the training, capacity building on skills of the health care providers**

**Moderator:** Ok. So…

**Respondent: Policies are there and they are being effected**

**Moderator:** Ok. The facilities you have in Korogocho you have said is one and in Viwandani is one. O could you tell me or could you have an idea if any either of the facility health care providers that manage the hypertensive services are trained or not

**Respondent: They are trained**

**Moderator:** Ok. So on the financial bit, could you tell me about the allocation of hypertension care

**Respondent: We don’t differentiate into specifics**

**Moderator:** Yeah

**Respondent: What we have is a comprehensive package for the sector that is the health sector**

**Moderator:** Mmmmhh

**Respondent: Under which it is very difficult to quantify specifically for hypertension**

**Moderator:** Mmmmhh

**Respondent: Because if it is for example file for procurement of commodities, they all go together including the other essential commodities and non-pharmaceuticals**

**Moderator:** Mmmmhh

**Respondent: So it is quite difficult to tease out specifically for hypertension it’s further a comprehensive package**

**Moderator:** Ok. So are there any challenges in time? You have talked about you have challenges in, you are not sure whether the health care providers are not well equipped in the facilities that you have told me. Do you have challenges with them using either equipment on blood pressure monitoring or any other thing like that?

**Respondent: The use of equipment is not a challenge at all**

**Moderator:** Yeah

**Respondent: The challenge comes when now it comes to management**

**Moderator:** Mmmmhh.

**Respondent: Ok. This is a hypertensive patient, what should I do?**

**Moderator:** Yeah

**Respondent: That’s where the challenge is but in terms of usage of equipment, taking of blood pressure, I can’t consider that to be a challenge**

**Moderator:** Ok. What are the, do you have anything to do with staffing challenges in the facilities that you manage?

**Respondent: Yes**

**Moderator:** Yeah

**Respondent: Big big challenge, and that’s why I said it may sound contradictory, am telling you that the people who are there are trained and then am saying there is lapse in the skills**

**Moderator:** Yes

**Respondent: This is because the few who are trained**

**Moderator: Yeah**

**Respondent: Out of the numbers that we have, a few of them are trained, so when this ones are absent for one reason or another,**

**Moderator: Mmmmhh**

**Respondent: The ones that are left are not able of course to manage**

**Moderator: Ok**

**Respondent: And honesty it’s a big challenge especially when it comes to HR**

**Moderator:** Mmmmhh

**Respondent: We are really struggling especially now during this COVID time coz if we are to withdraw some of our staffs to manage he quarantine centers and the isolation centers**

**Moderator:** Ok. So any challenges related to facilities working hours?

**Respondent: Definitely yes coz mostly those facilities don’t run over the weekend**

**Moderator:** Yeah

**Respondent: They run up to 5 in the evening so those who want to access services past those hours especially those who are coming from work places cannot be able to access the service. Those who only available over the weekend are not able to access the service**

**Moderator:** Yeah

**Respondent: Yes**

**Moderator:** Challenges related to medicine such as stock outs, do you have any challenges with that?

**Respondent: Sorry, in terms of?**

**Moderator:** Challenges related to medication such as stock out

**Respondent: Yes, definitely we usually experience challenges such as stock outs**

**Moderator:** Mmmmhh

**Respondent: There is that erratic supply of commodities but its beyond our control because that’s a procurement issue. We are able to quantify the needs; we are able to order on time byt we are not able to receive supplies on time**

**Moderator:** Mmmmhh

**Respondent: So it definitely affects**

**Moderator:** So now for example the last batch that you ordered, when was that?

**Respondent: Am not the one who does the ordering, it’s not within my scope**

**Moderator:** Ok

**Respondent: So I may not be able to specifically tell you when it was ordered or when it was delivered or what is the status**

**Moderator:** Ok

**Respondent: That is at a lower level actually at the sub county level**

**Moderator:** Ok. So what about the work capacity load on the employees providing the care? What do you think about that?

**Respondent: It is overwhelming**

**Moderator:** Mmmmhh.Ok. What in your opinion can be done to alleviate the access and uptake challenges for hypertension care? We are going to talk about specific levels and each level has its own category. So to the first one is individual or patient level perspective. What do you think can alleviate the access and uptake challenges for uncontrolled hypertension care?

**Respondent: Most important for me is education. If we have education for the clients to understand what they are going through and how it can be managed**

**Moderator: Yeah**

**Respondent: We need to empower them with information, what s hypertension, what cause it**

**Moderator: Mmmmhh**

**Respondent: You know if we demystify the disease at their level, teach them how to control so that they take care of their generations**

**Moderator: Ok**

**Respondent: I think because we realize that … (Not clear) so we need to empower the clients**

**Moderator:** Ok. From the community and family level perspective, what do you think would alleviate the access and uptake challenges?

**Respondent: Again ill priorities health education and then of course screening especially if there is a history of hypertension**

**Moderator:** Yeah

**Respondent: At the family level, screening is important**

**Moderator:** Yeah

**Respondent: At the community level we also need to do screenings because we realized that in most cases the disease is not symptomatic**

**Moderator:** Yes

**Respondent: So there could be many people outside here who don’t know their status**

**Moderator:** Mmmmhh

**Respondent: Their pressure, so we need to scale up screening at the community level**

**Moderator:** From the provider’s perspective, what do you think would alleviate that?

**Respondent: at the providers we need to build their capacity to manage he condition**

**Moderator:** Mmmmhh

**Respondent: And equip them with necessary equipment and commodities**

**Moderator: Mmmmhh**

**Respondent: And maybe enable them to reach the community and not to expect the community to come to the health providers. It would be better if the heal provider went to the community**

**Moderator:** Ok

**Respondent: Yes**

**Moderator:** And from the health systems perspective, what do you think would be…

**Respondent: From the?**

**Moderator:** Health system level perspective

**Respondent: Health system?**

**Moderator:** Yeah

**Respondent: I think one is that we need to strengthen the linkage between the community and the facility in terms of care, treatment**

**Moderator:** Yeah

**Respondent: that is from the community to the facility and back to the community**

**Moderator:** Yeah

**Respondent: We need to again build the capacity of the health care providers for them to be able to address those issues**

**Moderator:** Mmmmhh

**Respondent: And equip our facilities to have the necessary equipment and commodities to manage hypertension**

**Moderator:** Ok

**Respondent: And also very important, data management**

**Moderator:** Data management

**Respondent: Yes, that’s very very crucial**

**Moderator:** Mmmmhh

**Respondent: Because you realize that we may be doing a lot but it’s not accounted for in terms of data collection**

**Moderator:** Mmmmhh

**Respondent: So we need also to strengthen up that component**

**Moderator:** Mmmmhh

**Respondent: Yes**

**Moderator:** And then now from your office now or from the policy level perspective, what do you think will alleviate the access and uptake of the challenges that we are seeing

**Respondent: Now, I was saying. From the policy level we need mechanisms to monitor**

**Moderator:** Yeah

**Respondent: Monitoring and evaluating what is happening beyond the policy level.**

**Moderator:** Yeah

**Respondent: You know we need to do DQAs, audit data, support the sub county and the facilities, provide them with reporting tools,**

**Moderator:** Mmmmhh

**Respondent: That is the HMIS tools for reporting**

**Moderator:** Ok

**Respondent: Yes**

**Moderator: That’s fine. So we are almost done, remaining only two questions**

**Respondent: Yes**

**Moderator:** So how, everyone is talking about the COVID situation and how it has affected many lives from the world and narrow down to our community. So how has the COVID situation affected the provision of hypertensive services in the community?

**Respondent: COVID has greatly affected the especially considering the very vulnerable population is actually those living with comorbidities such as hypertension**

Moderator: Yes

**Respondent: and where else we are still with the LCD clinics as we call them, we are told to discourage hospital visits for those who have hypertension**

Moderator: Ok

**Respondent: What we have adopted is differentiated care model where we are working on reaching them at their house hold level instead of them coming to the facility**

Moderator: Ok

**Respondent: That now addresses two issues, number 1 is the reduced movement and adhering to what the ministry and WHO guides especially for those who have comorbidities**

Moderator: Ok

**Respondent: It’s also cautioning them in terms of costs of accessing the facility and we are also trying to see that not unless they have complications**

Moderator: Yeah

**Respondent: We have given them information on what to watch out for**

**Moderator: Mmmmhh**

**Respondent: That they can get their blood pressure monitored at the house hold level without them necessarily having to come to the facility. So it has actually been greatly … (Not clear) COVID**

Moderator: Ok

**Respondent: Of course there are those we no longer know where they went because they are no longer coming to the facility**

Moderator: Yes

**Respondent: We can neither call them. Actually our numbers have reduced**

Moderator: Yeah

**Respondent: When you compare like this time last year, the numbers being reported have really gone down**

Moderator: Ok

**Respondent: Yes**

**Moderator: is there anything else you would like us to talk abou hypertension care which we have not discussed?**

**Respondent: Yes, two things**

Moderator: Yeah

**Respondent: number 1 diet**

Moderator: Diet

**Respondent: Because it is involved a lot when it comes to hypertension and its one of the biggest modifiable risk factors**

Moderator: Yeah

**Respondent: Number 2, there is the lifestyle**

Moderator: Yeah

**Respondent: Talk about smoking, alcohol intake, lack of physical activities**

Moderator: Mmmmhh

**Respondent: All those are contributing factors when it comes to hypertension**

**Moderator:** Yeah

**Respondent: And I thing finally also other important thing is there is correlation between hypertension and many other conditions**

**Moderator:** Yeah

**Respondent: For example somebody who is diabetic is more prune to developing hypertension if the sugars are not well controlled**

**Moderator:** Mmmmhh

**Respondent: And of course other conditions like HIV. In one way or another contribute to…**

**Moderator:** Ok. I guess now we are done with the interview. Thank you very much for your time and I hope the information that you gave me will be able to get back to you and whatever gaps that will be found will be to be monitored and the information to be of help to both us and the community

**Respondent: Yes**

**Moderator:** Thank you so much for your time {Name}

**Respondent: You are most welcome**

…END…
