# Supplementary material for: A socio-ecological framework examination of drivers of blood pressure control among patients with comorbidities and on treatment in two Nairobi slums; a qualitative study
Source: PLOS Glob Public Health. 2023 Mar 10;3(3):e0001625. doi: 10.1371/journal.pgph.0001625 (PMC10021823; doi:10.1371/journal.pgph.0001625)
Supplement: S3 File — (ZIP) [file pgph.0001625.s003.zip › Policy makers/NRB_KII_PDM_200702_0150 docx.docx]

**Moderator**: **{Name}**

**Code**: **NRB**-**KII**-**PDM**-**200702**-**0150**

**Moderator:** So you confirm that I have read and you have understood the information that I have read to you and you had the opportunity to consider the information and ask questions

**Respondent:** Yes and I consent to participation or my participation in this study

**Moderator:** You understand that your participation is also voluntary and you are free to withdraw at any time without any of your legal rights being affected

**Respondent:** Very well

**Moderator:** You understand that the data being collected during this study may be looked to these individuals where it is relevant to your taking part in this study, you give permission for these individuals to have access to your data.

**Respondent:** Yes

**Moderator:** You confirm consenting to being audio recorded and you also consent to the use of anonymized verbatim quotations

**Respondent:** Yes

**Moderator:** And you are happy that your data may be used in future research

**Respondent:** Yes

**Moderator:** Now finally you have said you agree to take part in this study. Thank you very much for that now I am going to read for you a small sentence and I will introduce the questions themselves, so we have noticed that there are several challenges in access and uptake of hypertension which include; physical, structural, policy and financial challenges. So I will be seeking your views on uncontrolled hypertension particularly among those who are on treatment.

So on to the first question, in your view, what are the challenges in the access and uptake of hypertension care in the community you serve?

**Respondent:** Very good have told you that I serve at the national level and more often the note at the national level we have many concern with the policy development, then we have the responsibility of capacity building, the health worker and in this respecter we refer to hypertension which is one of cardiovascular condition. The national level we also have the referral facilities but I am sure of main concern here is generally at large, what are the challenges which we are facing in terms of making sure we have got good control of hypertensive clients or patients in this country. For one I would want to refer to the state wise survey which as ministry with partners we conducted in 2015, we shall maybe serve as our baseline whereby we realized from the survey that almost one in every four Kenyans adults has hypertension. But of those who have hypertension very few were under treatment, management which is about 8% and those were in good were 3% ,but that is long time since then and of course we realized that quite huge number of Kenyans more than 50% have never had their blood pressure tested, so that’s one of issue that tells us that looking at the number which has never screened for blood pressure tells you the level of awareness there is low, is quite needy and therefore we need to scale up our activities on advocacy and awareness creation largely because we want to believe that very few people have gone for screening because more often than note hypertension is at time present with no signs and symptoms and basically as you know hypertension is that resistant which the heart pisses pumps blood across the body and quite a time resistant as symptoms the clients or patients may have no symptoms still we get to that extreme.

Having said that, then what are the challenges we are facing in terms of having patients have good control of hypertension. Number one I have talked of low awareness within the community, number two most of the time this condition presents without signs and symptoms, number three the capacity of health workers or health care providers to manage hypertension is a bit wanting. This is following some work with AMPATH at Gusii and Transzoia quite a number of health workers didn’t have adequate knowledge to manage hypertension if I can go by my study. So that’s the third matter. Then the other issue which I want to say is equipment and essentials medicines thank to our president now he has one of his priorities he wants to address before his time to retire comes in 2022.The big four one of them is universal health coverage, it is true that UHC the ministry has been able to avail quite substantial with number of equipments particularly BP machines with the low facilities starting from level two that is dispensary upwards. Then again we have developed a list as ministry of essential medicines more so for these starting on hypertension and they are now available at KEMSA and facilities and counties are easily able to get these supplies. This hasn’t been the case before, ok?

**Moderator:** Yes. The fourth issue was basically the availability of equipments and essential medicines so but for now the issue of the basic equipment and medicine may not be, we can’t consider that a very major factor because it has been addressed by UHC to larger extent although again now it may have been addressed to that extent by UHC but again despite that you find that some of our client still go to private facilities and expenditure out of pocket is fairly high and that need the issue of affordability. But again when we did a study and looked at although the results were not good, we don’t want to go to much to the issues of availability and cost mark us for hypertensive drugs because of having them easily available from our supply chain and I think issue has improved unlike before when now drugs are easily available at a fair cost but remember not all clients are able to cater, afford that, again most of the clients may lack necessary knowledge of where to acquire medication and affordable management of hypertension.

So are there any challenges that are related to physical the number of facilities in the area?

**Respondent:** Good, generally let me say that as far as availability of physical facilities where these services are available it may not wouldn’t say that a major challenge in terms of the management or control of hypertension, because you find in our country through ministry of health we have embraced primary health care and through this we are able to offer services even at the community level where we have community health volunteers and this is one of issue which have been embraced by universal health coverage and taking account health services are devolved and counties are really supporting this initiative of CHV and one of the basic equipment of counties and health sectors says equipping this health care provider with equipping is basic equipment like BP machine and not withstanding now unlike before when you were to go to a facility and meet a facility without a BP machine. I think today this is not a normal practice but sometime back from few years ago that was an issue and they really contributed to poor management of hypertension because maybe a client would go to a facility and find there is no BP machine but I don’t think that’s the case now likewise even the equipment the BP machine are ready available even in chemist and most of hypertension patients have personal BP machine ,but that notwithstanding the patient may know the numbers but the issue is whoever managing the patient does that person have adequate capacity? I think that’s where we could be now talking of now challenges ,serious challenges starting from but all is not lost because having done our and having realized we have challenges with control of hypertension and the ministry will cause have to put some measures into place so that mitigating factors and one of them was to make sure that we embrace primary health care ,offer that services at lowest level available equipment am sure with the devolutional health the issue of infrastructure that dispensary and all that isn’t a major issue but we had a challenge as I have told you with health care provider capacity then initially we had issues with what availability of essential medicine but I don’t think that is much of an issue and again but we still have challenges with advocacy and awareness creation. This is one condition which is easily manageable if we are able to pass information to the population at the right time then the population gets aware of the risks factor especially the multipliable risk factors like health diet and when we talk of health diet we need to tell them what is health diet because if we talk to a common Kenyan maybe health diet you might think that is meat, kachumbari.And also we need to address the issue, harmful issue of tobacco, physical inactivity. Those are identifiable risk factors, I think the population may not have adequate knowledge about them which might help us from the word go leave alone having good management of hypertension but avoiding the condition from developing even after the condition developed we need to continue taking care of these modifiable risk factors including other things like lipidemia the level of hand lipid in our blood and all that, great.

**Moderator:** And from where you sit at the moment as a policy maker and decision maker do you think there are challenges in making policy and clear guidelines for hypertension care

**Respondent:** Good, we do that by highlighting on policy issues as ministry we have what we call Kenya health policy framework 2014-2030 and this document clearly the directions which we have to follow as a country in terms of policy we talk of provision of accessible health care and making sure there is equity in provision of these services. First of all we would want to strengthen health leadership, what do I mean by health leadership? You see like now the health is devolved function in order for those conditions like hypertension to get adequate attention we have to bid the capacity the leadership of county starting from the Governor, county assembly .These are the people who allocate funds. We don’t give them the correct information about the importance of these things, you find that they may take money in putting up a dispensary rather than training a health worker on how to manage because they are the owner of the money so we are very strong on that capacity of health leadership from national to the county. Similarly health financing is another issue, we have to make sure health budget is allocated adequate finances in order for us to have sound health systems ranging from health care providers the infrastructure and availability of essential medicines ,that would go very well towards improving the control of making sure that we have a good control of hypertension. Apart from that we need to build a very strong health information systems whereby we talk of now having into right from the ministry level of service provision to the highest level and as a ministry we need to know how many people are suffering from hypertension, how many are able to get the medication they need, how many are under control so health is very keen and I think I have talked about other issues about health worker where we need to have to build their capacity. Then from there I think at policy level we talk of policy objectives and I don’t want to go down to these because we have around six policy objectives and out of those six policies objectives about five of them directly or indirectly addresses that communicable disease and one of the commonest communicable condition is hypertension and therefore that shows the priority we are according hypertension matches the prevalence although the financing maybe an issue then when it comes to issues of guidelines we develop employment for insomnia managing hypertension but cardiovascular diseases taking to account the most common cardiovascular condition is hypertension and therefore the guideline for the management of hypertension are very well or elaborately addressed in our guideline for management of cardiovascular diseases right from the community level to the highest level. We have also highlighted on the referral system both way bottom up and up bottom so that it is not an issue but this is the document we developed in 2018, that tells you what was happening there before may not have been very well coordinated and any health care provider may have been managing hypertension in the way one believe it is best but now we have some guidelines and where there you are getting your management from Aga khan or from that small facility let me say the care is the same. We are also trying as much as possible although we may not have the same equipment we are trying to bring issues of equity on board whereby we have basic equipment in all the facility. I have given you even more 15 minutes now, we have very pertinent one I think have talked

**Moderator:** So you have talked about across the board, do you think you have any challenges with staffing issues with ones who take care of hypertension patients?

**Respondent:** Good apart from the knowledge and skills which we are trying to improve and that one may be because of function of the ministry of health at national level capacity and that one I would say we are and again when it comes to service delivery in terms of numbers of health care providers that becomes a challenge . Definitely we can’t talk of adequate health care providers in generally and particularly we were to talk of hypertension you know how that becomes a specialized area, so issues of staffing yes we have some issues

**Moderator:** So we can combine that on how about capacity of building or work load providing the care?

**Respondent:** When I talk of now the staffing and work load you find the workload is so high because the staffing level hasn’t been achieved so we need to employ more health care providers so that we can bring the workload down, upon employment we need them to capacity build more often than not where it comes to hypertension of that field so that they can give that very essential care required for control of hypertension and not only for control but also for appropriate referral to know when to refer either up or downwards because we may have many patients on hypertension under good control maybe at a district level or county level increasing the workload and therefore that may end up resulting to some may be not achieving desire level management. So if we have patients who are well controlled you can downward them to the lower level and remain with those you need there, they are needy that is where now we are talking of referral system up and down and increase the number of staff very critical

**Moderator:** And you have talked earlier you had challenges of essentials medicines but at the moment you don’t, the medicine have been supplied

**Respondent:** Let me put the correct situations, with UHC it has improved the level of availability of essential medicines but of course not a 100% there is still some challenges I am sure you may not be able to provide to meet the need of Kenyans as per desire up to a certain percentage but it has really some improvement from, you get it?

**Moderator:**  in your opinion what can be done in these communities to alleviate the access and uptake challenges for uncontrolled hypertension care, on individuals you are going to look on different perspectives. We are going to start with patient perspectives ,we had talked about if patient are being taught of what hypertension is and them being able be aware of signs and symptoms because you had said earlier that sometime hypertension don’t have signs and symptoms

And you had also talked about they should be taught of harmful use of alcohol use, tobacco use and they should be taught on physical activities, so anything else you think you want to add to that?

**Respondent:** Thank you on trying to guide me on the issue of access but I want to believe generally I have addressed the issue of access before, I have said this is a condition more often than not it is un simplematic so don’t expect a normal person to go to hospital to be checked on what maybe the person believes it doesn’t exist so one thing as I had said earlier we need to increase level of awareness among our population that hypertension is normal referred to as silent killer. More often it that present without signs and symptoms therefore even if you feel you are ok please make sure you go for periodic checkup. And this is what we are advocating for even at the country level and when a client comes for periodic checkup this client should not be asked to pay for that and if one has to pay it has to be something minimal, so awareness then periodic check – up and also what we call mass screening. We should have more also at county level the culture of testing people up whenever the Is a need arise like when the governor has called people for meeting to improve access let the nations that religion come with a BP machine they sit at the gate so that as people come for baraza they get tested and you will find wananchi will always offer themselves for that and that is why I even talked of leadership. You see some as something distracting their peace not forgetting whoever is being screened is their people ,so in terms of patients access the most important thing here is creating awareness and also offering those facilities for screening

**Moderator:** And from the community and family level perspective do you think we could do to alleviate this hypertension?

**Respondent:** Is awareness,awareness,awareness and on improving on services delivery ,making sure we have enough community care providers not only at dispensary level starting from the community level ,community health volunteers who can be moving from household checking their BP ,giving them advice on; awareness, risks after capacity building our health care workers and providing basic equipment . BP machine can serve very many household

**Moderator:** From the provider you had talked about some of us you need to have capacity building on health care providers on how to manage hypertension, anything else?

**Respondent:** Capacity building and of course it doesn’t only start ,you see now I’m talking about health care providers one thing I didn’t even talk of is that one of the thing you realize is that the health care providers leave their areas ,the institution ,the training some of them don’t come up well equipped to handle hypertension so for now the national level we are working with all those institution that provide training for health care provider starting with universities,tertially levels like KMTC that is Kenya Medical Training College. We also have faith based organizations, they also have their own training facility. We have trained quite a number of almost half of the lecturers we are incorporating management of cardiovascular condition which include hypertension ,we are reviewing curriculum such that any health care provider who reach our health training today and equipped with adequate knowledge to handle these conditions .So we are going right from the trainings ,whoever trains the students the we go for those who come out without adequate training we train them and even now the broadening the school by increasing the number of service providers particularly the primary health care level that is where we need to put our emphasis on.

**Moderator:** Health system you said you have tried to equip them with equipment and essential medicines which are somewhere available despite the small hitches that you have also talked about the health care providers are also not so many but they can be able to manage the patience they can see. Anything else you would think from the health system level perspective?

**Respondent:** You know when you are talking of health systems you look at the issues holistically. We need to strengthen all those areas starting from the other day we had health budgets, that’s is where we have to start from financing. If we get enough allocation we have to go from health leadership looking at the national level we are lucky the president has recorgnized the importance of sound health but we have other small leaders. 47 county governments have the leadership of those governments understood the importance of financing health in their county like president, health products and technology. I think that you are aware that the ministry of health procured many equipment and took them to the counties now is the counties doing some of the information system very important without data whatever you refer is too you can’t plan. So health information is very important have talked of health workers, have also talked of self-delivery, how do we handle a client, how do we refer them and all that and then we have to make sure that we have adequate health infrastructure.

**Moderator:** So how about the current situation of COVID, how has it affected the provision of hypertensive care in the community

**Respondent:** I think this is open knowledge ,when COVID came on board ,initially most of the health care providers particularly the private ones turned down on the numbers they were reviewing similarly hypertension is normally reviewed in what we call non communicable diseases clinic particularly at the county level. Quit a number of them closed down but we can in hand able to advice the clinician and we have written guidelines and I am not just talking of verbal we had written guidelines on how they are supposed to manage their patience during COVID -19 cause you are aware with these chronic diseases and uncommunicable like hypertension are more likely to be affected by COVID in terms of the infection and the similarities of the infection that the normal population so we have advised that the clinician they do what we call the rescheduling of the clinics such that they make sure that the client will not make frequents visits at the clinic and the client has to go to the clinic they stagger their appointments, they should observe when they come for appointments ,they should observe issues the government is talking about. Social distance, hygiene and once the client gets to the hospital we make sure the client spend the least time I the facilities and that calls for clinician to have looked at the file even before the patient comes to the hospital ,the patient has to go to the hospital when some of them are accompanied by whole family. If you’re accompanied by a person only one person and when they send the patient home they make sure they have At least medication for three months. They give advice on diet those now how to mitigate these factors, how to keep away from tobacco. If one partakes alcohol we are assisting them on how to cease drinking ,how to do physical exercise during this COVID 19 period, how to relate with other relatives at home to avoid physical contact with as many people as possible if possible remain at home avoid social gathering as much as possible, should avoid entertaining visitors to their places. They should only have those visitors they think is necessary, while at home observe highest level of hygiene and then keep on looks on the danger and signs they should be checking their BP regularly as I had earlier indicated most of the patients have these guidelines. Then there are these basic needs they should observe like minor headache, chest pain, and swelling of the body so we have passed all this information to health care provider and patient so they are advised on what to do. And I told you all the clinics open countrywide all what we are doing is to take extra precaution so we continue to provide that essential services

**Moderator:** So is there anything else you would like to add concerning hypertension that we haven’t discussed?

**Respondent:** Let me say we are glad you have taken that field of study, would want more to come and do quite a number of study in the field of hypertension and cardiovascular in general, likewise I think I don’t want to say much about. I think all I would want to call upon is health providers and leaders in general and any other person who is involved or is a stakeholder in health to help us create awareness as far as management of hypertension is concerned. This will go far into improving the health seeking behavior for our people and this will help us detect hypertension early and even also help because the key to managing hypertension is knowing it early and also helping the population know that the are those more defiable risk factors which we can avoid at very early age and when I talk about those key players I health sector. I’m not only referring to health workers I’m also referring to sectors like agriculture whereby we should be able to produce enough food so that we live healthy .I’m talking of education sector where we need to play together to put hands together because you’d find if you go to most of the private schools you’d find a school without playground for children. Physical inactivity starts right from there. We are picking our children right from their doorstep so they don’t even walk to school, PE lessons have been substituted by mathematics or English lessons. We would want when ministry of roads is putting up roads, if you have ever visited Ngong road you’ll find out that Ngong road there is lanes for motorists, bodaboda and bicycles and there is footpath for those who are walking. Most of our roads are like that we even don’t encourage our people to walk or to cycle, you get it?

**Moderator:** yes

**Respondent:** So that’s why I’m saying we need to give issues affect wide approach and that way we would be able to bring hypertension down, the cases or prevalence would bring down and those who have unfortunate developed it because even if we all do this people would still develop hypertension. We create awareness as far as and most important thing is screening, screening, screening then we have a well-equipped health workforce with adequate essential medicines with sound infrastructure that’s it.Thank you.

**Moderator:** Thank you very much for your time and information, we hope whatever that you’ve said and whatever you’re going to get from the study will be of help to the community and country at large

**Respondent:** Thank you very much ,it will be helpful to the study and hope at the end of the day you share this with us, I’ll be grateful to get the results of the study so that we can also give it to improve on our studies.

**Moderator:** Thank you very much.

**…END…**
