# Supplementary material for: A socio-ecological framework examination of drivers of blood pressure control among patients with comorbidities and on treatment in two Nairobi slums; a qualitative study
Source: PLOS Glob Public Health. 2023 Mar 10;3(3):e0001625. doi: 10.1371/journal.pgph.0001625 (PMC10021823; doi:10.1371/journal.pgph.0001625)
Supplement: S3 File — (ZIP) [file pgph.0001625.s003.zip › Policy makers/NRB_KII_PDM_200703_0220 .docx]

**Moderator: {Name}**

**Respondent: {Name}**

**Occupation: Senior Medical Officer**

**Code: NRB-KII-PDM-200703_0220**

**Moderator:**  So confirm that I have read and you have understood the information sheet for the above study. You have had the opportunity to consider the information and ask questions

**Respondent: I confirm**

**Moderator:** Ok, you understand that your participation is voluntary and you are f to withdraw without any of your legal rights being affected

**Respondent: I understand**

**Moderator:** And you understand that the data being collected during the study may be looked at by individuals where it is relevant in taking part in the study and you give permission to these individuals to have access to your data

**Respondent: I agree**

**Moderator:** You confirm consenting to be audio recorded and you also consent to the use of anonymized verbatim quotations

**Respondent: Yes**

**Moderator:** And you are happy for your data to be used in future research

**Respondent: Yes**

**Moderator:** And finally you agree to take part in the above study

**Respondent: Yes**

**Moderator:** Ok. Am going to read to you a short statement and then we head right to the questions

**Respondent: Ok**

**Moderator:** This community has been identified to have a high burden of uncontrolled hypertension which is a leading factor to premature death and disability and we are trying to gather information about provision of hypertensive care in this community particularly to patients on treatment and have their blood pressure not under control

**Respondent: Yes**

**Moderator:** So I’ll be seeking your views in uncontrolled hypertension among those on treatment and the factors that are driving to these high rates and we’ve also noticed that there are several challenges in access and uptake of hypertension care which includes physical, structural policy and financial challenges

**Respondent: Yes**

**Moderator:** So am seeking also your view on uncontrolled hypertension particularly to patients who are on treatment and their pressure is not controlled

**Respondent: Ok**

**Moderator:** So on the first question, in your view, what are the challenges of access and uptake of hypertension care in the community that you serve

**Respondent: The way I think, there is a… I’ll put it in two buckets. There is a person factors**

**Moderator:** Yeah

**Respondent: That contributes to the status of uncontrolled hypertension and there is a system factor**

**Moderator:** Mmmmhh

**Respondent: So for system factors they include access barriers to care**

**Moderator:** Mmmmhh

**Respondent: Access in terms of the physical distance of where the care is provided for hypertension**

**Moderator:** Yeah

**Respondent: There are also financial barriers to access**

**Moderator:** Ok

**Respondent: because for most clients the care is out of pocket**

**Moderator:** Yeah

**Respondent: There is aa, there is also, I would call it supplied service barriers**

**Moderator:** Mmmmhh

**Respondent: Where the providers of the service where they are reachable or it’s affordable**

**Moderator:** Mmmmhh

**Respondent: The providers do not give value for money in terms of care for hypertension to clients**

**Moderator:** Mmmmhh

**Respondent: So the clients perceive as if it’s not worth the service**

**Moderator: Mmmmhh**

**Respondent: On person factors I think there is cultural influence**

**Moderator:** Cultural? Sorry, I can’t get you

**Respondent: Cultural influence for maybe these individuals to receive hypertension care Moderator:** Ok

**Respondent: There is individual willingness to seek care on hypertension**

**Moderator:** Mmmhhh

**Respondent: There is also a general lack of knowledge among the patients in the community**

**Moderator:** Mmmmhh

**Respondent: And then given the nature of condition that the way it manifests, it is essentially painless symptomless condition**

**Moderator:** Mmmmhh

**Respondent: The health seeking behavior of this population generally is scud toward seeking curative care therefore until symptoms show up, they would not prioritize access in care of hypertension**

**Moderator:** Ok so you have talked about the physical distance challenge to the facilities in your area. So what of the structural and policy guidelines of hypertension in the facilities you are serving

**Respondent: Come again**

**Moderator:** What of the policy. What challenges do you have with the policies for the guidelines for hypertension?

**Respondent: So there is…Eeehh I’ll call it unapparent lack of harmony in policies of the ministry**

**Moderator:** Mmmmhh

**Respondent: And the general programming within the ministry with consequence detrimental effect on care for hypertension**

**Moderator:** Mmmmhh

**Respondent: Where you find that up until late last year, the access to health products for hypertension were limited to certain level of health facilities basically from level four and above. Secondary care and above**

**Moderator:** Mmmmhh

**Respondent: Where at the same time the guidelines for care for hypertension were promoted cascading care for hypertension to the lowest level like level 2and 3**

**Moderator:** Mmmmhh

**Respondent: So by discordance what the guidelines for care are providing for and what can these facilities access products to provide the care contributed to some system side in providing improved access to hypertension care**

**Moderator:** Ok. So what of the financial challenges you have

**Respondent: As a ministry?**

**Moderator:** Yeah

**Respondent: Well there is challenge in the programming for hypertension which same for limited resources and the resources are financial and also technical and these lies both in national and county government**

**Moderator:** Ok

**Respondent: So you find that at the national level there are no dedicated budgets for programming for hypertension despite being condition that has high prevalence in the country**

**Moderator:** Ok

**Respondent: Further there is as is now there is no dedicated program officer and that translates down to the county**

**Moderator:** Ok

**Respondent: Yeah**

**Moderator:** And are there any challenges in terms of expertise, in health care providers and equipment **for** high blood pressure monitoring

**Respondent: Yes there is a challenge in both respects. There is challenge in terms of knowledge**

**Moderator:** Yeah

**Respondent: Of the providers to actually to prevent, detect and to care for hypertension**

**Moderator:** Hello, your network is not so ok

**Respondent: Is it?**

**Moderator:** Yeah, it’s a bit shaky

**Respondent: On my side I don’t know let me see, actually no. am not on Wifi. Is it better now?**

**Moderator:** Yeah its better

**Respondent: Ok. So I was saying there is a low level of knowledge which is generalized across the board for example human resource in terms of preventing, detecting and managing hypertension**

**Moderator:** Mmmmhh

**Respondent: There are obviously variances depending on where you are but what is a consistent is that it doesn’t matter whether you are in rural or urban setting, the level of knowledge is wanting**

**Moderator:** Mmmmhh

**Respondent: And unfortunately it’s not even tone carder, it cuts across the board**

**Moderator:** Ok

**Respondent: And there is also lack or shortage of tools and equipment to provide services for hypertension**

**Moderator:** Mmmmhh

**Respondent: It’s been our experience but I believe things are improving coz I have experienced that majority of health facilities do not even triage clients that are coming in so even them they do not ever pick up opportunistic presentation of hypertension**

**Moderator:** Ok

**Respondent: And that T… (Not clear) is very basic equipment, these translate to any other equipment for other health services that pertain hypertension**

**Moderator:** Ok

**Respondent: There are no weighing scales, height meters are not available and other equipment for screening for other cardio vascular diseases**

**Moderator:** So we are going to combine staffing challenges and about the capacity and work load of employees providing this care. What challenges do you have with that?

**Respondent: In our experience, the feedbacks we get from counties is that there is generally critical health workers shortage**

**Moderator: Yeah**

**Respondent:** And that is their report but from our own observation we have found out that in the health **facilities that provide hypertension care the clinics tend to be very under staffed**

**Moderator:** Yeah

**Respondent: And at the organization of providing service tends to lean towards overwhelming the staff that is there**

**Moderator: Mmmmhh**

**Respondent: Because the current organization is that the patient tend to be cooled**

**Moderator: Mmmmhh**

**Respondent: Like there is a clinic once in every two weeks so the provider get mass of patients to come in on a certain day**

**Moderator: Mmmmhh**

**Respondent: And that impact on the quality of care provided and also impact on the perception that there is health worker shortage**

**Moderator:** Ok

**Respondent: In terms of. What was the other part of that question?**

**Moderator:** The capacity and work load of employees

**Respondent: Yes, so along the same grill, there is no dedicated… to begin with**

**Moderator:** Mmmmhh

**Respondent: There is low level of knowledge, there is no structured continued capacity building around this area of hypertension**

**Moderator:** Mmmmhh

**Respondent: And even where some staff are trained they tend to be… there is high turnover of staff within the facility and also external to the facility so even when training is done**

**Moderator:** Mmmmhh

**Respondent: At the end of the day it tends not to have an impact because the staff that are trained don’t provide the service where they are trained. They are employed elsewhere or they look for greener pastures**

**Moderator:** Ok. And any challenge related to facility working hours?

**Respondent: Aaahh…to my understanding, in public sectors the standard is that all health facilities are open 24 hrs. but from our observation we find that hypertension services tend to be provided on specific days and mostly during the morning hours**

**Moderator: Ok**

**Respondent: So that is when clients are advised to present themselves for the clinic and once that window is lapsed then it is like the service is no longer available**

**Moderator:** Ok

**Respondent: So that contributes to crowding that is experienced during clinic days**

**Moderator:** Ok

**Respondent: Aaaahh…i believe in the private sector the organization is different so i have no comment on how that impact the quality of care and the work load of the health workers**

**Moderator:** Ok. And do you have any challenges related to medication stock out?

**Respondent: I would say yes. This is as reported by health workers and even pharmacists in the institutions that we’ve been able to visit**

**Moderator:** Yeah

**Respondent: They cry of supply chain challenges consistently and there is no pattern as it is**

**Moderator:** Mmmmhh

**Respondent: Sometimes it’s for one product, sometimes for all products**

**Moderator:** Mmmhhh

**Respondent: Yeah but what’s constants that there is frequent stock out of medication**

**Moderator:** Ok

**Respondent: And it doesn’t matter, it could be a level 5 facility, it could be a level 3**

**Moderator:** Ok. So in your opinion, what can be done to alleviate the access and uptake challenges of uncontrolled hypertension care? We will talk about this in different perspectives and from an individual perspective or patient’s perspective. What do you think we should do to alleviate access and uptake challenges of uncontrolled hypertension?

**Respondent: That’s from the client’s perspective?**

**Moderator: Yeah**

**Respondent: I believe the foremost thing would be to secure the availability of the service**

**Moderator: Yeah**

**Respondent: For this clients that it will not be a guessing game whether or not if they show at the clinic they would actually get the full service that they seek**

**Moderator: Ok**

**Respondent: But am also positive that the patients will appreciate some enhancement of financing around hypertension in terms of lessening toehold or the burden of paying for services to manage the hypertension**

**Moderator: Mmmmhh.Ok and what of from the family level perspective what are the things that we should alleviate to access the challenge of uncontrolled hypertension?**

**Respondent: At the family level i would… I would start with intensive and sustained education of all community members**

**Moderator: Mmmmhh**

**Respondent: on the condition, the prevention and care for it coz depending on who the client is , some decision to spend family income on thecae of the condition largely depends on the buy line of the decision maker**

**Moderator: Ok**

**Respondent: So I would priorities sustained and good quality education for the community members**

**Moderator: Mmmmhh**

**Respondent: To enable clients manage the condition**

**Moderator: Ok. And what of from the providers perspective. From the health care provider perspective, what do you think are the factors that should alleviate the challenges of access and uptake?**

**Respondent: From the providers perspective i think the providers would really appreciate systems or structures that enable better follow up without necessarily adding workload to these providers**

**Moderator: Mmmmhh**

**Respondent: Because the current organization on how services are provided is at in between visit which can take period of between 6 and 3 months**

**Moderator: Mmmmhh**

**Respondent: if there is no contact for clients so if there were, i believe if there were systems or structures that would enable some feedback from the clients on their status in order to catch any setbacks or any alarms in the course of these patients medication journey before the next visit**

**Moderator: Mmmmhh**

**Respondent: I think it will real help coz the main concern for health workers in terms of improved health care would be what does that impact on the work load**

**Moderator: Ok. And from the health system perspective, what do you think we should do?**

**Respondent: From the health system enable number one integration of programs**

**Respondent: Aaahh… i strongly believe that the facilitation we have in terms of providing care across all conditions is fueled by the vertical programs that exist and the programs were integrated then we wouldn’t need dedicated …. ( Not clear) for providing care for hypertension and things like that.it would then require that at any opportunity that someone interacts with the health system then this critical or high burdened conditions are addressed or at least enquired about to minimize the late presentation which is currently the state**

**Moderator:** Mmmmhh and from the policy level perspective, what do you think from the position that you are in. what do you think are the things that would alleviate the access and uptake challenges for uncontrolled hypertension

**Respondent: From policy level I think first there has been quite some movement on the right direction advocating for hypertension control and prevention in terms of Kenya health policy this is one of the policy objectives and i believe in now expired strategic plan, hypertension related indicators were included the strategic plan**

**Moderator:** Mmmmhh

**Respondent: But where the gap comes in terms of seeing through, there is limitation of strategies and guidelines and therefore the MMEs involved implementing this. so on the MMEs side is a real gap**

**Moderator:** Mmmmhh

**Respondent: There have been strides made especially with formation of National Health Systems there is a tool to collect …. (Not clear) data on hypertension from a patients level but them there has not been enough support ensure that this tools are full utilised across the country. so the existing the system partly think only half the country in terms counties know that this tools exist and how they are to be used and actually put them to use**

**Moderator:** Ok. So about the current situation of COVID

**Respondent: Yeah**

**Moderator:** How has it affected the provision of hypertensive care in the community that you serve?

**Respondent: I believe that even the containment measure that were issued and the drive to tell people to stay at home**

**Moderator:** Mmmmhh

**Respondent: A lot of interpreted that as them not to making trips to attend the clinic**

**Moderator:** Yeah

**Respondent: But on the same hand, health facilities allusion the name of debunking or removing patients from the hospital, they shut down selective or cold clinics**

**Moderator:** Ok

**Respondent: So this kind of resorted into there being interruption in the provision of service of hypertension**

**Moderator:** Ok. And is there. We are on the last question. is there anything else you would like to talk about hypertension care and we have not discussed

**Respondent: Yes, I would say we are not creating demand enough for the prevention and the control of hypertension**

**Moderator:** Mmmhhhh

**Respondent: I think there is a lot that can be done by all stake holders that is the government, civil service and the users of the service**

**Moderator:** Mmmmhh

**Respondent: There is a lot they need to put in to stem the surge of current hypertension care**

**Moderator:** Mmmmhh

**Respondent: There is a lot that can be done in terms of advocacy**

**Moderator:** Ok, Thank you very much for your time and i think the information that you have given me will be of importance to the community and the country at large on hypertension care and the patients who are suffering from hypertension. So we will share the information after the study is done and we will get to know where the gap is

**Respondent: Ok**

**…END…**
