# Supplementary material for: A socio-ecological framework examination of drivers of blood pressure control among patients with comorbidities and on treatment in two Nairobi slums; a qualitative study
Source: PLOS Glob Public Health. 2023 Mar 10;3(3):e0001625. doi: 10.1371/journal.pgph.0001625 (PMC10021823; doi:10.1371/journal.pgph.0001625)
Supplement: S3 File — (ZIP) [file pgph.0001625.s003.zip › Policy makers/RUA_KII_PDM_200701_2332.docx]

**Moderator: {Name}**

**Respondent:** {Name} **Code: RUA-KII-PDM-200701_2332.mp3

Moderator:** You confirm that i have read and you have understood the information about the study and i have given you the opportunity to consider the information and ask questions and I have answered these questions satisfactorily

**Respondent:  Ok. Yes**
**Moderator**: You understand also that your participation is voluntary and that you are free to withdraw at any time without giving any reasons, with our your legal rights being affected and you understand your data collected during this study may be looked at by individuals where it is relevant to your taking part in this study. You give permission to these individuals to access your data?
**Respondent:** **Moderator:** Yes
**Respondent:** And you confirm...
**Moderator:** That’s what am sharing. Yeah?
**Respondent:  Ok
Moderator:** You confirm consenting to be audio recorded and you also consent to the use of anonymized verbatim quotations **Respondent: Yes
Moderator**: And you are happy for your data to be used in future research
**Respondent: Yes. That’s fine, that’s fine
Moderator**: Finally you agree to take part in the above study
**Respondent: Yes**
**Moderator:** Ok. On to the study it self **Respondent: Ok
Moderator:** There are several challenges in access and uptake of hypertension which include physical, structural and financial challenges **Respondent: Yes
Moderator:** So I’ll be seeking your views on uncontrolled hypertension particularly among those who are on treatment **Respondent: Ok
Moderator:**  So in your views, what are the challenges in access and uptake of hypertension in the community you serve **Respondent: Sorry
Moderator:** In your view, **Respondent: Mmmmhh
Moderator:** What are the challenges in access and uptake of hypertension care in the community you serve **Respondent: That’s for already screened and diagnosed cases?
Moderator:** Yeah, the patients who are on treatment but still not controlled **Respondent: It is probably the monitoring**. **The monitoring bit because many times you will find that somebody is diagnosed but now the monitoring and follow up many medical general clinics has not been embraced. It’s not as consistent and I think that’s the problem. That’s majorly the problem
Moderator:** Mmmmhhh **Respondent: I think because follow up will entail maybe follow up on lifestyle modification, adherence to treatment
Moderator:** Mmmhh. Ok **Respondent: Check on control whether the blood sugar is… the blood pressure is being controlled probably …. (Not clear) treatment
Moderator:** Mmmmhh
**Respondent: I think that has been lacking quite a bit
Moderator:** Ok **Respondent: And of course access to facility in terms of maybe laboratory tests
Moderator:** Mmmmhh **Respondent: I think it could be a challenge in regards to accessing in terms of facilities, lab maybe availability of reagents probably or maybe equipment for that matter
Moderator:** Mmmmhh **Respondent: Yes
Moderator:** Ok. What of for the physical. The number of public facilities in the area, do you have a challenge with that?

**Respondent: Yeah, of course we would like to have easier geographical access
Moderator:** Mmmmhh
**Respondent: And coupled also with expertise and infrastructure
Moderator:** Ok **Respondent: Because human resource is also important. If you don’t have somebody who is well trained or well skilled to handle then still you wouldn’t say that is adequate
Moderator:** Ok

**Respondent: Yes
Moderator:** What of from the policy level, policy or clear guidelines for hypertension care. Do you have any challenge with that?  **Respondent: Aaahh, maybe the decentralization or maybe the cascading of the guidelines
Moderator**; Mmmhhh

**Respondent: It should be to quite a bigger number of health care workers we have not managed to cascade it to the required number. We have yes guidelines , we have policies but now sharing or sending this information to the people who should be taking care of this patients probably may need to know more
Moderator:**  Ok **Respondent: Yes
Moderator:** What of the financial allocation to hypertension care **Respondent: We have not had focus or maybe support skewed towards this
Moderator:** Mmmmhh

**Respondent: Probably we can only look at it in terms of a bit of capacity building which we wouldn’t say that it’s enough and course commodity we have the government supply but still especially now for agents and those testing facilities….(Not clear) because some of his monitoring equipment sometimes they breakdown and my need some maintenance and things
Moderator:** Any challenges with staffing? **Respondent: Of course we always have shortages of staffs as per the standard norms
Moderator Mmmmhhh**

**Respondent: Yeah. I think basically that is one and number two build them in capacity skillfully to manage patients
Moderator:** Ok. So I think the challenge of staffing can also go with how about capacity of workload on employees that are providing this care **Respondent: Of course workload is high
Moderator:** Yeah
**Respondent: Because sometimes if he workload is high then you are not able to screen appropriately
Moderator:** Mmmmhh

**Respondent: The patients for you to be able to capture people who are maybe sick and they don’t know that they are sick
Moderator:** Ok. And what are the challenges related to facility working hours in your area **Respondent: Of course now there is access of the facilities over the weekend and at night
Moderator:**  Mmmhhh

**Respondent: So this complications may come at night so the success is limited
Moderator** Mmmmmhh
**Respondent: So access on time can only be from 8-5 and Monday to Friday, outside those hours you may not get access to care
Moderator:** Mmmmhh **Respondent: Yes
Moderator:** Do you have any challenges related to medical such as medication stock out in your area?

**Respondent: Aaaahhh...Not recently
Moderator:** Mmmmhh
**Respondent: We’ve had it before but recently it’s not a bid big challenge
Moderator:** Ok. S at the moment currently you have stock?

**Respondent: I would say we are doing better
Moderator**: Ok. So what in your opinion can be done in this community to alleviate the uptake and access challenges of uncontrolled hypertension care? We are going to talk this in different perspectives. So now from the patient’s perspective, what do you think can be done in this community to alleviate the access and uptake of uncontrolled hypertension **Respondent: Of course communication, I mean sensitization to the community decency deeds and how take care of them
Moderator:** Mmmmhh **Respondent: Of course preventive promotive and for the ones who are already sick on the essence of follow up
Moderator:** Mmmmhh

**Respondent: Basically is communication to the patients, that’s the biggest thin that would be intervention
Moderator:** Ok. Anything else you would lie to add to that? **Respondent: Aaaaahh. Of course encouraging them to screening and checkups and routine follow ups especially if you’ve been diagnosed
Moderator:** Mmmmmh

**Respondent: Yes
Moderator**: From the community and family level perspective, what do you think can be done to alleviate this? **Respondent: Aahhhh, community maybe we may have the of course through community strategy people support especially the ones who have had complications which may incapacitate in one way or the other
Moderator:** Mmmmmh **Respondent: Involvement of community strategy maybe the community health volunteers and at family level, family supports
Moderator:** Mmmmmh

**Respondent: In the various aspects which could try and maybe mitigate or maybe improve the outcome of some of these diseases
Moderator:** Ok. From the provides perspective, what do you think can be done? **Respondent: providers for one is increasing the staff numbers, number 2 maybe accesses to facilities and infrastructure
Moderator:** Mmmmmh

**Respondent: Number 3, capacity building on things that will focus on all those challenges that may have … (Not clear) to
Moderator: Ok. From the health systems level, what do you think can be done there?
Respondent: Of course more focus. More focus especially at the primary health care level
Moderator:** Mmmmmh **Respondent: Or we have guidelines, yes we have the good will from the government and of course the support. Financial support as a pillar for every system I think its important
Moderator:** Mmmmhh

**Respondent: That support needs to be there. It will come with more training, more staff
Moderator:** Ok. From the policy level? **Respondent: Policy level of course maybe increased research coz we rely so much on information coming out probably not in our set up
Moderator:** Ok

**Respondent: I thin research operations and other sectors I think it’s important.
Moderator:** Mmmmhhh
**Respondent: That’s one thing that we need to be keen on.
Moderator:** And how has the COVID situation affected your provision of care to hypertensive patients in the community you serve?
**Respondent: Of course initially we had numbers go down. They took a deep because of the fear. We haven’t managed to go back to our normal
Moderator:** Yeah

**Respondent: So even follow up for the kind of patients is still not as it was
Moderator:** Mmmmhhh
**Respondent: I think that’s one thing we are observing at the health care facilities
Moderator**: Is there anything else you would like to talk about in relation to hypertension care which we have not discussed

**Respondent: Aaahh… in which view?
Moderator:** Anything that we’ve not discussed about hypertension and these clients that have uncontrolled hypertension **Respondent: Maybe integration of hypertension diabetes care
Moderator:** Yeah **Respondent: In the system
Moderator:** Mmmmhh

**Respondent: The fact that we are treating it so much as a separate service delivery. It needs to be much integrated the more integrated the more we will be able to do the follow so that we can have these people even if it through presenting promotive immunization or whenever …(Not clear) That way we will be able to do screenings so that we can diagnose people earlier and have an intervention
Moderator:** Thank you so much for your information {occupation}and I hope the information you have given us will help us as a community and the country at large
**Respondent: yes
Moderator:** So thank you very much for your time

**…END…**
